# Supplementary material for: Site- and enantioselective cross-coupling of saturated N-heterocycles with carboxylic acids by cooperative Ni/photoredox catalysis
Source: Nat Commun. 2023 Jan 9;14:125. doi: 10.1038/s41467-023-35800-0 (PMC9829739; doi:10.1038/s41467-023-35800-0)
Supplement: Supplementary file 1 — Supplementary Information [file 41467_2023_35800_MOESM1_ESM.pdf]

Site- and enantioselective cross-coupling of saturated *N*-heterocycles with carboxylic acids by cooperative Ni/photoredox catalysis

Xiaomin Shu, De Zhong, Qian Huang, Leitao Huan, and Haohua Huo*

State Key Laboratory of Physical Chemistry of Solid Surfaces, Key Laboratory of Chemical Biology of Fujian Province, and College of Chemistry and Chemical Engineering, Xiamen University, Xiamen 361005, China

E-mail: hhuo@xmu.edu.cn

X.S. and D.Z. contributed equally to this work.

Supplementary Information

Table of Contents

I. **Supplementary Methods** S–2

1.1 General Information S–2

1.2 Preparation of Chiral Ligand (*S*, *R*)-L1 S–3

1.3 Optimization of Reaction Conditions S–4

1.4 Catalytic Enantioselective α-Acylation of Saturated Azacycles S–11

1.5 Synthetic Utility S–46

1.6 Mechanistic Studies S–56

1.7 Assignment of Absolute Configuration S–63

1.8 ^{1}H-NMR, ^{13}C-NMR, and ^{19}F-NMR Spectra S–65

1.9 Stereoselectivity Analysis S–143

II. **Supplementary References** S–200

I. Supplementary Methods

1.1 General Information

Unless otherwise noted, reactions were performed with rigorous exclusion of air and moisture. *N*-protected amines were prepared according to a literature procedure, and all analytical data matched that report.[1] Anhydrous PhH (99%, Energy Chemical, extra dry, with molecular sieves, Water≤50 ppm (by K.F.), EnergySeal). Anhydrous *i*-PrOAc (99.5%, Energy Chemical, extra dry, with molecular sieves, Water≤50 ppm (by K.F.), EnergySeal). $NiCl_2$·glyme (97%, Strem), $NiBr_2$·glyme (97%, Strem), NH_4Cl (99.999%, Alfa Aesar), Na_2HPO_4 (>99.0%, Sigma-Aldrich), K_2HPO_4 (>99.95%, Sigma-Aldrich), $NaHCO_3$ (>99.7%, Sigma-Aldrich), 2,6-lutidine (98%, Alfa Aesar), and DMDC (Rhawn), and all commercially available carboxylic acids (Alfa Aesar, Energy Chemical, TCI, and Sigam-Aldrich) were used as received.

NMR spectra were collected on a Bruker 400 MHz, a Bruker 500 MHz, or a Varian 500 MHz spectrometer at ambient temperature. HPLC analyses were carried out on an Agilent 1260 series system with Daicel CHIRALPAK® or Daicel CHIRALCEL® columns (4.6 × 250 mm, particle size 3 μm). FT-IR measurements were carried out on a Nicolet AVATER FTIR330 spectrometer. High resolution mass spectra (ESI) were recorded by the instrumentation center of Department of Chemistry, Xiamen University, on a high-resolution LC/MS instrument. Emission intensities were recorded on a Hitachi F7000 fluorescence spectrophotometer in a 10.0 mm quartz cuvette. Optical rotation data were obtained with an Anton Paar MCP 500 polarimeter at 589 nm and at 25 °C, using a 50 mm path-length cell in the solvent and at the concentration indicated. GC analyses were obtained on an Agilent 6890A GC. Flash column chromatography was performed using silica gel (200–300 mesh). Blue LED lamps (40 W; Kessil PR160L) were used to irradiate the reaction mixtures.

1.2 Preparation of Chiral Ligand (*S*, *R*)-L1

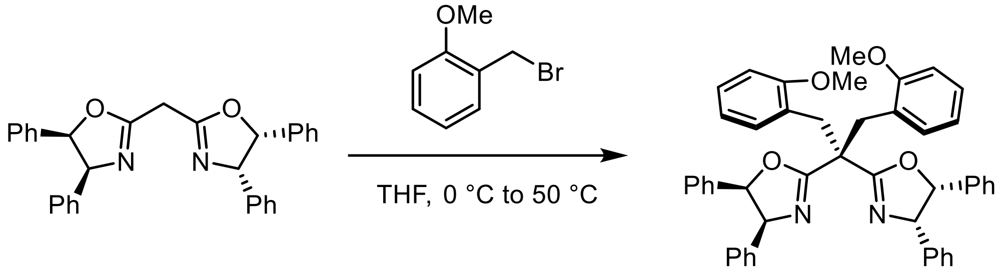

To a solution of bis((4*S*,5*R*)-4,5-diphenyl-4,5-dihydrooxazol-2-yl)methane (2.29 g, 5.0 mmol, 1.0 equiv) in THF (50 mL) was added NaH (60% in oil) (0.60 g, 15.0 mmol, 3.0 equiv) slowly in potions at 0 °C in a 250 mL of round bottom flask. The mixture was stirred at 0 °C for 0.5 h under N_2 atmosphere. Next, the 1-(bromomethyl)-2-methoxybenzene (3.02 g, 15.0 mmol, 3.0 equiv), dissolved in dry THF (10 mL), was added dropwise to the flask, and the mixture was stirred at 50 °C for another 12 h. Then, saturated brine was added carefully to quench the reaction. The solvent was evaporated under reduced pressure and the residue was further extracted with dichloromethane. The combined organic layer was dried over Na_2SO_4, filtered, and concentrated under vacuum. The residue was purified by flash column chromatography on silica gel (1:5 EtOAc/ Petroleum ether) to yield (*S*, *R*)-**L1** as a white solid (3.29 g, 94% yield).

^{1}H NMR (500 MHz, $CDCl_3$) δ 7.64 (d, *J* = 7.5 Hz, 2H), 7.30 – 7.24 (m, 2H), 7.01 – 6.86 (m, 24H), 5.65 (dd, *J* = 10.0, 3.0 Hz, 2H), 5.43 (dd, *J* = 10.0, 3.0 Hz, 2H), 3.94 – 3.78 (m, 10H). ^{13}C NMR (126 MHz, $CDCl_3$) δ 168.62, 158.44, 137.63, 136.10, 132.17, 128.14, 127.85, 127.41, 127.38, 127.10, 126.90, 126.72, 126.30, 120.08, 110.29, 86.03, 73.73, 55.36, 48.92, 34.19.

FT-IR (film): 2934, 2835, 1652, 1494, 1245, 1117, 1027, 754 cm^{-1}.

HRMS (ESI-MS) *m/z* $[M+H]^+$ calcd for $C_{47}H_{43}N_2O_4$: 699.3217, found: 699.3212.

$[\alpha]^{25}_D$ = –333.4 (*c* 1.0, CH_2Cl_2).

1.3 Optimization of Reaction Conditions

General Procedure A (GP-A): In a glovebox, Ir[dF(CF_3)ppy]$_2$(dtbbpy)PF_6 (1.1 mg, 0.001 mmol, 1 mol%), $NiCl_2$·glyme (2.2 mg, 0.010 mmol, 10 mol%), (*S*, *R*)-**L1** (9.1 mg, 0.013 mmol, 13 mol%), (4'-hexyl-[1,1'-biphenyl]-4-yl)(pyrrolidin-1-yl)methanone (**1**) (100.6 mg, 0.30 mmol, 3.0 equiv), a Teflon stir bar, and anhydrous benzene (1.0 mL) were added sequentially to a 4-mL vial. The vial was sealed with a septum cap and wrapped with electrical tape. The reaction mixture was stirred at room temperature for 30 min, after which it turned to a pale-yellow solution. Next, a solution of the 4-methoxy-4-oxobutanoic acid (**2**) (100 μL, 13.2 mg, 0.10 mmol, 1.0 equiv) was added via a 100-μL microsyringe. Then, 2,6-lutidine (17.5 μL, 0.15 mmol, 1.5 equiv) and DMDC (16.0 μL, 0.15 mmol, 1.5 equiv) were added sequentially via microsyringe. Next, the vial was transferred out of the glovebox, and then vacuum grease was liberally applied to cover the entire top of septum cap. Then, the reaction mixture was stirred at 20 °C in an EtOH bath for 1 min before being irradiated with a 40 W the blue LED lamp (Kessil PR160L, 427 nm). The reaction was stirred under blue LED irradiation at 20 °C for 24 hours. Next, the lamp was turned off and then tetradecane (26.0 μL, 0.10 mmol) was added as an internal standard. The mixture was filtered through a small plug of silica gel, which was flushed with acetone (~6 mL). A portion of the filtrate (0.1 mL) was diluted with acetone (total volume: 1 mL) and analyzed via GC, and the remainder of the filtrate was concentrated via rotary evaporation, and the pure product was isolated by preparative TLC on silica gel (1:6 Acetone/petroleum ether).

The results for the optimization of the reaction conditions were revealed in Table 1 and Table S1-S7. **GP-A** was followed, using the corresponding carboxylic acid (0.10 mmol) and saturated *N*-heterocycle (0.30 mmol). The yield and regioselective ratios were determined via GC analysis with tetradecane as an internal standard. The ee values were determined via HPLC analysis after purification by preparative thin-layer chromatography.

Note: Although the reaction is not highly sensitive to oxygen and can be performed under air in a capped 4-mL vial (as show below), to ensure the reproducible results, the coupling is recommended to set up using a glovebox, as described above.

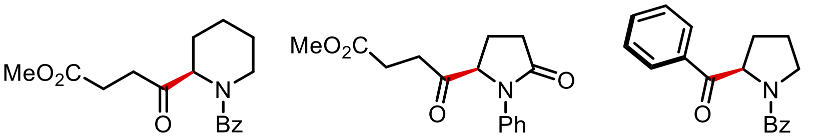

	**18**	**22**	**36**
air-free conditions:	64% yield, 95% ee	84% yield, 88% ee	83% yield, 96% ee
under air in a capped vial:	68% yield, 93% ee	85% yield, 86% ee	85% yield, 92% ee

Supplementary Table 1. Effect of ligand for the α-acylation of *N*-Bz-piperidine.

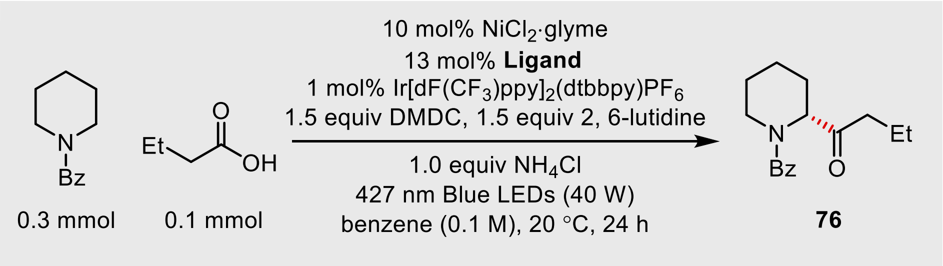

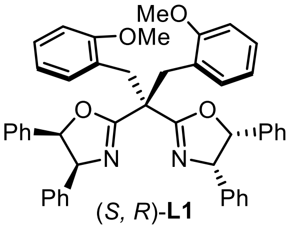

(*S, R*)-**L1**
72% yield, 90% ee

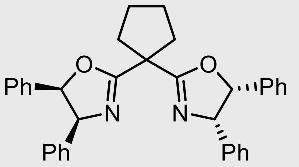

(*S, R*)-**L2**
81% yield, 91% ee

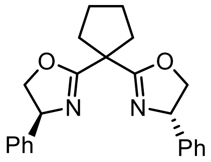

(*S*)-**L3**
87% yield, 81% ee

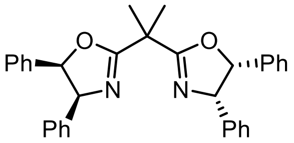

(*S, R*)-**L9**
46% yield, 66% ee

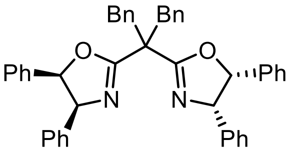

(*S, R*)-**L10**
72% yield, 84% ee

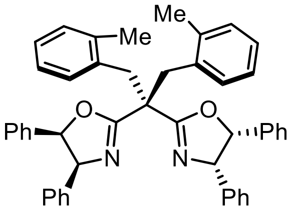

(*S, R*)-**L11**
65% yield, 88% ee

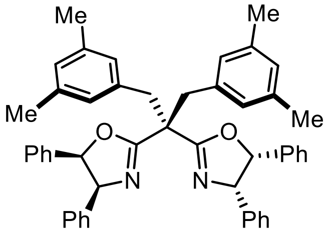

(*S, R*)-**L12**
61% yield, 91% ee

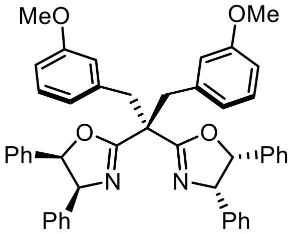

(*S, R*)-**L13**
74% yield, 82% ee

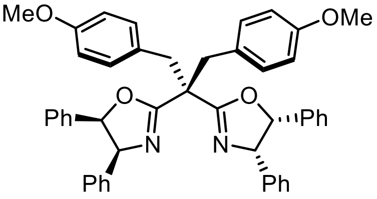

(*S, R*)-**L14**
79% yield, 86% ee

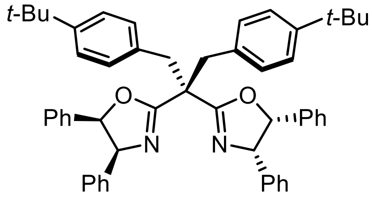

(*S, R*)-**L15**
77% yield, 90% ee

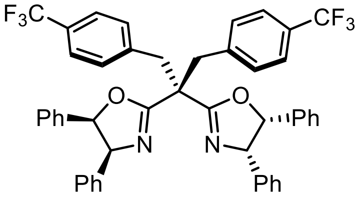

(*S, R*)-**L16**
22% yield, 81% ee

The yield was determined by GC analysis with tetradecane as internal standard. The ee value was determined by HPLC analysis.

Supplementary Table 2. Effect of base.

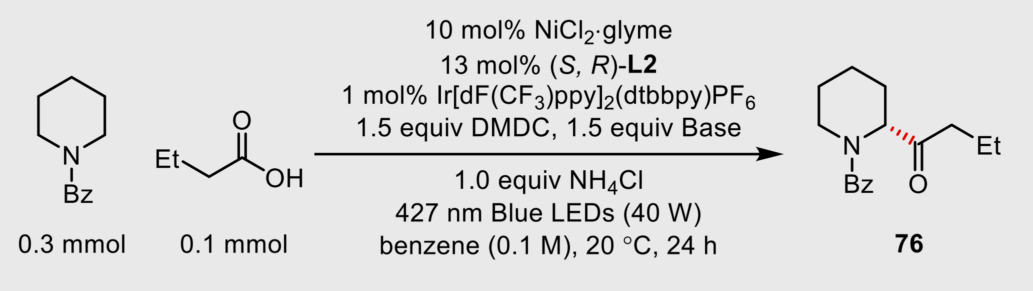

entry	Base	yield (%)	ee (%)
1	2,6-lutidine	82	90
2	Na_3PO_4	68	64
3	Na_2HPO_4	90	79
4	K_3PO_4	3	-
5	K_2HPO_4	89	70
6	Na_2CO_3	40	70
7	$NaHCO_3$	89	69
8	K_2CO_3	81	65
9	$KHCO_3$	0	-

The yield was determined by GC analysis with tetradecane as internal standard. The ee value was determined by HPLC analysis.

Supplementary Table 3. Effect of solvent.

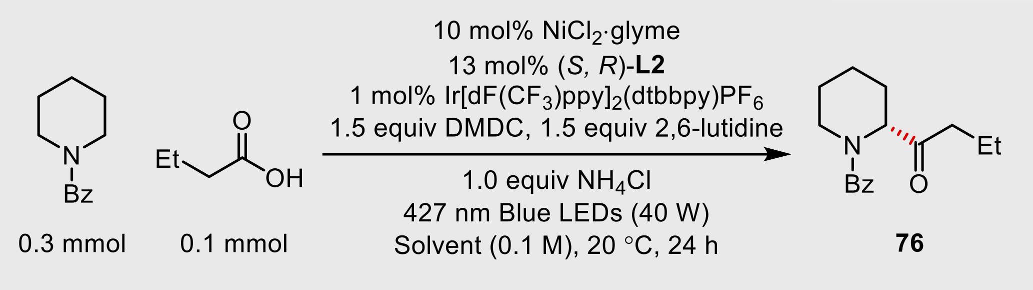

entry	Solvent	yield (%)	ee (%)
1	Benzene	82	90
2	MeOAc	37	82
3	EtOAc	61	84
4	^{i}PrOAc	76	87
5	^{t}BuOAc	21	-
6	MTBE	66	74
7	CPME	10	-
8	$PhCF_3$	21	89

The yield was determined by GC analysis with tetradecane as internal standard. The ee value was determined by HPLC analysis.

Supplementary Table 4. Effect of ligand for the α-acylation of *N*-Bz-pyrrolidine.

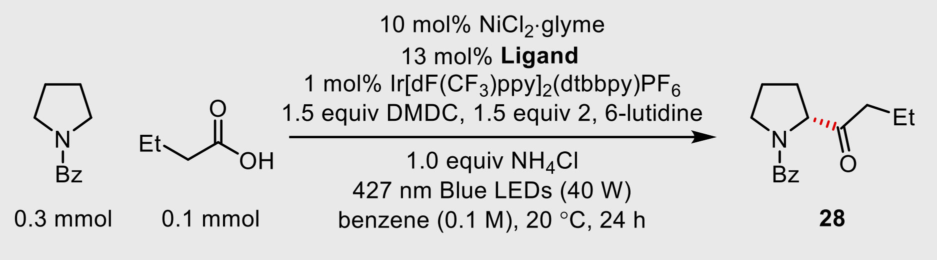

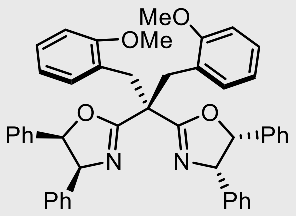

(*S*, *R*)-**L1**
74% yield, 94% ee

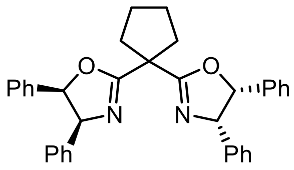

(*S*, *R*)-**L2**
71% yield, 86% ee

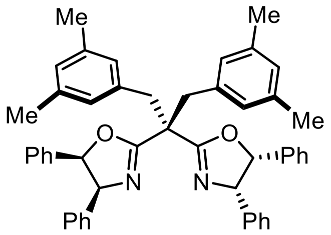

(*S*, *R*)-**L12**
68% yield, 93% ee

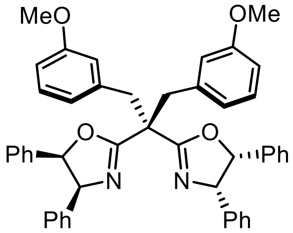

(*S*, *R*)-**L13**
60% yield, 89% ee

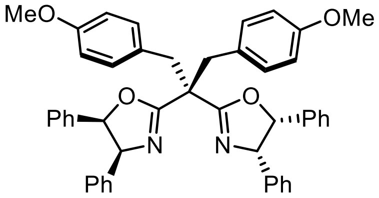

(*S*, *R*)-**L14**
62% yield, 89% ee

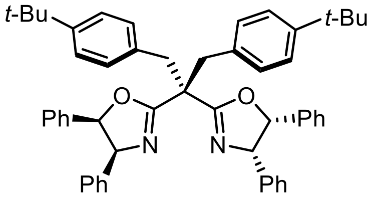

(*S*, *R*)-**L15**
69% yield, 91% ee

The yield was determined by GC analysis with tetradecane as internal standard. The ee value was determined by HPLC analysis.

Supplementary Table 5. Effect of additive.

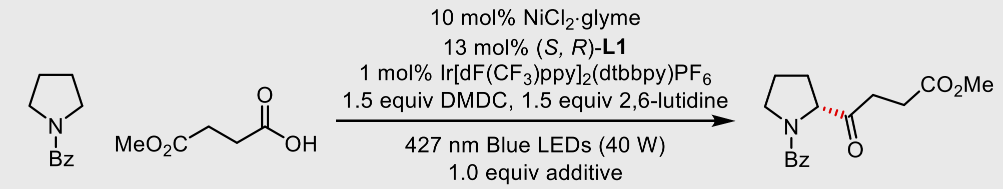

13

entry	additive	additive remaining	yield, ee
1	none	NA	79, 97
2	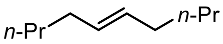	81	74, 96
3	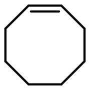	87	70, 96
4	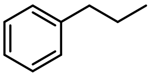	87	78, 94
5	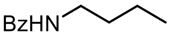	99	73, 94
6	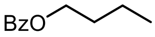	93	80, 95
7	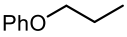	90	77, 95
8	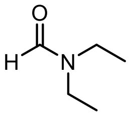	90	77, 94
9	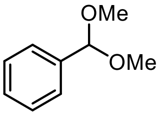	90	78, 95
10	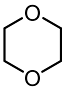	93	79, 94
11	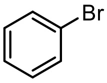	82	75, 95
12	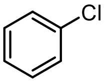	100	83, 95
13	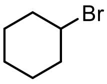	99	80, 95
14	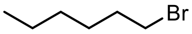	92	78, 95

The additive remaining and yield were determined by GC analysis with tetradecane as internal standard. The ee value was determined by HPLC analysis.

Supplementary Table 6. Effect of HAT catalysts.

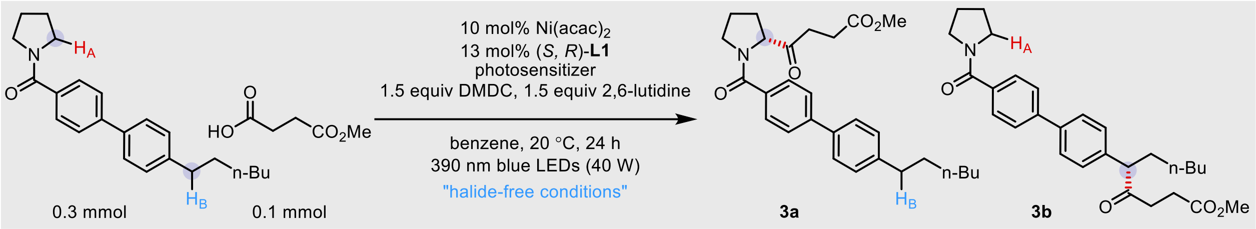

entry	photosensitizer	yield (%)	r.r. (**3a**/**3b**)	ee of **3a** (%)
1	1 mol% Ir[dF(CF_3)ppy]$_2$(dtbbpy)PF_6	4	-	-
2	1 mol% Ir[dF(CF_3)ppy]$_2$(dtbbpy)PF_6, 25 mol% DMBP	4	-	-
3	1 mol% Ir[dF(CF_3)ppy]$_2$(dtbbpy)PF_6, 25 mol% quinuclidine	0	-	-
4	5 mol% TBATD	0	-	-
5	25 mol% **H2-H9** (as shown below)	as shown below	-	as shown below

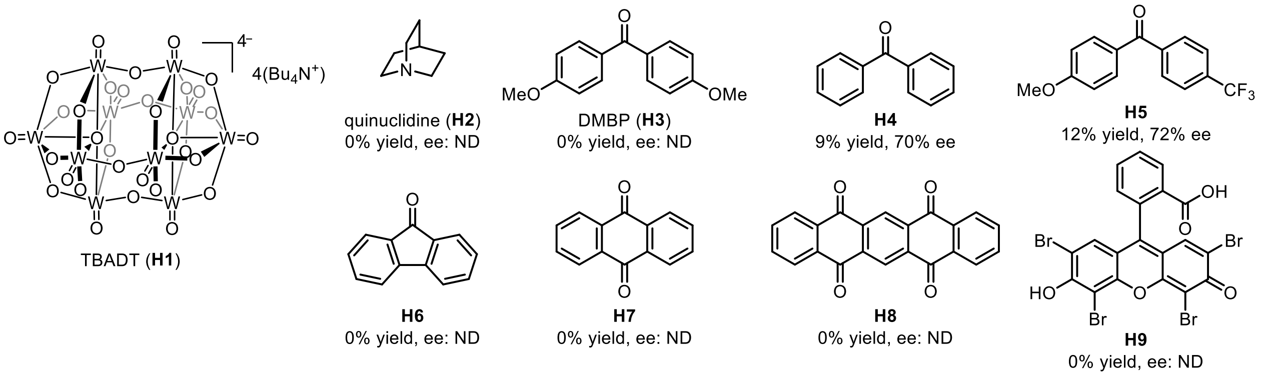

The yield and regioselective ratios were determined by GC analysis with tetradecane as internal standard. The ee value was determined by HPLC analysis.

Supplementary Table 7. Effect of acyl surrogates.

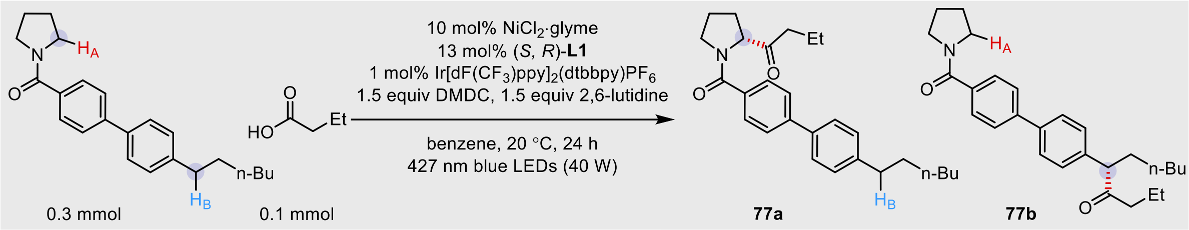

entry	variation from standard conditions	yield (%)	r.r. (**77a**/**77b**)	ee of **77a** (%)
1	none	56	> 20:1	87
2	**C1**, instead of (acid+DMDC)	50	> 20:1	87
3	**C2**, instead of (acid+DMDC)	60	> 20:1	82
4	**C3**, instead of (acid+DMDC)	6	> 20:1	84

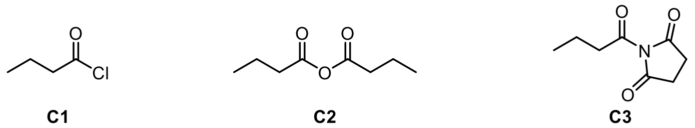

The yield and regioselective ratios were determined by GC analysis with tetradecane as internal standard. The ee value was determined by HPLC analysis.

1.4 Catalytic Enantioselective α-Acylation of Saturated Azacycles

General Procedure B (GP-B): Catalytic enantioselective α-acylation of saturated azacycles with carboxylic acids. In a glovebox, Ir[dF(CF_3)ppy]$_2$(dtbbpy)PF_6 (2.2 mg, 0.002 mmol, 1 mol%), $NiCl_2$·glyme (4.4 mg, 0.020 mmol, 10 mol%), (*S*, *R*)-**L1** (18.2 mg, 0.026 mmol, 13 mol%), *N*-heterocycle (0.60 mmol, 3.0 equiv), a Teflon stir bar, and anhydrous benzene (2.0 mL) was sequentially added to a 15-mL vial. The reaction mixture was stirred at room temperature for 30 min, after which it turned to a pale-yellow suspension (if the carboxylic acid was a solid, it was added as a solid directly at this point). Next, the vial was closed with a PTFE septum cap and wrapped with electrical tape. Then, carboxylic acids (0.20 mmol, 1.0 equiv), 2,6-lutidine (35.0 μL, 0.30 mmol, 1.5 equiv) and DMDC (32.2 μL, 0.30 mmol, 1.5 equiv) were added sequentially via microsyringe. Next, the vial was transferred out of the glovebox, and then vacuum grease was liberally applied to cover the entire top of the septum cap. Then, the reaction mixture was stirred at 20 °C in an EtOH bath for 1 min before being irradiated with a 40 W the blue LED lamp (Kessil PR160L, 427 nm). The reaction was stirred under blue LED irradiation at 20 °C for 24 hours. The reaction mixture was then passed through a short pad of silica gel, with acetone as the eluent (~20 mL). The resulting mixture was concentrated, and the residue was purified by flash chromatography on silica gel or preparative thin-layer chromatography on silica gel.

For compounds **5**, **12**, **18**, **22**, **36–41**, **60**, **67** and **68**, in place of the standard conditions, (*S*, *R*)-**L2** (13 mol%) were used. For compounds **23–26**, in place of the standard conditions, C–H nucleophile (4.0 equiv), (*S*, *R*)-**L2** (13 mol%, for **23**) or (*R*, *S*)-**L2** (13 mol%, for **24–26**) were used. For compounds **11, 63–66, 69** and **70**, in place of the standard conditions, the reactions were run in the absence of 2,6-lutidine.

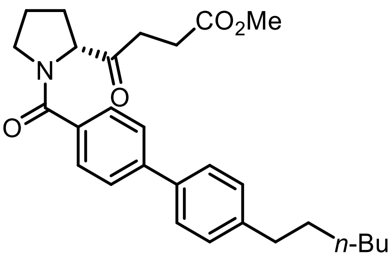

3a

Methyl (*R*)-4-(1-(4'-hexyl-[1,1'-biphenyl]-4-carbonyl)pyrrolidin-2-yl)-4-oxobutanoate (**3a**, from (*S*, *R*)-**L1**). The title compound was synthesized according to **GP-B** from 4-methoxy-4-oxobutanoic acid and (4'-hexyl-[1,1'-biphenyl]-4-yl)(pyrrolidin-1-yl)methanone. The product was purified by preparative TLC on silica gel (1:6 Acetone/Petroleum ether). Yellow oil.

(*S*, *R*)-**L1**: 57.8 mg, 64% yield, 95% ee, 28:1 rr;

(*R*, *S*)-**L1**: 55.8 mg, 62% yield, 94% ee, 28:1 rr.

HPLC analysis: The ee was determined via HPLC on a CHIRALCEL OD-3 column (15.0% 2-PrOH in hexanes, 1.0 mL/min); retention times for compound obtained using (*S*, *R*)-**L1**: 12.2 min (major), 14.1 min (minor).

^{1}H NMR (500 MHz, $CDCl_3$) (81:19 mixture of rotamers) δ 7.68 – 7.55 (m, 4.00H), 7.53 – 7.47 (m, 2.00H), 7.28 – 7.24 (m, 2.00H), 4.78 (dd, *J* = 8.4, 5.8 Hz, 0.81H), 3.84 – 3.56 (m, 5.38H), 3.03 – 2.87 (m, 1.81H), 2.79 – 2.70 (m, 1.00H), 2.68 – 2.57 (m, 3.00H), 2.32 – 2.22 (m, 1.00H), 2.07 – 1.97 (m, 2.00H), 1.92 – 1.83 (m, 1.00H), 1.67 – 1.60 (m, 2.00H), 1.39 – 1.28 (m, 6.00H), 0.92 – 0.85 (m, 3.00H). ^{13}C NMR (126 MHz, $CDCl_3$) major isomer δ 206.99, 173.25, 169.43, 143.03, 142.73, 137.37, 134.29, 128.86, 127.79, 126.88, 126.61, 64.90, 51.64, 50.29, 35.52, 34.39, 31.63, 31.31, 28.91, 28.15, 27.30, 25.42, 22.51, 13.99.

FT-IR (film): 3447, 2927, 1729, 1625, 1412, 1209, 767 cm^{-1}.

HRMS (ESI-MS) *m/z* $[M+H]^+$ calcd for $C_{28}H_{36}NO_4$: 450.2639, found: 450.2637.

$[\alpha]^{25}_D$ = +37.2 (*c* 1.0, CH_2Cl_2); 95% ee from (*S*, *R*)-**L1**.

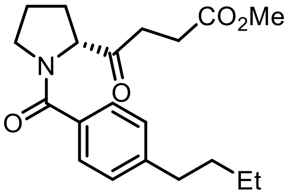

4

Methyl (*R*)-4-(1-(4-butylbenzoyl)pyrrolidin-2-yl)-4-oxobutanoate (**4**, from (*S*, *R*)-**L1**). The title compound was synthesized according to **GP-B** from 4-methoxy-4-oxobutanoic acid and (4-butylphenyl)(pyrrolidin-1-yl)methanone. The product was purified by preparative TLC on silica gel (1:4 Acetone/Petroleum ether). Yellow oil.

(*S*, *R*)-**L1**: 47.3 mg, 68% yield, 96% ee, >20:1 rr;

(*R*, *S*)-**L1**: 47.2 mg, 68% yield, 95% ee, >20:1 rr.

HPLC analysis: The ee was determined via HPLC on a CHIRALCEL OD-3 column (15.0% 2-PrOH in hexanes, 1.0 mL/min); retention times for compound obtained using (*S*, *R*)-**L1**: 9.9 min (major), 13.4 min (minor).

^{1}H NMR (400 MHz, CD_2Cl_2) (87:13 mixture of rotamers) δ 7.51 – 7.41 (m, 1.74H), 7.24 (d, *J* = 8.0 Hz, 1.74H), 7.18 (s, 0.52H), 4.77 – 4.59 (m, 0.87H), 4.50 (d, *J* = 8.8 Hz, 0.13H), 3.73 – 3.53 (m,

5.13H), 3.01 – 2.76 (m, 1.87H), 2.71 – 2.54 (m, 4.00H), 2.30 – 2.20 (m, 1.00H), 1.99 – 1.92 (m, 2.00H), 1.91 – 1.82 (m, 1.00H), 1.66 – 1.56 (m, 2.00H), 1.42 – 1.31 (m, 2.00H), 0.93 (t, *J* = 7.3 Hz, 3.00H). ^{13}C NMR (126 MHz, CD_2Cl_2) major isomer δ 207.66, 173.51, 169.92, 145.95, 133.91, 128.59, 127.68, 65.58, 51.95, 50.79, 35.85, 34.40, 33.83, 28.71, 27.76, 25.90, 22.71, 14.07.

FT-IR (film): 2955, 2930, 1735, 1625, 1420, 1207, 760 cm^{-1}.

HRMS (ESI-MS) *m/z* $[M+H]^+$ calcd for $C_{20}H_{28}NO_4$: 346.2013, found: 346.2010.

$[\alpha]^{25}_D$ = +55.0 (*c* 1.0, CH_2Cl_2); 96% ee from (*S*, *R*)-**L1**.

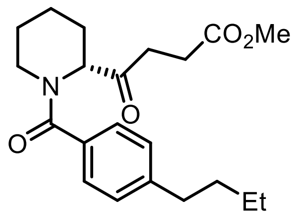

5

Methyl (*R*)-4-(1-(4-butylbenzoyl)piperidin-2-yl)-4-oxobutanoate (**5**, from (*S*, *R*)-**L2**). The title compound was synthesized according to **GP-B** from 4-methoxy-4-oxobutanoic acid and (4-butylphenyl)(piperidin-1-yl)methanone. The product was purified by preparative TLC on silica gel (1:4 Acetone/Petroleum ether). Colorless oil.

(*S*, *R*)-**L2**: 43.2 mg, 60% yield, 94% ee, >20:1 rr;

(*R*, *S*)-**L2**: 43.3 mg, 60% yield, 93% ee, >20:1 rr.

HPLC analysis: The ee was determined via HPLC on a CHIRALCEL OD-3 column (10.0% 2-PrOH in hexanes, 1.0 mL/min); retention times for compound obtained using (*S*, *R*)-**L2**: 9.6 min (major), 11.3 min (minor).

^{1}H NMR (400 MHz, CD_2Cl_2) (66:34 mixture of rotamers) δ 7.34 (d, *J* = 7.8 Hz, 1.66H), 7.29 – 7.19 (m, 2.34H), 5.31 – 5.23 (m, 0.66H), 4.64 – 4.33 (m, 0.34H), 3.76 – 3.59 (m, 3.66H), 3.18 – 3.09 (m, 0.68H), 2.99 – 2.75 (m, 2.00H), 2.74 – 2.45 (m, 4.66H), 2.41 – 2.20 (m, 1.00H), 1.90 (brs, 0.66H), 1.78 – 1.68 (m, 2.00H), 1.66 – 1.57 (m, 2.34H), 1.51 – 1.33 (m, 4.00H), 0.94 (t, *J* = 7.4 Hz, 3.00H). ^{13}C NMR (126 MHz, CD_2Cl_2) major isomer δ 207.56, 173.43, 171.78, 145.30, 133.62, 128.79, 127.30, 59.17, 52.00, 46.70, 35.84, 34.30, 33.88, 27.94, 26.03, 25.53, 22.72, 21.30, 14.08

FT-IR (film): 2931, 2858, 1740, 1633, 1421, 1276, 999, 760 cm^{-1}.

HRMS (ESI-MS) *m/z* $[M+H]^+$ calcd for $C_{21}H_{30}NO_4$: 360.2169, found: 360.2166.

$[\alpha]^{25}_D$ = +74.7 (*c* 1.0, CH_2Cl_2); 94% ee from (*S*, *R*)-**L2**.

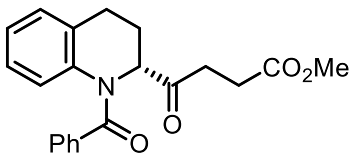

6

Methyl (*R*)-4-(1-benzoyl-1,2,3,4-tetrahydroquinolin-2-yl)-4-oxobutanoate (**6**, from (*S*, *R*)-**L1**). The title compound was synthesized according to **GP-B** from 4-methoxy-4-oxobutanoic acid and (3,4-dihydroquinolin-1(2*H*)-yl)(phenyl)methanone. The product was purified by preparative TLC on silica gel (1:3 EtOAc/Petroleum ether). Yellow oil.

(*S*, *R*)-**L1**: 59.6 mg, 88% yield, 92% ee, >20:1 rr;

(*R*, *S*)-**L1**: 55.5 mg, 82% yield, 90% ee, >20:1 rr.

HPLC analysis: The ee was determined via HPLC on a CHIRALCEL OD-3 column (15.0% 2-PrOH in hexanes, 1.0 mL/min); retention times for compound obtained using (*S*, *R*)-**L1**: 16.7 min (major), 22.6 min (minor).

^{1}H NMR (400 MHz, $CDCl_3$) δ 7.37 – 7.28 (m, 3H), 7.22 (t, *J* = 7.5 Hz, 2H), 7.14 (d, *J* = 7.5 Hz, 1H), 6.98 (t, *J* = 7.5 Hz, 1H), 6.84 (td, *J* = 7.6, 1.5 Hz, 1H), 6.56 (d, *J* = 8.0 Hz, 1H), 5.09 (t, *J* = 8.5 Hz, 1H), 3.62 (s, 3H), 3.03 – 2.91 (m, 1H), 2.89 – 2.78 (m, 2H), 2.78 – 2.63 (m, 2H), 2.61 – 2.50 (m, 2H), 1.93 – 1.82 (m, 1H). ^{13}C NMR (101 MHz, $CDCl_3$) δ 206.69, 173.08, 170.41, 138.41, 134.96, 132.72, 130.55, 128.92, 127.97, 127.45, 126.39, 125.83, 124.85, 62.64, 51.66, 33.85, 27.52, 27.26, 26.17.

FT-IR (film): 3448, 2952, 1728, 1639, 1490, 1350, 1208, 724 cm^{-1}.

HRMS (ESI-MS) *m/z* $[M+H]^+$ calcd for $C_{21}H_{22}NO_4$: 352.1543, found: 352.1539.

$[\alpha]^{25}_D$ = +176.3 (*c* 1.0, CH_2Cl_2); 92% ee from (*S*, *R*)-**L1**.

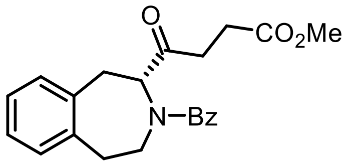

7

Methyl (*R*)-4-(3-benzoyl-2,3,4,5-tetrahydro-1*H*-benzo[*d*]azepin-2-yl)-4-oxobutanoate (**7**, from (*S*, *R*)-**L1**). The title compound was synthesized according to **GP-B** from methyl 4-methoxy-4-oxobutanoic acid and phenyl(1,2,4,5-tetrahydro-3*H*-benzo[*d*]azepin-3-yl)methanone. The product was purified by preparative TLC on silica gel (1:4 Acetone/Petroleum ether). Yellow oil.

(*S*, *R*)-**L1**: 33.7 mg, 46% yield, 81% ee, >20:1 rr;

(*R*, *S*)-**L1**: 32.7 mg, 45% yield, 78% ee, >20:1 rr.

HPLC analysis: The ee was determined via HPLC on a CHIRALCEL OD-3 column (25.0% 2-PrOH in hexanes, 0.8 mL/min); retention times for compound obtained using (*S*, *R*)-**L1**: 11.4 min (major), 14.6 min (minor).

^{1}H NMR (500 MHz, $CDCl_3$) (81:19 mixture of rotamers) δ 7.49 – 7.27 (m, 4.62H), 7.26 – 7.16 (m, 3.00H), 7.12 – 7.06 (m, 1.00H), 6.96 (d, *J* = 7.4 Hz, 0.38H), 5.37 (dd, *J* = 9.4, 4.7 Hz, 0.81H), 4.86 – 4.75 (m, 0.19H), 4.53 (dd, *J* = 8.9, 5.0 Hz, 0.19H), 3.86 – 3.79 (m, 0.81H), 3.70 – 3.63 (m, 3.00H), 3.62 – 3.54 (m, 0.81H), 3.46 – 3.38 (m, 0.81H), 3.33 – 3.28 (m, 0.19H), 3.28 – 3.20 (m, 0.81H), 3.20 – 3.09 (m, 0.38H), 3.09 – 3.02 (m, 0.38H), 3.00 – 2.85 (m, 3.00H), 2.84 – 2.44 (m,

2.62H). ^{13}C NMR (126 MHz, $CDCl_3$) major isomer δ 205.96, 173.07, 172.49, 138.10, 136.37, 135.89, 130.21, 129.67, 129.52, 128.46, 127.26, 127.02, 126.61, 61.54, 51.77, 45.12, 35.08, 34.67, 34.55, 27.56.

FT-IR (film): 3449, 2921, 2850, 1721, 1632, 1421, 1208, 751 cm^{-1}.

HRMS (ESI-MS) *m/z* $[M+H]^+$ calcd for $C_{22}H_{24}NO_4$: 366.1700, found: 366.1697.

$[\alpha]^{25}_D$ = +41.5 (*c* 1.0, CH_2Cl_2); 81% ee from (*S*, *R*)-**L1**.

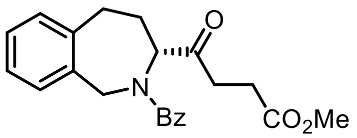

8

Methyl (*R*)-4-(2-benzoyl-2,3,4,5-tetrahydro-1*H*-benzo[*c*]azepin-3-yl)-4-oxobutanoate (**8**, from (*S*, *R*)-**L1**). The title compound was synthesized according to **GP-B** from 4-methoxy-4-oxobutanoic acid and phenyl(1,3,4,5-tetrahydro-2*H*-benzo[*c*]azepin-2-yl)methanone. The product was purified by preparative TLC on silica gel (1:5 Acetone/Petroleum ether). Yellow oil.

(*S*, *R*)-**L1**: 32.2 mg, 44% yield, 84% ee, 16:1 rr;

(*R*, *S*)-**L1**: 32.3 mg, 44% yield, 83% ee, 16:1 rr.

HPLC analysis: The ee was determined via HPLC on a CHIRALPAK AD-3 column (20.0% 2-PrOH in hexanes, 1.0 mL/min); retention times for compound obtained using (*S*, *R*)-**L1**: 9.3 min (major), 18.9 min (minor).

^{1}H NMR (500 MHz, $CDCl_3$) (78:22 mixture of rotamers) δ 7.48 – 7.36 (m, 2.00H), 7.35 – 7.27 (m, 2.00H), 7.25 – 7.22 (m, 1.44H), 7.20 – 7.11 (m, 2.00H), 7.08 – 7.02 (m, 0.78H), 6.56 (d, *J* = 7.5 Hz, 0.78H), 5.40 – 5.33 (m, 0.22H), 5.30 (t, *J* = 7.5 Hz, 0.78H), 4.68 – 4.59 (m, 0.78H), 4.50 (t, *J* = 5.9 Hz, 0.22H), 4.47 – 4.39 (m, 0.78H), 4.25 – 4.14 (m, 0.22H), 3.72 – 3.60 (m, 3.00H), 3.16 – 3.07 (m, 1.00H), 3.05 – 2.91 (m, 2.44H), 2.86 – 2.79 (m, 0.22H), 2.79 – 2.53 (m, 2.56H), 2.46 – 2.34 (m, 1.78H). ^{13}C NMR (126 MHz, $CDCl_3$) major isomer δ 207.09, 173.15, 173.10, 139.58, 136.43, 135.49, 130.13, 130.02, 128.16, 128.11, 127.47, 127.15, 126.10, 63.42, 51.79, 51.22, 34.46, 31.94, 27.49, 26.73.

FT-IR (film): 2922, 1736, 1636, 1413, 1247, 702 cm^{-1}.

HRMS (ESI-MS) *m/z* $[M+H]^+$ calcd for $C_{22}H_{24}NO_4$: 366.1700, found: 366.1697.

$[\alpha]^{25}_D$ = +27.0 (*c* 1.0, CH_2Cl_2); 84% ee from (*S*, *R*)-**L1**.

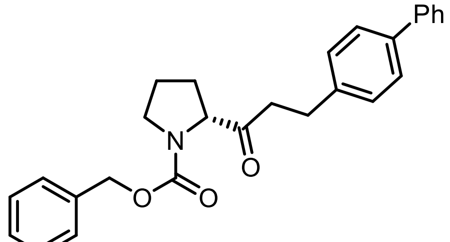

9

Benzyl (*R*)-2-(3-([1,1'-biphenyl]-4-yl)propanoyl)pyrrolidine-1-carboxylate (**9**, from (*S*, *R*)-**L1**). The title compound was synthesized according to **GP-B** from 3-([1,1'-biphenyl]-4-yl)propanoic acid and benzyl pyrrolidine-1-carboxylate. The product was purified by preparative TLC on silica gel (1:6 Acetone/Petroleum ether). Yellowish-green oil.

(*S*, *R*)-**L1**: 51.0 mg, 62% yield, 88% ee, >20:1 rr;

(*R*, *S*)-**L1**: 52.8 mg, 64% yield, 89% ee, >20:1 rr.

HPLC analysis: The ee was determined via HPLC on a CHIRALCEL OD-3 column (15.0% 2-PrOH in hexanes, 1.0 mL/min); retention times for compound obtained using (*S*, *R*)-**L1**: 17.4 min (minor), 20.3 min (major).

^{1}H NMR (500 MHz, $CDCl_3$) (51:49 mixture of rotamers) δ 7.60 – 7.56 (m, 2.00H), 7.54 – 7.48 (m, 2.00H), 7.46 – 7.42 (m, 2.00H), 7.40 – 7.26 (m, 7.00H), 7.14 (d, *J* = 7.9 Hz, 1.00H), 5.22 – 5.04 (m, 2.00H), 4.46 (dd, *J* = 8.8, 4.4 Hz, 0.51H), 4.36 (dd, *J* = 8.8, 4.8 Hz, 0.49H), 3.63 – 3.49 (m, 2.00H), 3.02 – 2.96 (m, 1.00H), 2.90 – 2.81 (m, 2.00H), 2.77 – 2.60 (m, 1.00H), 2.20 – 2.05 (m, 1.00H), 1.89 – 1.81 (m, 2.00H), 1.81 – 1.73 (m, 1.00H). ^{13}C NMR (126 MHz, $CDCl_3$) δ 208.63, 208.45, 155.02, 154.26, 140.89, 140.82, 140.21, 139.95, 139.05, 138.98, 136.64, 136.34, 128.73, 128.66, 128.64, 128.45, 128.42, 128.06, 127.92, 127.77, 127.10, 127.08, 127.02, 126.89, 67.12, 67.00, 65.13, 64.93, 47.24, 46.68, 41.06, 40.37, 29.65, 28.97, 28.81, 28.49, 24.27, 23.52.

FT-IR (film): 3448, 1702, 1415, 1356, 1117, 698 cm^{-1}.

HRMS (ESI-MS) *m/z* $[M+Na]^+$ calcd for $C_{27}H_{27}NNaO_3$: 436.1883, found: 436.1881.

$[\alpha]^{25}_{D}$ = +33.7 (*c* 1.0, CH_2Cl_2); 88% ee from (*S*, *R*)-**L1**.

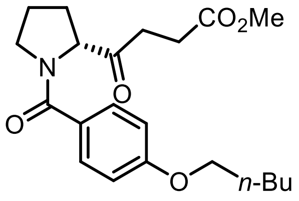

10

Methyl (*R*)-4-oxo-4-(1-(4-(pentyloxy)benzoyl)pyrrolidin-2-yl)butanoate (**10**, from (*S*, *R*)-**L1**). The title compound was synthesized according to **GP-B** from 4-methoxy-4-oxobutanoic acid and (4-(pentyloxy)phenyl)(pyrrolidin-1-yl)methanone. The product was purified by preparative TLC on silica gel (1:4 Acetone/Petroleum ether). Yellow oil.

(*S*, *R*)-**L1**: 48.2 mg, 64% yield, 94% ee, 17:1 rr;

(*R*, *S*)-**L1**: 49.2 mg, 66% yield, 94% ee, 17:1 rr.

HPLC analysis: The ee was determined via HPLC on a CHIRALCEL OD-3 column (20.0% 2-PrOH in hexanes, 1.0 mL/min); retention times for compound obtained using (*S*, *R*)-**L1**: 9.6 min (major), 13.6 min (minor).

^{1}H NMR (400 MHz, $CDCl_3$) (87:13 mixture of rotamers) δ 7.54 (d, *J* = 8.6 Hz, 1.74H), 6.88 (d, *J* = 8.6 Hz, 2.26H), 4.74 (dd, *J* = 7.8, 6.5 Hz, 0.87H), 4.53 (brs, 0.13H), 3.97 (t, *J* = 6.6 Hz, 2.00H), 3.82 – 3.55 (m, 5.00H), 3.01 – 2.52 (m, 3.87H), 2.39 (brs, 0.13H), 2.32 – 2.19 (m, 1.00H), 2.08 – 1.94 (m, 2.00H), 1.92 – 1.83 (m, 1.00H), 1.83 – 1.74 (m, 2.00H), 1.49 – 1.33 (m, 4.00H), 0.97 – 0.85 (m, 3.00H). ^{13}C NMR (126 MHz, $CDCl_3$) major isomer δ 207.19, 173.24, 169.33, 160.75, 129.28, 127.72, 113.89, 68.00, 65.00, 51.60, 50.40, 34.28, 28.73, 28.11, 28.03, 27.27, 25.51, 22.30, 13.87.

FT-IR (film): 2954, 1736, 1608, 1421, 1253, 1174, 843, 765 cm^{-1}.

HRMS (ESI-MS) *m/z* $[M+H]^+$ calcd for $C_{21}H_{30}NO_5$: 376.2118, found: 376.2116.

$[\alpha]^{25}_{D}$ = +47.2 (*c* 1.0, CH_2Cl_2); 94% ee from (*S*, *R*)-**L1**.

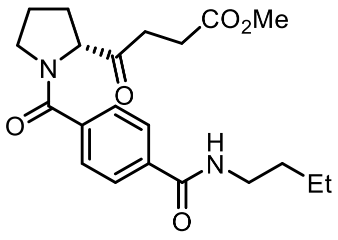

11

Methyl (*R*)-4-(1-(4-(butylcarbamoyl)benzoyl)pyrrolidin-2-yl)-4-oxobutanoate (**11**, from (*S*, *R*)-**L1**). The title compound was synthesized according to **GP-B** from 4-methoxy-4-oxobutanoic acid and *N*-butyl-4-(pyrrolidine-1-carbonyl)benzamide. The product was purified by preparative TLC on silica gel (1:1:3 Acetone/DCM/Petroleum ether). White solid.

(*S*, *R*)-**L1**: 41.0 mg, 53% yield, 88% ee, 15:1 rr;

(*R*, *S*)-**L1**: 40.3 mg, 52% yield, 89% ee, 15:1 rr.

HPLC analysis: The ee was determined via HPLC on a CHIRALPAK AD-3 column (20.0% 2-PrOH in hexanes, 1.0 mL/min); retention times for compound obtained using (*S*, *R*)-**L1**: 14.7 min (major), 20.5 min (minor).

^{1}H NMR (400 MHz, CD_2Cl_2) (87:13 mixture of rotamers) δ 7.85 – 7.66 (m, 2.00H), 7.62 – 7.44 (m, 1.74H), 7.27 (d, *J* = 8.0 Hz, 0.26H), 6.65 (t, *J* = 5.9 Hz, 1.00H), 4.69 (dd, *J* = 8.5, 5.8 Hz, 0.87H), 4.43 (dd, *J* = 9.1, 3.0 Hz, 0.13H), 3.76 – 3.44 (m, 5.13H), 3.39 (td, *J* = 7.2, 5.7 Hz, 2.00H), 2.88 (t, *J* = 6.5 Hz, 1.74H), 2.71 – 2.52 (m, 2.00H), 2.50 – 2.42 (m, 0.13H), 2.32 – 2.20 (m, 1.00H), 2.01 – 1.92 (m, 2.00H), 1.91 – 1.83 (m, 1.00H), 1.64 – 1.51 (m, 2.00H), 1.46 – 1.34 (m, 2.00H), 0.94 (t, *J* = 7.3 Hz, 3.00H). ^{13}C NMR (126 MHz, CD_2Cl_2) δ 207.24, 207.17, 173.43, 169.73, 169.05, 166.87, 166.83, 140.22, 139.14, 137.02, 127.62, 127.45, 127.32, 127.01, 67.38, 65.47, 52.04, 51.97, 50.62, 47.15, 40.19, 34.62, 34.31, 32.08, 30.41, 28.69, 27.75, 27.50, 25.78, 22.80, 20.56, 13.96.

FT-IR (film): 2955, 2930, 1735, 1625, 1420, 1403, 1207, 760 cm^{-1}.

HRMS (ESI-MS) *m/z* $[M+H]^+$ calcd for $C_{21}H_{29}N_2O_5$: 389.2071, found: 389.2066.

$[\alpha]^{25}_D$ = +52.8 (*c* 1.0, CH_2Cl_2); 88% ee from (*S*, *R*)-**L1**.

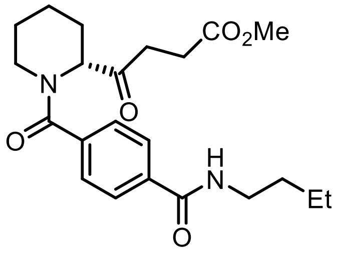

12

Methyl (*R*)-4-(1-(4-(butylcarbamoyl)benzoyl)piperidin-2-yl)-4-oxobutanoate (**12**, from (*S*, *R*)-**L2**). The title compound was synthesized according to **GP-B** from 4-methoxy-4-oxobutanoic acid and *N*-butyl-4-(piperidine-1-carbonyl)benzamide. The product was purified by preparative TLC on silica gel (1:1:4 Acetone/EtOAc/Petroleum ether). Colorless oil.

(*S*, *R*)-**L2**: 40.6 mg, 50% yield, 93% ee, 19:1 rr;

(*R*, *S*)-**L2**: 40.7 mg, 51% yield, 92% ee, 19:1 rr.

HPLC analysis: The ee was determined via HPLC on a CHIRALPAK IF column (25.0% 2-PrOH in hexanes, 0.8 mL/min); retention times for compound obtained using (*S*, *R*)-**L2**: 20.2 min (major), 26.5 min (minor).

^{1}H NMR (400 MHz, CD_2Cl_2) (86:14 mixture of rotamers) δ 7.83 – 7.68 (m, 2.00H), 7.50 – 7.33 (m, 1.72H), 7.31 – 7.26 (m, 0.28H), 7.16 (d, *J* = 7.2 Hz, 0.14H), 6.73 – 6.56 (m, 0.86H), 5.32 – 5.27 (m, 0.75H), 4.79 – 4.71 (m, 0.11H), 4.56 (d, *J* = 13.2 Hz, 0.14H), 4.24 (brs, 0.14H), 3.72 – 3.49 (m, 4.00H), 3.38 (q, *J* = 6.6 Hz, 1.86H), 3.30 – 3.06 (m, 1.00H), 2.90 – 2.77 (m, 1.72H), 2.66 – 2.52 (m, 2.28H), 2.39 – 2.16 (m, 1.00H), 1.99 – 1.90 (m, 1.00H), 1.78 – 1.65 (m, 2.00H), 1.60 – 1.49 (m, 2.72H), 1.39 (p, *J* = 7.4 Hz, 3.28H), 0.93 (t, *J* = 7.3 Hz, 3.00H). ^{13}C NMR (126 MHz, CD_2Cl_2) δ 208.03, 207.16, 173.39, 173.28, 170.93, 169.40, 166.87, 166.82, 140.15, 138.96, 136.55, 135.29, 127.71, 127.64, 127.51, 127.27, 127.24, 126.75, 59.14, 59.00, 52.04, 46.55, 40.44, 40.19, 34.75, 34.30, 33.62, 32.08, 27.96, 27.92, 26.82, 25.87, 25.61, 25.17, 21.35, 21.15, 20.55, 19.07, 14.02, 13.96.

FT-IR (film): 3332, 2954, 1738, 1720, 1635, 1544, 1436, 1278, 859 cm^{-1}.

HRMS (ESI-MS) *m/z* $[M+H]^+$ calcd for $C_{22}H_{31}N_2O_5$: 403.2227 found: 403.2224.

$[\alpha]^{25}_D$ = +51.6 (*c* 1.0, CH_2Cl_2); 93% ee from (*S*, *R*)-**L2**.

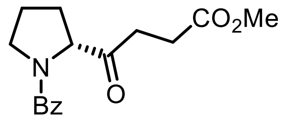

13

Methyl (*R*)-4-(1-benzoylpyrrolidin-2-yl)-4-oxobutanoate (**13**, from (*S*, *R*)-**L1**). The title compound was synthesized according to **GP-B** from 4-methoxy-4-oxobutanoic acid and *N*-Bz-pyrrolidine. The product was purified by preparative TLC on silica gel (1:4 Acetone/Petroleum ether). Yellow oil.

(*S*, *R*)-**L1**: 41.6 mg, 72% yield, 96% ee; (*R*, *S*)-**L1**: 42.4 mg, 73% yield, 97% ee.

HPLC analysis: The ee was determined via HPLC on a CHIRALCEL OD-3 column (20.0% 2-PrOH in hexanes, 1.0 mL/min); retention times for compound obtained using (*S*, *R*)-**L1**: 11.2 min (major), 14.7 min (minor).

^{1}H NMR (400 MHz, $CDCl_3$) (87:13 mixture of rotamers) δ 7.56 – 7.50 (m, 1.72H), 7.44 – 7.27 (m, 3.28H), 4.74 (dd, *J* = 8.1, 5.8 Hz, 0.87H), 4.44 (dd, *J* = 8.5, 2.1 Hz, 0.13H), 3.76 (t, *J* = 7.0 Hz, 0.26H), 3.67 – 3.57 (m, 4.00H), 3.56 – 3.49 (m, 0.87H), 3.00 – 2.83 (m, 1.87H), 2.77 – 2.65 (m, 1.00H), 2.63 – 2.52 (m, 1.00H), 2.30 – 2.20 (m, 1.00H), 2.04 – 1.94 (m, 2.00H), 1.89 – 1.80 (m, 1.00H). ^{13}C NMR (101 MHz, $CDCl_3$) δ 206.98, 173.25, 169.63, 135.94, 130.17, 129.54, 128.36, 128.19, 127.14, 126.45, 66.89, 64.79, 51.66, 50.22, 46.68, 34.37, 29.93, 28.15, 27.27, 27.02, 25.33, 22.40.

FT-IR (film): 3448, 1727, 1625, 1417, 1212, 702 cm^{-1}.

HRMS (ESI-MS) *m/z* $[M+H]^+$ calcd for $C_{16}H_{20}NO_4$: 290.1387, found: 290.1383.

$[\alpha]^{25}_D$ = +70.3 (*c* 1.0, CH_2Cl_2); 96% ee from (*S*, *R*)-**L1**.

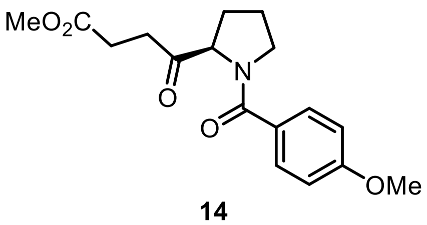

14

Methyl (*R*)-4-(1-(4-methoxybenzoyl)pyrrolidin-2-yl)-4-oxobutanoate (**14**, from (*S*, *R*)-**L1**). The title compound was synthesized according to **GP-B** from 4-methoxy-4-oxobutanoic acid and (4-methoxyphenyl)(pyrrolidin-1-yl)methanone. The product was purified by preparative TLC on silica gel (1:3 Acetone/Petroleum ether). Yellow oil.

(*S*, *R*)-**L1**: 42.6 mg, 67% yield, 97% ee; (*R*, *S*)-**L1**: 44.7 mg, 70% yield, 97% ee.

HPLC analysis: The ee was determined via HPLC on a CHIRALCEL OD-3 column (25.0% 2-PrOH in hexanes, 0.8 mL/min); retention times for compound obtained using (*S*, *R*)-**L1**: 11.5 min (major), 16.2 min (minor).

^{1}H NMR (400 MHz, $CDCl_3$) (90:10 mixture of rotamers) δ 7.62 – 7.49 (m, 1.90H), 6.94 – 6.84 (m, 2.10H), 4.74 (dd, *J* = 8.0, 6.2 Hz, 0.90H), 4.52 (brs, 0.10H), 3.82 (s, 3.00H), 3.71 – 3.58 (m, 5.00H), 3.01 – 2.82 (m, 2.00H), 2.76 – 2.66 (m, 1.00H), 2.65 – 2.54 (m, 1.00H), 2.30 – 2.19 (m, 1.00H), 2.04 – 1.94 (m, 2.00H), 1.90 – 1.84 (m, 1.00H). ^{13}C NMR (101 MHz, $CDCl_3$) major isomer δ 207.23, 173.33, 169.34, 161.19, 129.36, 128.03, 113.44, 65.04, 55.29, 51.69, 50.46, 34.38, 28.17, 27.32, 25.56.

FT-IR (film): 3448, 1727, 1609, 1423, 1254, 1174, 765 cm^{-1}.

HRMS (ESI-MS) *m/z* $[M+H]^+$ calcd for $C_{17}H_{22}NO_5$: 320.1492, found: 320.1488.

$[\alpha]^{25}_D$ = +65.6 (*c* 1.0, CH_2Cl_2); 97% ee from (*S*, *R*)-**L1**.

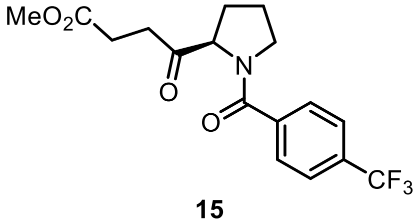

15

Methyl (*R*)-4-oxo-4-(1-(4-(trifluoromethyl)benzoyl)pyrrolidin-2-yl)butanoate (**15**, from (*S*, *R*)-**L1**). The title compound was synthesized according to **GP-B** from 4-methoxy-4-oxobutanoic acid and pyrrolidin-1-yl(4-(trifluoromethyl)phenyl)methanone. The product was purified by preparative TLC on silica gel (1:4 Acetone/Petroleum ether). Yellow oil.

(*S*, *R*)-**L1**: 48.2 mg, 67% yield, 96% ee; (*R*, *S*)-**L1**: 48.6 mg, 68% yield, 96% ee.

HPLC analysis: The ee was determined via HPLC on a CHIRALPAK AD-3 column (20.0% 2-PrOH in hexanes, 1.0 mL/min); retention times for compound obtained using (*S*, *R*)-**L1**: 9.8 min (major), 22.4 min (minor).

^{1}H NMR (400 MHz, $CDCl_3$) (88:12 mixture of rotamers) δ 7.71 – 7.58 (m, 3.76H), 7.42 (d, *J* = 8.0 Hz, 0.24H), 4.83 – 4.71 (m, 0.88H), 4.42 (dd, *J* = 8.8, 2.7 Hz, 0.12H), 3.78 – 3.75 (m, 0.12H), 3.69 – 3.63 (m, 2.76H), 3.62 – 3.53 (m, 1.24H), 3.51 – 3.44 (m, 0.88H), 2.98 – 2.84 (m, 1.76H), 2.79 – 2.71 (m, 0.88H), 2.68 – 2.49 (m, 1.24H), 2.39 – 2.22 (m, 1.12H), 2.07 – 1.95 (m, 2.00H), 1.92 –

1.84 (m, 1.00H). ^{13}C NMR (101 MHz, $CDCl_3$) δ 206.48, 206.33, 173.21, 172.53, 168.91, 168.20, 140.57, 139.48, 132.00 (q, J_{C-F} = 32.8 Hz), 127.52, 127.03, 125.35 (q, J_{C-F} = 3.8 Hz), 123.64 (q, J_{C-F} = 272.5 Hz), 67.06, 64.75, 51.78, 51.73, 50.10, 46.71, 34.58, 33.66, 29.92, 28.09, 27.25, 27.06, 25.23, 22.27.^{19}F NMR (376 MHz, $CDCl_3$) δ −62.90 (s, 0.36F), −62.93 (s, 2.64F).

FT-IR (film): 3458, 1731, 1633, 1436, 1325, 1127, 856 cm^{-1}.

HRMS (ESI-MS) *m/z* $[M+H]^+$ calcd for $C_{17}H_{19}F_3NO_4$: 358.1261, found: 358.1256.

$[\alpha]^{25}_D$ = +58.2 (*c* 1.0, CH_2Cl_2); 96% ee from (*S*, *R*)-**L1**.

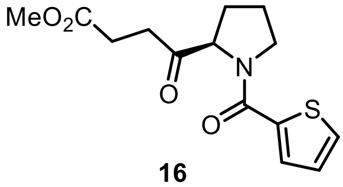

16

Methyl (*R*)-4-oxo-4-(1-(thiophene-2-carbonyl)pyrrolidin-2-yl)butanoate (**16**, from (*S*, *R*)-**L1**). The title compound was synthesized according to **GP-B** from 4-methoxy-4-oxobutanoic acid and pyrrolidin-1-yl(thiophen-2-yl)methanone. The product was purified by preparative TLC on silica gel (1:3 Acetone/Petroleum ether). Yellow oil.

(*S*, *R*)-**L1**: 44.5 mg, 75% yield, 97% ee; (*R*, *S*)-**L1**: 43.9 mg, 74% yield, 97% ee.

HPLC analysis: The ee was determined via HPLC on a CHIRALCEL OD-3 column (20.0% 2-PrOH in hexanes, 1.0 mL/min); retention times for compound obtained using (*S*, *R*)-**L1**: 15.9 min (major), 21.4 min (minor).

^{1}H NMR (400 MHz, $CDCl_3$) δ 7.56 (d, *J* = 3.8 Hz, 1H), 7.48 (d, *J* = 5.0 Hz, 1H), 7.10 – 7.04 (m, 1H), 4.75 (dd, *J* = 8.3, 5.1 Hz, 1H), 3.99 – 3.86 (m, 2H), 3.64 (s, 3H), 2.89 (t, *J* = 6.4 Hz, 2H), 2.76 – 2.65 (m, 1H), 2.60 – 2.51 (m, 1H), 2.27 – 2.07 (m, 2H), 2.06 – 1.96 (m, 2H). ^{13}C NMR (101 MHz, $CDCl_3$) δ 206.87, 173.28, 161.85, 138.28, 130.29, 130.22, 127.19, 65.85, 51.66, 49.52, 34.49, 27.64, 27.25, 25.49.

FT-IR (film): 3448, 1727, 1601, 1436, 1210, 739 cm^{-1}.

HRMS (ESI-MS) *m/z* $[M+H]^+$ calcd for $C_{14}H_{18}NO_4S$: 296.0951, found: 296.0949.

$[\alpha]^{25}_D$ = +61.9 (*c* 1.0, CH_2Cl_2); 97% ee from (*S*, *R*)-**L1**.

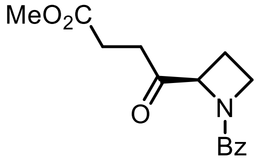

17

Methyl (*R*)-4-(1-benzoylazetidin-2-yl)-4-oxobutanoate (**17**, from (*S*, *R*)-**L1**). The title compound was synthesized according to **GP-B** from 4-methoxy-4-oxobutanoic acid and azetidin-1-yl(phenyl)methanone. The product was purified by preparative TLC on silica gel (1:3 Acetone/Petroleum ether). Yellow oil.

(*S*, *R*)-**L1**: 37.3 mg, 68% yield, 85% ee; (*R*, *S*)-**L1**: 37.2 mg, 68% yield, 84% ee.

HPLC analysis: The ee was determined via HPLC on a CHIRALCEL OD-3 column (20.0% 2-PrOH in hexanes, 1.0 mL/min); retention times for compound obtained using (*S*, *R*)-**L1**: 12.5 min (major), 16.6 min (minor).

^{1}H NMR (400 MHz, $CDCl_3$) δ 7.73 – 7.57 (m, 2H), 7.50 – 7.36 (m, 3H), 5.14 – 4.92 (m, 1H), 4.44 – 4.30 (m, 1H), 4.20 – 4.09 (m, 1H), 3.65 (s, 3H), 3.19 – 2.78 (m, 2H), 2.72 – 2.60 (m, 2H), 2.59 – 2.35 (m, 2H). ^{13}C NMR (101 MHz, $CDCl_3$) δ 206.65, 173.10, 170.64, 132.32, 131.39, 128.40, 127.90, 65.64, 51.73, 51.50, 33.91, 27.22, 19.86.

FT-IR (film): 3445, 2072, 1633, 1404, 1207, 711 cm^{-1}.

HRMS (ESI-MS) *m/z* $[M+H]^+$ calcd for $C_{15}H_{18}NO_4$: 276.1230, found: 276.1228.

$[\alpha]^{25}_{D}$ = +178.6 (*c* 1.0, CH_2Cl_2); 85% ee from (*S*, *R*)-**L1**.

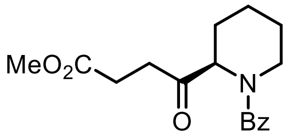

18

Methyl (*R*)-4-(1-benzoylpiperidin-2-yl)-4-oxobutanoate (**18**, from (*S*, *R*)-**L2**). The title compound was synthesized according to **GP-B** from 4-methoxy-4-oxobutanoic acid and phenyl(piperidin-1-yl)methanone. The product was purified by preparative TLC on silica gel (1:4 Acetone/Petroleum ether). Yellow oil.

(*S*, *R*)-**L2**: 38.2 mg, 63% yield, 94% ee; (*R*, *S*)-**L2**: 39.2 mg, 65% yield, 95% ee.

HPLC analysis: The ee was determined via HPLC on a CHIRALCEL OD-3 column (15.0% 2-PrOH in hexanes, 1.0 mL/min); retention times for compound obtained using (*S*, *R*)-**L2**: 9.5 min (major), 12.2 min (minor).

^{1}H NMR (400 MHz, $CDCl_3$) (75:25 mixture of rotamers) δ 7.65 – 7.28 (m, 5.00H), 5.40 – 5.32 (m, 0.75H), 4.66 (d, *J* = 13.5 Hz, 0.13H), 4.34 (s, 0.12H), 3.70 – 3.62 (m, 3.50H), 3.23 – 3.12 (m, 0.75H), 2.95 – 2.75 (m, 1.75H), 2.67 – 2.50 (m, 2.25H), 2.41 – 2.31 (m, 0.75H), 1.94 – 1.62 (m, 3.00H), 1.61 – 1.31 (m, 3.00H). ^{13}C NMR (101 MHz, $CDCl_3$) major isomer δ 207.02, 173.15, 171.50, 135.66, 129.67, 128.45, 126.79, 58.48, 51.77, 46.20, 33.81, 27.41, 25.55, 25.10, 20.65.

FT-IR (film): 3463, 2925, 1716, 1628, 1417, 1261, 703 cm^{-1}.

HRMS (ESI-MS) *m/z* $[M+H]^+$ calcd for $C_{17}H_{22}NO_4$: 304.1543, found: 304.1541.

$[\alpha]^{25}_{D}$ = +78.4 (*c* 1.0, CH_2Cl_2); 94% ee from (*S*, *R*)-**L2**.

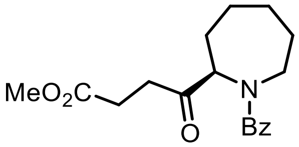

19

Methyl (*R*)-4-(1-benzoylazepan-2-yl)-4-oxobutanoate (**19**, (*S*, *R*)-**L1**). The title compound was synthesized according to **GP-B** from 4-methoxy-4-oxobutanoic acid and azepan-1-yl(phenyl)methanone. The product was purified by preparative TLC on silica gel (1:5 Acetone/Petroleum ether). Yellow oil.

(*S*, *R*)-**L1**: 36.4 mg, 57% yield, 88% ee; (*R*, *S*)-**L1**: 39.1 mg, 62% yield, 89% ee.

HPLC analysis: The ee was determined via HPLC on a CHIRALCEL OD-3 column (15.0% 2-PrOH in hexanes, 1.0 mL/min); retention times for compound obtained using (*S*, *R*)-**L1**: 10.5 min (major), 13.6 min (minor).

^{1}H NMR (400 MHz, $CDCl_3$) (81:19 mixture of rotamers) δ 7.40 – 7.34 (m, 4.62H), 7.23 – 7.19 (m, 0.38H), 4.87 (dd, *J* = 11.8, 5.0 Hz, 0.81H), 4.53 – 4.45 (m, 0.19H), 4.30 – 4.23 (m, 0.19H), 3.71 – 3.60 (m, 4.00H), 3.32 – 3.22 (m, 0.81H), 2.99 – 2.89 (m, 1.62H), 2.80 – 2.68 (m, 1.00H), 2.67 – 2.51 (m, 1.38H), 2.39 – 2.29 (m, 1.00H), 2.02 – 1.91 (m, 1.00H), 1.83 – 1.73 (m, 2.00H), 1.72 – 1.66 (m, 1.00H), 1.54 – 1.27 (m, 3.00H). ^{13}C NMR (101 MHz, $CDCl_3$) δ 207.69, 207.31, 173.35, 172.76, 172.72, 171.66, 136.80, 136.47, 129.34, 129.21, 128.71, 128.38, 126.37, 125.93, 67.18, 64.62, 51.80, 51.70, 47.15, 43.51, 34.42, 33.98, 30.71, 30.64, 29.64, 28.66, 28.41, 27.99, 27.42, 27.21, 26.14, 25.10.

FT-IR (film): 3463, 2927, 1722, 1633, 1417, 1261, 705 cm^{-1}.

HRMS (ESI-MS) *m/z* $[M+H]^+$ calcd for $C_{18}H_{24}NO_4$: 318.1700, found: 318.1697.

$[\alpha]^{25}_D$ = +37.3 (*c* 1.0, CH_2Cl_2); 88% ee from (*S*, *R*)-**L1**.

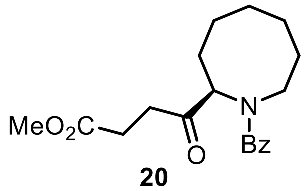

20

Methyl (*R*)-4-(1-benzoylazocan-2-yl)-4-oxobutanoate (**20**, from (*S*, *R*)-**L1**). The title compound was synthesized according to **GP-B** from 4-methoxy-4-oxobutanoic acid and azocan-1-yl(phenyl)methanone. The product was purified by preparative TLC on silica gel (1:5 Acetone/Petroleum ether). Yellow oil.

(*S*, *R*)-**L1**: 34.0 mg, 51% yield, 80% ee; (*R*, *S*)-**L1**: 34.9 mg, 53% yield, 80% ee.

HPLC analysis: The ee was determined via HPLC on a CHIRALCEL OD-3 column (15.0% 2-PrOH in hexanes, 1.0 mL/min); retention times for compound obtained using (*S*, *R*)-**L1**: 10.3 min (major), 12.0 min (minor).

^{1}H NMR (500 MHz, $CDCl_3$) (68:32 mixture of rotamers) δ 7.43 – 7.36 (m, 4.68H), 7.27 (d, *J* = 4.0 Hz, 0.32H), 4.64 (dd, *J* = 11.2, 4.1 Hz, 0.68H), 4.32 – 4.27 (m, 0.32H), 4.22 (dd, *J* = 11.2, 4.1 Hz, 0.32H), 3.70 – 3.63 (m, 3.32H), 3.55 – 3.50 (m, 0.68H), 3.43 – 3.36 (m, 0.68H), 2.91 – 2.82 (m, 1.68H), 2.75 – 2.68 (m, 0.68H), 2.62 – 2.56 (m, 1.00H), 2.56 – 2.48 (m, 0.68H), 2.47 – 2.40 (m, 0.32H), 2.19 – 2.02 (m, 2.00H), 2.01 – 1.91 (m, 0.68H), 1.72 – 1.65 (m, 2.00H), 1.63 – 1.53 (m, 3.00H), 1.49 – 1.39 (m, 2.00H). ^{13}C NMR (126 MHz, $CDCl_3$) δ 207.26, 206.78, 173.41, 172.72, 172.28, 171.66, 136.96, 136.82, 129.22, 129.21, 128.77, 128.41, 126.12, 125.88, 68.13, 64.48, 51.79, 51.73, 47.59, 43.83, 34.15, 33.93, 28.19, 27.75, 27.40, 26.47, 26.23, 26.13, 26.06, 25.59, 25.55, 25.11, 24.78, 24.13.

FT-IR (film): 3458, 2928, 1721, 1632, 1436, 1209, 704 cm^{-1}.

HRMS (ESI-MS) *m/z* $[M+H]^+$ calcd for $C_{19}H_{26}NO_4$: 332.1856, found: 332.1851.

$[\alpha]^{25}_D$ = +43.3 (*c* 1.0, CH_2Cl_2); 80% ee from (*S*, *R*)-**L1**.

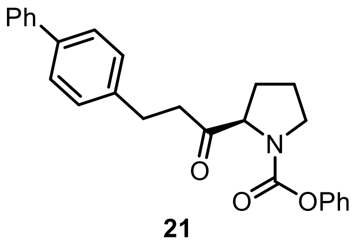

21

Phenyl (*R*)-2-(3-([1,1'-biphenyl]-4-yl)propanoyl)pyrrolidine-1-carboxylate (**21**, from (*S*, *R*)-**L1**). The title compound was synthesized according to **GP-B** from 3-([1,1'-biphenyl]-4-yl)propanoic acid and phenyl pyrrolidine-1-carboxylate. The product was purified by preparative TLC on silica gel (6:1 Acetone/Petroleum ether). Yellow oil.

(*S*, *R*)-**L1**: 49.0 mg, 61% yield, 89% ee; (*R*, *S*)-**L1**: 49.3 mg, 62% yield, 90% ee.

HPLC analysis: The ee was determined via HPLC on a CHIRALPAK AD-3 column (15.0% 2-PrOH in hexanes, 1.0 mL/min); retention times for compound obtained using (*S*, *R*)-**L1**: 12.0 min (major), 14.4 min (minor).

^{1}H NMR (500 MHz, $CDCl_3$) (50:50 mixture of rotamers) δ 7.60 – 7.51 (m, 3.00H), 7.50 – 7.41 (m, 3.00H), 7.40 – 7.26 (m, 4.50H), 7.25 – 7.15 (m, 2.50H), 7.05 – 7.00 (m, 1.00H), 4.59 (dd, *J* = 8.8, 4.4 Hz, 0.50H), 4.52 (dd, *J* = 8.7, 4.4 Hz, 0.50H), 3.78 – 3.61 (m, 2.00H), 3.03 – 2.82 (m, 4.00H), 2.30 – 2.11 (m, 1.00H), 1.98 – 1.77 (m, 3.00H). ^{13}C NMR (126 MHz, $CDCl_3$) δ 207.95, 207.81, 153.40, 152.65, 151.19, 151.01, 140.89, 140.78, 140.15, 139.86, 139.12, 139.03, 129.20, 128.73, 128.68, 127.13, 127.09, 127.04, 126.91, 126.89, 125.31, 125.26, 121.59, 121.54, 65.33, 65.05, 47.35, 47.18, 41.47, 40.59, 29.61, 28.99, 28.97, 28.49, 24.33, 23.40.

FT-IR (film): 3436, 1717, 1488, 1394, 1203, 761 cm^{-1}.

HRMS (ESI-MS) *m/z* $[M+H]^+$ calcd for $C_{26}H_{26}NO_3$: 400.1907, found: 400.1904.

$[\alpha]^{25}_D$ = +21.8 (*c* 1.0, CH_2Cl_2); 89% ee from (*S*, *R*)-**L1**.

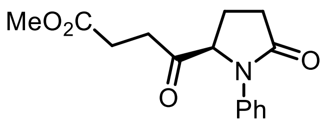

22

Methyl (*R*)-4-oxo-4-(5-oxo-1-phenylpyrrolidin-2-yl)butanoate (**22**, from (*S*, *R*)-**L2**). The title compound was synthesized according to **GP-B** from 4-methoxy-4-oxobutanoic acid and 1-phenylpyrrolidin-2-one. The product was purified by preparative TLC on silica gel (1:4 Acetone/Petroleum ether). Yellow oil.

(*S*, *R*)-**L2**: 45.7 mg, 83% yield, 88% ee; (*R*, *S*)-**L2**: 46.7 mg, 84% yield, 88% ee.

HPLC analysis: The ee was determined via HPLC on a CHIRALPAK AS-3 column (40.0% 2-PrOH in hexanes, 0.8 mL/min); retention times for compound obtained using (*S*, *R*)-**L2**: 17.7 min (major), 26.3 min (minor).

^{1}H NMR (400 MHz, $CDCl_3$) δ 7.46 – 7.40 (m, 2H), 7.37 – 7.31 (m, 2H), 7.18 – 7.12 (m, 1H), 4.85 (dd, *J* = 9.4, 4.1 Hz, 1H), 3.63 (s, 3H), 2.78 – 2.65 (m, 2H), 2.63 – 2.51 (m, 4H), 2.51 – 2.44 (m, 1H), 2.26 – 2.16 (m, 1H). ^{13}C NMR (101 MHz, $CDCl_3$) δ 206.94, 174.31, 172.70, 138.11, 129.05, 125.42, 121.40, 67.29, 51.80, 33.23, 30.74, 27.05, 21.64.

FT-IR (film): 3448, 2956, 1687, 1598, 1260, 755 cm^{-1}.

HRMS (ESI-MS) *m/z* $[M+H]^+$ calcd for $C_{15}H_{18}NO_4$: 276.1230, found: 276.1227.

$[\alpha]^{25}_D$ = +38.9 (*c* 1.0, CH_2Cl_2); 88% ee from (*S*, *R*)-**L2**.

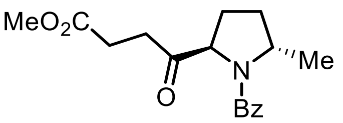

23

Methyl 4-((2*R*,5*S*)-1-benzoyl-5-methylpyrrolidin-2-yl)-4-oxobutanoate (**23**, from (*S*, *R*)-**L2**). The title compound was synthesized according to **GP-B** from 4-methoxy-4-oxobutanoic acid and (*S*)-(2-methylpyrrolidin-1-yl)(phenyl)methanone. The product was purified by preparative TLC on silica gel (1:4 Acetone/Petroleum ether). Colorless oil.

(*S*, *R*)-**L2**: 38.9 mg, 64% yield, 99:1 dr; (*R*, *S*)-**L2**: 32.3 mg, 53% yield, 34:66 dr.

HPLC analysis: The dr was determined via HPLC on a CHIRALPAK AD-3 column (25.0% 2-PrOH in hexanes, 0.8 mL/min); retention times for compound obtained using (*S*, *R*)-**L2** 6.8 min (major), 11.2 min (minor).

^{1}H NMR (500 MHz, $CDCl_3$) (76:24 mixture of rotamers) δ 7.52 – 7.44 (m, 1.52H), 7.41 – 7.34 (m, 2.24H), 7.33 – 7.26 (m, 1.24H), 4.82 (dd, *J* = 9.0, 2.8 Hz, 0.76H), 4.64 – 4.50 (m, 0.48H), 4.29 – 4.10 (m, 0.76H), 3.65 (s, 2.28H), 3.57 (s, 0.72H), 2.99 – 2.91 (m, 0.76H), 2.88 – 2.81 (m, 0.76H), 2.79 – 2.71 (m, 0.76H), 2.60 – 2.50 (m, 1.00H), 2.45 – 2.32 (m, 0.48H), 2.30 – 2.11 (m, 1.76H), 2.11 – 1.95 (m, 1.48H), 1.61 – 1.55 (m, 1.00H), 1.31 (d, *J* = 6.4 Hz, 0.72H), 0.86 (d, *J* = 6.4 Hz, 2.28H). ^{13}C NMR (126 MHz, $CDCl_3$) δ 206.48, 206.45, 173.24, 172.60, 170.43, 170.21, 137.78, 136.90, 129.52, 129.23, 128.30, 128.18, 126.74, 126.44, 67.25, 64.52, 55.31, 53.85, 51.66, 34.63, 33.86, 31.80, 29.48, 27.61, 27.30, 26.99, 25.43, 21.17, 19.86.

FT-IR (film): 2970, 1727, 1628, 1409, 1210, 704 cm^{-1}.

HRMS (ESI-MS) *m/z* $[M+H]^+$ calcd for $C_{17}H_{22}NO_4$: 304.1543, found: 304.1542.

$[\alpha]^{25}_{D}$ = +90.5 (*c* 1.0, CH_2Cl_2); 99:1 dr from (*S*, *R*)-**L2**.

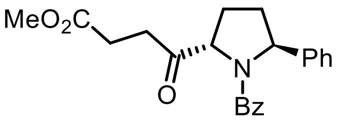

24

Methyl 4-((2*S*,5*S*)-1-benzoyl-5-phenylpyrrolidin-2-yl)-4-oxobutanoate (**24**, from (*R*, *S*)-**L2**). The title compound was synthesized according to **GP-B** from 4-methoxy-4-oxobutanoic acid and (*S*)-phenyl(2-phenylpyrrolidin-1-yl)methanone. The products were purified by preparative TLC on silica gel (1:4 Acetone/Petroleum ether). Yellow oil.

(*S*, *R*)-**L2**: 37.4 mg, 51% yield, 96:4 dr; (*R*, *S*)-**L2**: 47.3 mg, 65% yield, >99:1 dr.

HPLC analysis: The dr was determined via HPLC on a CHIRALCEL OD-3 column (20.0% 2-PrOH in hexanes, 1.0 mL/min); retention times for compound obtained using (*R*, *S*)-**L2**: 12.9 min (major), 14.4 min (minor).

^{1}H NMR (500 MHz, $CDCl_3$) (83:17 mixture of rotamers) δ 7.49 – 7.28 (m, 1.66H), 7.25 – 7.18 (m, 3.00H), 7.18 – 7.06 (m, 4.00H), 6.98 – 6.95 (m, 1.34H), 5.65 – 5.56 (m, 0.17H), 5.17 – 4.95 (m, 1.66H), 4.89 – 4.83 (m, 0.17H), 3.71 – 3.59 (m, 3.00H), 3.09 – 2.92 (m, 1.66H), 2.89 – 2.78 (m, 0.83H), 2.70 – 2.54 (m, 1.17H), 2.53 – 2.43 (m, 1.00H), 2.34 – 2.13 (m, 1.34H), 2.09 – 2.02 (m, 1.00H), 1.89 – 1.77 (m, 1.00H). ^{13}C NMR (126 MHz, $CDCl_3$) δ 206.36, 206.15, 173.31, 172.64, 170.97, 170.49, 143.50, 142.77, 137.43, 136.34, 129.54, 129.38, 128.47, 128.25, 127.78, 127.04, 126.99, 126.88, 126.36, 125.43, 125.39, 67.73, 65.50, 63.87, 61.09, 51.71, 34.92, 34.10, 34.02, 31.52, 27.52, 27.35, 27.01, 25.10.

FT-IR (film): 3442, 2922, 1724, 1631, 1397, 698 cm^{-1}.

HRMS (ESI-MS) *m/z* $[M+H]^+$ calcd for $C_{22}H_{24}NO_4$: 366.1700, found: 366.1697.

$[\alpha]^{25}_D$ = –122.3 (*c* 1.0, CH_2Cl_2); >99:1 dr from (*R*, *S*)-**L2**.

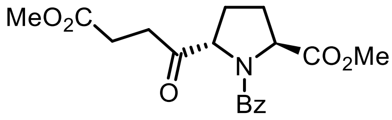

25

Methyl (2*S*,5*S*)-1-benzoyl-5-(4-methoxy-4-oxobutanoyl)pyrrolidine-2-carboxylate (**25**, from (*R*, *S*)-**L2**). The title compound was synthesized according to **GP-B** from methyl 4-methoxy-4-oxobutanoic acid and methyl benzoyl-*L*-prolinate. The product was purified by preparative TLC on silica gel (1:3 Acetone/Petroleum ether). Colorless oil.

(*S*, *R*)-**L2**: 45.3 mg, 65% yield, 52:48 dr; (*R*, *S*)-**L2**: 49.0 mg, 71% yield, 1:99 dr.

HPLC analysis: The dr was determined via HPLC on a CHIRALPAK AD-3 column (30.0% 2-PrOH in hexanes, 0.8 mL/min); retention times for compound obtained using (*R*, *S*)-**L2**: 13.7 min (minor), 26.9 min (major).

^{1}H NMR (500 MHz, $CDCl_3$) (64:36 mixture of rotamers) δ 7.39 – 7.30 (m, 5.00H), 4.95 (dd, *J* = 9.2, 2.5 Hz, 0.64H), 4.85 (dd, *J* = 9.1, 2.0 Hz, 0.36H), 4.63 (dd, *J* = 9.3, 1.8 Hz, 0.36H), 4.47 (dd, *J* = 8.6, 1.8 Hz, 0.64H), 3.77 (s, 1.08H), 3.65 (s, 1.92H), 3.60 (s, 1.08H), 3.51 (s, 1.92H), 3.01 – 2.94 (m, 0.64H), 2.92 – 2.85 (m, 0.64H), 2.82 – 2.75 (m, 0.64H), 2.66 – 2.49 (m, 1.44H), 2.42 – 2.30 (m, 1.00H), 2.27 – 2.22 (m, 0.64H), 2.22 – 2.16 (m, 0.64H), 2.13 – 2.05 (m, 1.00H), 2.04 – 1.98 (m, 1.00H), 1.78 – 1.72 (m, 0.36H). ^{13}C NMR (126 MHz, $CDCl_3$) δ 206.47, 206.29, 173.23, 172.61, 172.37, 172.36, 170.47, 170.22, 136.61, 136.06, 129.88, 129.68, 128.34, 126.50, 126.38, 66.91, 64.83, 61.72, 59.44, 52.30, 52.24, 51.76, 51.71, 34.79, 34.03, 29.83, 28.30, 27.29, 27.07, 26.98, 26.03.

FT-IR (film): 2954, 1739, 1644, 1404, 1210, 704 cm^{-1}.

HRMS (ESI-MS) *m/z* $[M+H]^+$ calcd for $C_{18}H_{22}NO_6$: 348.1442, found: 348.1437.

$[\alpha]^{25}_D$ = –104.2 (*c* 1.0, CH_2Cl_2); 1:99 dr from (*R*, *S*)-**L2**.

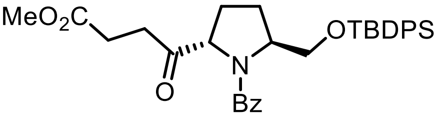

26

Methyl 4-((2*S*,5*R*)-1-benzoyl-5-((tert-butyldiphenylsilyl)oxy)pyrrolidin-2-yl)-4-oxobutanoate (**26**, from (*R*, *S*)-**L2**). The title compound was synthesized according to **GP-B** from 4-methoxy-4-oxobutanoic acid and (*R*)-(2-((*tert*-butyldiphenylsilyl)oxy)pyrrolidin-1-yl)(phenyl)methanone. The product was purified by preparative TLC on silica gel (1:5 Acetone/Petroleum ether). Colorless oil.

(*S*, *R*)-**L2**: 58.0 mg, 52% yield, 67:33 dr; (*R*, *S*)-**L2**: 61.1 mg, 55% yield, 1:99 dr.

HPLC analysis: The dr was determined via HPLC on a CHIRALPAK AD-3 column (15.0% 2-PrOH in hexanes, 1.0 mL/min); retention times for compound obtained using (*R*, *S*)-**L2**: 7.1 min (minor), 10.1 min (major).

^{1}H NMR (400 MHz, $CDCl_3$) (65:35 mixture of rotamers) δ 7.74 – 7.63 (m, 1.35H), 7.55 – 7.50 (m, 1.35H), 7.46 – 7.27 (m, 12.30H), 4.86 (dd, *J* = 9.7, 1.9 Hz, 0.65H), 4.63 (d, *J* = 9.2 Hz, 0.35H), 4.24 – 4.16 (m, 0.65H), 4.09 – 3.96 (m, 0.35H), 3.89 – 3.80 (m, 0.35H), 3.71 – 3.56 (m, 3.35H), 3.34

– 3.27 (m, 0.65H), 3.24 – 3.18 (m, 0.65H), 3.04 – 2.93 (m, 0.65H), 2.89 – 2.75 (m, 1.35H), 2.64 – 2.38 (m, 2.00H), 2.36 – 2.27 (m, 0.65H), 2.23 – 2.14 (m, 1.00H), 2.12 – 2.04 (m, 1.00H), 2.04 – 1.96 (m, 1.00H), 1.65 – 1.54 (m, 0.35H), 1.11 (s, 3.15H), 0.97 (s, 5.85H). ^{13}C NMR (126 MHz, $CDCl_3$) δ 206.71, 206.62, 173.23, 172.59, 170.92, 170.20, 137.66, 136.51, 135.49, 135.47, 135.41, 135.25, 133.43, 132.82, 129.77, 129.69, 129.65, 129.47, 129.35, 128.28, 128.19, 127.81, 127.73, 127.70, 127.67, 126.89, 126.30, 67.87, 65.15, 64.72, 64.19, 60.42, 59.41, 51.67, 34.70, 33.87, 28.48, 27.33, 27.32, 27.02, 26.89, 26.69, 25.67, 25.46, 19.27, 19.00.

FT-IR (film): 2953, 1736, 1633, 1407, 1121, 703 cm^{-1}.

HRMS (ESI-MS) *m/z* [M+H]$^+$ calcd for $C_{33}H_{40}NO_5Si$: 558.2670, found: 558.2672.

$[\alpha]^{25}_{D}$ = –63.6 (*c* 1.0, CH_2Cl_2); 1:99 dr from (*R*, *S*)-**L2**.

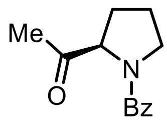

27

(*R*)-1-(1-Benzoylpyrrolidin-2-yl)ethan-1-one (**27**, from (*S*, *R*)-**L1**). The title compound was synthesized according to **GP-B** from acetic acid and *N*-Bz-pyrrolidine. The product was purified by preparative TLC on silica gel (1:4 Acetone/Petroleum ether). Yellow solid.

(*S*, *R*)-**L1**: 31.6 mg, 73% yield, 94% ee; (*R*, *S*)-**L1**: 32.5 mg, 75% yield, 94% ee.

HPLC analysis: The ee was determined via HPLC on a CHIRALPAK AD-3 column (25.0% 2-PrOH in hexanes, 0.8 mL/min); retention times for compound obtained using (*S*, *R*)-**L1**: 5.6 min (major), 7.7 min (minor).

^{1}H NMR (500 MHz, $CDCl_3$) (87:13 mixture of rotamers) δ 7.57 – 7.52 (m, 1.74H), 7.43 – 7.38 (m, 2.26H), 7.38 – 7.28 (m, 1.00H), 4.73 (dd, *J* = 8.5, 6.0 Hz, 0.87H), 4.42 (dd, *J* = 8.6, 1.6 Hz, 0.13H), 3.77 (t, *J* = 6.4 Hz, 0.26H), 3.63 – 3.58 (m, 0.87H), 3.56 – 3.50 (m, 0.87H), 2.31 – 2.19 (m, 3.74H), 1.99 – 1.92 (m, 1.26H), 1.90 – 1.83 (m, 2.00H). ^{13}C NMR (126 MHz, $CDCl_3$) δ 206.61, 169.61, 137.17, 135.99, 130.18, 129.58, 128.36, 128.20, 127.18, 126.45, 67.47, 65.24, 50.13, 46.64, 29.84, 28.11, 27.01, 26.28, 25.31, 22.45.

FT-IR (film): 3440, 1716, 1625, 1422, 1162, 702 cm^{-1}.

HRMS (ESI-MS) *m/z* [M+H]$^+$ calcd for $C_{13}H_{16}NO_2$: 218.1176, found: 218.1174.

$[\alpha]^{25}_{D}$ = +48.3 (*c* 1.0, CH_2Cl_2); 94% ee from (*S*, *R*)-**L1**.

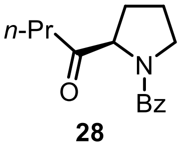

28

(*R*)-1-(1-Benzoylpyrrolidin-2-yl)butan-1-one (**28**, from (*S*, *R*)-**L1**). The title compound was synthesized according to **GP-B** from butyric acid and *N*-Bz-pyrrolidine. The product was purified by preparative TLC on silica gel (2:3 EtOAc/ Petroleum ether). Yellow oil.

(*S*, *R*)-**L1**: 32.5 mg, 66% yield, 93% ee; (*R*, *S*)-**L1**: 32.0 mg, 65% yield, 94% ee.

HPLC analysis: The ee was determined via HPLC on a CHIRALPAK AD-3 column (25.0% 2-PrOH in hexanes, 0.8 mL/min); retention times for compound obtained using (*S*, *R*)-**L1**: 5.4 min (major), 8.0 min (minor).

^{1}H NMR (400 MHz, $CDCl_3$) (85:15 mixture of rotamers) δ 7.59 – 7.51 (m, 1.70H), 7.45 – 7.33 (m, 2.70H), 7.36 – 7.27 (m, 0.60H), 4.80 – 4.68 (m, 0.85H), 4.42 (dd, *J* = 8.8, 1.8 Hz, 0.15H), 3.81 – 3.74 (m, 0.30H), 3.66 – 3.56 (m, 0.85H), 3.58 – 3.47 (m, 0.85H), 2.71 – 2.59 (m, 0.85H), 2.58 – 2.46 (m, 0.85H), 2.31 – 2.10 (m, 1.30H), 2.00 – 1.91 (m, 1.00H), 1.90 – 1.79 (m, 2.00H), 1.73 – 1.60 (m, 1.70H), 1.35 – 1.27 (m, 0.30H), 0.93 (t, *J* = 7.4 Hz, 2.55H), 0.70 (t, *J* = 7.4 Hz, 0.45H). ^{13}C NMR (101 MHz, $CDCl_3$) δ 208.70, 169.51, 136.11, 130.11, 129.46, 128.31, 128.17, 127.19, 126.50, 67.01, 64.74, 50.19, 46.68, 41.90, 40.99, 29.93, 28.27, 25.37, 22.44, 16.72, 16.61, 13.70, 13.49.

FT-IR (film): 3436, 2964, 1721, 1626, 1418, 702 cm^{-1}.

HRMS (ESI-MS) *m/z* $[M+H]^+$ calcd for $C_{15}H_{20}NO_2$: 246.1489, found: 246.1486.

$[\alpha]^{25}_D$ = +39.2 (*c* 1.0, CH_2Cl_2); 93% ee from (*S*, *R*)-**L1**.

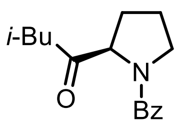

29

(*R*)-1-(1-Benzoylpyrrolidin-2-yl)-3-methylbutan-1-one (**29**, from (*S*, *R*)-**L1**). The title compound was synthesized according to **GP-B** from 3-methylbutanoic acid and *N*-Bz-pyrrolidine. The product was purified by preparative TLC on silica gel (1:4 Acetone/Petroleum ether). Colorless oil.

(*S*, *R*)-**L1**: 38.0 mg, 73% yield, 93% ee; (*R*, *S*)-**L1**: 38.0 mg, 73% yield, 94% ee.

HPLC analysis: The ee was determined via HPLC on a CHIRALPAK AD-3 column (20.0% 2-PrOH in hexanes, 1.0 mL/min); retention times for compound obtained using (*S*, *R*)-**L1**: 5.8 min (major), 8.8 min (minor).

^{1}H NMR (400 MHz, $CDCl_3$) (85:15 mixture of rotamers) δ 7.60 – 7.51 (m, 1.70H), 7.43 – 7.35 (m, 2.55H), 7.34 – 7.29 (m, 0.75H), 4.83 – 4.63 (m, 0.85H), 4.41 (dd, *J* = 8.8, 2.1 Hz, 0.15H), 3.80 – 3.72 (m, 0.30H), 3.64 – 3.56 (m, 0.85H), 3.55 – 3.47 (m, 0.85H), 2.59 – 2.51 (m, 0.85H), 2.44 – 2.36 (m, 0.85H), 2.29 – 2.18 (m, 1.85H), 2.13 – 2.02 (m, 0.30H), 2.01 – 1.79 (m, 3.85H), 1.78 – 1.60 (m, 0.30H), 0.94 (dd, *J* = 11.2, 6.6 Hz, 5.10H), 0.72 (d, *J* = 6.6 Hz, 0.45H), 0.65 (d, *J* = 6.6 Hz, 0.45H). ^{13}C NMR (101 MHz, $CDCl_3$) δ 208.08, 169.51, 136.20, 130.08, 129.45, 128.32, 128.17, 127.19, 126.56, 67.33, 64.97, 50.18, 48.98, 47.98, 46.62, 29.80, 28.06, 25.32, 23.89, 23.66, 22.59, 22.53, 22.40, 22.25.

FT-IR (film): 3448, 2956, 1720, 1628, 1417, 1058, 702 cm^{-1}.

HRMS (ESI-MS) *m/z* $[M+H]^+$ calcd for $C_{16}H_{22}NO_2$: 260.1645, found: 260.1643.

$[\alpha]^{25}_D$ = +72.8 (*c* 1.0, CH_2Cl_2); 93% ee from (*S*, *R*)-**L1**.

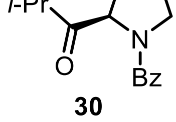

30

(*R*)-1-(1-Benzoylpyrrolidin-2-yl)-2-methylpropan-1-one (**30**, from (*S*, *R*)-**L1**). The title compound was synthesized according to **GP-B** from isobutyric acid and *N*-Bz-pyrrolidine. The product was purified by preparative TLC on silica gel (1:3 EtOAc/Petroleum ether). Colorless oil.

(*S*, *R*)-**L1**: 28.2 mg, 57% yield, 86% ee; (*R*, *S*)-**L1**: 28.6 mg, 58% yield, 87% ee.

HPLC analysis: The ee was determined via HPLC on a CHIRALPAK AD-3 column (15.0% 2-PrOH in hexanes, 1.0 mL/min); retention times for compound obtained using (*S*, *R*)-**L1**: 6.8 min (major), 8.9 min (minor).

^{1}H NMR (500 MHz, $CDCl_3$) (81:19 mixture of rotamers) δ 7.59 – 7.50 (m, 1.62H), 7.45 – 7.26 (m, 3.38H), 4.89 (dd, *J* = 8.2, 6.1 Hz, 0.81H), 4.59 (dd, *J* = 9.2, 2.8 Hz, 0.19H), 3.84 – 3.70 (m, 0.38H), 3.67 – 3.58 (m, 0.81H), 3.56 – 3.45 (m, 0.81H), 3.02 – 2.81 (m, 0.81H), 2.37 – 2.18 (m, 1.19H), 2.03 – 1.92 (m, 1.00H), 1.89 – 1.78 (m, 2.00H), 1.22 (d, *J* = 6.9 Hz, 2.43H), 1.15 (d, *J* = 6.9 Hz, 2.43H), 0.91 (d, *J* = 6.8 Hz, 0.57H), 0.46 (d, *J* = 7.0 Hz, 0.57H). ^{13}C NMR (126 MHz, $CDCl_3$) δ 212.52, 211.50, 169.26, 137.51, 136.20, 130.04, 129.33, 128.31, 128.14, 127.21, 126.59, 65.80, 63.28, 50.16, 46.61, 39.05, 37.31, 29.89, 28.74, 25.49, 22.41, 18.40, 18.04, 17.94, 17.72.

FT-IR (film): 3483, 2970, 1719, 1629, 1413, 1049, 702 cm^{-1}.

HRMS (ESI-MS) *m/z* $[M+H]^+$ calcd for $C_{15}H_{20}NO_2$: 246.1489, found: 246.1487.

$[\alpha]^{25}_D$ = +59.6 (*c* 1.0, CH_2Cl_2); 86% ee from (*S*, *R*)-**L1**.

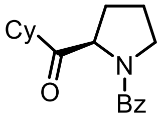

31

(*R*)-(1-Benzoylpyrrolidin-2-yl)(cyclohexyl)methanone (**31**, from (*S*, *R*)-**L1**). The title compound was synthesized according to **GP-B** from cyclohexanecarboxylic acid and *N*-Bz-pyrrolidine. The product was purified by preparative TLC on silica gel (2:3 EtOAc/Petroleum ether). Yellow solid.

(*S*, *R*)-**L1**: 36.4 mg, 64% yield, 81% ee; (*R*, *S*)-**L1**: 37.2 mg, 65% yield, 85% ee.

HPLC analysis: The ee was determined via HPLC on a CHIRALPAK AD-3 column (15.0% 2-PrOH in hexanes, 1.0 mL/min); retention times for compound obtained using (*S*, *R*)-**L1**: 7.8 min (major), 11.0 min (minor).

^{1}H NMR (400 MHz, $CDCl_3$) (81:19 mixture of rotamers) 7.61 – 7.47 (m, 1.62H), 7.46 – 7.26 (m, 3.38H), 4.87 (dd, *J* = 8.5, 5.8 Hz, 0.81H), 4.56 (dd, *J* = 9.1, 2.7 Hz, 0.19H), 3.83 – 3.69 (m, 0.38H), 3.67 – 3.58 (m, 0.81H), 3.55 – 3.46 (m, 0.81H), 2.75 – 2.57 (m, 0.81H), 2.29 – 2.17 (m, 1.00H), 2.14 – 2.02 (m, 1.00H), 2.01 – 1.90 (m, 1.19H), 1.87 – 1.75 (m, 4.19H), 1.69 – 1.63 (m, 0.81H), 1.57 – 1.38 (m, 2.00H), 1.38 – 1.18 (m, 3.00H), 1.14 – 0.91 (m, 0.81H), 0.75 – 0.62 (m, 0.19H). ^{13}C NMR (126 MHz, $CDCl_3$) δ 211.76, 210.65, 170.54, 169.19, 137.52, 136.19, 130.02, 129.28, 128.27, 128.12, 127.22, 126.62, 65.92, 63.32, 50.17, 49.06, 47.38, 46.57, 29.77, 28.67, 28.55, 28.27, 28.14, 27.74, 25.80, 25.65, 25.60, 25.50, 25.40, 25.11, 22.39.

FT-IR (film): 3478, 2929, 1716, 1628, 1416, 1143, 701 cm^{-1}.

HRMS (ESI-MS) *m/z* $[M+H]^+$ calcd for $C_{18}H_{24}NO_2$: 286.1802, found: 286.1799.

$[\alpha]^{25}_D$ = +46.7 (*c* 1.0, CH_2Cl_2); 81% ee from (*S*, *R*)-**L1**.

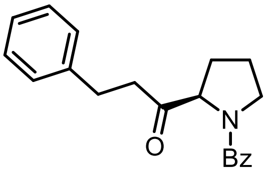

32

(*R*)-1-(1-Benzoylpyrrolidin-2-yl)-3-phenylpropan-1-one (**32**, from (*S*, *R*)-**L1**). The title compound was synthesized according to **GP-B** from 3-phenylpropanoic acid and *N*-Bz-pyrrolidine. The product was purified by preparative TLC on silica gel (2:3 EtOAc/Petroleum ether). Yellow oil.

(*S*, *R*)-**L1**: 39.6 mg, 64% yield, 96% ee; (*R*, *S*)-**L1**: 43.5 mg, 71% yield, 97% ee.

HPLC analysis: The ee was determined via HPLC on a CHIRALCEL OD-3 column (20.0% 2-PrOH in hexanes, 1.0 mL/min); retention times for compound obtained using (*S*, *R*)-**L1**: 12.5 min (major), 14.4 min (minor).

^{1}H NMR (500 MHz, $CDCl_3$) (88:12 mixture of rotamers) δ 7.63 – 7.53 (m, 2.00H), 7.45 – 7.34 (m, 3.00H), 7.32 – 7.27 (m, 1.76H), 7.25 – 7.16 (m, 3.00H), 7.04 (d, *J* = 7.5 Hz, 0.24H), 4.74 (dd, *J* = 8.4, 6.2 Hz, 0.88H), 4.41 (dd, *J* = 9.0, 3.2 Hz, 0.12H), 3.79 – 3.74 (m, 0.24H), 3.62 – 3.55 (m, 0.88H), 3.55 – 3.49 (m, 0.88H), 3.06 – 2.95 (m, 2.64H), 2.92 – 2.84 (m, 0.88H), 2.72 – 2.65 (m, 0.12H), 2.62 – 2.51 (m, 0.36H), 2.20 – 2.12 (m, 1.00H), 1.95 – 1.87 (m, 1.00H), 1.86 – 1.77 (m, 1.00H), 1.76 – 1.68 (m, 1.00H). ^{13}C NMR (126 MHz, $CDCl_3$) δ 207.64, 207.56, 170.25, 169.53, 141.09, 140.41, 136.55, 135.93, 130.12, 129.98, 129.46, 128.33, 128.26, 128.14, 127.13, 126.42, 126.13, 125.93, 66.96, 64.82, 50.11, 46.64, 41.54, 40.94, 29.61, 29.27, 29.02, 27.91, 25.24, 22.35.

FT-IR (film): 3443, 1722, 1626, 1448, 1418, 700 cm^{-1}.

HRMS (ESI-MS) *m/z* [M+Na]$^+$ calcd for $C_{20}H_{21}NNaO_2$: 330.1465, found: 330.1459.

$[\alpha]^{25}_D$ = +46.7 (*c* 1.0, CH_2Cl_2); 96% ee from (*S*, *R*)-**L1**.

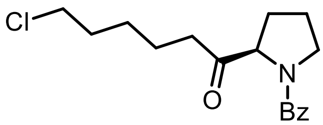

33

(*R*)-1-(1-Benzoylpyrrolidin-2-yl)-6-chlorohexan-1-one (**33**, from (*S*, *R*)-**L1**). The title compound was synthesized according to **GP-B** from 6-chlorohexanoic acid and *N*-Bz-pyrrolidine. The product was purified by preparative TLC on silica gel (1:1 EtOAc/Petroleum ether). Yellow oil.

(*S*, *R*)-**L1**: 43.6 mg, 71% yield, 97% ee; (*R*, *S*)-**L1**: 44.6 mg, 72% yield, 96% ee.

HPLC analysis: The ee was determined via HPLC on a CHIRALPAK AD-3 column (20.0% 2-PrOH in hexanes, 1.0 mL/min); retention times for compound obtained using (*S*, *R*)-**L1**: 9.8 min (major), 19.2 min (minor).

^{1}H NMR (400 MHz, $CDCl_3$) (87:13 mixture of rotamers) δ 7.58 – 7.51 (m, 1.74H), 7.42 – 7.28 (m, 3.26H), 4.78 – 4.66 (m, 0.87H), 4.44 (dd, *J* = 8.9, 2.8 Hz, 0.13H), 3.93 – 3.68 (m, 0.39H), 3.67 – 3.57 (m, 1.00H), 3.57 – 3.47 (m, 2.61H), 3.44 (t, *J* = 6.7 Hz, 0.26H), 2.75 – 2.65 (m, 0.87H), 2.60 – 2.51 (m, 0.87H), 2.27 – 2.18 (m, 1.00H), 2.00 – 1.92 (m, 1.00H), 1.89 – 1.82 (m, 2.00H), 1.80 – 1.74 (m, 1.74H), 1.71 – 1.60 (m, 2.00H), 1.51 – 1.41 (m, 1.74H), 1.29 – 1.25 (m, 0.26H), 1.21 – 1.15 (m, 0.26H). ^{13}C NMR (101 MHz, $CDCl_3$) δ 208.50, 208.17, 170.35, 169.54, 137.34, 136.01, 130.16,

129.49, 128.33, 128.20, 127.18, 126.55, 66.92, 64.75, 50.19, 46.70, 44.80, 44.56, 39.70, 38.87, 32.35, 32.10, 30.03, 28.33, 26.35, 26.10, 25.39, 22.44, 22.33.

FT-IR (film): 3473, 2934, 1721, 1627, 1413, 722 cm^{-1}.

HRMS (ESI-MS) *m/z* $[M+H]^+$ calcd for $C_{17}H_{23}ClNO_2$: 308.1412, found: 308.1409.

$[\alpha]^{25}_D$ = +64.5 (*c* 1.0, CH_2Cl_2); 97% ee from (*S*, *R*)-**L1**.

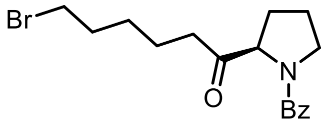

34

(*R*)-1-(1-Benzoylpyrrolidin-2-yl)-6-bromohexan-1-one (**34**, from (*S*, *R*)-**L1**). The title compound was synthesized according to **GP-B** from 6-bromohexanoic acid and *N*-Bz-pyrrolidine. The product was purified by preparative TLC on silica gel (1:2 EtOAc/Petroleum ether). Brown oil.

(*S*, *R*)-**L1**: 42.2 mg, 60% yield, 96% ee; (*R*, *S*)-**L1**: 42.1 mg, 60% yield, 96% ee.

HPLC analysis: The ee was determined via HPLC on a CHIRALPAK AD-3 column (20.0% 2-PrOH in hexanes, 1.0 mL/min); retention times for compound obtained using (*S*, *R*)-**L1**: 8.7 min (major), 17.5 min (minor).

^{1}H NMR (400 MHz, $CDCl_3$) (86:14 mixture of rotamers) δ 7.59 – 7.48 (m, 1.72H), 7.45 – 7.26 (m, 3.28H), 4.72 (dd, *J* = 8.5, 5.9 Hz, 0.86H), 4.50 – 4.37 (m, 0.14H), 3.82 – 3.71 (m, 0.28H), 3.65 – 3.58 (m, 0.86H), 3.56 – 3.49 (m, 0.86H), 3.44 – 3.22 (m, 2.00H), 2.77 – 2.65 (m, 0.86H), 2.61 – 2.50 (m, 0.86H), 2.31 – 2.17 (m, 1.14H), 2.01 – 1.93 (m, 1.14H), 1.90 – 1.79 (m, 4.00H), 1.70 – 1.62 (m, 1.72H), 1.52 – 1.41 (m, 1.72H), 1.31 – 1.24 (m, 0.28H), 1.22 – 1.14 (m, 0.28H). ^{13}C NMR (101 MHz, $CDCl_3$) δ 208.49, 208.16, 170.35, 169.54, 137.33, 135.99, 130.17, 129.50, 128.34, 128.20, 127.18, 126.54, 66.92, 64.75, 50.19, 46.71, 39.67, 38.84, 33.61, 33.30, 32.52, 32.24, 30.03, 28.33, 27.62, 27.36, 25.39, 22.50, 22.31, 22.19.

FT-IR (film): 3445, 2936, 1720, 1627, 1416, 701 cm^{-1}.

HRMS (ESI-MS) *m/z* $[M+H]^+$ calcd for $C_{17}H_{23}BrNO_2$: 352.0907, found: 352.0903.

$[\alpha]^{25}_D$ = +48.3 (*c* 1.0, CH_2Cl_2); 96% ee from (*S*, *R*)-**L1**.

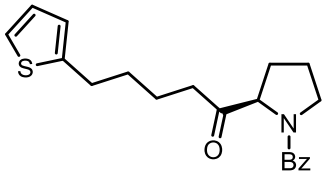

35

(*R*)-1-(1-Benzoylpyrrolidin-2-yl)-5-(thiophen-2-yl)pentan-1-one (**35**, from (*S*, *R*)-**L1**). The title compound was synthesized according to **GP-B** from 5-(thiophen-2-yl)pentanoic acid and *N*-Bz-pyrrolidine. The product was purified by preparative TLC on silica gel (2:3 EtOAc/Petroleum ether). Yellow oil.

(*S*, *R*)-**L1**: 30.1 mg, 45% yield, 97% ee; (*R*, *S*)-**L1**: 31.3 mg, 46% yield, 97% ee.

HPLC analysis: The ee was determined via HPLC on a CHIRALPAK AD-3 column (20.0% 2-PrOH in hexanes, 1.0 mL/min); retention times for compound obtained using (*S*, *R*)-**L1**: 8.4 min (major), 18.4 min (minor).

^{1}H NMR (400 MHz, $CDCl_3$) (85:15 mixture of rotamers) δ 7.60 – 7.49 (m, 1.70H), 7.46 – 7.33 (m, 2.70H), 7.32 – 7.28 (m, 0.60H), 7.13 – 7.03 (m, 1.00H), 6.95 – 6.85 (m, 1.00H), 6.80 – 6.70 (m, 1.00H), 4.74 (dd, *J* = 8.6, 5.8 Hz, 0.85H), 4.44 (dd, *J* = 8.7, 2.9 Hz, 0.15H), 3.82 – 3.73 (m, 0.30H), 3.67 – 3.57 (m, 0.85H), 3.57 – 3.49 (m, 0.85H), 2.94 – 2.79 (m, 1.70H), 2.78 – 2.64 (m, 1.30H), 2.63 – 2.49 (m, 1.00H), 2.27 – 2.18 (m, 1.00H), 2.02 – 1.92 (m, 1.00H), 1.88 – 1.81 (m, 2.00H), 1.76 – 1.68 (m, 3.40H), 1.46 – 1.40 (m, 0.30H), 1.38 – 1.31 (m, 0.30H). ^{13}C NMR (126 MHz, $CDCl_3$) major isomer δ 208.48, 169.53, 145.05, 136.07, 130.14, 128.19, 127.19, 126.63, 124.08, 122.78, 64.74, 50.17, 39.65, 31.22, 29.68, 28.31, 25.38, 22.69.

FT-IR (film): 3448, 2923, 1717, 1626, 1410, 698 cm^{-1}.

HRMS (ESI-MS) *m/z* $[M+H]^+$ calcd for $C_{20}H_{24}NO_2S$: 342.1522, found: 342.1518.

$[\alpha]^{25}_D$ = +60.7 (*c* 1.0, CH_2Cl_2); 97% ee from (*S*, *R*)-**L1**.

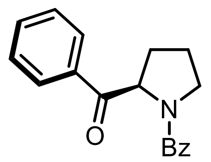

36

(*R*)-Pyrrolidine-1,2-diylbis(phenylmethanone) (**36**, from (*S*, *R*)-**L2**). The title compound was synthesized according to **GP-B** from benzoic acid and *N*-Bz-pyrrolidine. The product was purified by preparative TLC on silica gel (3:2 EtOAc/Petroleum ether). White solid.

(*S*, *R*)-**L2**: 45.2 mg, 81% yield, 96% ee; (*R*, *S*)-**L2**: 47.4 mg, 85% yield, 96% ee.

HPLC analysis: The ee was determined via HPLC on a CHIRALCEL OD-3 column (15.0% 2-PrOH in hexanes, 1.0 mL/min); retention times for compound obtained using (*S*, *R*)-**L2**: 10.8 min (major), 17.6 min (minor).

^{1}H NMR (400 MHz, $CDCl_3$) (84:16 mixture of rotamers) δ 8.15 – 7.98 (m, 1.68H), 7.64 – 7.54 (m, 2.84H), 7.52 – 7.45 (m, 1.84H), 7.43 – 7.29 (m, 3.16H), 7.22 – 7.16 (m, 0.48H), 5.70 (dd, *J* = 8.7, 4.7 Hz, 0.84H), 5.28 – 5.25 (m, 0.16H), 3.92 – 3.85 (m, 0.32H), 3.77 – 3.68 (m, 0.84H), 3.65 – 3.56 (m, 0.84H), 2.45 – 2.35 (m, 0.84H), 2.06 – 1.89 (m, 3.16H). ^{13}C NMR (101 MHz, $CDCl_3$) δ 198.25, 197.67, 170.37, 169.23, 136.31, 135.32, 134.24, 133.39, 133.21, 129.99, 129.40, 128.59, 128.48, 128.24, 128.14, 127.92, 127.25, 126.42, 63.94, 61.31, 50.05, 46.71, 31.11, 29.39, 25.31, 22.34.

FT-IR (film): 3445, 1691, 1627, 1418, 1224, 702 cm^{-1}.

HRMS (ESI-MS) *m/z* $[M+H]^+$ calcd for $C_{18}H_{18}NO_2$: 280.1332, found: 280.1329.

$[\alpha]^{25}_D$ = +27.1 (*c* 1.0, CH_2Cl_2); 96% ee from (*S*, *R*)-**L2**.

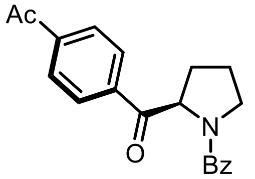

37

1-(4-(Benzoyl-*D*-prolyl)phenyl)ethan-1-one (**37**, from (*S*, *R*)-**L2**). The title compound was synthesized according to **GP-B** from 4-acetylbenzoic acid and *N*-Bz-pyrrolidine. The product was purified by preparative TLC on silica gel (1:4 Acetone/Petroleum ether). Yellow oil.

(*S*, *R*)-**L2**: 30.7 mg, 48% yield, 98% ee; (*R*, *S*)-**L2**: 32.0 mg, 50% yield, 98% ee.

HPLC analysis: The ee was determined via HPLC on a CHIRALCEL OD-3 column (30.0% 2-PrOH in hexanes, 0.8 mL/min); retention times for compound obtained using (*S*, *R*)-**L2**: 16.6 min (major), 37.2 min (minor).

^{1}H NMR (400 MHz, $CDCl_3$) (85:15 mixture of rotamers) δ 8.19 – 7.96 (m, 3.55H), 7.93 – 7.78 (m, 0.45H), 7.67 – 7.57 (m, 1.85H), 7.45 – 7.37 (m, 2.55H), 7.31 – 7.26 (m, 0.30H), 7.21 – 7.17 (m, 0.30H), 5.66 (dd, *J* = 8.8, 5.1 Hz, 0.85H), 5.32 – 5.20 (m, 0.15H), 3.94 – 3.83 (m, 0.30H), 3.77 – 3.69 (m, 0.85H), 3.67 – 3.57 (m, 0.85H), 2.66 – 2.56 (m, 3.00H), 2.45 – 2.30 (m, 1.00H), 2.09 – 2.01 (m, 1.00H), 1.99 – 1.93 (m, 2.00H). ^{13}C NMR (101 MHz, $CDCl_3$) δ 197.57, 197.42, 170.36, 169.28, 140.19, 139.44, 138.80, 137.17, 135.99, 130.16, 129.51, 128.63, 128.57, 128.45, 128.31, 128.20, 128.10, 127.25, 126.38, 64.08, 61.56, 50.07, 46.74, 30.98, 29.24, 26.79, 26.72, 25.42, 22.40.

FT-IR (film): 3448, 1686, 1625, 1420, 1265, 701 cm^{-1}.

HRMS (ESI-MS) *m/z* $[M+H]^+$ calcd for $C_{20}H_{20}NO_3$: 322.1438, found: 322.1434.

$[\alpha]^{25}_D$ = –2.3 (*c* 1.0, CH_2Cl_2); 98% ee from (*S*, *R*)-**L2**.

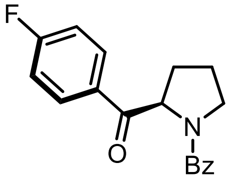

38

(*R*)-(1-Benzoylpyrrolidin-2-yl)(4-fluorophenyl)methanone (**38**, from (*S*, *R*)-**L2**). The title compound was synthesized according to **GP-B** from 4-fluorobenzoic acid and *N*-Bz-pyrrolidine. The product was purified by preparative TLC on silica gel (3:2 EtOAc/Petroleum ether). White solid.

(*S*, *R*)-**L2**: 50.5 mg, 85% yield, 96% ee; (*R*, *S*)-**L2**: 47.9 mg, 81% yield, 97% ee.

HPLC analysis: The ee was determined via HPLC on a CHIRALCEL OD-3 column (15.0% 2-PrOH in hexanes, 1.0 mL/min); retention times for compound obtained using (*S*, *R*)-**L2**: 10.1 min (major), 21.9 min (minor).

^{1}H NMR (500 MHz, $CDCl_3$) (85:15 mixture of rotamers) δ 8.13 – 8.00 (m, 1.70H), 7.65 – 7.56 (m, 2.00H), 7.47 – 7.36 (m, 2.70H), 7.30 – 7.27 (m, 0.30H), 7.21 – 7.11 (m, 2.00H), 7.01 (t, *J* = 8.4 Hz, 0.30H), 5.72 – 5.61 (m, 0.85H), 5.22 (dd, *J* = 9.1, 2.5 Hz, 0.15H), 3.91 – 3.81 (m, 0.30H), 3.75 – 3.68 (m, 0.85H), 3.64 – 3.57 (m, 0.85H), 2.41 – 2.27 (m, 1.00H), 2.06 – 1.99 (m, 1.00H), 1.98 – 1.90 (m, 2.00H). ^{13}C NMR (101 MHz, $CDCl_3$) δ 196.76, 196.31, 170.36, 169.28, 165.81 (d, $J_{C\text{-}F}$ = 254.9 Hz), 137.22, 136.15, 131.79 (d, $J_{C\text{-}F}$ = 3.2 Hz), 131.17 (d, $J_{C\text{-}F}$ = 9.3 Hz), 130.62 (d, $J_{C\text{-}F}$ = 9.3 Hz), 130.10, 129.47, 128.28, 128.18, 127.27, 126.41, 115.84 (d, $J_{C\text{-}F}$ = 22.0 Hz), 115.76 (d, $J_{C\text{-}F}$ = 22.0 Hz),

63.73, 61.13, 50.08, 46.73, 31.14, 29.37, 25.38, 22.41. ^{19}F NMR (471 MHz, $CDCl_3$) δ −103.90 (s, 0.45F), −104.80 (s, 2.55F).

FT-IR (film): 3471, 1694, 1626, 1598, 1417, 1226, 1157, 701 cm^{-1}.

HRMS (ESI-MS) *m/z* $[M+H]^+$ calcd for $C_{18}H_{17}FNO_2$: 298.1238, found: 298.1234.

$[\alpha]^{25}_D$ = +19.9 (*c* 1.0, CH_2Cl_2); 96% ee from (*S*, *R*)-**L2**.

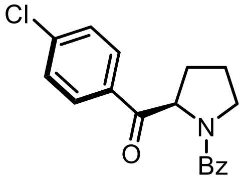

39

(*R*)-(1-Benzoylpyrrolidin-2-yl)(4-chlorophenyl)methanone (**39**, from (*S*, *R*)-**L2**). The title compound was synthesized according to **GP-B** from 4-chlorobenzoic acid and *N*-Bz-pyrrolidine. The product was purified by preparative TLC on silica gel (1:1 EtOAc/Petroleum ether). Colorless oil.

(*S*, *R*)-**L2**: 48.6 mg, 77% yield, 97% ee; (*R*, *S*)-**L2**: 51.9 mg, 83% yield, 97% ee.

HPLC analysis: The ee was determined via HPLC on a CHIRALCEL OD-3 column (15.0% 2-PrOH in hexanes, 1.0 mL/min); retention times for compound obtained using (*S*, *R*)-**L2**: 11.4 min (major), 26.0 min (minor).

^{1}H NMR (400 MHz, $CDCl_3$) (85:15 mixture of rotamers) δ 8.06 – 7.95 (m, 1.70H), 7.64 – 7.57 (m, 1.70H), 7.56 – 7.52 (m, 0.30H), 7.49 – 7.37 (m, 4.25H), 7.35 – 7.26 (m, 0.60H), 7.24 – 7.15 (m, 0.45H), 5.63 (dd, *J* = 8.8, 4.9 Hz, 0.85H), 5.24 – 5.17 (m, 0.15H), 3.94 – 3.84 (m, 0.30H), 3.77 – 3.69 (m, 0.85H), 3.66 – 3.58 (m, 0.85H), 2.43 – 2.29 (m, 1.00H), 2.10 – 1.90 (m, 3.00H). ^{13}C NMR (101 MHz, $CDCl_3$) δ 196.74, 169.25, 139.65, 137.21, 136.10, 133.76, 130.10, 129.90, 129.48, 129.31, 128.93, 128.28, 128.18, 127.26, 126.40, 63.76, 61.18, 50.06, 46.72, 31.09, 29.32, 25.39, 22.40.

FT-IR (film): 3448, 1693, 1626, 1417, 1222, 1091, 702 cm^{-1}.

HRMS (ESI-MS) *m/z* $[M+H]^+$ calcd for $C_{18}H_{17}ClNO_2$: 314.0942, found: 314.0938.

$[\alpha]^{25}_D$ = +9.6 (*c* 1.0, CH_2Cl_2); 97% ee from (*S*, *R*)-**L2**.

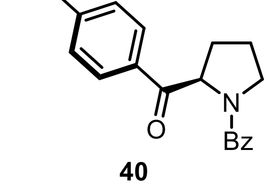

40

(*R*)-(1-Benzoylpyrrolidin-2-yl)(4-methoxyphenyl)methanone (**40**, from (*S*, *R*)-**L2**). The title compound was synthesized according to **GP-B** from 4-methoxybenzoic acid and *N*-Bz-pyrrolidine. The product was purified by preparative TLC on silica gel (1:4 Acetone/Petroleum ether). Yellow solid.

(*S*, *R*)-**L2**: 43.7 mg, 71% yield, 97% ee; (*R*, *S*)-**L2**: 43.7 mg, 71% yield, 96% ee.

HPLC analysis: The ee was determined via HPLC on a CHIRALCEL OD-3 column (20.0% 2-PrOH in hexanes, 1.0 mL/min); retention times for compound obtained using (*S*, *R*)-**L2**: 14.6 min (major), 24.2 min (minor).

^{1}H NMR (500 MHz, $CDCl_3$) (80:20 mixture of rotamers) δ 8.08 – 8.01 (m, 1.60H), 7.64 – 7.57 (m, 2.00H), 7.43 – 7.37 (m, 2.40H), 7.32 – 7.27 (m, 0.40H), 7.23 – 7.15 (m, 0.60H), 6.98 – 6.91 (m, 1.60H), 6.82 (d, *J* = 8.5 Hz, 0.40H), 5.68 (dd, *J* = 8.7, 4.9 Hz, 0.80H), 5.24 – 5.17 (m, 0.20H), 3.90 – 3.84 (m, 2.80H), 3.81 (s, 0.60H), 3.74 – 3.68 (m, 0.80H), 3.62 – 3.56 (m, 0.80H), 2.43 – 2.27 (m, 1.00H), 2.04 – 1.89 (m, 3.00H). ^{13}C NMR (126 MHz, $CDCl_3$) δ 196.07, 169.24, 163.64, 136.44, 130.83, 130.31, 129.93, 129.37, 128.19, 128.16, 128.13, 127.25, 126.46, 113.81, 63.65, 60.95, 55.43, 50.09, 46.74, 31.34, 29.56, 25.31, 22.38.

FT-IR (film): 3448, 1683, 1627, 1601, 1420, 1231, 1170, 701 cm^{-1}.

HRMS (ESI-MS) *m/z* $[M+H]^+$ calcd for $C_{19}H_{20}NO_3$: 310.1438, found: 310.1434.

$[\alpha]^{25}_{D}$ = +9.4 (*c* 1.0, CH_2Cl_2); 97% ee from (*S*, *R*)-**L2**.

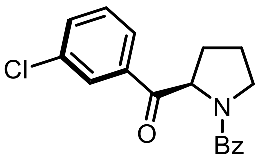

41

(*R*)-(1-Benzoylpyrrolidin-2-yl)(3-chlorophenyl)methanone (**41**, from (*S*, *R*)-**L2**). The title compound was synthesized according to **GP-B** from 3-chlorobenzoic acid and *N*-Bz-pyrrolidine. The product was purified by preparative TLC on silica gel (1:1 EtOAc/Petroleum ether). Yellow oil.

(*S*, *R*)-**L2**: 42.7 mg, 68% yield, 97% ee; (*R*, *S*)-**L2**: 42.7 mg, 68% yield, 97% ee.

HPLC analysis: The ee was determined via HPLC on a CHIRALCEL OD-3 column (20.0% 2-PrOH in hexanes, 1.0 mL/min); retention times for compound obtained using (*S*, *R*)-**L2**: 8.4 min (major), 13.8 min (minor).

^{1}H NMR (400 MHz, $CDCl_3$) (85:15 mixture of rotamers) δ 8.01 (t, *J* = 1.9 Hz, 0.85H), 7.93 (d, *J* = 7.7 Hz, 0.85H), 7.63 – 7.51 (m, 2.70H), 7.48 – 7.36 (m, 3.70H), 7.33 – 7.26 (m, 0.45H), 7.25 – 7.17 (m, 0.45H), 5.61 (dd, *J* = 8.8, 5.0 Hz, 0.85H), 5.20 (dd, *J* = 8.9, 2.4 Hz, 0.15H), 3.95 – 3.82 (m, 0.30H), 3.78 – 3.69 (m, 0.85H), 3.66 – 3.58 (m, 0.85H), 2.44 – 2.30 (m, 1.00H), 2.11 – 1.90 (m, 3.00H). ^{13}C NMR (101 MHz, $CDCl_3$) δ 196.77, 169.22, 137.03, 136.02, 134.91, 133.34, 133.10, 130.12, 129.95, 129.51, 128.52, 128.32, 128.17, 127.97, 127.25, 126.52, 126.38, 125.88, 63.85, 61.34, 50.04, 46.70, 31.05, 29.28, 25.39, 22.38.

FT-IR (film): 2924, 1698, 1627, 1417, 1206, 725 cm^{-1}.

HRMS (ESI-MS) *m/z* $[M+H]^+$ calcd for $C_{18}H_{17}ClNO_2$: 314.0942, found: 314.0938.

$[\alpha]^{25}_{D}$ = +15.1 (*c* 1.0, CH_2Cl_2); 97% ee from (*S*, *R*)-**L2**.

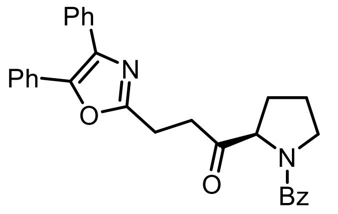

42

(*R*)-1-(1-Benzoylpyrrolidin-2-yl)-3-(4,5-diphenyloxazol-2-yl)propan-1-one (**42**, from (*S*, *R*)-**L1**). The title compound was synthesized according to **GP-B** from 3-(4,5-diphenyloxazol-2-yl)propanoic acid and *N*-Bz-pyrrolidine. The product was purified by preparative TLC on silica gel (1:4 Acetone/Petroleum ether). White solid.

(*S*, *R*)-**L1**: 49.0 mg, 54% yield, 99% ee; (*R*, *S*)-**L1**: 44.3 mg, 49% yield, 98% ee.

HPLC analysis: The ee was determined via HPLC on a CHIRALPAK AD-3 column (20.0% 2-PrOH in hexanes, 1.0 mL/min); retention times for compound obtained using (*S*, *R*)-**L1**: 9.8 min (major), 21.2 min (minor).

^{1}H NMR (400 MHz, $CDCl_3$) (85:15 mixture of rotamers) δ 7.70 – 7.49 (m, 5.85H), 7.47 – 7.26 (m, 9.15H), 4.83 (dd, *J* = 8.4, 6.0 Hz, 0.85H), 4.52 (dd, *J* = 8.8, 3.1 Hz, 0.15H), 3.85 – 3.75 (m, 0.30H), 3.71 – 3.60 (m, 0.85H), 3.61 – 3.51 (m, 0.85H), 3.36 – 3.10 (m, 3.40H), 3.09 – 2.80 (m, 0.30H), 2.36 – 2.12 (m, 1.30H), 2.14 – 2.03 (m, 0.85H), 2.06 – 1.94 (m, 1.15H), 1.92 – 1.81 (m, 1.00H). ^{13}C NMR (101 MHz, $CDCl_3$) major isomer δ 206.72, 169.70, 162.36, 145.34, 135.97, 134.93, 132.55, 130.23, 128.96, 128.55, 128.46, 128.34, 128.24, 127.92, 127.81, 127.23, 126.44, 64.91, 50.24, 36.30, 28.22, 25.38, 21.77.

FT-IR (film): 3448, 1724, 1627, 1415, 765, 696 cm^{-1}.

HRMS (ESI-MS) *m/z* $[M+H]^+$ calcd for $C_{29}H_{27}N_2O_3$: 451.2016, found: 451.2014.

$[\alpha]^{25}_D$ = +60.8 (*c* 1.0, CH_2Cl_2); 99% ee from (*S*, *R*)-**L1**.

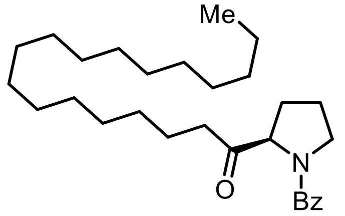

43

(*R*)-1-(4-(1-(Thiophene-2-carbonyl)pyrrolidin-2-yl)phenyl)ethan-1-one (**43** from (*S*, *R*)-**L1**). The title compound was synthesized according to **GP-B** from stearic acid and *N*-Bz-pyrrolidine. The product was purified by preparative TLC on silica gel (1:4 Acetone/Petroleum ether). White solid.

(*S*, *R*)-**L1**: 45.2 mg, 51% yield, 94% ee; (*R*, *S*)-**L1**: 45.4 mg, 51% yield, 92% ee.

HPLC analysis: The ee was determined via HPLC on a CHIRALPAK AD-3 column (5.0% 2-PrOH in hexanes, 0.8 mL/min); retention times for compound obtained using (*S*, *R*)-**L1**: 7.5 min (major), 14.3 min (minor).

^{1}H NMR (400 MHz, $CDCl_3$) (83:17 mixture of rotamers) δ 7.58 – 7.51 (m, 1.66H), 7.44 – 7.27 (m, 3.34H), 4.75 (dd, *J* = 8.6, 5.8 Hz, 0.83H), 4.43 (dd, *J* = 8.9, 2.7 Hz, 0.17H), 3.81 – 3.74 (m, 0.34H), 3.65 – 3.58 (m, 0.83H), 3.56 – 3.47 (m, 0.83H), 2.71 – 2.61 (m, 0.83H), 2.59 – 2.46 (m,

0.83H), 2.29 – 2.14 (m, 1.17H), 2.03 – 1.92 (m, 1.17H), 1.90 – 1.79 (m, 2.34H), 1.67 – 1.58 (m, 1.66H), 1.32 – 1.20 (m, 28.00H), 0.87 (t, *J* = 6.7 Hz, 3.00H). ^{13}C NMR (101 MHz, $CDCl_3$) δ 208.85, 169.52, 136.21, 130.10, 129.47, 128.32, 128.19, 127.23, 126.58, 67.01, 64.72, 50.19, 46.69, 40.13, 39.19, 31.88, 30.00, 29.65, 29.61, 29.58, 29.45, 29.42, 29.31, 29.25, 28.99, 28.33, 25.39, 23.30, 23.21, 22.64, 22.50, 14.05.

FT-IR (film): 2924, 2853, 1723, 1632, 1411, 700 cm^{-1}.

HRMS (ESI-MS) *m/z* [M+H]$^+$ calcd for $C_{29}H_{48}NO_2$: 442.3680, found: 442.3675.

$[\alpha]^{25}_D$ = +39.2 (*c* 1.0, CH_2Cl_2); 94% ee from (*S*, *R*)-**L1**.

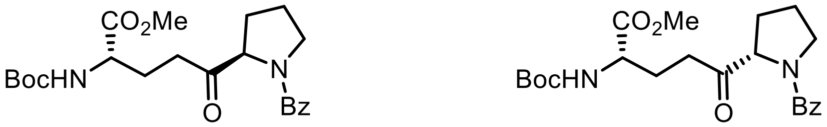

44 **45**

Methyl (*S*)-5-((*R*)-1-benzoylpyrrolidin-2-yl)-2-((*tert*-butoxycarbonyl)amino)-5-oxopentanoate (**44**, from (*S*, *R*)-**L1**). The title compound was synthesized according to **GP-B** from (*S*)-4-((tert-butoxycarbonyl)amino)-5-methoxy-5-oxopentanoic acid and *N*-Bz-pyrrolidine. The product was purified by preparative TLC on silica gel (2:7 Acetone/Petroleum ether). Yellow oil.

(*S*, *R*)-**L1**: 52.5 mg, 63% yield, 99:1 dr; (*R*, *S*)-**L1**: 57.1 mg, 68% yield, 2:98 dr.

HPLC analysis: The dr was determined via HPLC on a CHIRALPAK AD-3 column (20.0% 2-PrOH in hexanes, 1.0 mL/min); retention times for compound obtained using (*S*, *R*)-**L1**: 14.2 min (major), 25.7 min (minor).

NMR data for the product **44**:

^{1}H NMR (400 MHz, $CDCl_3$) (80:20 mixture of rotamers) δ 7.61 – 7.50 (m, 1.80H), 7.47 – 7.33 (m, 3.00H), 7.30 – 7.26 (m, 0.20H), 5.15 (d, *J* = 8.6 Hz, 0.80H), 4.91 (s, 0.20H), 4.72 (dd, *J* = 8.3, 5.6 Hz, 0.80H), 4.43 (d, *J* = 8.7 Hz, 0.20H), 4.34 – 4.22 (m, 0.80H), 4.14 – 4.05 (m, 0.20H), 3.83 – 3.69 (m, 3.20H), 3.67 – 3.59 (m, 1.00H), 3.58 – 3.49 (m, 1.00H), 2.88 – 2.79 (m, 0.80H), 2.73 – 2.55 (m, 1.00H), 2.30 – 2.12 (m, 2.00H), 2.07 – 1.82 (m, 4.00H), 1.42 (s, 9.00H). ^{13}C NMR (126 MHz, $CDCl_3$) major isomer δ 207.79, 172.77, 169.59, 155.45, 135.90, 130.21, 128.20, 127.19, 79.79, 64.84, 53.05, 52.27, 50.18, 35.91, 28.28, 28.23, 25.85, 25.37.

NMR data for the product **45**:

^{1}H NMR (400 MHz, $CDCl_3$) (79:21 mixture of rotamers) δ 7.64 – 7.49 (m, 1.79H), 7.47 – 7.30 (m, 3.00H), 7.28 (s, 0.21H), 5.12 (d, *J* = 8.4 Hz, 0.79H), 4.95 (s, 0.21H), 4.71 (dd, *J* = 8.3, 5.7 Hz, 0.79H), 4.40 (d, *J* = 9.0 Hz, 0.21H), 4.36 – 4.24 (m, 0.79H), 4.18 – 4.03 (m, 0.21H), 3.86 – 3.69 (m, 3.21H), 3.67 – 3.45 (m, 2.00H), 2.87 – 2.63 (m, 1.79H), 2.46 – 2.33 (m, 0.21H), 2.28 – 2.16 (m, 1.79H), 2.05 – 1.80 (m, 4.00H), 1.43 (s, 9.00H). ^{13}C NMR (126 MHz, $CDCl_3$) major isomer δ 207.55, 172.77, 169.47, 155.45, 135.90, 130.11, 128.13, 127.10, 79.71, 64.69, 52.69, 52.20, 50.10, 35.80, 28.17, 26.10, 25.31.

FT-IR (film): 3226, 2977, 1713, 1625, 1519, 1420, 1166, 1052, 702 cm^{-1}.

HRMS (ESI-MS) *m/z* [M+Na]$^+$ calcd for $C_{22}H_{30}N_2NaO_6$: 441.1996, found: 441.1994.

$[\alpha]^{25}_D$ = +49.1 (c 1.0, CH_2Cl_2); 99:1 dr from (*S*, *R*)-**L1**.

$[\alpha]^{25}_D$ = –37.9 (c 1.0, CH_2Cl_2); 2:98 dr from (*R*, *S*)-**L1**

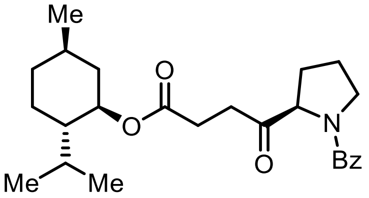

46

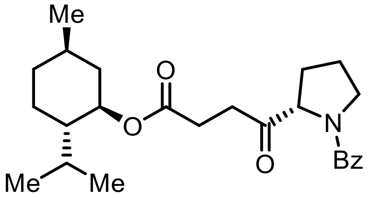

47

(1*R*,2*S*,5*R*)-2-Isopropyl-5-methylcyclohexyl 4-((*R*)-1-benzoylpyrrolidin-2-yl)-4-oxobutanoate (**46**, from (*S*, *R*)-**L1**). The title compound was synthesized according to **GP-B** from 4-(((1*R*,2*S*,5*R*)-2-isopropyl-5-methylcyclohexyl)oxy)-4-oxobutanoic acid and *N*-Bz-pyrrolidine. The product was purified by preparative TLC on silica gel (1:3 Acetone/Petroleum ether). Yellow oil.

(*S*, *R*)-**L1**: 58.7 mg, 71% yield, 98:2 dr; (*R*, *S*)-**L1**: 59.1 mg, 71% yield, 2:98 dr.

HPLC analysis: The dr was determined via HPLC on a CHIRALPAK AD-3 column (10.0% 2-PrOH in hexanes, 1.0 mL/min); retention times for compound obtained using (*S*, *R*)-**L1**: 11.2 min (major), 18.1 min (minor).

NMR data for the product **46**:

^{1}H NMR (500 MHz, $CDCl_3$) (90:10 mixture of rotamers) δ 7.58 – 7.50 (m, 1.80H), 7.43 – 7.35 (m, 2.80H), 7.34 – 7.29 (m, 0.40H), 4.78 – 4.70 (m, 0.90H), 4.68 – 4.56 (m, 1.00H), 4.48 – 4.38 (m, 0.10H), 3.78 – 3.74 (m, 0.20H), 3.67 – 3.58 (m, 1.00H), 3.57 – 3.46 (m, 0.90H), 3.07 – 2.98 (m, 0.20H), 2.98 – 2.83 (m, 1.80H), 2.72 – 2.63 (m, 0.90H), 2.60 – 2.51 (m, 0.90H), 2.49 – 2.42 (m, 0.10H), 2.27 – 2.19 (m, 1.00H), 2.03 – 1.92 (m, 3.00H), 1.89 – 1.80 (m, 2.00H), 1.69 – 1.61 (m, 2.00H), 1.48 – 1.40 (m, 1.00H), 1.38 – 1.31 (m, 1.00H), 1.02 – 0.91 (m, 2.00H), 0.90 – 0.80 (m, 7.00H), 0.78 – 0.69 (m, 3.00H). ^{13}C NMR (126 MHz, $CDCl_3$) major isomer δ 206.92, 172.34, 169.54, 135.97, 130.12, 128.14, 127.14, 74.34, 64.74, 50.20, 46.87, 40.74, 34.49, 34.13, 31.28, 28.14, 27.75, 26.15, 25.32, 23.38, 21.87, 20.62, 16.24.

NMR data for the product **47**:

^{1}H NMR (500 MHz, $CDCl_3$) (90:10 mixture of rotamers) δ 7.59 – 7.50 (m, 1.80H), 7.43 – 7.35 (m, 2.80H), 7.34 – 7.30 (m, 0.40H), 4.78 – 4.69 (m, 0.90H), 4.69 – 4.62 (m, 0.90H), 4.61 – 4.56 (m, 0.10H), 4.43 (dd, *J* = 9.0, 3.0 Hz, 0.10H), 3.79 – 3.74 (m, 0.20H), 3.67 – 3.58 (m, 1.00H), 3.57 – 3.45 (m, 0.90H), 3.03 (s, 0.20H), 2.90 (t, *J* = 6.5 Hz, 1.80H), 2.74 – 2.65 (m, 0.90H), 2.60 – 2.47 (m, 1.00H), 2.29 – 2.20 (m, 1.00H), 2.05 – 1.92 (m, 3.00H), 1.90 – 1.80 (m, 2.00H), 1.68 – 1.61 (m, 2.00H), 1.49 – 1.40 (m, 1.00H), 1.39 – 1.31 (m, 1.00H), 1.06 – 0.91 (m, 2.00H), 0.90 – 0.80 (m, 7.00H), 0.76 – 0.69 (m, 3.00H). ^{13}C NMR (126 MHz, $CDCl_3$) major isomer δ 206.86, 172.34, 169.56, 135.99, 130.14, 128.15, 127.17, 74.38, 64.81, 50.21, 46.91, 40.78, 34.45, 34.16, 31.29, 28.13, 27.80, 26.15, 25.29, 23.41, 21.90, 20.62, 16.26.

FT-IR (film): 3443, 2954, 1725, 1629, 1413, 1208, 701 cm^{-1}.

HRMS (ESI-MS) *m/z* $[M+H]^+$ calcd for $C_{25}H_{36}NO_4$: 414.2639, found: 414.2637.

$[\alpha]^{25}_{D}$ = +14.3 (*c* 1.0, CH_2Cl_2); 98:2 dr from (*S*, *R*)-**L1**.

$[\alpha]^{25}_{D}$ = –79.8 (*c* 1.0, CH_2Cl_2); 2:98 dr from (*R*, *S*)-**L1**.

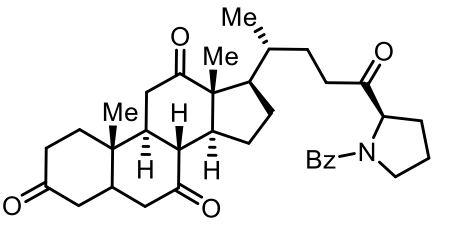

48

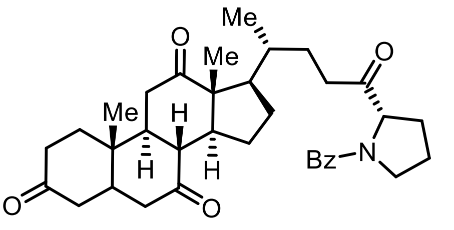

49

(8*R*,9*S*,10*S*,13*R*,14*S*,17*R*)-17-((*R*)-5-((*R*)-1-Benzoylpyrrolidin-2-yl)-5-oxopentan-2-yl)-10,13-dimethyldodecahydro-3*H*-cyclopenta[*a*]phenanthrene-3,7,12(2*H*,4*H*)-trione (**48**, from (*S*, *R*)-**L1**). The title compound was synthesized according to **GP-B** from (4*R*)-4-((8*R*,9*S*,10*S*,13*R*,14*S*,17*R*)-10,13-dimethyl-3,7,12-trioxohexadecahydro-1*H*-cyclopenta[*a*]phenanthren-17-yl)pentanoic acid and *N*-Bz-pyrrolidine. The product was purified by preparative TLC on silica gel (3:2 EtOAc/Petroleum ether). White solid.

(*S*, *R*)-**L1**: 70.4 mg, 63% yield, 98:2 dr; (*R*, *S*)-**L1**: 69.5 mg, 62% yield, 3:97 dr.

HPLC analysis: The dr was determined via HPLC on a CHIRALPAK AD-3 column (40.0% 2-PrOH in hexanes, 0.8 mL/min); retention times for compound obtained using (*S*, *R*)-**L1**: 18.9 min (major), 25.9 min (minor).

NMR data for the product **48**:

^{1}H NMR (500 MHz, $CDCl_3$) (83:17 mixture of rotamers) δ 7.61 – 7.47 (m, 1.66H), 7.45 – 7.26 (m, 3.34H), 4.86 – 4.63 (m, 0.83H), 4.47 – 4.42 (m, 0.17H), 3.84 – 3.70 (m, 0.34H), 3.65 – 3.57 (m, 0.83H), 3.57 – 3.48 (m, 0.83H), 2.94 – 2.78 (m, 3.00H), 2.77 – 2.48 (m, 1.66H), 2.36 – 2.17 (m, 7.00H), 2.17 – 2.06 (m, 2.34H), 2.05 – 1.79 (m, 9.00H), 1.64 – 1.55 (m, 1.00H), 1.44 – 1.27 (m, 5.83H), 1.27 – 1.22 (m, 1.17H), 1.05 (s, 2.49H), 1.00 (s, 0.51H), 0.83 (d, *J* = 6.6 Hz, 2.49H), 0.68 (d, *J* = 6.6 Hz, 0.51H). ^{13}C NMR (101 MHz, $CDCl_3$) major isomer δ 211.95, 209.02, 208.94, 208.57, 169.53, 136.18, 130.11, 128.20, 127.20, 64.80, 56.88, 51.72, 50.19, 48.95, 46.78, 45.67, 45.52, 44.92, 42.74, 38.59, 36.84, 36.42, 35.95, 35.23, 35.19, 28.66, 28.39, 27.45, 25.40, 25.10, 21.84, 18.80, 11.84.

NMR data for the product **49**:

^{1}H NMR (500 MHz, $CDCl_3$) (83:17 mixture of rotamers) δ 7.61 – 7.45 (m, 1.66H), 7.45 – 7.27 (m, 3.17H), 7.26 – 7.20 (m, 0.17H), 4.83 – 4.64 (m, 0.83H), 4.45 (d, *J* = 8.8 Hz, 0.17H), 3.84 – 3.71 (m, 0.34H), 3.68 – 3.56 (m, 0.83H), 3.56 – 3.45 (m, 0.83H), 2.93 – 2.81 (m, 2.66H), 2.66 – 2.61 (m, 1.34H), 2.40 – 2.06 (m, 9.00H), 2.06 – 1.51 (m, 10.83H), 1.43 – 1.16 (m, 7.17H), 1.06 (s, 2.49H), 1.03 (s, 0.51H), 0.85 (d, *J* = 5.6 Hz, 2.49H), 0.63 (d, *J* = 5.6 Hz, 0.51H). ^{13}C NMR (126 MHz, $CDCl_3$) major isomer δ 212.05, 209.03, 208.68, 169.48, 136.16, 130.12, 128.20, 127.23, 64.82, 56.89, 51.77, 50.19, 48.97, 46.80, 45.71, 45.55, 44.94, 42.75, 38.60, 37.10, 36.44, 35.97, 35.31, 35.23, 28.66, 28.50, 27.55, 25.43, 25.11, 21.86, 18.82, 11.86.

FT-IR (film): 2963, 2877, 1712, 1627, 1417, 732 cm^{-1}.

HRMS (ESI-MS) *m/z* $[M+H]^+$ calcd for $C_{35}H_{46}NO_5$: 560.3371, found: 560.3369.

$[\alpha]^{25}_D$ = +48.4 (*c* 1.0, CH_2Cl_2); 98:2 dr from (*S*, *R*)-**L1**.

$[\alpha]^{25}_D$ = –14.0 (*c* 1.0, CH_2Cl_2); 3:97 dr from (*R*, *S*)-**L1**.

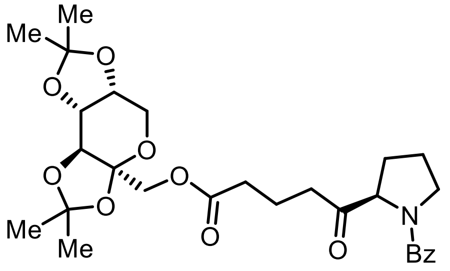

50

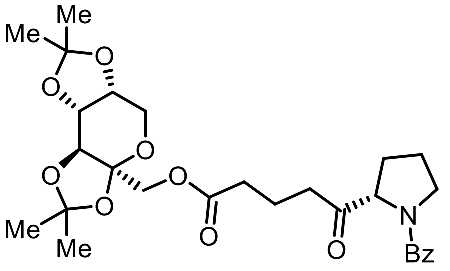

51

((3a*S*,5a*R*,8a*R*,8b*S*)-2,2,7,7-Tetramethyltetrahydro-3a*H*-bis([1,3]dioxolo)[4,5-*b*:4',5'-*d*]pyran-3a-yl)methyl 5-((*R*)-1-benzoylpyrrolidin-2-yl)-5-oxopentanoate (**50**, from (*S*, *R*)-**L1**). The title compound was synthesized according to **GP-B** from 5-oxo-5-(((3a*S*,5a*R*,8a*R*,8b*S*)-2,2,7,7-tetramethyltetrahydro-3a*H*-bis([1,3]dioxolo)[4,5-*b*:4',5'-*d*]pyran-3a-yl)methoxy)pentanoic acid and *N*-Bz-pyrrolidine. The product was purified by preparative TLC on silica gel (1:4 Acetone/Petroleum ether). Yellow oil.

(*S*, *R*)-**L1**: 76.2 mg, 72% yield, 97:3 dr; (*R*, *S*)-**L1**: 82.4 mg, 78% yield, 2:98 dr.

HPLC analysis: The dr was determined via HPLC on a CHIRALPAK AD-3 column (25.0% 2-PrOH in hexanes, 0.8 mL/min); retention times for compound obtained using (*S*, *R*)-**L1**: 12.1 min (major), 21.3 min (minor).

NMR data for the product **50**:

^{1}H NMR (500 MHz, $CDCl_3$) (80:20 mixture of rotamers) δ 7.54 – 7.49 (m, 1.60H), 7.44 – 7.26 (m, 3.40H), 4.76 – 4.63 (m, 0.80H), 4.56 (dd, *J* = 7.8, 2.7 Hz, 1.00H), 4.48 – 4.31 (m, 1.20H), 4.30 – 4.23 (m, 1.00H), 4.20 (dd, *J* = 8.0, 1.8 Hz, 1.00H), 4.04 – 3.93 (m, 1.00H), 3.91 – 3.81 (m, 1.00H), 3.80 – 3.64 (m, 1.40H), 3.62 – 3.56 (m, 0.80H), 3.54 – 3.48 (m, 0.80H), 2.80 – 2.71 (m, 0.80H), 2.68 – 2.58 (m, 0.80H), 2.41 (t, *J* = 7.2 Hz, 1.80H), 2.35 – 2.26 (m, 0.20H), 2.26 – 2.16 (m, 1.00H), 2.14 – 2.04 (m, 0.20H), 2.00 – 1.89 (m, 3.00H), 1.89 – 1.75 (m, 2.20H), 1.49 (s, 3.00H), 1.44 (s, 3.00H), 1.36 (s, 3.00H), 1.30 (s, 3.00H). ^{13}C NMR (126 MHz, $CDCl_3$) δ 207.91, 172.38, 171.94, 170.22, 169.46, 135.96, 130.11, 129.46, 128.29, 128.14, 127.15, 126.46, 109.00, 108.61, 101.42, 101.34, 70.68, 70.46, 70.39, 69.97, 66.81, 65.18, 65.00, 64.76, 61.15, 50.13, 46.68, 38.50, 37.79, 32.83, 32.45, 30.03, 28.25, 26.36, 25.79, 25.36, 25.15, 23.97, 22.49, 18.33, 18.10.

NMR data for the product **51**:

^{1}H NMR (500 MHz, $CDCl_3$) (80:20 mixture of rotamers) δ 7.56 – 7.49 (m, 1.60H), 7.43 – 7.24 (m, 3.40H), 4.75 – 4.63 (m, 0.80H), 4.56 (dd, *J* = 7.9, 2.7 Hz, 1.00H), 4.45 – 4.29 (m, 1.20H), 4.29 – 4.22 (m, 1.00H), 4.20 (dd, *J* = 8.0, 1.8 Hz, 1.00H), 4.04 – 3.93 (m, 1.00H), 3.90 – 3.82 (m, 1.00H), 3.80 – 3.64 (m, 1.40H), 3.63 – 3.56 (m, 0.80H), 3.54 – 3.47 (m, 0.80H), 2.81 – 2.69 (m, 0.80H), 2.68 – 2.60 (m, 0.80H), 2.47 – 2.37 (m, 1.60H), 2.36 – 2.27 (m, 0.20H), 2.25 – 2.15 (m, 1.00H), 2.14 – 2.04 (m, 0.20H), 2.03 – 1.90 (m, 3.00H), 1.90 – 1.78 (m, 2.20H), 1.50 (s, 3.00H), 1.44 (s, 3.00H), 1.37 (s, 3.00H), 1.30 (s, 3.00H). ^{13}C NMR (126 MHz, $CDCl_3$) δ 207.89, 172.37, 169.46, 135.97, 130.11, 129.49, 128.31, 128.14, 127.15, 126.44, 108.99, 108.63, 101.41, 70.68, 70.45, 70.39, 69.97, 66.82, 65.21, 64.98, 64.76, 61.14, 50.13, 46.67, 38.48, 37.79, 32.84, 32.50, 30.02, 28.26, 26.36, 25.78, 25.36, 25.17, 23.97, 22.48, 18.32, 18.11.

FT-IR (film): 3463, 2988, 1740, 1628, 1416, 1382, 1252, 1071, 731 cm^{-1}.

HRMS (ESI-MS) *m/z* $[M+H]^+$ calcd for $C_{28}H_{38}NO_9$: 532.2541, found: 532.2542.

$[\alpha]^{25}_{D}$ = +13.7 (*c* 1.0, CH_2Cl_2); 97:3 dr from (*S*, *R*)-**L1**.

$[\alpha]^{25}_{D}$ = –50.5 (*c* 1.0, CH_2Cl_2); 2:98 dr from (*R*, *S*)-**L1**.

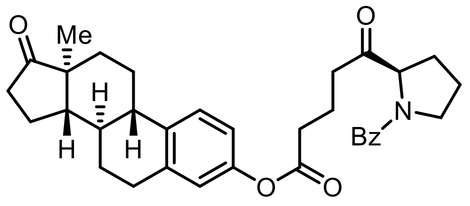

52

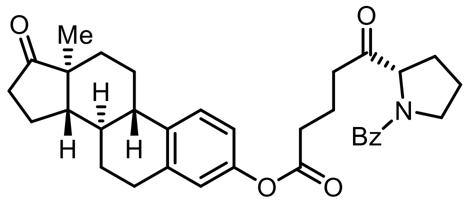

53

(8*R*,9*S*,13*S*,14*S*)-13-Methyl-17-oxo-7,8,9,11,12,13,14,15,16,17-decahydro-6*H*-cyclopenta[*a*]phenanthren-3-yl 5-((*R*)-1-benzoylpyrrolidin-2-yl)-5-oxopentanoate (**52**, from (*S*, *R*)-**L1**). The title compound was synthesized according to **GP-B** from 5-(((8*R*,9*S*,13*S*,14*S*)-13-methyl-17-oxo-7,8,9,11,12,13,14,15,16,17-decahydro-6*H*-cyclopenta[*a*]phenanthren-3-yl)oxy)-5-oxopentanoic acid and *N*-Bz-pyrrolidine. The product was purified by preparative TLC on silica gel (1:4 Acetone/Petroleum ether). White solid.

(*S*, *R*)-**L1**: 48.8 mg, 45% yield, 99:1 dr; (*R*, *S*)-**L1**: 50.7 mg, 47% yield, 2:98 dr.

HPLC analysis: The dr was determined via HPLC on a CHIRALPAK AD-3 column (40.0% 2-PrOH in hexanes, 0.8 mL/min); retention times for compound obtained using (*S*, *R*)-**L1**: 15.0 min (major), 30.5 min (minor).

NMR data for the product **52**:

^{1}H NMR (500 MHz, $CDCl_3$) (85:15 mixture of rotamers) δ 7.63 – 7.53 (m, 1.70H), 7.48 – 7.27 (m, 4.00H), 7.17 – 6.95 (m, 0.30H), 6.89 – 6.76 (m, 2.00H), 4.76 (dd, *J* = 8.5, 5.9 Hz, 0.85H), 4.54 – 4.41 (m, 0.15H), 3.88 – 3.75 (m, 0.30H), 3.68 – 3.61 (m, 0.85H), 3.60 – 3.51 (m, 0.85H), 3.00 – 2.82 (m, 2.85H), 2.79 – 2.69 (m, 1.00H), 2.67 – 2.59 (m, 1.70H), 2.56 – 2.46 (m, 1.15H), 2.44 – 2.36 (m, 1.15H), 2.33 – 2.22 (m, 2.15H), 2.20 – 2.04 (m, 3.85H), 2.04 – 1.94 (m, 3.15H), 1.93 – 1.86 (m, 2.15H), 1.67 – 1.41 (m, 5.85H), 0.97 – 0.86 (m, 3.00H). ^{13}C NMR (126 MHz, $CDCl_3$) major isomer δ 208.02, 172.03, 169.59, 148.49, 137.88, 137.21, 135.96, 130.19, 128.21, 127.20, 126.24, 121.50, 118.69, 64.86, 50.38, 50.19, 47.86, 44.07, 38.49, 37.94, 35.77, 33.17, 31.49, 29.29, 28.32, 26.26, 25.68, 25.43, 21.51, 18.54, 13.76.

NMR data for the product **53**:

^{1}H NMR (500 MHz, $CDCl_3$) (85:15 mixture of rotamers) δ 7.62 – 7.53 (m, 1.70H), 7.47 – 7.27 (m, 4.00H), 7.09 – 6.73 (m, 2.30H), 4.75 (dd, *J* = 8.5, 5.9 Hz, 0.85H), 4.54 – 4.42 (m, 0.15H), 3.85 – 3.75 (m, 0.30H), 3.69 – 3.61 (m, 0.85H), 3.60 – 3.51 (m, 0.85H), 2.96 – 2.82 (m, 2.85H), 2.79 – 2.70 (m, 1.00H), 2.67 – 2.60 (m, 1.70H), 2.55 – 2.47 (m, 1.15H), 2.44 – 2.37 (m, 1.15H), 2.32 – 2.23 (m, 2.15H), 2.20 – 2.05 (m, 3.85H), 2.04 – 1.95 (m, 3.15H), 1.92 – 1.85 (m, 2.15H), 1.67 – 1.42 (m, 5.85H), 0.96 – 0.79 (m, 3.00H). ^{13}C NMR (126 MHz, $CDCl_3$) major isomer δ 208.00, 172.02, 169.57, 148.48, 137.86, 137.20, 135.95, 130.17, 128.19, 127.18, 126.23, 121.49, 118.67, 64.85, 50.36, 50.18, 47.85, 44.06, 38.48, 37.93, 35.76, 33.15, 31.48, 29.28, 28.31, 26.25, 25.67, 25.42, 21.49, 18.53, 13.74.

FT-IR (film): 3450, 2922, 1735, 1626, 1413, 1150, 796 cm^{-1}.

HRMS (ESI-MS) *m/z* $[M+H]^+$ calcd for $C_{34}H_{40}NO_5$: 542.2901, found: 542.2900.

$[\alpha]^{25}_D$ = +109.7 (*c* 1.0, CH_2Cl_2); 99:1 dr from (*S*, *R*)-**L1**.

$[\alpha]^{25}_D$ = +43.9 (*c* 1.0, CH_2Cl_2); 2:98 dr from (*R*, *S*)-**L1**.

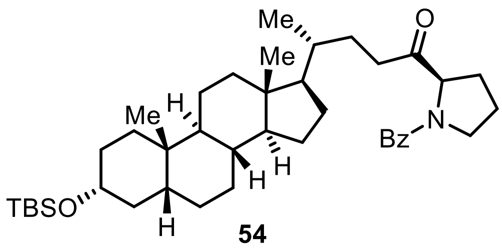
54

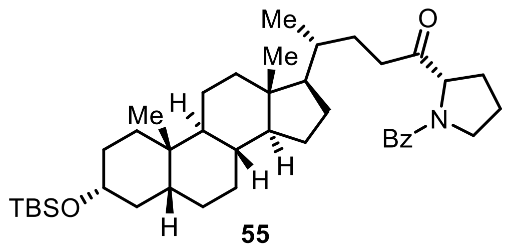
55

(4*R*)-1-((*R*)-1-Benzoylpyrrolidin-2-yl)-4-((3*R*,8*R*,9*S*,10*S*,13*R*,14*S*,17*R*)-3-((*tert*-butyldimethylsilyl)oxy)-10,13-dimethylhexadecahydro-1*H*-cyclopenta[*a*]phenanthren-17-yl)pentan-1-one (**54**, from (*S*, *R*)-**L1**). The title compound was synthesized according to **GP-B** from (4*R*)-4-((3*R*,8*R*,9*S*,10*S*,13*R*,14*S*,17*R*)-3-((*tert*-butyldimethylsilyl)oxy)-10,13-dimethylhexadecahydro-1*H*-cyclopenta[*a*]phenanthren-17-yl)pentanoic acid and *N*-Bz-pyrrolidine. The product was purified by preparative TLC on silica gel (1:5 EtOAc/Petroleum ether). White solid.

(*S*, *R*)-**L1**: 61.8 mg, 48% yield, 96:4 dr; (*R*, *S*)-**L1**: 60.4 mg, 47% yield, 3:97 dr.

HPLC analysis: The dr was determined via HPLC on a CHIRALPAK AD-3 column (5.0% 2-PrOH in hexanes, 1.0 mL/min); retention times for compound obtained using (*S*, *R*)-**L1**: 5.8 min (major), 7.6 min (minor).

NMR data for the product **54**:

^{1}H NMR (500 MHz, $CDCl_3$) (81:19 mixture of rotamers) δ 7.59 – 7.49 (m, 1.62H), 7.46 – 7.27 (m, 3.38H), 4.84 – 4.66 (m, 0.81H), 4.53 – 4.40 (m, 0.19H), 3.84 – 3.73 (m, 0.38H), 3.68 – 3.50 (m, 2.62H), 2.77 – 2.41 (m, 1.62H), 2.31 – 2.05 (m, 1.19H), 1.99 – 1.90 (m, 1.81H), 1.89 – 1.70 (m, 6.81H), 1.67 – 1.62 (m, 1.00H), 1.58 – 1.51 (m, 2.00H), 1.45 – 1.30 (m, 8.57H), 1.29 – 1.18 (m, 3.19H), 1.17 – 1.07 (m, 2.81H), 1.07 – 0.94 (m, 3.00H), 0.94 – 0.90 (m, 3.00H), 0.90 – 0.85 (m, 12.00H), 0.68 – 0.55 (m, 3.00H), 0.08 – 0.02 (m, 6.00H). ^{13}C NMR (126 MHz, $CDCl_3$) major isomer δ 209.26, 169.52, 136.24, 130.12, 128.22, 127.26, 72.83, 64.77, 56.37, 56.09, 50.22, 42.71, 42.30, 40.20, 40.14, 37.05, 36.92, 35.87, 35.58, 35.30, 34.58, 31.02, 29.27, 28.41, 28.20, 27.30, 26.39, 25.97, 25.42, 24.21, 23.38, 20.80, 18.46, 18.32, 12.03, –4.60.

NMR data for the product **55**:

^{1}H NMR (500 MHz, $CDCl_3$) (81:19 mixture of rotamers) δ 7.60 – 7.51 (m, 1.62H), 7.44 – 7.37 (m, 2.38H), 7.37 – 7.26 (m, 1.00H), 4.82 – 4.70 (m, 0.81H), 4.48 – 4.42 (m, 0.19H), 3.85 – 3.73 (m, 0.38H), 3.66 – 3.50 (m, 2.62H), 2.70 – 2.47 (m, 1.62H), 2.30 – 2.16 (m, 1.19H), 2.02 – 1.91 (m, 1.81H), 1.91 – 1.62 (m, 8.39H), 1.58 – 1.51 (m, 2.00H), 1.47 – 1.29 (m, 9.00H), 1.24 – 1.06 (m, 5.00H), 1.06 – 0.95 (m, 3.00H), 0.94 – 0.90 (m, 3.00H), 0.90 – 0.85 (m, 12.00H), 0.69 – 0.58 (m, 3.00H), 0.08 – 0.01 (m, 6.00H). ^{13}C NMR (126 MHz, $CDCl_3$) major isomer δ 209.38, 169.51, 136.22, 130.12, 128.21, 127.26, 72.82, 64.77, 56.39, 56.04, 50.21, 42.71, 42.29, 40.21, 40.15, 36.98, 36.92, 35.85, 35.57, 35.27, 34.57, 31.01, 29.28, 28.52, 28.20, 27.29, 26.38, 25.96, 25.45, 24.20, 23.37, 20.80, 18.48, 18.31, 12.02, -4.61.

FT-IR (film): 2928, 2861, 1724, 1631, 1412, 1079, 836, 774 cm^{-1}.

HRMS (ESI-MS) *m*/*z* $[M+H]^+$ calcd for $C_{41}H_{66}NO_3Si$: 648.4806, found: 648.4807.

$[\alpha]^{25}_D$ = +47.1 (*c* 1.0, CH_2Cl_2); 96:4 dr from (*S*, *R*)-**L1**.

$[\alpha]^{25}_D$ = –1.5 (*c* 1.0, CH_2Cl_2); 3:97 dr from (*R*, *S*)-**L1**.

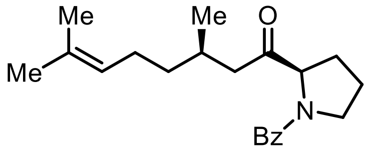

56

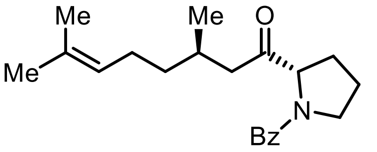

57

(*R*)-1-((*R*)-1-Benzoylpyrrolidin-2-yl)-3,7-dimethyloct-6-en-1-one (**56**, from (*S*, *R*)-**L1**). The title compound was synthesized according to **GP-B** from (*R*)-3,7-dimethyloct-6-enoic acid and *N*-Bz-pyrrolidine. The product was purified by preparative TLC on silica gel (1:6 Acetone/Petroleum ether). Yellow oil.

(*S*, *R*)-**L1**: 35.0 mg, 53% yield, 97:3 dr; (*R*, *S*)-**L1**: 33.8 mg, 52% yield, 3:97 dr.

HPLC analysis: The dr was determined via HPLC on a CHIRALPAK AD-3 column (15% 2-PrOH in hexanes, 1.0 mL/min); retention times for compound obtained using (*S*, *R*)-**L1**: 5.8 min (major), 9.9 min (minor).

NMR data for the product **56**:

^{1}H NMR (500 MHz, $CDCl_3$) (84:16 mixture of rotamers) δ 7.57 – 7.52 (m, 1.68H), 7.42 – 7.36 (m, 2.68H), 7.33 – 7.30 (m, 0.64H), 5.20 – 5.05 (m, 0.84H), 5.00 (t, *J* = 7.4 Hz, 0.16H), 4.72 (dd, *J* = 8.5, 6.1 Hz, 0.84H), 4.41 (dd, *J* = 8.9, 2.9 Hz, 0.16H), 3.76 (t, *J* = 6.8 Hz, 0.32H), 3.66 – 3.58 (m, 1.00H), 3.55 – 3.49 (m, 0.84H), 3.04 (s, 0.16H), 2.59 – 2.45 (m, 1.68H), 2.25 – 2.17 (m, 1.00H), 2.12 – 2.06 (m, 1.00H), 2.00 – 1.92 (m, 2.52H), 1.88 – 1.80 (m, 2.48H), 1.71 – 1.64 (m, 3.16H), 1.61 – 1.53 (m, 2.84H), 1.41 – 1.31 (m, 1.00H), 1.23 – 1.14 (m, 1.00H), 0.95 (d, *J* = 6.6 Hz, 2.52H), 0.59 (d, *J* = 6.6 Hz, 0.48H). ^{13}C NMR (126 MHz, $CDCl_3$) major isomer δ 208.26, 169.51, 136.20, 131.28, 130.08, 128.17, 127.20, 124.41, 65.12, 50.17, 47.45, 36.96, 28.19, 25.62, 25.46, 25.39, 19.67, 17.58.

NMR data for the product **57**:

^{1}H NMR (500 MHz, $CDCl_3$) (82:18 mixture of rotamers) δ 7.61 – 7.51 (m, 1.82H), 7.42 – 7.36 (m, 2.46H), 7.34 – 7.28 (m, 0.72H), 5.09 (t, *J* = 7.2 Hz, 0.82H), 4.99 (t, *J* = 7.2 Hz, 0.18H), 4.81 – 4.71 (m, 0.82H), 4.42 (d, *J* = 8.1 Hz, 0.18H), 3.82 – 3.72 (m, 0.36H), 3.68 – 3.57 (m, 1.00H), 3.56 – 3.48 (m, 0.82H), 3.04 (s, 0.18H), 2.72 – 2.59 (m, 0.82H), 2.41 – 2.32 (m, 0.82H), 2.28 – 2.17 (m, 1.00H), 2.16 – 2.07 (m, 1.00H), 2.02 – 1.92 (m, 2.54H), 1.88 – 1.80 (m, 2.46H), 1.73 – 1.63 (m, 3.18H), 1.63 – 1.50 (m, 2.82H), 1.42 – 1.31 (m, 1.00H), 1.25 – 1.18 (m, 1.00H), 0.92 (d, *J* = 6.6 Hz, 2.46H), 0.68 (d, *J* = 6.6 Hz, 0.54H). ^{13}C NMR (126 MHz, $CDCl_3$) major isomer δ 208.06, 169.48, 136.23, 131.30, 130.06, 128.17, 127.19, 124.40, 64.84, 50.18, 47.59, 36.96, 28.16, 28.03, 25.63, 25.48, 25.28, 19.68, 17.59.

FT-IR (film): 2962, 2925, 1721, 1630, 1412, 1056, 701 cm^{-1}.

HRMS (ESI-MS) *m*/*z* $[M+H]^+$ calcd for $C_{21}H_{30}NO_2$: 328.2271, found: 328.2268.

$[\alpha]^{25}_{D}$ = +65.8 (*c* 1.0, CH_2Cl_2); 97:3 dr from (*S*, *R*)-**L1**.

$[\alpha]^{25}_{D}$ = –40.8 (*c* 1.0, CH_2Cl_2); 3:97 dr from (*R*, *S*)-**L1**.

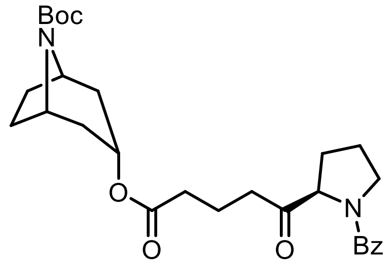

58

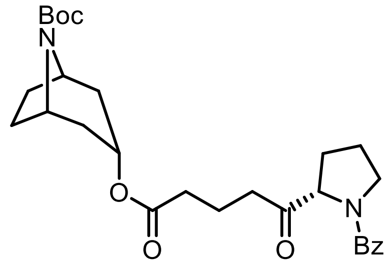

59

***tert*-Butyl (1*R*,3*r*,5*S*)-3-((5-((*R*)-1-benzoylpyrrolidin-2-yl)-5-oxopentanoyl)oxy)-8-azabicyclo[3.2.1]octane-8-carboxylate** (**58**, from (*S*, *R*)-**L1**). The title compound was synthesized according to **GP-B** from 5-(((1*R*,3*r*,5*S*)-8-(tert-butoxycarbonyl)-8-azabicyclo[3.2.1]octan-3-yl)oxy)-5-oxopentanoic acid and *N*-Bz-pyrrolidine. The product was purified by preparative TLC on silica gel (1:4 Acetone/Petroleum ether). Yellow oil.

(*S*, *R*)-**L1**: 65.5 mg, 66% yield, 99:1 dr; (*R*, *S*)-**L1**: 61.8 mg, 62% yield, 1:99 dr.

HPLC analysis: The dr was determined via HPLC on a CHIRALCEL OD-3 column (25.0% 2-PrOH in hexanes, 0.8 mL/min); retention times for compound obtained using (*S*, *R*)-**L1**: 13.4 min (major), 15.1 min (minor).

NMR data for the product **58**:

^{1}H NMR (500 MHz, $CDCl_3$) (87:13 mixture of rotamers) δ 7.56 – 7.50 (m, 1.74H), 7.44 – 7.34 (m, 2.74H), 7.33 – 7.27 (m, 0.52H), 5.09 – 4.99 (m, 1.00H), 4.69 (dd, *J* = 8.5, 6.1 Hz, 0.87H), 4.43 (dd, *J* = 9.3, 2.7 Hz, 0.13H), 4.17 (brs, 1.00H), 4.10 (brs, 1.00H), 3.79 – 3.72 (m, 0.26H), 3.63 – 3.57 (m, 0.87H), 3.55 – 3.48 (m, 0.87H), 2.79 – 2.71 (m, 0.87H), 2.66 – 2.58 (m, 0.87H), 2.41 – 2.28 (m, 2.00H), 2.26 – 2.16 (m, 1.26H), 2.14 – 2.08 (m, 1.00H), 2.06 – 2.02 (m, 1.00H), 1.98 – 1.88 (m, 7.00H), 1.86 – 1.79 (m, 2.00H), 1.72 – 1.63 (m, 2.00H), 1.42 (s, 9.00H). ^{13}C NMR (126 MHz, $CDCl_3$) major isomer δ 208.02, 172.29, 169.51, 153.22, 135.88, 130.19, 128.16, 127.16, 79.19, 67.76, 64.80, 52.56, 51.82, 50.16, 38.57, 35.72, 35.02, 33.65, 28.38, 28.31, 28.21, 27.53, 25.42, 18.40.

NMR data for the product **59**:

^{1}H NMR (500 MHz, $CDCl_3$) (88:12 mixture of rotamers) δ 7.55 – 7.48 (m, 1.76H), 7.42 – 7.26 (m, 3.24H), 5.08 – 4.98 (m, 1.00H), 4.68 (dd, *J* = 8.4, 6.0 Hz, 0.88H), 4.42 (d, *J* = 8.6 Hz, 0.12H), 4.16 (brs, 1.00H), 4.10 (brs, 1.00H), 3.78 – 3.71 (m, 0.24H), 3.62 – 3.56 (m, 0.88H), 3.55 – 3.47 (m, 0.88H), 2.79 – 2.69 (m, 0.88H), 2.67 – 2.57 (m, 0.88H), 2.39 – 2.27 (m, 2.00H), 2.26 – 2.16 (m, 1.24H), 2.11 – 2.02 (m, 2.00H), 1.98 – 1.87 (m, 7.00H), 1.86 – 1.79 (m, 2.00H), 1.67 (t, *J* = 13.5 Hz, 2.00H), 1.42 (s, 9.00H). ^{13}C NMR (126 MHz, $CDCl_3$) major isomer δ 208.00, 172.26, 169.48, 153.19, 135.86, 130.16, 128.14, 127.13, 79.16, 67.73, 64.78, 52.54, 51.74, 50.14, 38.53, 35.70, 35.02, 33.62, 28.36, 28.29, 28.16, 27.55, 25.39, 18.37.

FT-IR (film): 3448, 1629, 1408, 1162, 1032 cm^{-1}.

HRMS (ESI-MS) *m*/*z* $[M+H]^+$ calcd for $C_{28}H_{39}N_2O_6$: 499.2803, found: 499.2801.

$[\alpha]^{25}_{D}$ = +35.4 (*c* 1.0, CH_2Cl_2); 99:1 dr from (*S*, *R*)-**L1**.

$[\alpha]^{25}_{D}$ = –37.5 (*c* 1.0, CH_2Cl_2); 1:99 dr from (*R*, *S*)-**L1**.

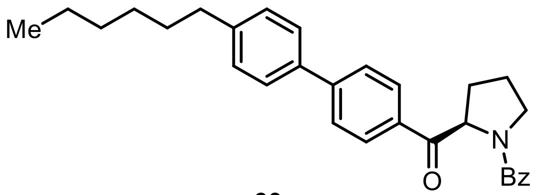

60

(*R*)-(1-Benzoylpyrrolidin-2-yl)(4'-hexyl-[1,1'-biphenyl]-4-yl)methanone (**60**, from (*S*, *R*)-**L2**). The title compound was synthesized according to **GP-B** from 4'-hexyl-[1,1'-biphenyl]-4-carboxylic acid and *N*-Bz-pyrrolidine. The product was purified by preparative TLC on silica gel (2:3 EtOAc/Petroleum ether). White solid.

(*S*, *R*)-**L2**: 49.0 mg, 56% yield, 95% ee; (*R*, *S*)-**L2**: 51.4 mg, 58% yield, 94% ee.

HPLC analysis: The ee was determined via HPLC on a CHIRALCEL OD-3 column (15.0% 2-PrOH in hexanes, 1.0 mL/min); retention times for compound obtained using (*S*, *R*)-**L2**: 11.7 min (major), 23.3 min (minor).

^{1}H NMR (500 MHz, $CDCl_3$) (83:17 mixture of rotamers) δ 8.13 (d, *J* = 8.1 Hz, 1.66H), 7.74 – 7.61 (m, 3.83H), 7.60 – 7.53 (m, 2.00H), 7.50 (d, *J* = 7.9 Hz, 0.51H), 7.45 – 7.39 (m, 2.49H), 7.36 – 7.33 (m, 0.34H), 7.29 (d, *J* = 8.0 Hz, 1.66H), 7.24 – 7.19 (m, 0.51H), 5.75 (dd, *J* = 8.8, 4.9 Hz, 0.83H), 5.31 (d, *J* = 8.8 Hz, 0.17H), 3.96 – 3.87 (m, 0.34H), 3.79 – 3.71 (m, 0.83H), 3.67 – 3.59 (m, 0.83H), 2.71 – 2.61 (m, 2.00H), 2.46 – 2.30 (m, 1.00H), 2.08 – 1.92 (m, 3.00H), 1.71 – 1.62 (m, 2.00H), 1.42 – 1.29 (m, 6.00H), 0.95 – 0.87 (m, 3.00H). ^{13}C NMR (126 MHz, $CDCl_3$) δ 197.76, 197.23, 170.35, 169.20, 146.04, 145.89, 143.47, 143.22, 137.32, 137.10, 136.66, 136.37, 133.72, 132.54, 129.96, 129.41, 129.07, 128.98, 128.94, 128.55, 128.25, 128.13, 127.28, 127.04, 126.99, 126.94, 126.47, 63.94, 61.26, 50.07, 46.73, 35.57, 31.64, 31.30, 31.27, 31.22, 29.46, 28.93, 25.35, 22.52, 22.37, 14.01.

FT-IR (film): 3448, 2927, 1690, 1628, 1602, 1418, 1227, 1003, 700 cm^{-1}.

HRMS (ESI-MS) *m/z* $[M+H]^+$ calcd for $C_{30}H_{34}NO_2$: 440.2584, found: 440.2582.

$[\alpha]^{25}_{D}$ = –14.4 (*c* 1.0, CH_2Cl_2); 95% ee from (*S*, *R*)-**L2**.

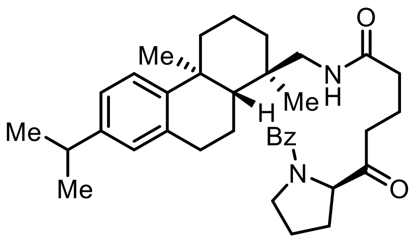

61

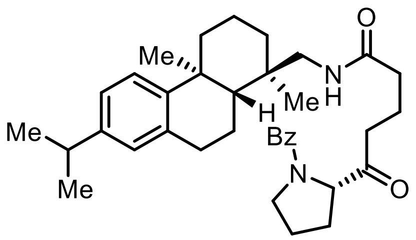

62

5-((*R*)-1-Benzoylpyrrolidin-2-yl)-*N*-(((1*R*,4a*S*,10a*R*)-7-isopropyl-1,4a-dimethyl-1,2,3,4,4a,9,10,10a-octahydrophenanthren-1-yl)methyl)-5-oxopentanamide (**61**, from (*S*, *R*)-**L1**). The title compound was synthesized according to **GP-B** from 5-((((1*R*,4a*S*,10a*R*)-7-isopropyl-1,4a-dimethyl-1,2,3,4,4a,9,10,10a-octahydrophenanthren-1-yl)methyl)amino)-5-oxopentanoic acid and *N*-Bz-pyrrolidine. The product was purified by preparative TLC on silica gel (1:3 Acetone/Petroleum ether). Yellow oil.

(*S*, *R*)-**L1**: 40.5 mg, 36% yield, 99:1 dr; (*R*, *S*)-**L1**: 39.1 mg, 35% yield, 1:99 dr.

HPLC analysis: The dr was determined via HPLC on a CHIRALCEL OD-3 column (15.0% 2-PrOH in hexanes, 1.0 mL/min); retention times for compound obtained using (*S*, *R*)-**L1**: 7.4 min (major), 10.4 min (minor).

NMR data for the product **61**:

^{1}H NMR (500 MHz, $CDCl_3$) (83:17 mixture of rotamers) δ 7.61 – 7.34 (m, 4.32H), 7.33 – 7.26 (m, 1.00H), 7.19 – 7.11 (m, 1.00H), 7.01 – 6.94 (m, 1.00H), 6.90 – 6.82 (m, 1.00H), 6.68 – 6.59 (m, 0.68H), 4.39 (t, *J* = 7.2 Hz, 0.83H), 3.69 – 3.61 (m, 0.17H), 3.60 – 3.51 (m, 0.83H), 3.48 – 3.42 (m, 1.34H), 2.88 – 2.76 (m, 3.34H), 2.75 – 2.59 (m, 0.83H), 2.39 – 2.14 (m, 3.83H), 2.13 – 1.80 (m, 6.00H), 1.79 – 1.48 (m, 5.83H), 1.46 – 1.15 (m, 13.00H), 0.93 – 0.85 (m, 3.00H). ^{13}C NMR (126 MHz, $CDCl_3$) major isomer δ 208.57, 173.22, 169.14, 147.42, 145.43, 135.84, 134.91, 130.22, 128.26, 127.07, 126.83, 123.97, 123.63, 65.45, 50.19, 49.68, 44.37, 38.22, 38.11, 37.27, 37.14, 35.79, 35.15, 33.37, 29.95, 28.19, 25.54, 25.18, 23.94, 20.09, 18.94, 18.77, 18.66.

NMR data for the product **62**:

^{1}H NMR (500 MHz, $CDCl_3$) (81:19 mixture of rotamers) δ 7.60 – 7.47 (m, 1.81H), 7.47 – 7.26 (m, 3.19H), 7.18 – 7.12 (m, 1.00H), 7.07 – 6.79 (m, 2.19H), 6.60 (t, *J* = 6.5 Hz, 0.81H), 4.58 (dd, *J* = 8.6, 5.8 Hz, 0.81H), 3.81 – 3.72 (m, 0.19H), 3.67 – 3.51 (m, 1.00H), 3.46 – 3.38 (m, 0.81H), 3.24 – 3.05 (m, 1.81H), 2.90 – 2.74 (m, 3.57H), 2.50 – 2.43 (m, 0.81H), 2.31 – 2.15 (m, 3.00H), 2.11 – 2.03 (m, 1.00H), 2.03 – 1.95 (m, 1.62H), 1.95 – 1.83 (m, 3.00H), 1.76 – 1.66 (m, 3.38H), 1.65 – 1.59 (m, 1.00H), 1.49 – 1.37 (m, 2.00H), 1.37 – 1.14 (m, 12.00H), 0.92 (s, 3.00H). ^{13}C NMR (126 MHz, $CDCl_3$) major isomer δ 208.69, 173.35, 169.33, 147.21, 145.34, 135.74, 134.84, 130.31, 128.24, 127.17, 126.82, 124.05, 123.67, 65.38, 50.15, 49.55, 44.88, 38.37, 38.12, 37.55, 37.38, 35.97, 35.24, 33.35, 29.99, 28.10, 25.32, 25.30, 23.98, 23.85, 20.35, 18.83, 18.58.

FT-IR (film): 3397, 2927, 1621, 1423, 1265, 734 cm^{-1}.

HRMS (ESI-MS) *m*/*z* $[M+H]^+$ calcd for $C_{36}H_{49}N_2O_3$: 557.3738, found: 557.3737.

$[\alpha]^{25}_{D}$ = +13.5 (*c* 1.0, CH_2Cl_2); 99:1 dr from (*S*, *R*)-**L1**.

$[\alpha]^{25}_{D}$ = +8.1 (*c* 1.0, CH_2Cl_2); 1:99 dr from (*R*, *S*)-**L1**.

1.5 Synthetic Utility

Concurrent rapid access to Taxol derivatives:

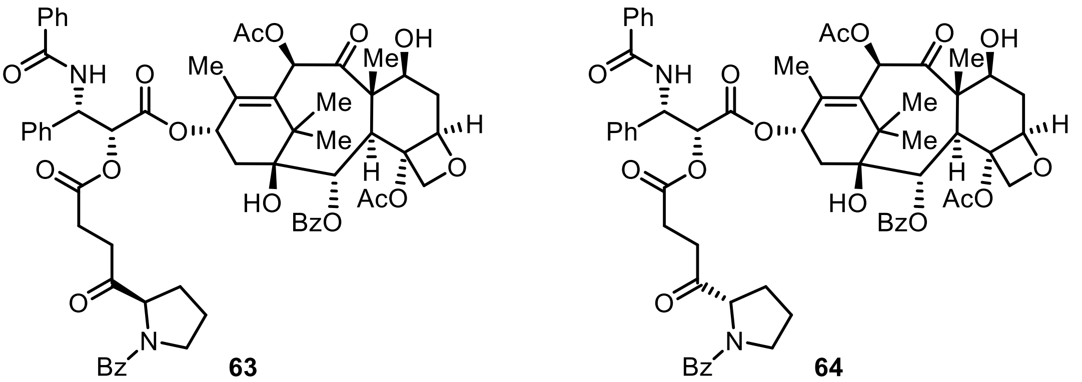

(2a*R*,4*S*,4a*S*,6*R*,9*S*,11*S*,12*S*,12b*S*)-9-(((2*R*,3*S*)-3-Benzamido-2-((4-((*R*)-1-benzoylpyrrolidin-2-yl)-4-oxobutanoyl)oxy)-3-phenylpropanoyl)oxy)-12-(benzoyloxy)-4,11-dihydroxy-4a,8,13,13-tetramethyl-5-oxo-3,4,4a,5,6,9,10,11,12,12a-decahydro-1*H*-7,11-methanocyclodeca[3,4]benzo[1,2-*b*]oxete-6,12b(2a*H*)-diyl diacetate (**63**, from (*S*, *R*)-**L1**). The title compound was synthesized according to **GP-B** from 4-(((1*S*,2*R*)-1-benzamido-3-(((2a*R*,4*S*,4a*S*,6*R*,9*S*,11*S*,12*S*,12b*S*)-6,12b-diacetoxy-12-(benzoyloxy)-4,11-dihydroxy-4a,8,13,13-tetramethyl-5-oxo-2a,3,4,4a,5,6,9,10,11,12,12a,12b-dodecahydro-1*H*-7,11-methanocyclodeca[3,4]benzo[1,2-*b*]oxet-9-yl)oxy)-3-oxo-1-phenylpropan-2-yl)oxy)-4-oxobutanoic acid and *N*-Bz-pyrrolidine. The product was purified by flash chromatography on silica gel (1:1 Acetone/Petroleum ether). White solid.

(*S*, *R*)-**L1**: 132.5 mg, 60% yield, 98:2 dr; (*R*, *S*)-**L1**: 128.4 mg, 58% yield, 2:98 dr.

HPLC analysis: The dr was determined via HPLC on a CHIRALPAK IF column (50.0% 2-PrOH in hexanes, 0.8 mL/min); retention times for compound obtained using (*S*, *R*)-**L1**: 26.7 min (major), 62.4 min (minor).

NMR data for the product **63**:

^{1}H NMR (400 MHz, $CDCl_3$) (89:11 mixture of rotamers) δ 8.15 – 8.07 (m, 2.00H), 8.02 (d, *J* = 9.0 Hz, 0.89H), 7.81 – 7.69 (m, 2.00H), 7.62 – 7.57 (m, 1.00H), 7.53 – 7.47 (m, 2.11H), 7.45 – 7.26 (m, 12.00H), 7.24 – 7.18 (m, 1.00H), 6.28 (s, 1.00H), 6.24 – 6.14 (m, 1.00H), 5.94 (dd, *J* = 9.1, 3.9 Hz, 1.00H), 5.65 (d, *J* = 7.1 Hz, 1.00H), 5.43 (d, *J* = 3.9 Hz, 1.00H), 4.93 (dd, *J* = 9.6, 2.3 Hz, 1.00H), 4.57 (dd, *J* = 8.2, 6.3 Hz, 1.00H), 4.46 – 4.35 (m, 1.00H), 4.32 – 4.14 (m, 2.00H), 3.81 – 3.73 (m, 1.11H), 3.64 – 3.48 (m, 1.89H), 3.14 – 3.03 (m, 0.89H), 2.89 – 2.66 (m, 2.89H), 2.60 – 2.47 (m, 2.22H), 2.42 – 2.24 (m, 4.00H), 2.22 – 2.14 (m, 4.00H), 2.09 – 2.05 (m, 1.00H), 2.01 – 1.89 (m, 5.00H), 1.89 – 1.80 (m, 3.00H), 1.66 (s, 3.00H), 1.23 (s, 0.33H), 1.20 (s, 2.67H), 1.11 (s, 3.00H). ^{13}C NMR (101 MHz, $CDCl_3$) major isomer δ 206.20, 203.81, 171.41, 171.10, 169.76, 169.47, 168.25, 167.47, 166.81, 142.99, 137.16, 135.44, 134.06, 133.51, 132.51, 131.45, 130.37, 130.14, 129.24, 128.72, 128.58, 128.23, 128.20, 128.14, 127.48, 127.15, 126.99, 84.37, 80.87, 78.93, 76.33, 75.59, 75.07, 74.23, 71.99, 71.56, 64.77, 58.40, 52.95, 50.24, 45.52, 43.06, 35.47, 34.47, 28.33, 27.46, 26.69, 25.54, 22.43, 22.03, 20.73, 14.72, 9.52.

NMR data for the product **64**:

^{1}H NMR (400 MHz, $CDCl_3$) (91:9 mixture of rotamers) δ 8.19 – 8.08 (m, 2.00H), 7.79 – 7.70 (m, 2.00H), 7.68 – 7.55 (m, 2.00H), 7.53 – 7.43 (m, 4.00H), 7.43 – 7.27 (m, 9.18H), 7.24 – 7.16 (m, 1.82H), 6.29 (s, 1.00H), 6.24 – 6.13 (m, 1.00H), 5.97 (dd, *J* = 9.0, 4.0 Hz, 1.00H), 5.66 (d, *J* = 7.1 Hz, 1.00H), 5.51 (d, *J* = 3.9 Hz, 0.91H), 5.47 – 5.41 (m, 0.09H), 4.95 (dd, *J* = 9.6, 2.3 Hz, 1.00H), 4.61 (dd, *J* = 8.3, 5.5 Hz, 0.91H), 4.52 – 4.36 (m, 1.09H), 4.33 – 4.14 (m, 2.00H), 3.80 (d, *J* = 7.0 Hz, 1.00H), 3.76 – 3.69 (m, 0.18H), 3.61 – 3.48 (m, 1.82H), 3.02 – 2.63 (m, 3.82H), 2.61 – 2.59 (m, 0.18H), 2.59 – 2.48 (m, 2.09H), 2.42 (s, 2.82H), 2.36 – 2.27 (m, 1.09H), 2.26 – 2.16 (m, 4.00H), 2.11 – 2.04 (m, 1.00H), 1.95 – 1.77 (m, 8.00H), 1.66 (s, 3.00H), 1.23 (s, 0.54H), 1.20 (s, 2.73H), 1.12 (s, 2.73H). ^{13}C NMR (101 MHz, $CDCl_3$) major isomer δ 206.59, 203.80, 171.63, 171.11, 169.86, 169.84, 167.99, 167.36, 166.91, 142.89, 137.15, 135.70, 133.64, 133.56, 132.61, 131.55, 130.29, 130.17, 129.22, 128.84, 128.64, 128.30, 128.23, 127.31, 127.09, 126.78, 84.39, 80.94, 78.99, 76.36, 75.58, 75.08, 74.17, 72.02, 71.58, 64.93, 58.44, 52.93, 50.25, 45.55, 43.09, 35.49, 34.08, 28.26, 27.66, 26.68, 25.31, 22.57, 22.04, 20.74, 14.70, 9.54.

FT-IR (film): 3440, 1722, 1618, 1243, 1071, 702 cm^{-1}.

HRMS (ESI-MS) *m/z* $[M+Na]^+$ calcd for $C_{62}H_{66}N_2NaO_{17}$: 1133.4254, found: 1133.4255.

$[\alpha]^{25}_D$ = –39.2 (*c* 1.0, CH_2Cl_2); 98:2 dr from (*S*, *R*)-**L1**.

$[\alpha]^{25}_D$ = –66.2 (*c* 1.0, CH_2Cl_2); 2:98 dr from (*R*, *S*)-**L1**.

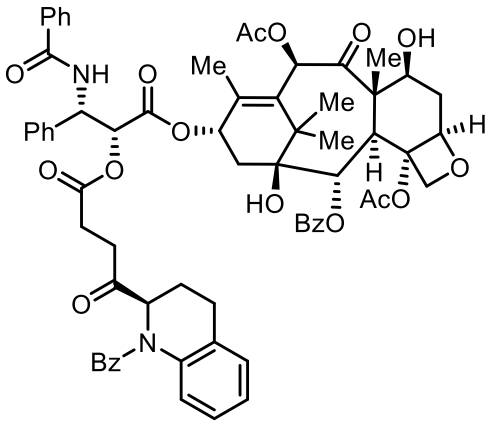

65

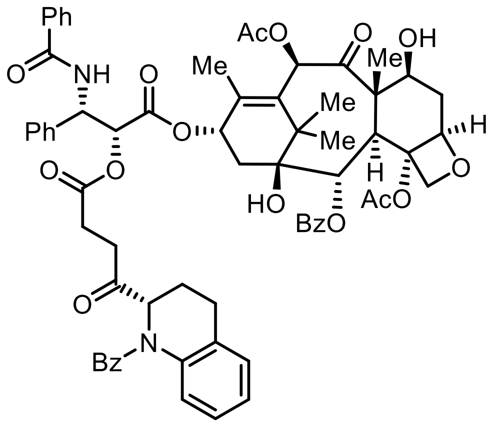

66

(2a*R*,4*S*,4a*S*,6*R*,9*S*,11*S*,12*S*,12b*S*)-9-(((2*R*,3*S*)-3-Benzamido-2-((4-((*R*)-1-benzoyl-1,2,3,4-tetrahydroquinolin-2-yl)-4-oxobutanoyl)oxy)-3-phenylpropanoyl)oxy)-12-(benzoyloxy)-4,11-dihydroxy-4a,8,13,13-tetramethyl-5-oxo-3,4,4a,5,6,9,10,11,12,12a-decahydro-1*H*-7,11-methanocyclodeca[3,4]benzo[1,2-*b*]oxete-6,12b(2a*H*)-diyl diacetate (65, from (*S*, *R*)-**L1**). The title compound was synthesized according to **GP-B** from 4-(((1*S*,2*R*)-1-benzamido-3-(((2a*R*,4*S*,4a*S*,6*R*,9*S*,11*S*,12*S*,12b*S*)-6,12b-diacetoxy-12-(benzoyloxy)-4,11-dihydroxy-4a,8,13,13-tetramethyl-5-oxo-2a,3,4,4a,5,6,9,10,11,12,12a,12b-dodecahydro-1*H*-7,11-methanocyclodeca[3,4]benzo[1,2-*b*]oxet-9-yl)oxy)-3-oxo-1-phenylpropan-2-yl)oxy)-4-oxobutanoic acid and (3,4-dihydroquinolin-1(2*H*)-yl)(phenyl)methanone. The product was purified by flash chromatography on silica gel (1:2 Acetone/Petroleum ether). White solid.

(*S*, *R*)-**L1**: 198.3 mg, 85% yield, 98:2 dr; (*R*, *S*)-**L1**: 196.6 mg, 84% yield, 2:98 dr.

HPLC analysis: The dr was determined via HPLC on a CHIRALPAK IF column (50.0% 2-PrOH in hexanes, 0.8 mL/min); retention times for compound obtained using (*S*, *R*)-**L1**: 27.8 min (major), 34.1 min (minor).

NMR data for the product **65**:

^{1}H NMR (400 MHz, $CDCl_3$) δ 8.18 – 8.10 (m, 2H), 7.80 – 7.71 (m, 3H), 7.62 – 7.57 (m, 1H), 7.54 – 7.48 (m, 2H), 7.45 – 7.26 (m, 8H), 7.25 – 7.13 (m, 6H), 6.99 (td, *J* = 7.5, 1.0 Hz, 1H), 6.84 (td, *J* = 7.8, 1.2 Hz, 1H), 6.51 (dd, *J* = 8.0, 1.1 Hz, 1H), 6.30 (s, 1H), 6.28 – 6.18 (m, 1H), 6.00 (dd, *J* = 9.1, 3.4 Hz, 1H), 5.68 (d, *J* = 7.1 Hz, 1H), 5.48 (d, *J* = 3.4 Hz, 1H), 5.03 – 4.93 (m, 2H), 4.49 – 4.40 (m, 1H), 4.34 – 4.16 (m, 2H), 3.81 (d, *J* = 7.0 Hz, 1H), 3.07 – 2.87 (m, 2H), 2.83 – 2.62 (m, 4H), 2.58 – 2.49 (m, 2H), 2.45 – 2.32 (m, 5H), 2.25 – 2.14 (m, 4H), 1.94 – 1.82 (m, 5H), 1.79 – 1.65 (m, 4H), 1.24 (s, 3H), 1.13 (s, 3H). ^{13}C NMR (101 MHz, $CDCl_3$) δ 205.99, 203.83, 171.38, 171.13, 170.48, 169.75, 168.10, 167.37, 166.90, 143.00, 138.24, 137.26, 134.67, 133.84, 133.55, 132.72, 132.56, 131.53, 130.70, 130.17, 129.25, 128.89, 128.87, 128.64, 128.31, 128.29, 128.00, 127.50, 127.39, 127.05, 126.44, 125.72, 125.02, 84.39, 80.94, 79.06, 76.37, 75.61, 75.12, 74.16, 72.04, 71.68, 62.65, 58.45, 52.91, 45.54, 43.10, 35.55, 35.49, 33.79, 27.47, 27.43, 26.73, 26.14, 22.53, 22.07, 20.74, 14.73, 9.54.

NMR data for the product **66**:

^{1}H NMR (400 MHz, $CDCl_3$) δ 8.17 – 8.10 (m, 2H), 7.87 – 7.78 (m, 2H), 7.68 – 7.47 (m, 4H), 7.45 – 7.26 (m, 9H), 7.25 – 7.07 (m, 5H), 7.03 – 6.95 (m, 1H), 6.82 (td, *J* = 7.8, 1.6 Hz, 1H), 6.49 (dd, *J* = 8.1, 1.2 Hz, 1H), 6.30 (s, 1H), 6.27 – 6.18 (m, 1H), 6.09 – 5.97 (m, 1H), 5.68 (d, *J* = 7.1 Hz, 1H), 5.62 – 5.50 (m, 1H), 4.96 (dd, *J* = 9.7, 2.3 Hz, 1H), 4.79 (t, *J* = 8.2 Hz, 1H), 4.49 – 4.40 (m, 1H), 4.33 – 4.16 (m, 2H), 3.81 (d, *J* = 7.0 Hz, 1H), 3.13 – 3.01 (m, 1H), 2.85 – 2.50 (m, 7H), 2.46 – 2.32 (m, 5H), 2.22 (s, 4H), 1.93 – 1.89 (m, 3H), 1.89 – 1.74 (m, 3H), 1.68 (s, 3H), 1.23 (s, 3H), 1.13 (s, 3H). ^{13}C NMR (101 MHz, $CDCl_3$) δ 206.63, 203.83, 171.37, 171.14, 170.57, 169.85, 168.04, 167.48, 166.95, 142.97, 138.12, 136.92, 134.62, 133.74, 133.55, 132.60, 132.56, 131.64, 130.74, 130.19, 129.22, 128.95, 128.91, 128.66, 128.40, 128.18, 128.00, 127.80, 127.43, 126.64, 126.41, 125.74, 125.16, 84.41, 80.96, 79.07, 76.39, 75.61, 75.12, 73.77, 72.07, 71.63, 62.61, 58.48, 52.57, 45.56, 43.11, 35.55, 35.50, 33.36, 27.62, 27.14, 26.73, 25.83, 22.53, 22.06, 20.75, 14.74, 9.55.

FT-IR (film): 3482, 1724, 1640, 1490, 1371, 1242, 1070, 708 cm^{-1}.

HRMS (ESI-MS) *m/z* $[M+Na]^+$ calcd for $C_{67}H_{68}N_2NaO_{17}$: 1195.4410, found: 1195.4406.

$[\alpha]^{25}_D$ = +10.3 (c 1.0, CH_2Cl_2); 98:2 dr from (*S*, *R*)-**L1**.

$[\alpha]^{25}_D$ = –93.7 (c 1.0, CH_2Cl_2); 2:98 dr from (*R*, *S*)-**L1**.

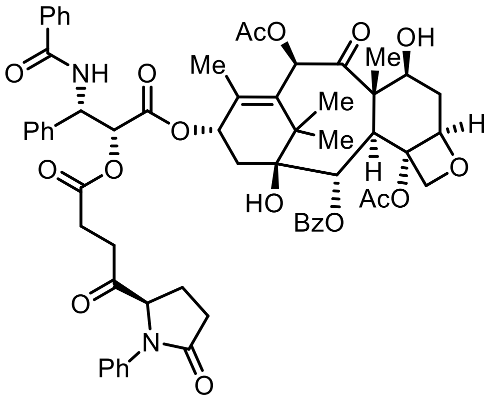

67

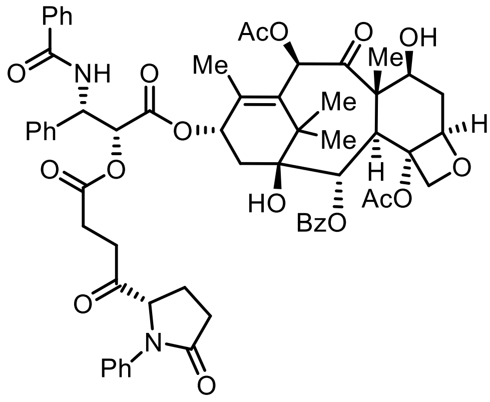

68

(2a*R*,4*S*,4a*S*,6*R*,9*S*,11*S*,12*S*,12b*S*)-9-(((2*R*,3*S*)-3-Benzamido-2-((4-oxo-4-((*R*)-5-oxo-1-phenylpyrrolidin-2-yl)butanoyl)oxy)-3-phenylpropanoyl)oxy)-12-(benzoyloxy)-4,11-dihydroxy-4a,8,13,13-tetramethyl-5-oxo-3,4,4a,5,6,9,10,11,12,12a-decahydro-1*H*-7,11-methanocyclodeca[3,4]benzo[1,2-*b*]oxete-6,12b(2a*H*)-diyl diacetate (**67**, from (*S*, *R*)-**L2**). The title compound was synthesized according to **GP-B** from 4-(((1*S*,2*R*)-1-benzamido-3-(((2a*R*,4*S*,4a*S*,6*R*,9*S*,11*S*,12*S*,12b*S*)-6,12b-diacetoxy-12-(benzoyloxy)-4,11-dihydroxy-4a,8,13,13-tetramethyl-5-oxo-2a,3,4,4a,5,6,9,10,11,12,12a,12b-dodecahydro-1*H*-7,11-methanocyclodeca[3,4]benzo[1,2-*b*]oxet-9-yl)oxy)-3-oxo-1-phenylpropan-2-yl)oxy)-4-oxobutanoic acid and 1-phenylpyrrolidin-2-one. The product was purified by flash chromatography on silica gel (2:3 Acetone/Petroleum ether). White solid.

(*S*, *R*)-**L2**: 149.3 mg, 68% yield, 95:5 dr; (*R*, *S*)-**L2**: 158.4 mg, 72% yield, 5:95 dr.

HPLC analysis: The dr was determined via HPLC on a CHIRALPAK IH column (60.0% 2-PrOH in hexanes, 0.8 mL/min); retention times for compound obtained using (*S*, *R*)-**L2**: 33.9 min (major), 54.3 min (minor).

NMR data for the product **67**:

^{1}H NMR (400 MHz, CDCl$_3$) δ 8.14 – 8.08 (m, 2H), 7.76 – 7.69 (m, 2H), 7.63 – 7.56 (m, 1H), 7.53 – 7.45 (m, 3H), 7.42 – 7.34 (m, 8H), 7.34 – 7.28 (m, 3H), 7.16 – 7.10 (m, 1H), 7.02 (d, *J* = 9.1 Hz, 1H), 6.31 – 6.25 (m, 1H), 6.22 – 6.14 (m, 1H), 5.92 (dd, *J* = 9.1, 3.7 Hz, 1H), 5.66 (d, *J* = 7.1 Hz, 1H), 5.47 (d, *J* = 3.8 Hz, 1H), 4.94 (dd, *J* = 9.6, 2.3 Hz, 1H), 4.84 – 4.74 (m, 1H), 4.47 – 4.34 (m, 1H), 4.28 (d, *J* = 8.5 Hz, 1H), 4.17 (d, *J* = 8.5 Hz, 1H), 3.78 (d, *J* = 7.0 Hz, 1H), 2.79 – 2.64 (m, 3H), 2.64 – 2.48 (m, 5H), 2.45 – 2.35 (m, 4H), 2.34 – 2.27 (m, 1H), 2.20 (s, 3H), 2.15 – 2.05 (m, 3H), 1.90 – 1.80 (m, 4H), 1.66 (s, 3H), 1.20 (s, 3H), 1.12 (s, 3H). ^{13}C NMR (101 MHz, CDCl$_3$) δ 206.31, 203.66, 174.28, 171.58, 171.05, 169.70, 167.88, 167.10, 166.81, 142.43, 137.99, 136.76, 133.59, 133.54, 132.78, 131.87, 130.10, 129.18, 129.05, 129.00, 128.59, 128.57, 128.47, 127.04, 126.60, 125.44, 121.36, 84.30, 80.93, 78.87, 76.30, 75.49, 75.01, 74.21, 71.97, 71.79, 67.05, 58.37, 52.84, 45.54, 43.07, 35.50, 35.40, 33.14, 30.59, 26.87, 26.66, 22.51, 21.96, 21.62, 20.71, 14.60, 9.50.

NMR data for the product **68**:

^{1}H NMR (400 MHz, CDCl$_3$) δ 8.15 – 8.08 (m, 2H), 7.77 – 7.69 (m, 2H), 7.64 – 7.56 (m, 1H), 7.53 – 7.45 (m, 3H), 7.43 – 7.29 (m, 11H), 7.18 – 7.10 (m, 1H), 7.00 (d, *J* = 9.0 Hz, 1H), 6.34 – 6.26 (m, 1H), 6.25 – 6.13 (m, 1H), 5.92 (dd, *J* = 9.1, 3.6 Hz, 1H), 5.66 (d, *J* = 7.1 Hz, 1H), 5.45 (d, *J* = 3.7 Hz, 1H), 4.94 (dd, *J* = 9.6, 2.3 Hz, 1H), 4.81 (dd, *J* = 9.3, 4.0 Hz, 1H), 4.48 – 4.38 (m, 1H), 4.28 (d, *J* = 8.4 Hz, 1H), 4.18 (d, *J* = 8.5 Hz, 1H), 3.79 (d, *J* = 7.0 Hz, 1H), 2.84 – 2.74 (m, 1H), 2.69 – 2.63

(m, 2H), 2.61 – 2.46 (m, 5H), 2.45 – 2.35 (m, 4H), 2.35 – 2.27 (m, 1H), 2.20 (s, 3H), 2.16 – 2.05 (m, 3H), 1.93 – 1.81 (m, 4H), 1.66 (s, 3H), 1.21 (s, 3H), 1.12 (s, 3H). ^{13}C NMR (101 MHz, $CDCl_3$) δ 206.62, 203.66, 174.26, 171.64, 171.06, 169.70, 167.88, 167.11, 166.83, 142.39, 138.00, 136.75, 133.56, 133.55, 132.85, 131.90, 130.10, 129.18, 129.07, 128.99, 128.61, 128.59, 128.44, 127.04, 126.60, 125.44, 121.23, 84.32, 80.96, 78.90, 76.31, 75.49, 75.02, 74.12, 71.96, 71.76, 67.11, 58.38, 52.73, 45.57, 43.08, 35.52, 35.42, 33.05, 30.65, 26.95, 26.68, 22.53, 21.95, 21.65, 20.72, 14.63, 9.52.

FT-IR (film): 3480, 2938, 1724, 1372, 1241, 1070, 710 cm^{-1}.

HRMS (ESI-MS) *m/z* [M+Na]$^+$ calcd for $C_{61}H_{64}N_2NaO_{17}$: 1119.4097, found: 1119.4096.

$[\alpha]^{25}_D$ = –37.3 (*c* 1.0, CH_2Cl_2); 95:5 dr from (*S*, *R*)-**L2**.

$[\alpha]^{25}_D$ = –60.5 (*c* 1.0, CH_2Cl_2); 5:95 dr from (*R*, *S*)-**L2**.

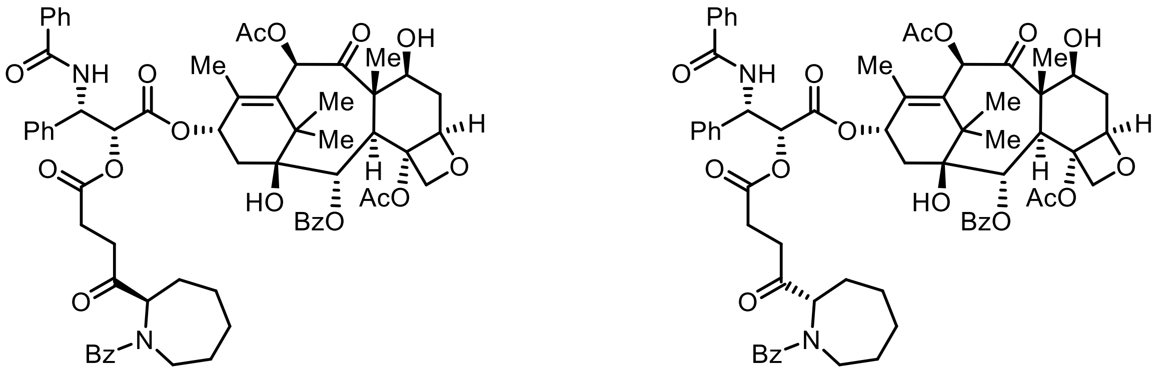

69 **70**

(2a*R*,4*S*,4a*S*,6*R*,9*S*,11*S*,12*S*,12b*S*)-9-(((2*R*,3*S*)-3-Benzamido-2-((4-((*R*)-1-benzoylazepan-2-yl)-4-oxobutanoyl)oxy)-3-phenylpropanoyl)oxy)-12-(benzoyloxy)-4,11-dihydroxy-4a,8,13,13-tetramethyl-5-oxo-3,4,4a,5,6,9,10,11,12,12a-decahydro-1*H*-7,11-methanocyclodeca[3,4]benzo[1,2-*b*]oxete-6,12b(2a*H*)-diyl diacetate (**69**, from (*S*, *R*)-**L1**). The title compound was synthesized according to **GP-B** from 4-(((1*S*,2*R*)-1-benzamido-3-(((2a*R*,4*S*,4a*S*,6*R*,9*S*,11*S*,12*S*,12b*S*)-6,12b-diacetoxy-12-(benzoyloxy)-4,11-dihydroxy-4a,8,13,13-tetramethyl-5-oxo-2a,3,4,4a,5,6,9,10,11,12,12a,12b-dodecahydro-1*H*-7,11-methanocyclodeca[3,4]benzo[1,2-*b*]oxet-9-yl)oxy)-3-oxo-1-phenylpropan-2-yl)oxy)-4-oxobutanoic acid and azepan-1-yl(phenyl)methanone. The product was purified by flash chromatography on silica gel (2:3 Acetone/Petroleum ether). White solid.

(*S*, *R*)-**L1**: 77.7 mg, 34% yield, 95:5 dr; (*R*, *S*)-**L1**: 73.6 mg, 32% yield, 7:93 dr.

HPLC analysis: The dr was determined via HPLC on a CHIRALPAK IF column (50.0% 2-PrOH in hexanes, 0.8 mL/min); retention times for compound obtained using (*S*, *R*)-**L1**: 26.2 min (major), 33.3 min (minor).

NMR data for the product **69**:

^{1}H NMR (400 MHz, $CDCl_3$) (81:19 mixture of rotamers) δ 8.15 – 8.09 (m, 2.00H), 7.85 – 7.68 (m, 3.00H), 7.62 – 7.56 (m, 1.00H), 7.54 – 7.27 (m, 14.00H), 7.25 – 7.15 (m, 1.00H), 6.28 (s, 1.00H), 6.25 – 6.16 (m, 1.00H), 6.01 – 5.91 (m, 1.00H), 5.66 (d, *J* = 7.0 Hz, 1.00H), 5.54 – 5.48 (m, 0.19H), 5.45 (d, *J* = 3.9 Hz, 0.81H), 4.94 (dd, *J* = 9.6, 2.3 Hz, 1.00H), 4.67 (dd, *J* = 11.7, 4.7 Hz, 0.81H), 4.46 – 4.38 (m, 1.00H), 4.32 – 4.14 (m, 2.19H), 3.82 – 3.73 (m, 1.19H), 3.63 – 3.56 (m, 0.81H), 3.38 – 3.19 (m, 0.81H), 3.16 – 2.97 (m, 0.81H), 2.91 – 2.62 (m, 3.19H), 2.59 – 2.48 (m, 2.19H), 2.42 – 2.24 (m, 4.00H), 2.22 – 2.15 (m, 4.00H), 2.12 – 2.02 (m, 1.00H), 1.95 – 1.85 (m, 6.00H), 1.79 – 1.69 (m, 2.00H), 1.68 – 1.61 (m, 4.00H), 1.51 – 1.30 (m, 3.00H), 1.21 (s, 3.00H), 1.12 (s, 3.00H). ^{13}C NMR

(101 MHz, $CDCl_3$) major isomer δ 206.67, 203.82, 172.70, 171.63, 171.11, 169.78, 168.30, 167.45, 166.86, 142.95, 137.29, 136.14, 133.95, 133.54, 132.61, 131.52, 130.16, 129.50, 129.28, 128.86, 128.61, 128.40, 128.29, 127.41, 127.09, 126.33, 84.40, 80.93, 78.98, 76.36, 75.60, 75.11, 74.19, 72.01, 71.64, 65.14, 58.44, 53.03, 47.37, 45.54, 43.10, 35.49, 34.25, 30.66, 28.55, 28.25, 27.56, 26.72, 26.23, 22.47, 22.06, 20.73, 14.73, 9.54.

NMR data for the product **70**:

^{1}H NMR (400 MHz, $CDCl_3$) (83:17 mixture of rotamers) δ 8.18 – 8.08 (m, 2.00H), 7.87 – 7.69 (m, 2.83H), 7.64 – 7.54 (m, 1.17H), 7.52 – 7.46 (m, 2.17H), 7.44 – 7.27 (m, 10.83H), 7.25 – 7.16 (m, 1.83H), 7.06 – 6.99 (m, 0.17H), 6.29 (s, 1.00H), 6.26 – 6.15 (m, 1.00H), 6.01 – 5.90 (m, 1.00H), 5.66 (d, *J* = 7.1 Hz, 1.00H), 5.54 – 5.49 (m, 0.83H), 5.45 (d, *J* = 4.0 Hz, 0.17H), 5.01 – 4.90 (m, 1.00H), 4.74 – 4.58 (m, 0.83H), 4.51 – 4.33 (m, 1.17H), 4.30 – 4.16 (m, 2.00H), 3.90 – 3.68 (m, 1.17H), 3.62 – 3.54 (m, 0.83H), 3.31 – 3.18 (m, 0.83H), 3.17 – 2.98 (m, 0.17H), 2.94 – 2.48 (m, 6.00H), 2.46 – 2.29 (m, 4.00H), 2.25 – 2.17 (m, 3.83H), 2.12 – 2.04 (m, 2.17H), 1.95 – 1.82 (m, 5.00H), 1.80 – 1.72 (m, 1.17H), 1.71 – 1.57 (m, 4.83H), 1.52 – 1.39 (m, 1.83H), 1.36 – 1.27 (m, 1.17H), 1.20 (s, 3.00H), 1.11 (s, 3.00H). ^{13}C NMR (101 MHz, $CDCl_3$) major isomer δ 206.68, 203.77, 172.76, 171.59, 171.06, 169.85, 167.89, 167.44, 166.86, 142.83, 137.11, 136.07, 133.63, 133.50, 132.63, 131.52, 130.14, 129.45, 129.24, 128.79, 128.61, 128.37, 128.24, 127.34, 126.69, 126.26, 84.37, 80.93, 78.94, 76.34, 75.56, 75.10, 74.18, 71.97, 71.55, 64.97, 58.41, 52.85, 47.32, 45.55, 43.08, 35.51, 33.87, 30.59, 28.34, 28.27, 27.70, 26.65, 26.07, 22.53, 22.05, 20.70, 14.68, 9.53.

FT-IR (film): 3448, 2931, 1723, 1613, 1243, 1071, 707 cm^{-1}.

HRMS (ESI-MS) *m/z* $[M+H]^+$ calcd for $C_{64}H_{71}N_2O_{17}$: 1139.4747, found: 1139.4746.

$[\alpha]^{25}_{D}$ = –44.4 (*c* 1.0, CH_2Cl_2); 95:5 dr from (*S*, *R*)-**L1**.

$[\alpha]^{25}_{D}$ = –55.8 (*c* 1.0, CH_2Cl_2); 7:93 dr from (*R*, *S*)-**L1**.

Gram-scale synthesis of 5:

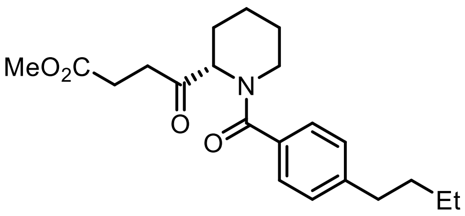

5

In a glovebox, a 200-mL flask, equipped with a Teflon stir bar, was charged with Ir[dF(CF$_3$)ppy]$_2$(dtbbpy)PF$_6$ (89.8 mg, 0.080 mmol, 1 mol%), NiCl$_2$·glyme (175.8 mg, 0.80 mmol, 10 mol%), (*R, S*)-**L2** (534.2 mg, 1.04 mmol, 13 mol%), (4-butylphenyl)(piperidin-1-yl)methanone (5.89 g, 24.0 mmol, 3.0 equiv), anhydrous PhH (80.0 mL), and the reaction mixture was allowed to stir at room temperature for 45 min, after which it turned to a pale yellow suspension. Next, 4-methoxy-4-oxobutanoic acid (1056.9 mg, 8.0 mmol, 1.0 equiv) was added as a solid, and followed by the addition of 2,6-lutidine (1.40 mL, 12.0 mmol, 1.5 equiv) via syringe. Then, DMDC (1.29 mL, 12.0 mmol, 1.5 equiv) was added dropwise via syringe. The flask was closed with a rubber stopper and wrapped with electrical tape. Next, the reaction mixture was transferred out of the glovebox, and then vacuum grease was liberally applied to cover the entire top of septum cap. Then, the reaction mixture was cooled to 20 °C and stirred at that temperature for 3 min before irradiation. The reaction was stirred at 20 °C for 28 hours under blue LED irradiation with 3*40W blue LED lamps (Kessil PR160L, 427 nm). The reaction mixture was then passed through a short pad of silica gel, with acetone as the eluent (~400 mL). The resulting mixture was concentrated, and the residue was purified by flash chromatography on silica gel (1:4 Acetone/Petroleum ether). Yellow oil.

(*R, S*)-**L2**: 1.710 g, 59% yield, 94% ee.

Iterative coupling sequence:

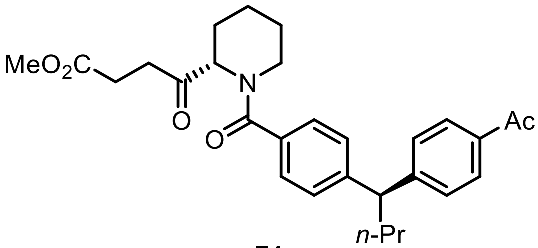

71

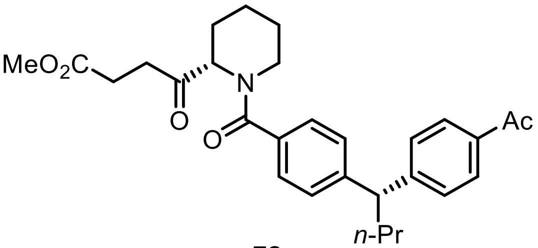

72

Methyl 4-((*S*)-1-(4-((*R*)-1-(4-acetylphenyl)butyl)benzoyl)piperidin-2-yl)-4-oxobutanoate (**71**, from (*S*)-**L6**).[2,3] In a glovebox, Ir[dF(CF$_3$)ppy]$_2$(dtbbpy)PF$_6$ (2.2 mg, 0.002 mmol, 1 mol%), NiBr$_2$·glyme (6.2 mg, 0.020 mmol, 10 mol%), (*S*)-**L6** (17.0 mg, 0.030 mmol, 15 mol%), K$_2$CO$_3$ (55.3 mg, 0.40 mmol, 2.0 equiv), methyl (*R*)-4-(1-(4-butylbenzoyl)piperidin-2-yl)-4-oxobutanoate (215.7 mg, 0.60 mmol, 3.0 equiv), a Teflon stir bar, and anhydrous EtOAc (2.0 mL) was add sequentially to 15-mL vial. The reaction mixture was stirred at room temperature for 30 min, after which it turned to a pale yellow suspension. 1-(4-Bromophenyl)ethan-1-one

(39.8 mg, 0.20 mmol, 1.0 equiv) was added as a solid. Next, the vial was closed with a PTFE septum cap and wrapped with electrical tape. Then, the vial was transferred out of the glovebox, and then vacuum grease was liberally applied to cover the punctures in the septum cap. Then, the reaction mixture was cooled to 20 °C and stirred at that temperature for 1 min before being irradiated with a 40 W the blue LED lamp (Kessil PR160L, 427 nm). The reaction was stirred under blue LED irradiation at 20 °C for 24 hours. The reaction mixture was then passed through a short pad of silica gel, with acetone as the eluent (~20 mL). The resulting mixture was concentrated, and the residue was purified by preparative TLC on silica gel (2:7 Acetone/Petroleum ether). Yellow oil.

(*S*)-**L6**: 46.3 mg, 48% yield, 92:8 dr; (*R*)-**L6**: 39.4 mg, 41% yield, 8:92 dr.

HPLC analysis: The dr was determined via HPLC on a CHIRALPAK IH column (40.0% 2-PrOH in hexanes, 0.8 mL/min); retention times for compound obtained using (*S*)-**L6**: 24.2 min (major), 31.8 min (minor).

NMR data for the product **71**:

^{1}H NMR (500 MHz, $CDCl_3$) (86:14 mixture of rotamers) δ 7.88 (d, *J* = 8.0 Hz, 2.00H), 7.44 – 7.26 (m, 6.00H), 5.35 (d, *J* = 5.6 Hz, 0.86H), 4.65 (d, *J* = 13.6 Hz, 0.14H), 4.36 (s, 0.14H), 4.00 (t, *J* = 7.8 Hz, 1.00H), 3.74 – 3.59 (m, 4.00H), 3.25 – 3.10 (m, 0.86H), 2.92 – 2.78 (m, 1.86H), 2.63 (t, *J* = 6.4 Hz, 1.86H), 2.56 (s, 3.28H), 2.36 (d, *J* = 13.6 Hz, 0.86H), 2.04 (q, *J* = 7.8 Hz, 2.14H), 1.76 – 1.68 (m, 2.00H), 1.59 – 1.53 (m, 0.86H), 1.49 – 1.36 (m, 2.00H), 1.31 – 1.25 (m, 2.14H), 0.93 (t, *J* = 7.3 Hz, 3.00H). ^{13}C NMR (126 MHz, $CDCl_3$) major isomer δ 206.98, 197.63, 173.12, 171.32, 150.18, 146.15, 135.35, 133.64, 128.58, 128.06, 127.91, 127.25, 58.51, 51.74, 50.88, 46.25, 37.39, 33.82, 27.41, 26.46, 25.59, 25.08, 20.93, 20.66, 13.89.

NMR data for the product **72**:

^{1}H NMR (500 MHz, $CDCl_3$) (86:14 mixture of rotamers) δ 7.87 (d, *J* = 7.8 Hz, 1.72H), 7.44 – 7.26 (m, 5.00H), 7.26 – 7.20 (m, 1.28H), 5.34 (d, *J* = 5.7 Hz, 0.86H), 4.64 (d, *J* = 13.4 Hz, 0.14H), 4.35 (s, 0.14H), 3.99 (t, *J* = 7.9 Hz, 1.00H), 3.74 – 3.60 (m, 4.00H), 3.16 (t, *J* = 12.5 Hz, 0.86H), 2.87 – 2.79 (m, 1.86H), 2.62 (t, *J* = 6.5 Hz, 1.86H), 2.55 (s, 3.28H), 2.35 (d, *J* = 13.7 Hz, 0.86H), 2.03 (q, *J* = 7.8 Hz, 2.14H), 1.75 – 1.68 (m, 2.00H), 1.55 (d, *J* = 10.9 Hz, 0.86H), 1.46 – 1.36 (m, 2.00H), 1.30 – 1.24 (m, 2.14H), 0.92 (t, *J* = 7.3 Hz, 3.00H). ^{13}C NMR (126 MHz, $CDCl_3$) major isomer δ 206.97, 197.62, 173.12, 171.32, 150.21, 146.15, 135.36, 133.65, 128.59, 128.06, 127.91, 127.25, 58.51, 51.74, 50.89, 46.25, 37.39, 33.82, 27.41, 26.46, 25.60, 25.08, 20.93, 20.66, 13.89.

FT-IR (film): 3474, 2955, 2870, 1738, 1682, 1633, 1422, 1269, 737 cm^{-1}.

HRMS (ESI-MS) *m/z* $[M+H]^+$ calcd for $C_{29}H_{36}NO_5$: 478.2588, found: 478.2586.

$[\alpha]^{25}_{D}$ = –51.1 (*c* 1.0, CH_2Cl_2); 92:8 dr from (*S*)-**L6**.

$[\alpha]^{25}_{D}$ = –53.2 (*c* 1.0, CH_2Cl_2); 8:92 dr from (*R*)-**L6**.

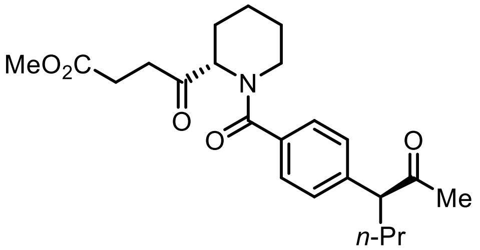

73

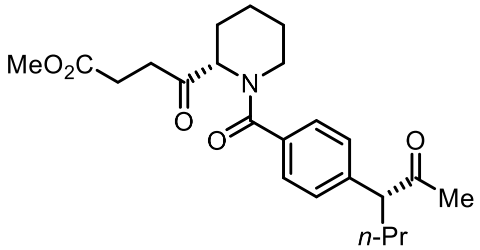

74

Methyl 4-oxo-4-((*S*)-1-(4-((*S*)-2-oxohexan-3-yl)benzoyl)piperidin-2-yl)butanoate (**73**, from (*S*)-**L3**).[4] In a glovebox, Ir[dF(CF_3)ppy]$_2$(dtbbpy)PF_6 (2.2 mg, 0.002 mmol, 1 mol%), $NiBr_2$·glyme (6.2 mg, 0.020 mmol, 10 mol%), (*S*)-**L3** (9.4 mg, 0.026 mmol, 13 mol%), NH_4Cl (10.7 mg, 0.20 mmol, 1.0 equiv), Na_2HPO_4 (42.6 mg, 0.30 mmol, 1.5 equiv), methyl (*R*)-4-(1-(4-butylbenzoyl)piperidin-2-yl)-4-oxobutanoate (215.7 mg, 0.60 mmol, 3.0 equiv), a Teflon stir bar, and anhydrous *i*-PrOAc (2.0 mL) was add sequentially to 15-mL vial. The reaction mixture was stirred at room temperature for 30 min, after which it turned to a purple suspension. Next, the vial was closed with a PTFE septum cap and wrapped with electrical tape. Then, acetic acid (11.5 μL, 0.20 mmol, 1.0 equiv) and DMDC (32.2 μL, 0.30 mmol, 1.5 equiv) were added sequentially via microsyringe. Next, the vial was transferred out of the glovebox, and then vacuum grease was liberally applied to cover the punctures in the septum cap. Then, the reaction mixture was cooled to 10 °C and stirred at that temperature for 1 min before being irradiated with a 40 W the blue LED lamp (Kessil PR160L, 427 nm). The reaction was stirred under blue LED irradiation at 10 °C for 25 hours. The reaction mixture was then passed through a short pad of silica gel, with acetone as the eluent (~20 mL). The resulting mixture was concentrated, and the residue was purified by preparative TLC on silica gel (1:3 Acetone/Petroleum ether). Colorless oil.

(*S*)-**L3**: 64.2 mg, 80% yield, 97:3 dr; (*R*)-**L3**: 65.1 mg, 81% yield, 2:98 dr.

HPLC analysis: The dr was determined via HPLC on a CHIRALPAK AD-3 column (20.0% 2-PrOH in hexanes, 1.0 mL/min); retention times for compound obtained using (*S*)-**L3**: 18.1 min (major), 19.7 min (minor).

NMR data for the product **73**:

^{1}H NMR (500 MHz, $CDCl_3$) (83:17 mixture of rotamers) δ 7.42 – 7.34 (m, 1.66H), 7.31 – 7.27 (m, 0.32H), 7.26 – 7.20 (m, 2.00H), 5.33 (d, *J* = 4.4 Hz, 0.83H), 4.63 (d, *J* = 13.8 Hz, 0.17H), 4.34 (s, 0.17H), 3.71 – 3.55 (m, 5.00H), 3.24 – 3.12 (m, 0.83H), 2.89 – 2.77 (m, 1.83H), 2.62 (t, *J* = 6.4 Hz, 1.83H), 2.55 (brs, 0.34H), 2.39 – 2.30 (m, 0.83H), 2.25 (d, *J* = 13.2 Hz, 0.17H), 2.04 (s, 2.83H), 2.01 – 1.93 (m, 1.17H), 1.76 – 1.68 (m, 2.00H), 1.67 – 1.61 (m, 1.17H), 1.58 – 1.53 (m, 0.83H), 1.48 – 1.34 (m, 2.00H), 1.22 – 1.14 (m, 2.00H), 0.85 (t, *J* = 7.4 Hz, 3.00H). ^{13}C NMR (126 MHz, $CDCl_3$) major isomer δ 207.92, 206.87, 173.07, 171.09, 140.80, 134.48, 128.29, 127.45, 59.16, 58.47, 51.72, 46.20, 33.88, 33.76, 29.13, 27.36, 25.55, 25.07, 20.61, 20.47, 13.82.

NMR data for the product **74**:

^{1}H NMR (500 MHz, $CDCl_3$) (83:17 mixture of rotamers) δ 7.39 (d, *J* = 8.0 Hz, 1.66H), 7.30 – 7.27 (m, 0.34H), 7.24 (d, *J* = 8.0 Hz, 2.00H), 5.33 (d, *J* = 4.7 Hz, 0.83H), 4.63 (d, *J* = 13.9 Hz, 0.17H), 4.34 (s, 0.17H), 3.70 – 3.55 (m, 5.00H), 3.26 – 3.07 (m, 0.83H), 2.94 – 2.73 (m, 1.83H), 2.61 (t, *J* = 6.4 Hz, 1.83H), 2.55 (s, 0.34H), 2.43 – 2.29 (m, 0.83H), 2.24 (d, *J* = 13.6 Hz, 0.17H), 2.06 – 1.92 (m, 4.00H), 1.76 – 1.67 (m, 2.00H), 1.67 – 1.61 (m, 1.17H), 1.56 (d, *J* = 8.9 Hz, 0.83H), 1.47 – 1.35 (m, 1.83H), 1.22 – 1.13 (m, 2.17H), 0.85 (t, *J* = 7.3 Hz, 3.00H). ^{13}C NMR (126 MHz, $CDCl_3$) major

isomer δ 207.89, 206.86, 173.06, 171.09, 140.81, 134.48, 128.28, 127.45, 59.17, 58.48, 51.72, 46.20, 33.87, 33.76, 29.09, 27.36, 25.54, 25.07, 20.61, 20.46, 13.82.

FT-IR (film): 3474, 2956, 1716, 1632, 1424, 1356, 1166, 848 cm^{-1}.

HRMS (ESI-MS) *m/z* $[M+Na]^+$ calcd for $C_{23}H_{31}NNaO_5$: 424.2094: found: 424.2108.

$[\alpha]^{25}_D$ = +46.5 (*c* 1.0, CH_2Cl_2); 97:3 dr from (*S*)-**L3**.

$[\alpha]^{25}_D$ = –156.8 (*c* 1.0, CH_2Cl_2); 2:98 dr from (*R*)-**L3**.

1.6 Mechanistic Studies

A. The importance of chloride in the reaction.

The procedure is the same as **GP-A**, and the reactions were quenched through a small plug of silica gel, which was flushed with acetone.

The yield was determined via GC analysis with tetradecane as an internal standard. The ee values were determined via HPLC analysis after purification by preparative TLC.

Supplementary Table 8. The importance of chloride for the reactions.

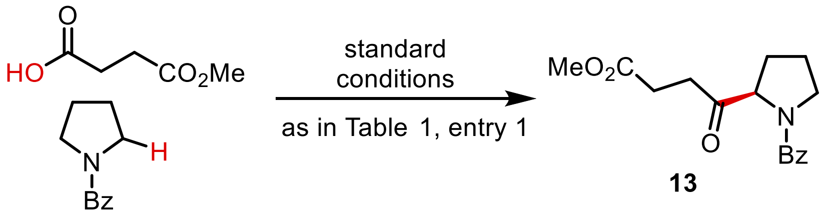

13

entry	Variation	results
1	$NiCl_2$·glyme (none)	73% yield, 97% ee
2	$NiBr_2$·glyme	8% yield, 98% ee
3	$Ni(COD)_2$	2% yield, ee: ND
4	$Ni(COD)_2$, 1.5 equiv of **LiCl** added	6% yield, ee: ND
5	$Ni(COD)_2$, **[Ir(III)]Cl** instead of $[Ir(III)]PF_6$	2% yield, ee: ND
6	$Ni(acac)_2$	28% yield, 89% ee
7	$Ni(acac)_2$, 1.5 equiv of **LiCl** added	64% yield, 95% ee
8	$Ni(acac)_2$, **[Ir(III)]Cl** instead of $[Ir(III)]PF_6$	38% yield, 60% ee

The use of chloride-free nickel salts in combination with [Ir(III)]Cl as a photocatalyst led to significantly lower efficiency (entries 3, 5, 6, and 8), which does not support the oxidatively chlorine generation (*E* > +1.21 V vs SCE in CH_3CN: S. Rohe, A. O. Morris, T. McCallum, L. Barriault, *Angew. Chem. Int. Ed.* **2018**, *57*, 15664–15669.).

B. The intermediacy of the *α*-amino radical.

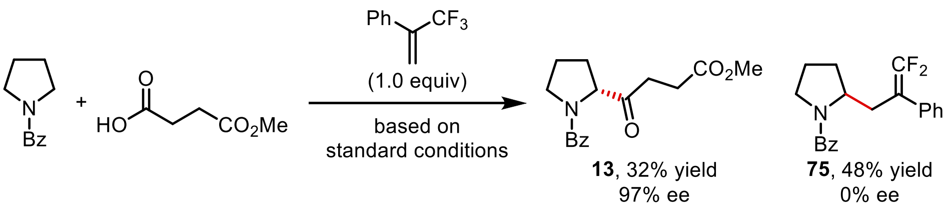

Control experiment in the absence of an acyl surrogate and the proposed reaction pathway:

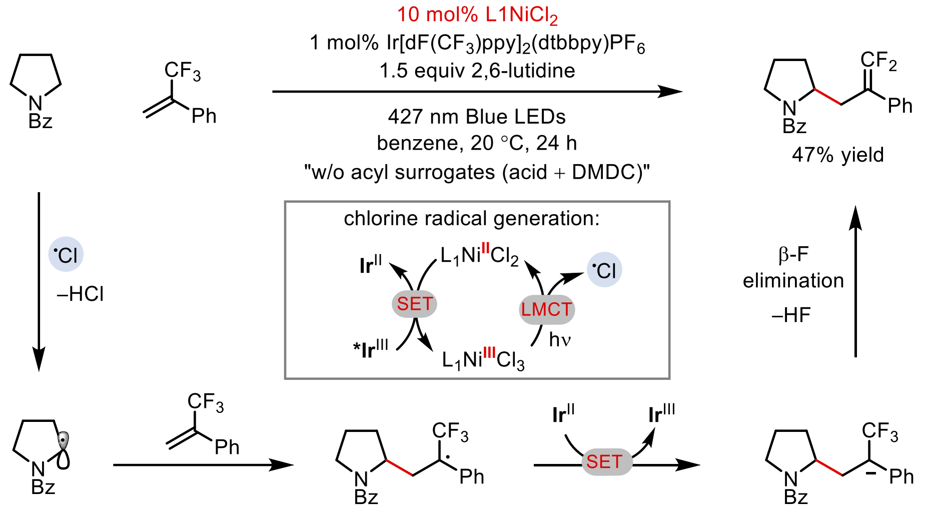

Supplementary Fig. 1 The intermediacy of α-amino radicals.

The procedure is the same as **GP-A**, except for the following change: following the addition of 4-methoxy-4-oxobutanoic acid and dimethyl dicarbonate, (3,3,3-trifluoroprop-1-en-2-yl)benzene (15.0 μL, 0.10 mmol, 1.0 equiv) was added by a microsrisyringe.

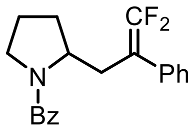

75

(2-(3,3-Difluoro-2-phenylallyl)pyrrolidin-1-yl)(phenyl)methanone. The title compound was synthesized according to **GP-A** from *N*-Bz-pyrrolidine and (3,3,3-trifluoroprop-1-en-2-yl)benzene (15.0 μL, 0.10 mmol, 1.0 equiv). The pure product was isolated by preparative TLC on silica gel (1:5 EtOAc/Petroleum ether) to give a yellow oil (15.6 mg, 48% yield).

^{1}H NMR (400 MHz, $CDCl_3$) (80:20 mixture of rotamers) δ 7.60 – 7.41 (m, 2.00H), 7.41 – 7.27 (m, 5.20H), 7.25 – 7.06 (m, 2.40H), 6.68 (d, *J* = 7.4 Hz, 0.40H), 4.42 – 4.24 (m, 0.80H), 3.85 – 3.78 (m, 0.20H), 3.75 – 3.68 (m, 0.40H), 3.39 – 3.23 (m, 1.60H), 3.19 – 3.06 (m, 0.80H), 2.86 – 2.71 (m, 0.80H), 2.40 – 2.29 (m, 0.40H), 2.04 – 1.94 (m, 1.00H), 1.91 – 1.83 (m, 1.00H), 1.77 – 1.63 (m, 2.00H). ^{13}C NMR (101 MHz, $CDCl_3$) δ 170.09, 169.88, 154.51 (dd, $J_{C\text{-}F}$ = 293.5, 287.7 Hz), 136.93, 133.10, 129.87, 129.30, 128.60, 128.54, 128.12 (t, $J_{C\text{-}F}$ = 3.2 Hz), 127.98, 127.52, 127.31, 127.23, 126.45, 89.95 (dd, *J* = 21.4, 13.5 Hz), 56.83, 56.04, 50.27, 45.80, 31.44, 30.12, 29.23, 24.96, 21.78. ^{19}F NMR (471 MHz, $CDCl_3$) δ −89.81 (dd, *J* = 115.2 Hz, 39.6 Hz, 0.40F), −90.20 (dd, *J* = 151.1 Hz, 41.1 Hz, 1.60F).

All spectroscopic data were consistent with the literature.[3]

C. Stoichiometric experiments with pregenerated Ni(II) acyl chloride species.

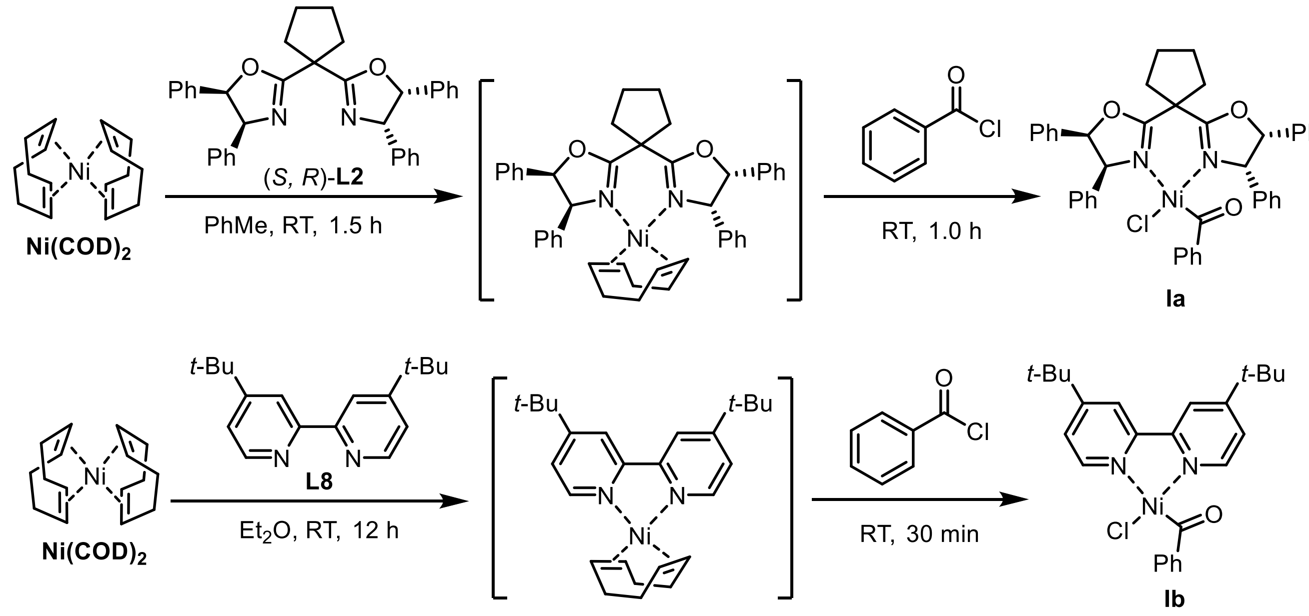

Supplementary Fig. 2 Synthesis of oxidative addition complex.

Synthesis of oxidative addition complex. The complexes were synthesized in analogy with the reported procedure.[5]

For **Ia**: In a 40-mL vial equipped with a PTFE-coated stirring bar, (*S, R*)-**L2** (512.7 mg, 1.00 mmol, 1.0 equiv) and $Ni(COD)_2$ (275.1 mg, 1.00 mmol, 1.0 equiv) were charged in a glovebox, then the dry toluene (10.0 ml) was added. The reaction was stirred at room temperature for 1.5 h, then benzoyl chloride (116.0 μL, 1.0 mmol, 1.0 equiv) were added via a microsyringe and the reaction was stirred at room temperature for 1.0 h. The complex was precipitated by adding pentane (25 ml), then was collected by filtration and washed thoroughly with pentane. The solid was dried in vacuo, affording **Ia** as a brown powder (372.5 mg, 52%), which was stored in the glovebox at –30 °C. Attempts to obtain crystals suitable for X-ray crystallography have been unsuccessful.

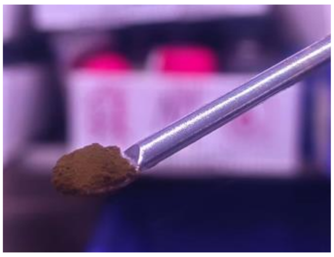

Note: *The sample is paramagnetic. ^{1}H and ^{13}C NMR showed signal broadening, therefore no complete analytical characterization was possible.*

HRMS (ESI-MS) calcd for ($[C_{42}H_{37}ClN_2NiO_3–Cl+MeCN]^+$): 716.2418, found: 716.2436.

Note: *A Ni(II) acyl chloride complex derived from the achiral ligand dtbbpy that has a comparable reactivity (Table 1, entry 8), can be isolated and characterized (as shown below).*

For **Ib**: In a glovebox, in a 20-mL vial equipped with a PTFE-coated stirring bar, 4,4′-di-*tert*-butyl-2,2′-pyridine (215.0 mg, 0.8 mmol, 1.0 equiv) and $Ni(COD)_2$ (220.0 mg , 0.8 mmol, 1.0 equiv) were charged in a glovebox, then the dry Et_2O (8.0 ml) was added. The resulting mixture was stirred for 12 h at room temperature. To the vial was added benzoyl chloride (93.0 μL, 0.8 mmol, 1.0 equiv) and stirred for 30 min. The resulting red suspension was collected by filtration and washed thoroughly with Et_2O. The solid was dried in vacuo, affording **Ib** as a red powder (246.9 mg, 66%), which was stored in the glovebox at –30 °C.

^1H NMR (500 MHz, CD_2Cl_2) δ 8.88 (s, 1H), 8.58 (d, *J* = 4.4 Hz, 2H), 7.86 (d, *J* = 9.7 Hz, 2H), 7.75 (s, 1H), 7.52 (s, 1H), 7.49 – 7.40 (m, 3H), 7.21 (s, 1H), 1.41 (s, 9H), 1.35 (s, 9H).

All spectroscopic data were consistent with the literature.[5]

Supplementary Table 9. Stoichiometric experiments with pregenerated Ni(II) acyl chloride species.

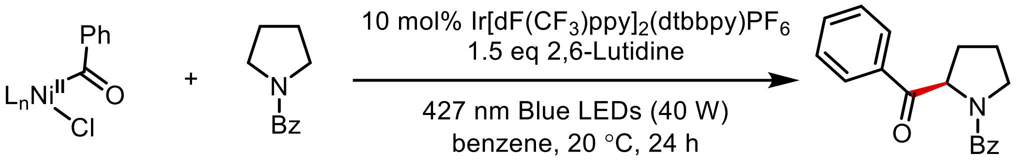

1.0 equiv x equiv **36**

entry	conditions	results
1	**Ia**, 3.0 equiv *N*-Bz pyrrolidine, benzene (0.5 mL)	8% yield, 60% ee
2	**Ia**, 15.0 equiv *N*-Bz pyrrolidine, benzene (1.0 mL)	22% yield, 69% ee
3	**Ib**, 3.0 equiv *N*-Bz pyrrolidine, benzene (0.5 mL)	3% yield, 0% ee
4	**Ib**, 15.0 equiv *N*-Bz pyrrolidine, benzene (1.0 mL)	11% yield, 0% ee

Representative procedure for stoichiometric experiments.

In a glovebox, **Ia** or **Ib** (0.05 mmol, 1.0 equiv), $Ir[dF(CF_3)ppy]_2(dtbbpy)PF_6$ (5.5 mg, 0.005 mmol, 10 mol%), *N*-Bz-pyrrolidine (131.5 mg, 0.75 mmol, 15.0 equiv), a Teflon stir bar, and anhydrous benzene (1.0 mL) were added sequentially to a 4-mL vial. Next, the vial was sealed with a septum cap and wrapped with electrical tape. Then, 2,6-lutidine (9.0 μL, 0.075 mmol, 1.5 equiv) was added via a microsyringe. The vial was transferred out of the glovebox. Then, the reaction mixture was stirred at 20 °C in an EtOH bath for 1 min before being irradiated with a 40 W the blue LED lamp (Kessil PR160L, 427 nm). The reaction was stirred under blue LED irradiation at 20 °C for 24 hours. Next, the lamp was turned off and tetradecane (13.0 μL, 0.05 mmol) was added as an internal standard. The mixture was filtered through a small plug of silica gel, which was flushed with acetone (~5 mL). The yield was determined via GC analysis with tetradecane as an internal standard. The ee values were determined via HPLC analysis after purification by preparative thin-layer chromatography.

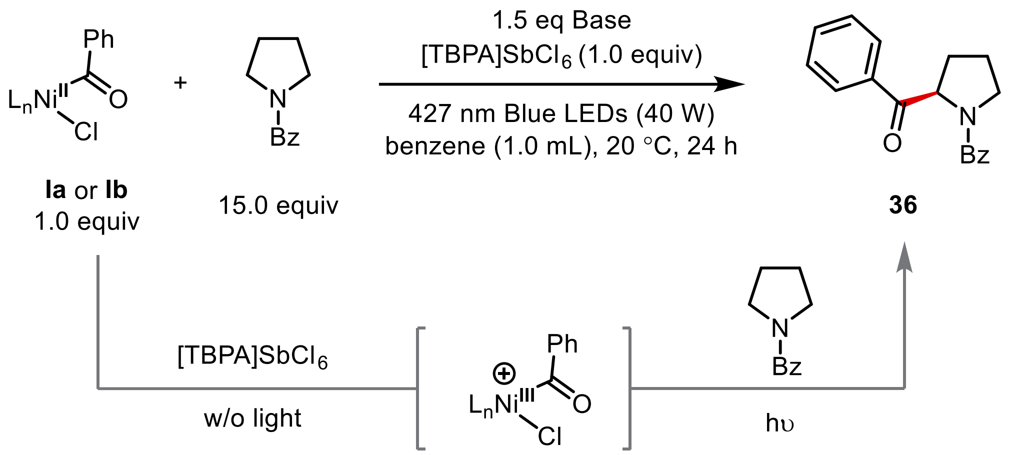

entry	Base	Yield of **36**
1	2,6-Lutidine	0%
2	$NaHCO_3$	0%

Supplementary Fig. 3 Stoichiometric oxidation experiments with pregenerated Ni(II) acyl chloride species.

Representative procedure for stoichiometric oxidation experiments. In a glovebox, **Ia** or **Ib** (0.05 mmol, 1.0 equiv), *N*-Bz-pyrrolidine (131.5 mg, 0.75 mmol, 15.0 equiv), [TBPA]$SbCl_6$ (40.9 mg, 0.05 mmol, 1.0 equiv), a Teflon stir bar, and anhydrous benzene (1.0 mL) were added sequentially to a 4-mL vial. The vial was sealed with a septum cap and wrapped with electrical tape. Next, 2,6-lutidine (9.0 µL, 0.075 mmol, 1.5 equiv) was added via a microsyringe. The vial was transferred out of the glovebox. Then, the reaction mixture was stirred at 20 °C in an EtOH bath for 1 min before being irradiated with a 40 W the blue LED lamp (Kessil PR160L, 427 nm). The reaction was stirred under blue LED irradiation at 20 °C for 24 hours. Next, the lamp was turned off and tetradecane (13.0 µL, 0.05 mmol) was added as an internal standard. The mixture was filtered through a small plug of silica gel, which was flushed with acetone (~5 mL). The yield was determined via GC analysis with tetradecane as an internal standard.

D. Cyclic voltammogram studies and luminescence quenching experiments.

Cyclic voltammogram studies. The cyclic voltammograms were recorded in an electrolyte of Et_4NPF_6 (0.1 M) in MeCN at 20 °C using a glassy carbon disk working electrode (diameter, 1 mm), a Pt wire auxiliary electrode and a SCE reference electrode. The scan rate was 100 mV/s.

Synthesis of L1$NiCl_2$. In a 15-mL vial equipped with a PTFE-coated stirring bar, (*S*, *R*)-**L1** (12.6 mg, 0.018 mmol, 1.2 equiv) and $NiCl_2$·glyme (3.3 mg, 0.015 mmol, 1.0 equiv) were charged in a glovebox, then the dry MeCN (5.0 ml) was added. The reaction was stirred at room temperature for 0.5 hours. The complex was used in cyclic voltammogram studies without further purification.

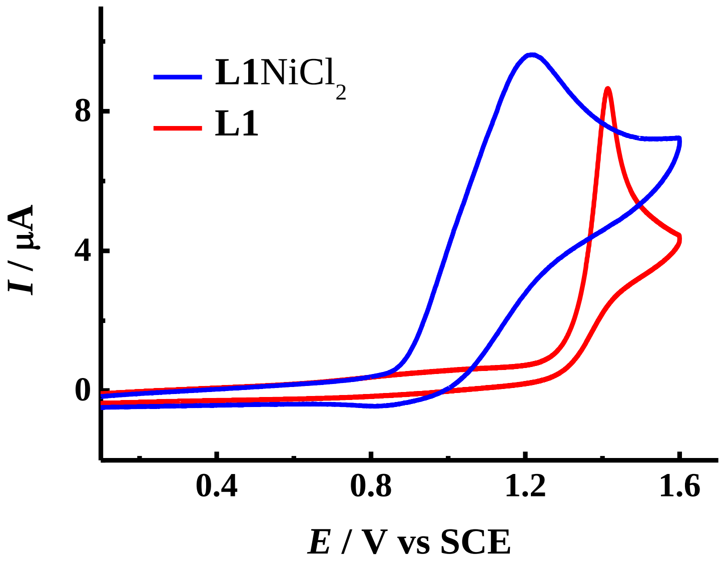

Supplementary Fig. 4 Cyclic voltammograms of the **L1**$NiCl_2$ (3.0 mM) and the chiral ligand ((*S*, *R*)-**L1**) (3.0 mM) in MeCN.

Steady-state emission spectra were obtained at room temperature using a Hitachi F7000 fluorescence spectrophotometer with a scan range of 430 nm → 600 nm at a scan rate of 2400 nm/min and resolution of 0.5 nm. Emission slit width was maintained at 1.0 nm and excitation slit width was maintained at 2.5 nm. The iridium complex solutions were excited at 380 nm.

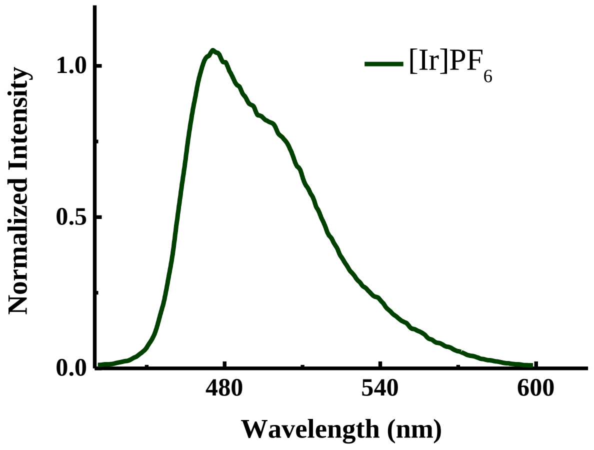

Supplementary Fig. 5 Steady-state emission spectra for [Ir]PF_6 in EtOAc (40 μm)

Luminescence quenching experiments. Emission intensities were recorded on a Hitachi F7000 fluorescence spectrophotometer in a 10.0 mm quartz cuvette. These solution of [Ir]PF_6 was excited at 425 nm and the emission intensity was measured at 475 nm (emission maximum). The concentration was 40 μm in EtOAc. The concentration of the quencher stock solution was 100 mM in EtOAc (**L1** and 78) or THF (**L1**$NiCl_2$) or DME (LiCl). For each quenching experiment, 10 μL of this stock solution were titrated to a solution (2.0 mL) of [Ir]PF_6 (40 μM in EtOAc) in a screw-top 10.0 mm quartz cuvette. The addition of 10 μL stock solution refers to an increase of the quencher concentration of 0.5 mM. After degassing with an argon stream for 3 minutes, the emission intensity was collected.

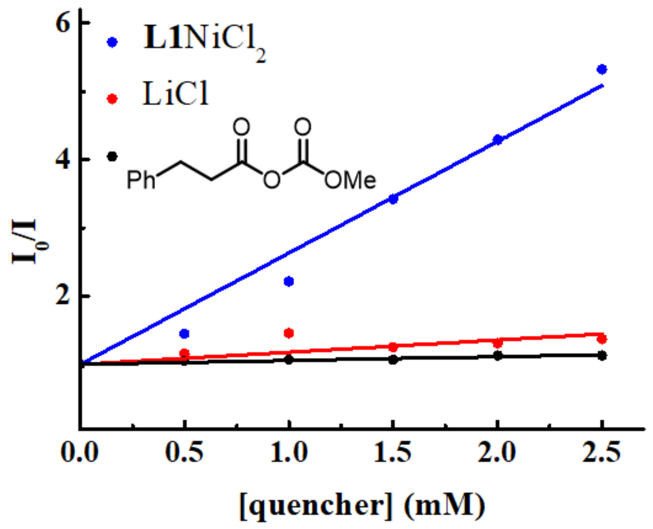

Supplementary Fig. 6 Luminescence quenching experiments.

1.7 Assignment of Absolute Configuration

The configuration of the coupling product **40** illustrated in Figure 2 using (*S, R*)-**L2**, was determined via X-ray crystallography.

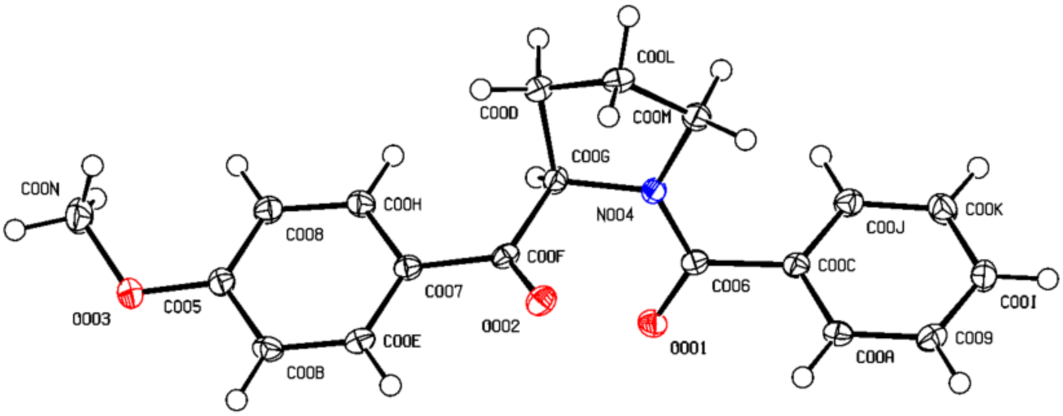

Supplementary Fig. 7 Thermal ellipsoid plot at the 50% probability level.

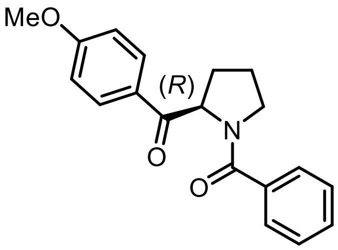

(*R*)-1-(4-(1-(3-Methoxybenzoyl)pyrrolidin-2-yl)phenyl)ethan-1-one. X-ray quality crystals were obtained by slow evaporation of a saturated solution in hexane and DCM of a sample synthesized using (*S, R*)-**L2**. A crystal of $C_{19}H_{19}NO_3$ was selected and mounted in a nylon loop in immersion oil. All measurements were made on a XtaLAB Synergy, Dualflex, HyPix diffractometer with filtered Cu-Kα radiation at a temperature of 100.15 K. Using Olex2[6], the structure was solved with the olex2.solve[7] structure solution program using direct methods and refined with the SHELXL[8] refinement package using least squares minimization. The absolute stereochemistry was determined on the basis of the absolute structure parameter.

Supplementary Table 10. Crystal data and structure refinement for the product 40 in Fig. 3.

Identification code	**compound 40**	
Empirical formula	$C_{19}H_{19}NO_3$	
Formula weight	309.35	
Temperature	100.15 K	
Wavelength	1.54184 Å	
Crystal system	orthorhombic	
Space group	$P2_12_12_1$	
Unit cell dimensions	a = 6.33970(10) Å	α= 90°
	b = 11.0813(2) Å	β= 90°
	c = 22.5319(4) Å	γ = 90°
Volume	1582.91(5) $Å^3$	
Z	4	
Density (calculated)	1.298 g/cm^3	
Absorption coefficient	0.709 mm^{-1}	
F(000)	656.0	
Theta range for data collection	7.848 to 150.122°.	
Index ranges	-6 ≤ h ≤ 7, -13 ≤ k ≤ 13, -27 ≤ l ≤ 27	
Reflections collected	36581	
Independent reflections	3101 [R(int) = 0.0463, R(sigma) = 0.0171]	
Data / restraints / parameters	3101/ 0 / 209	
Goodness-of-fit on F^2	1.075	
Final R indices [I>2sigma(I)]	R1 = 0.0270, wR2 = 0.0685	
R indices (all data)	R1 = 0.0277, wR2 = 0.0690	
Absolute structure parameter [Flack]	0.04 (5)	
Largest diff. peak and hole	0.12 and -0.18 e.$Å^{-3}$	

1.8 ^{1}H-NMR, ^{13}C-NMR and ^{19}F-NMR Spectra

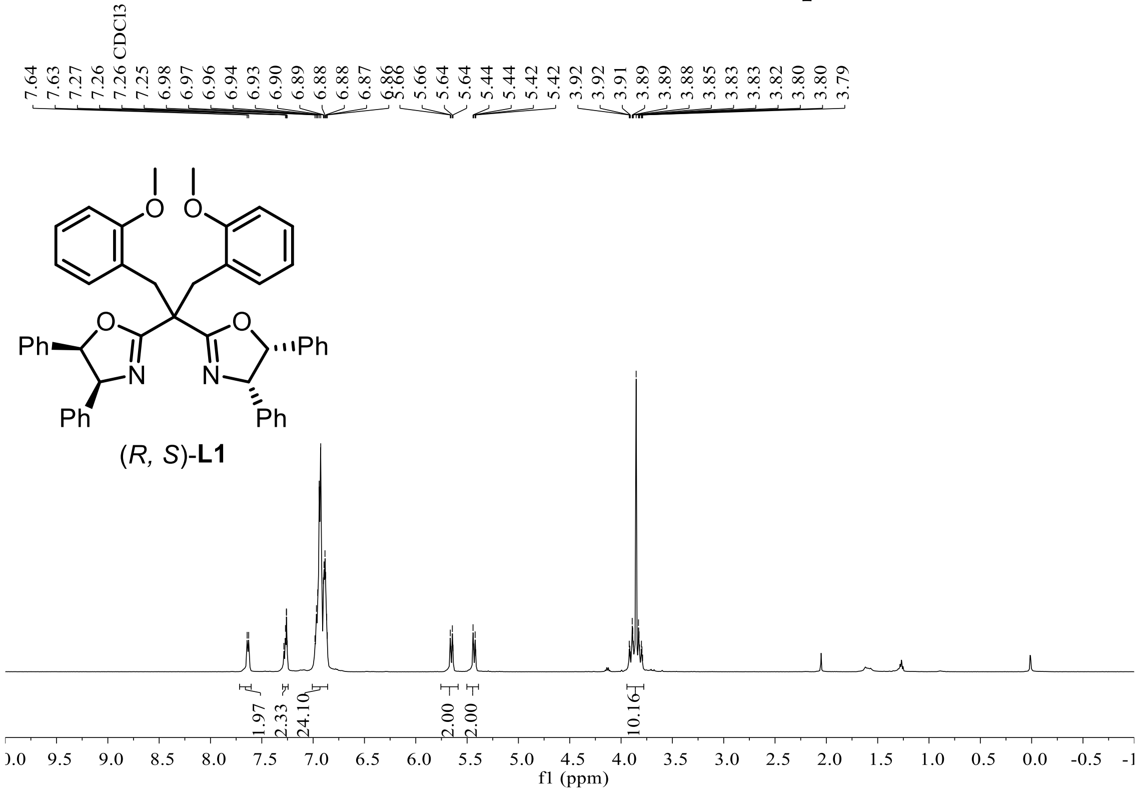

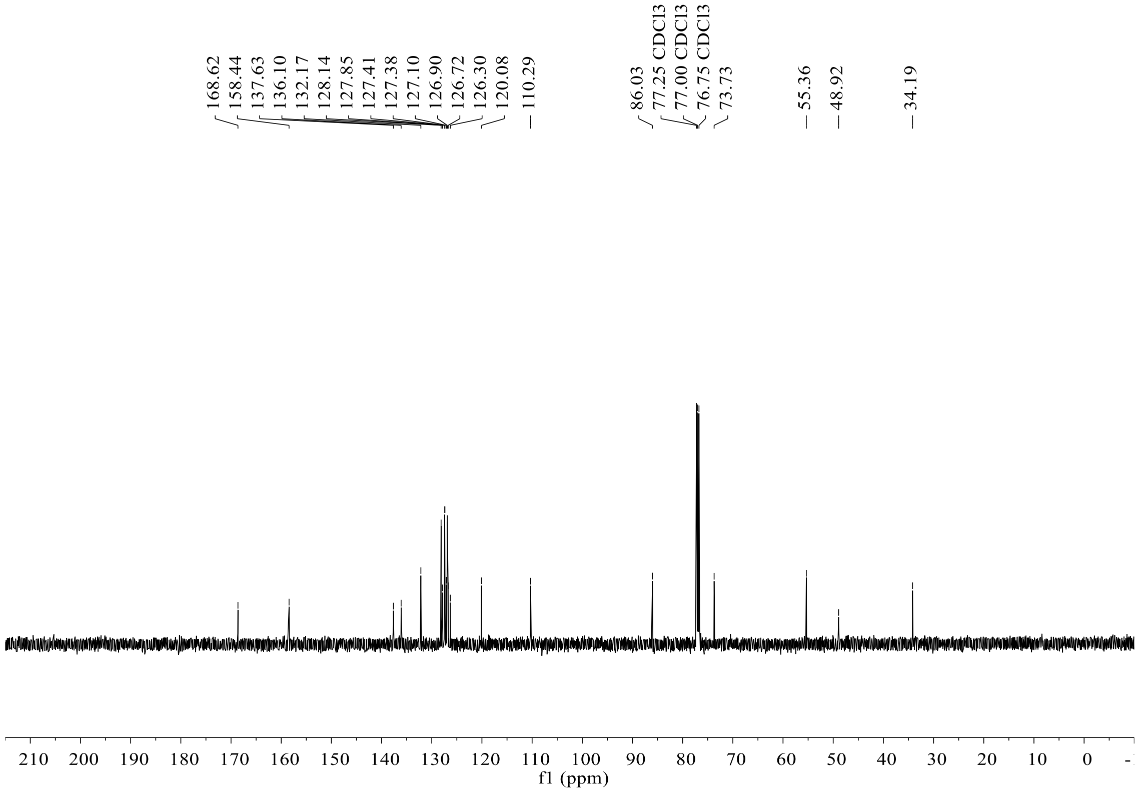

Supplementary Fig. 8 ^{1}H NMR (500 MHz, $CDCl_3$) and ^{13}C NMR (126 MHz, $CDCl_3$) spectrum of (*R*, *S*)-**L1**.

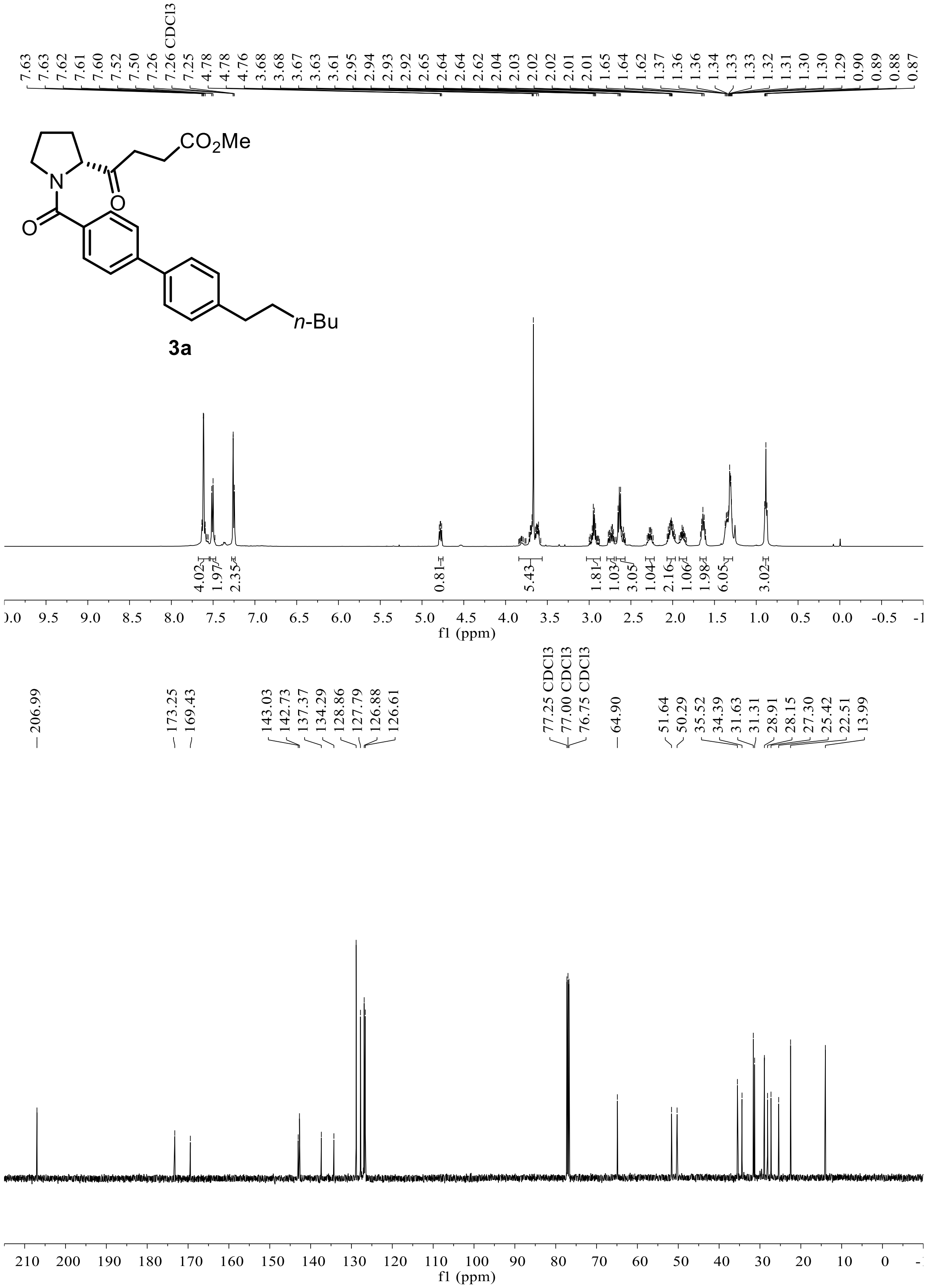

Supplementary Fig. 9 ^{1}H NMR (500 MHz, $CDCl_3$) and ^{13}C NMR (126 MHz, $CDCl_3$) spectrum of **3a**.

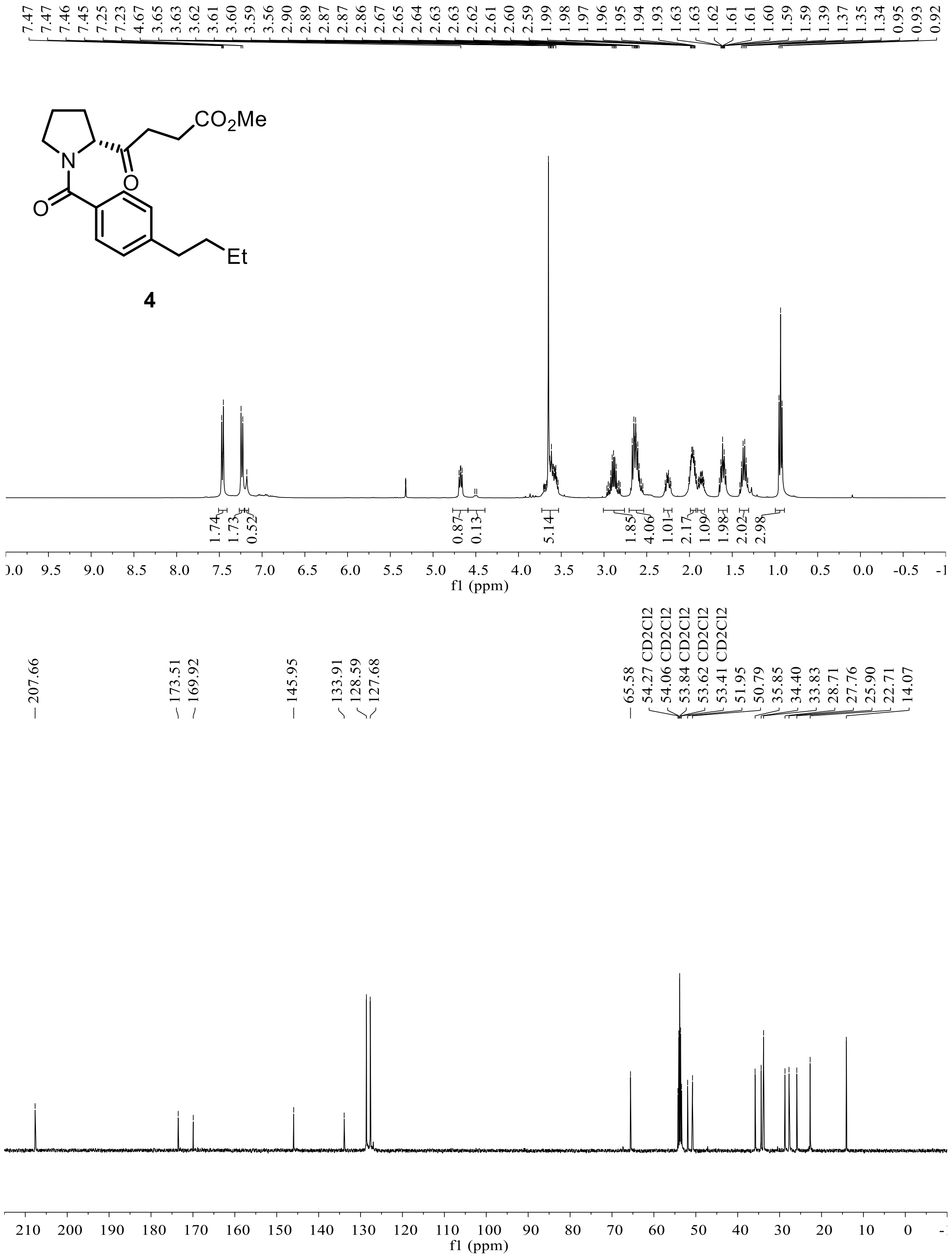

Supplementary Fig. 10 ^{1}H NMR (400 MHz, CD_2Cl_2) and ^{13}C NMR (126 MHz, CD_2Cl_2) spectrum of **4**.

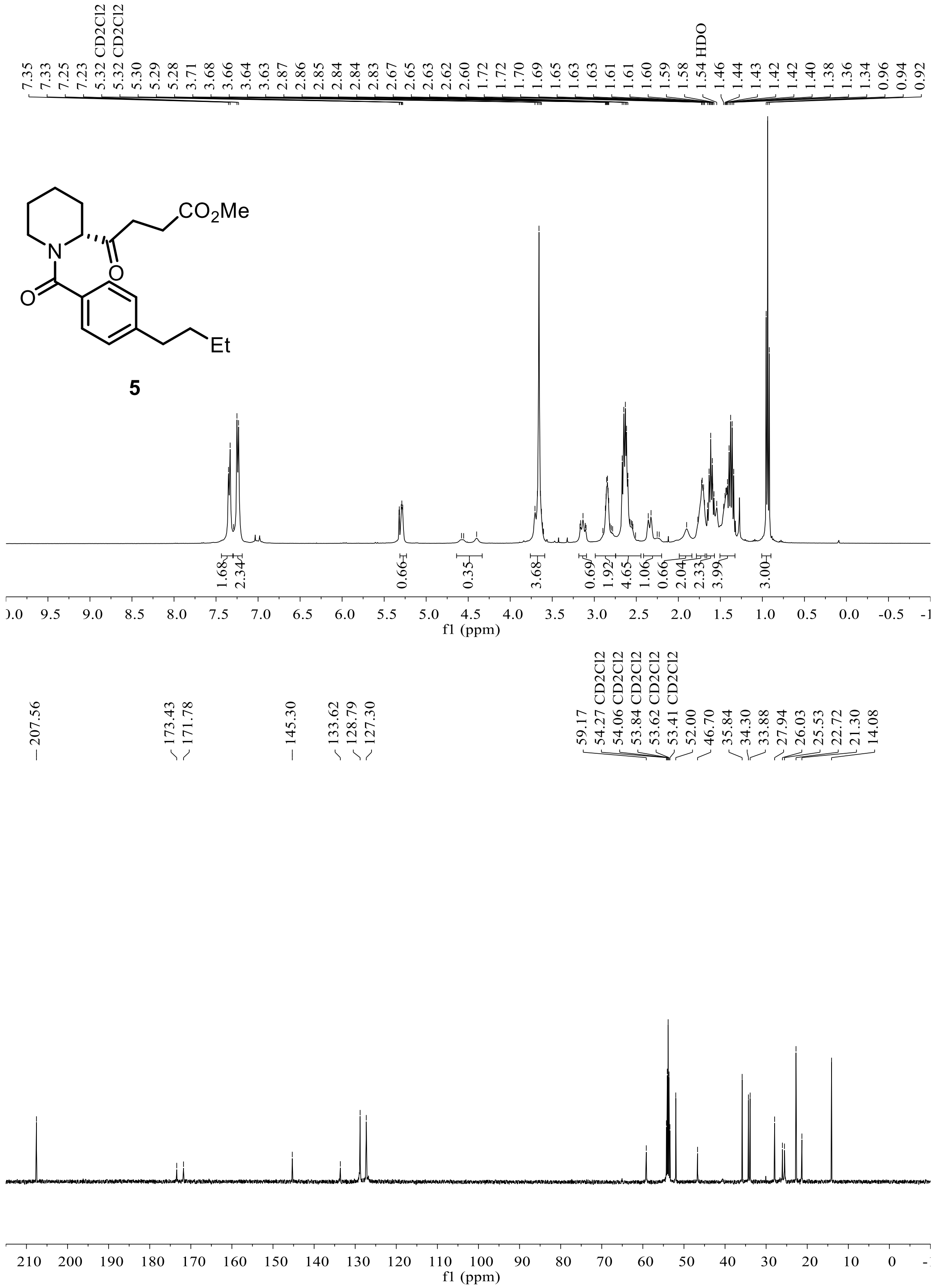

Supplementary Fig. 11 ^{1}H NMR (400 MHz, CD_2Cl_2) and ^{13}C NMR (126 MHz, CD_2Cl_2) spectrum of **5**.

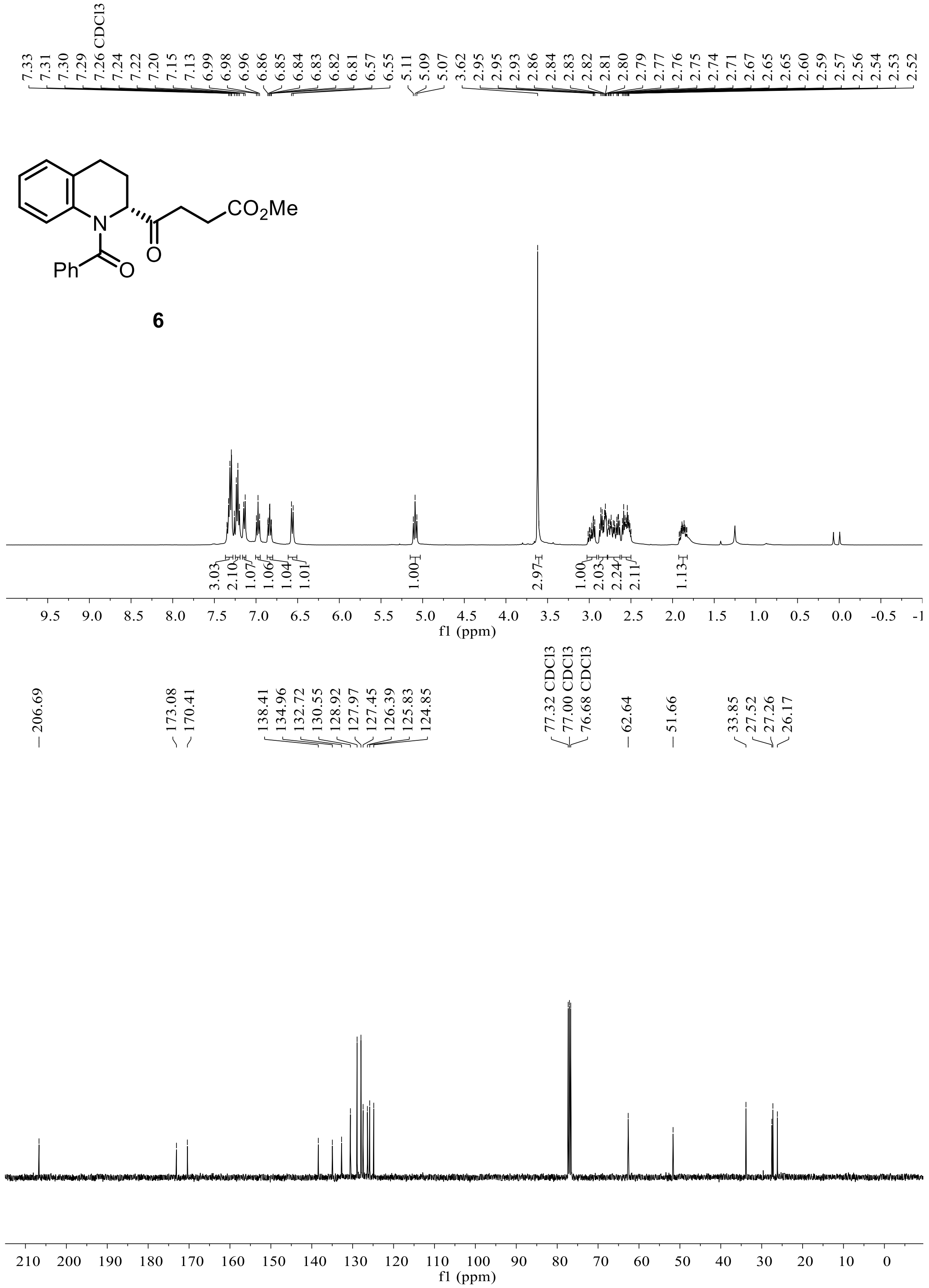

Supplementary Fig. 12 ^1H NMR (400 MHz, $CDCl_3$) and ^{13}C NMR (101 MHz, $CDCl_3$) spectrum of **6**.

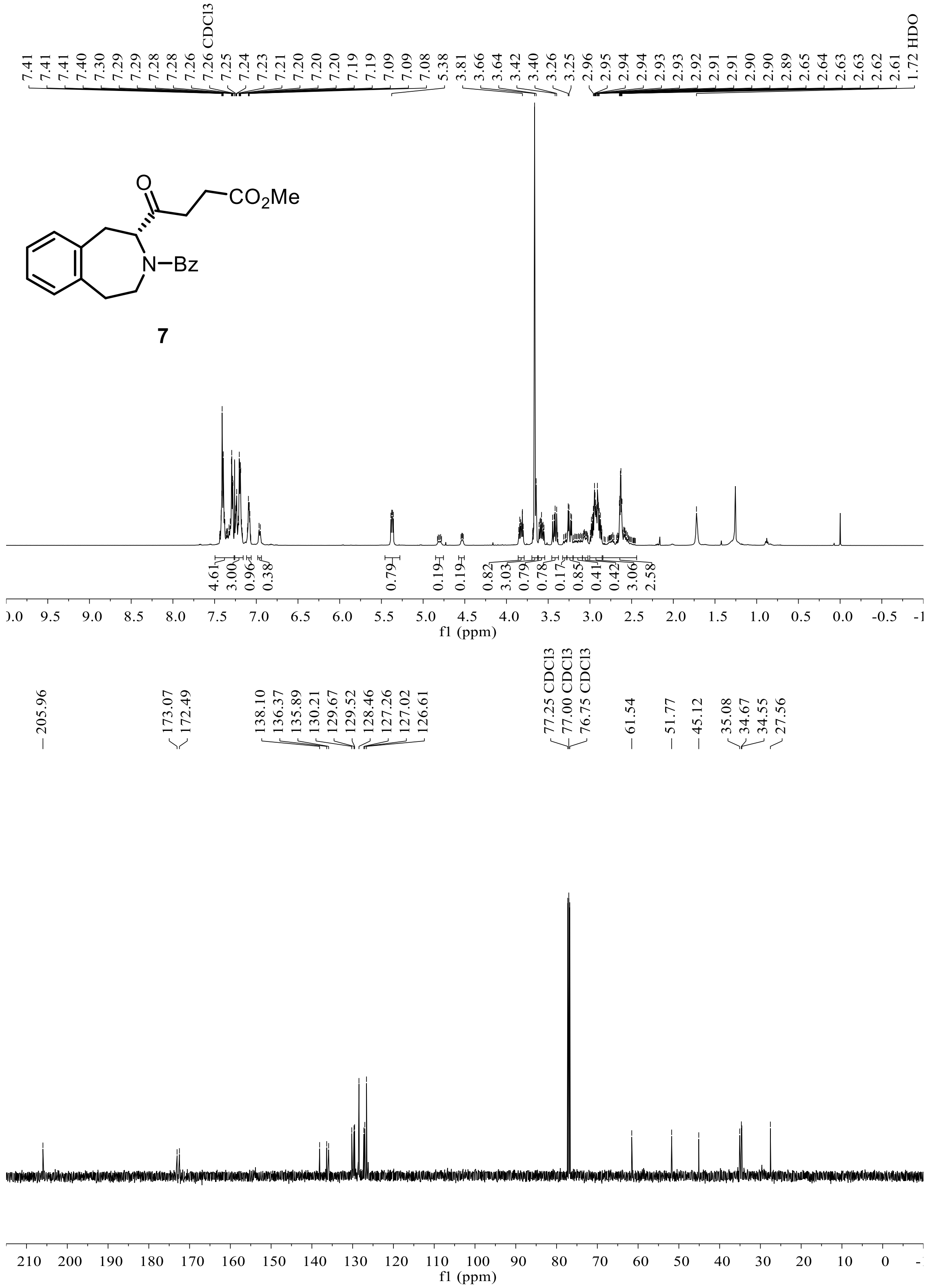

Supplementary Fig. 13 ¹H NMR (500 MHz, $CDCl_3$) and ¹³C NMR (126 MHz, $CDCl_3$) spectrum of **7**.

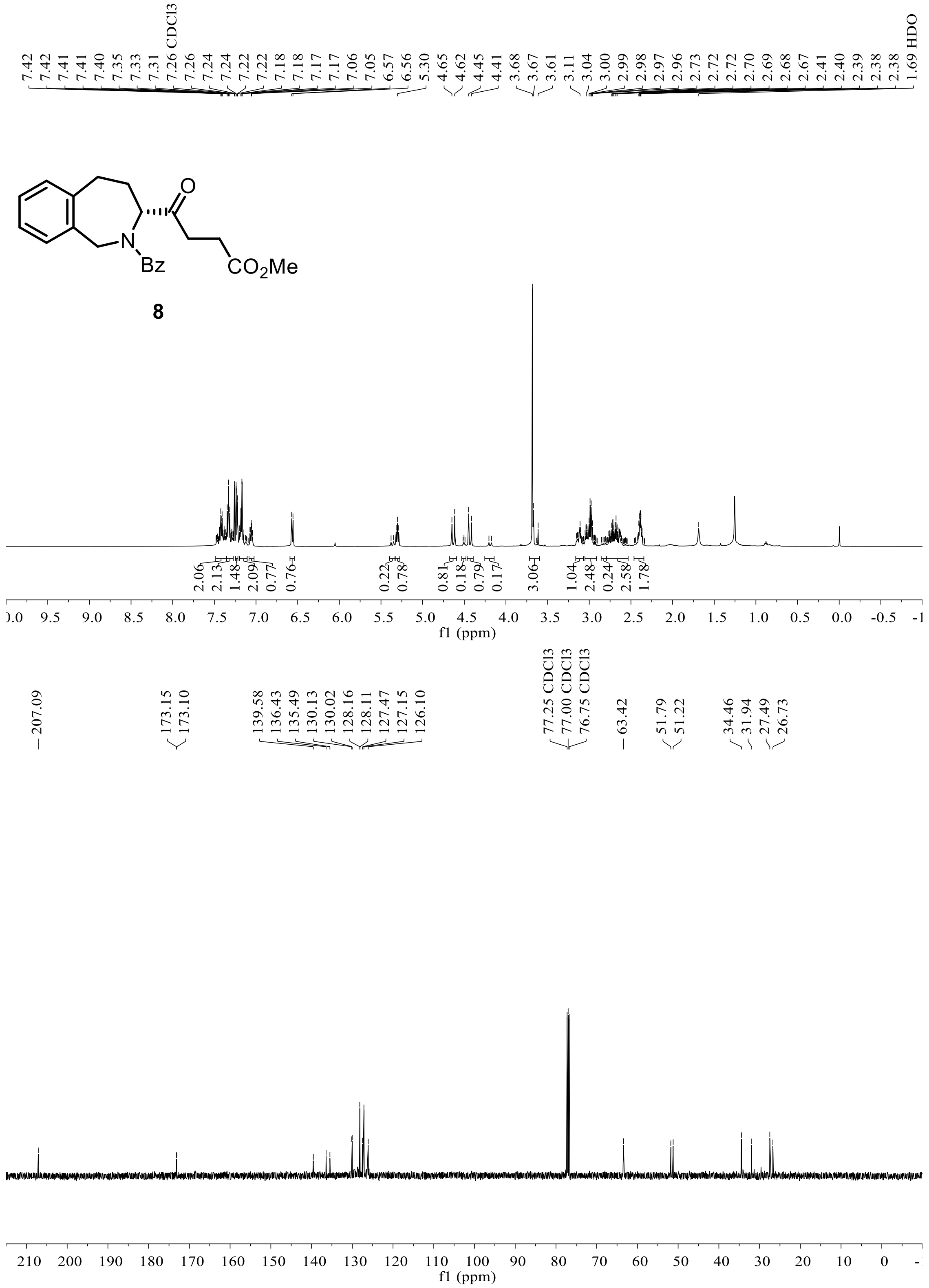

Supplementary Fig. 14 ^{1}H NMR (500 MHz, $CDCl_3$) and ^{13}C NMR (126 MHz, $CDCl_3$) spectrum of **8**.

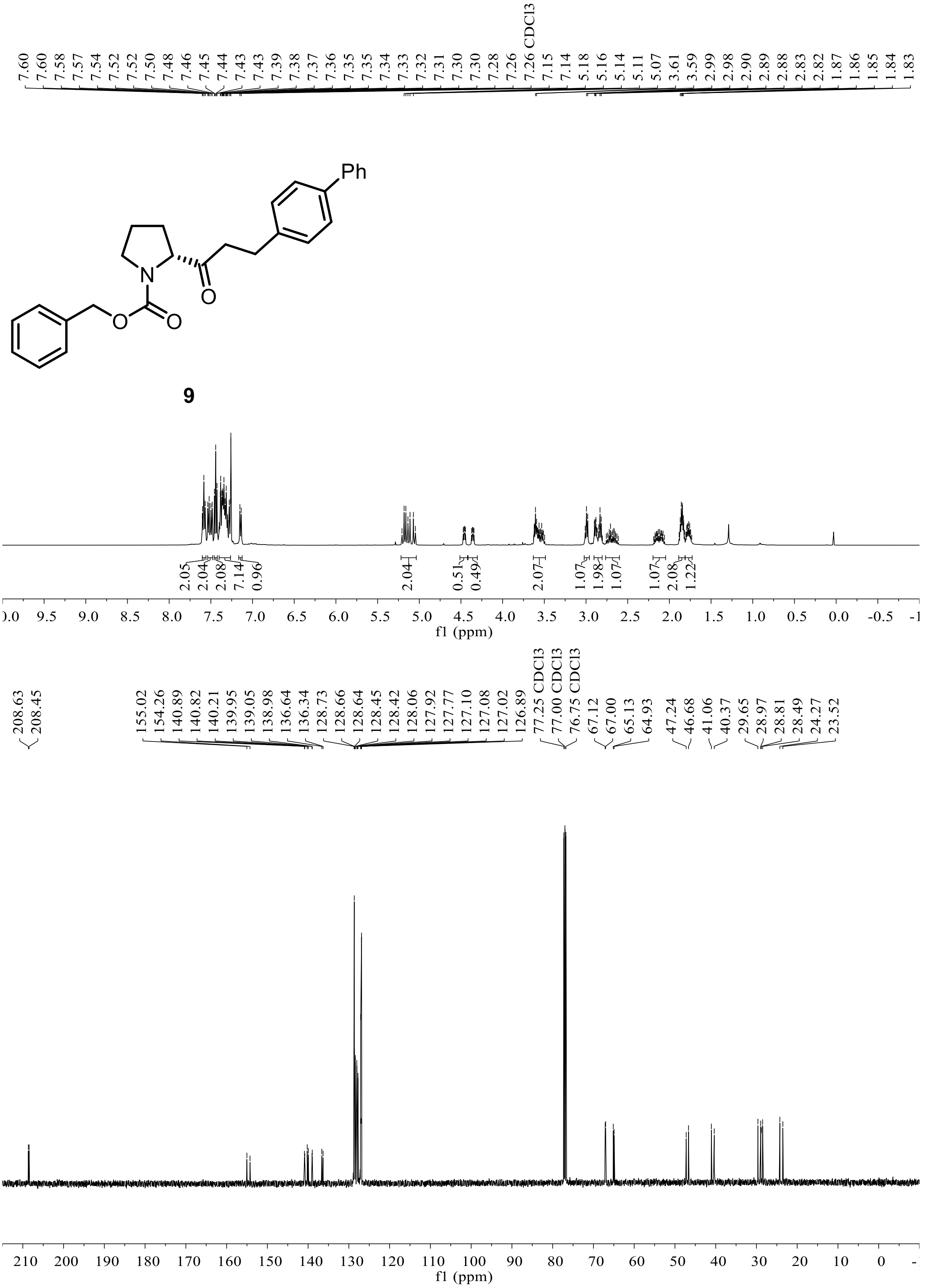

Supplementary Fig. 15 ^{1}H NMR (500 MHz, $CDCl_3$) and ^{13}C NMR (126 MHz, $CDCl_3$) spectrum of **9**.

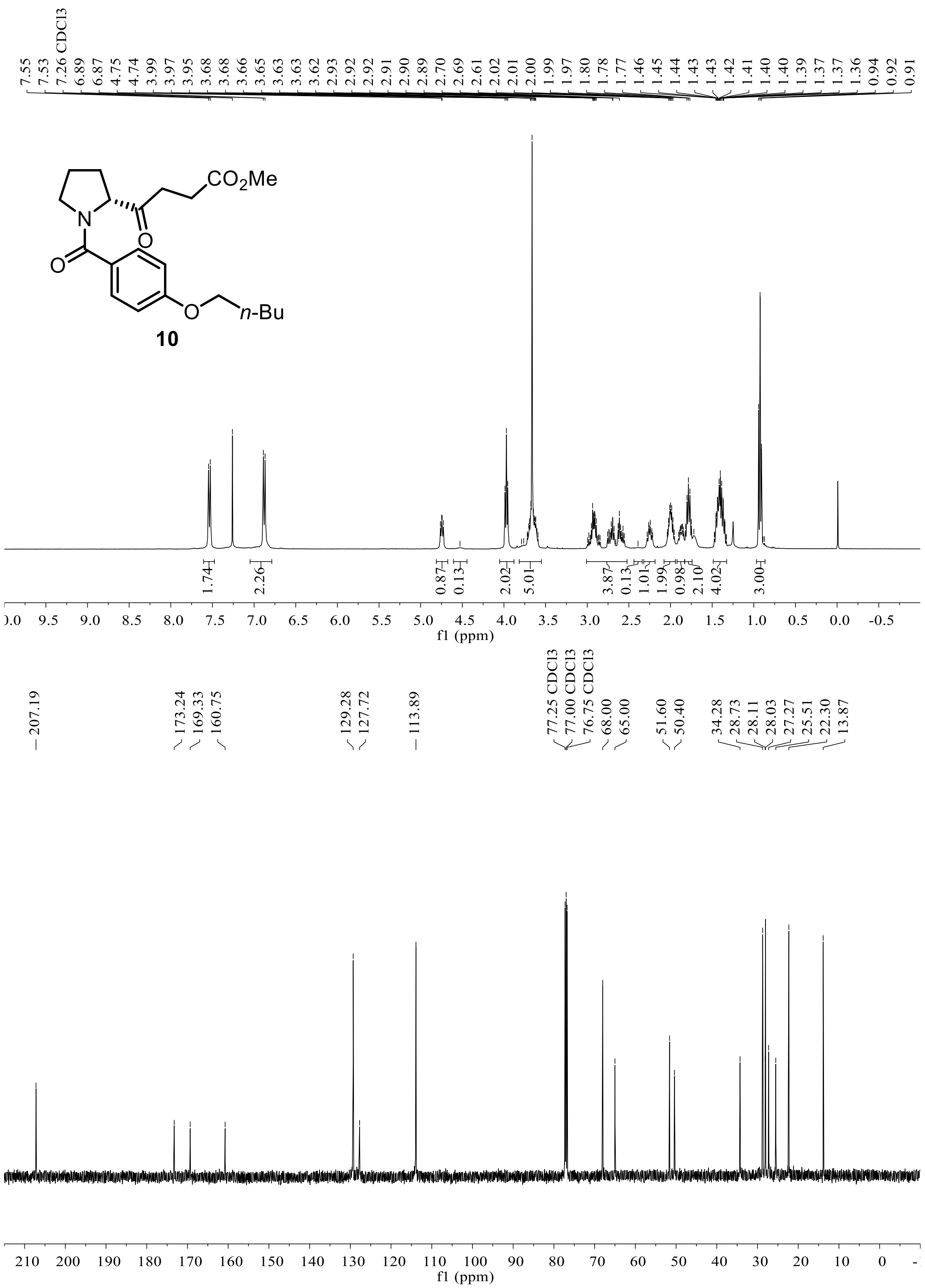

Supplementary Fig. 16 ^{1}H NMR (400 MHz, $CDCl_3$) and ^{13}C NMR (126 MHz, $CDCl_3$) spectrum of **10**.

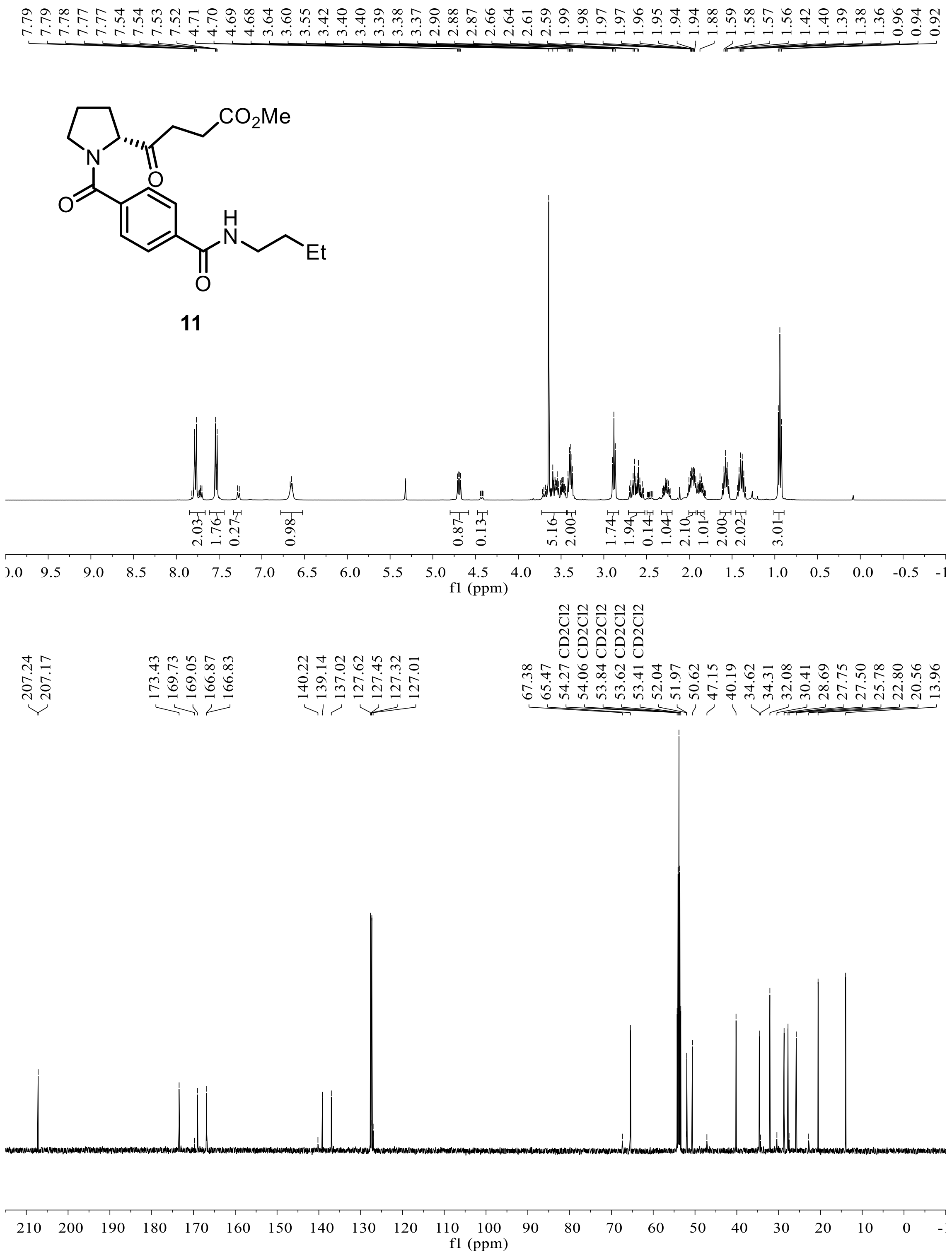

Supplementary Fig. 17 ^{1}H NMR (400 MHz, CD_2Cl_2) and ^{13}C NMR (126 MHz, CD_2Cl_2) spectrum of **11**.

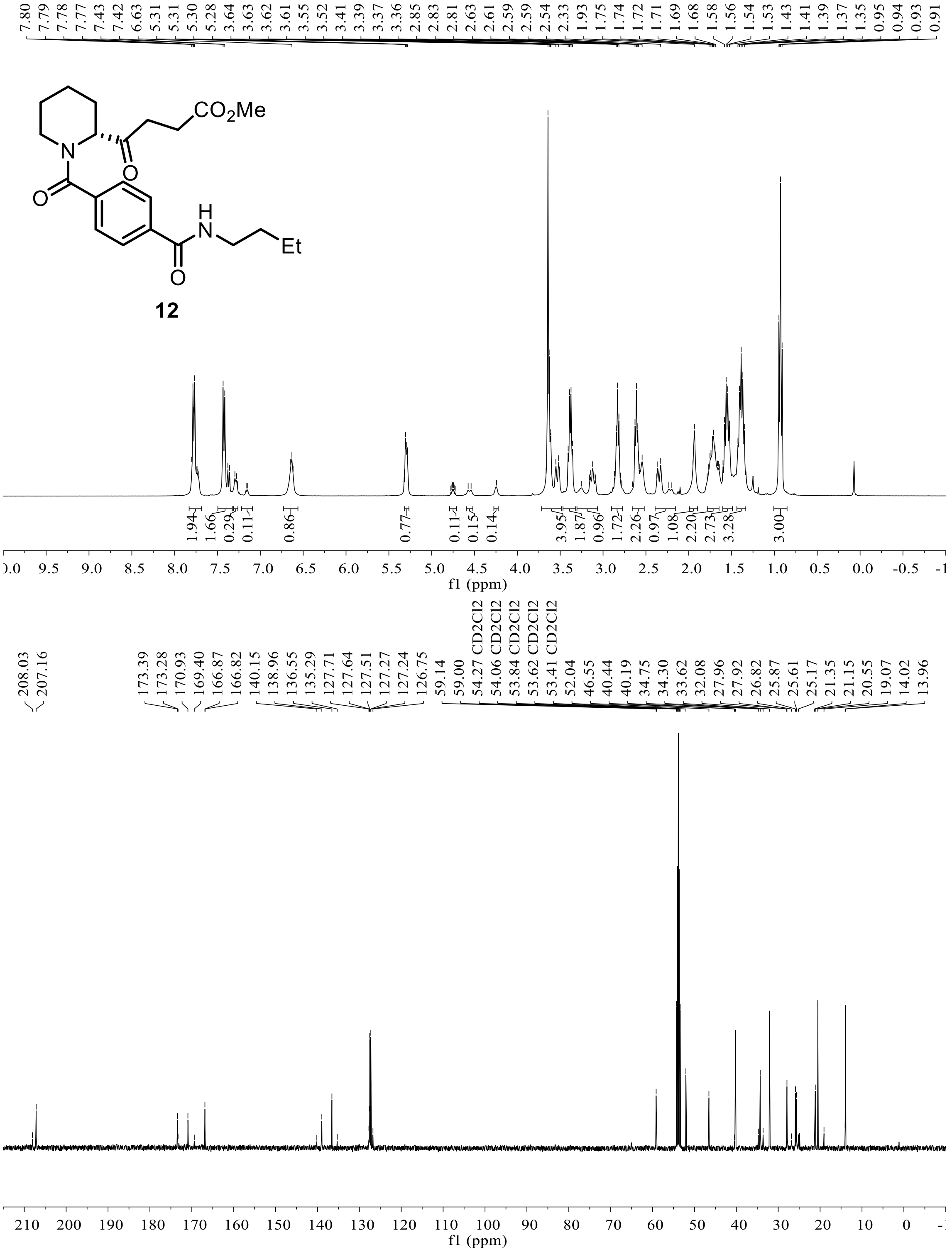

Supplementary Fig. 18 ^{1}H NMR (400 MHz, CD_2Cl_2) and ^{13}C NMR (126 MHz, CD_2Cl_2) spectrum of **12**.

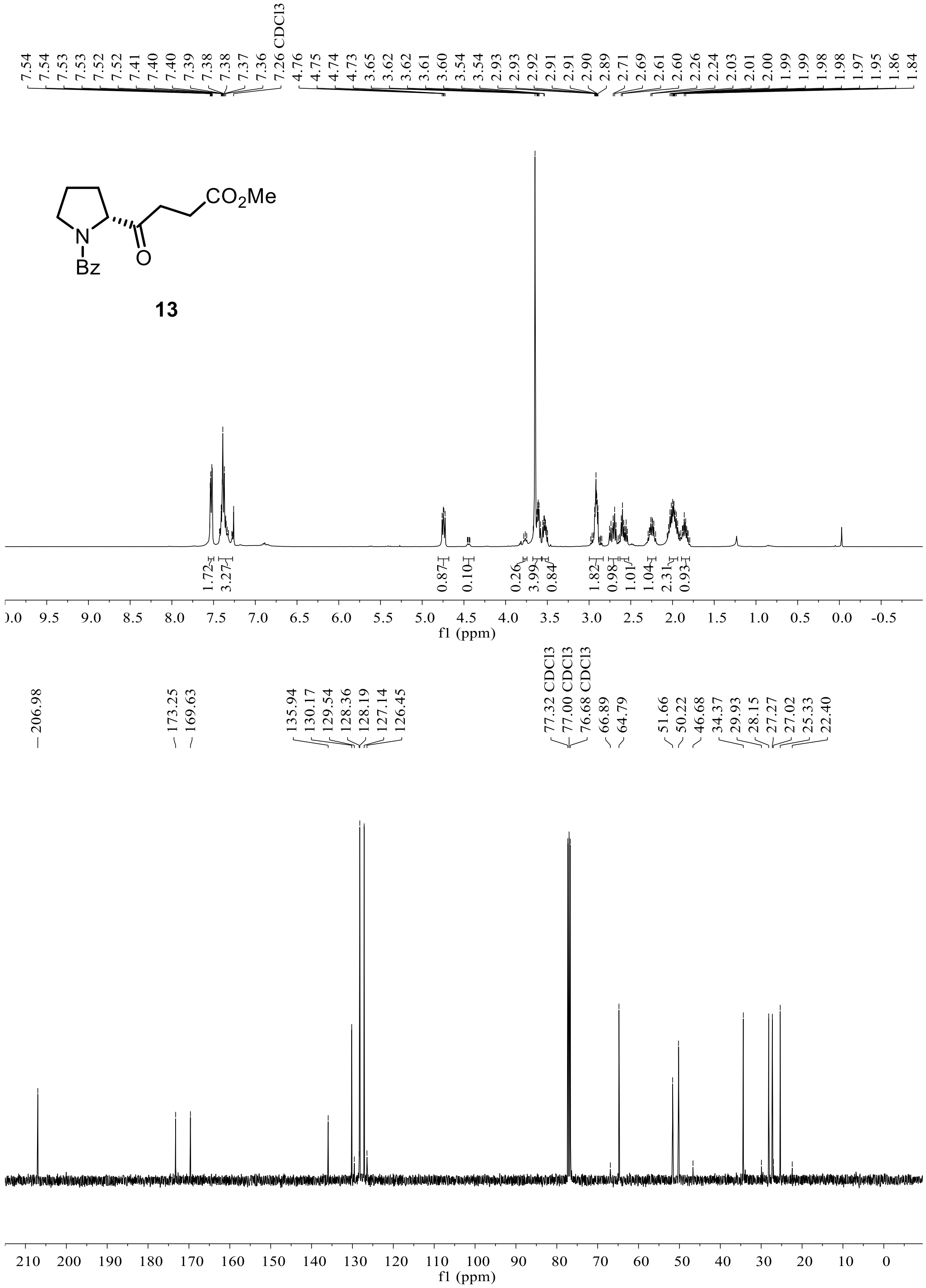

Supplementary Fig. 19 ^{1}H NMR (400 MHz, $CDCl_3$) and ^{13}C NMR (101 MHz, $CDCl_3$) spectrum of **13**.

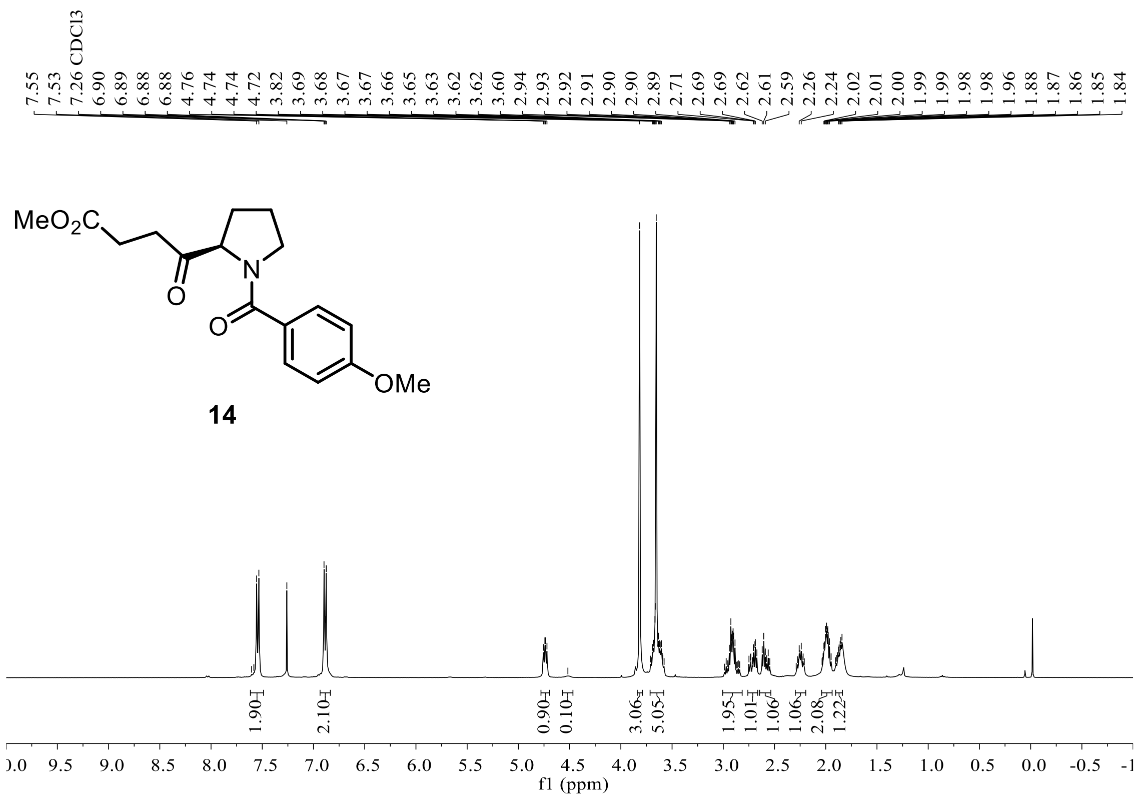

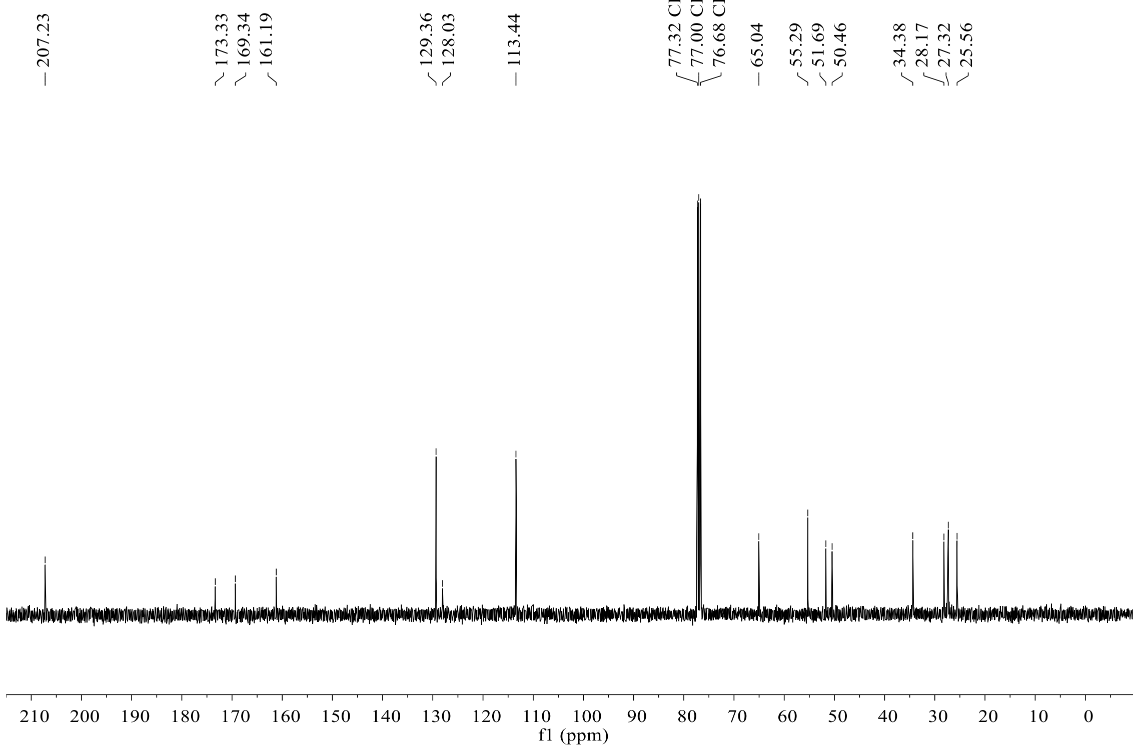

Supplementary Fig. 20 ^{1}H NMR (400 MHz, $CDCl_3$) and ^{13}C NMR (101 MHz, $CDCl_3$) spectrum of **14**.

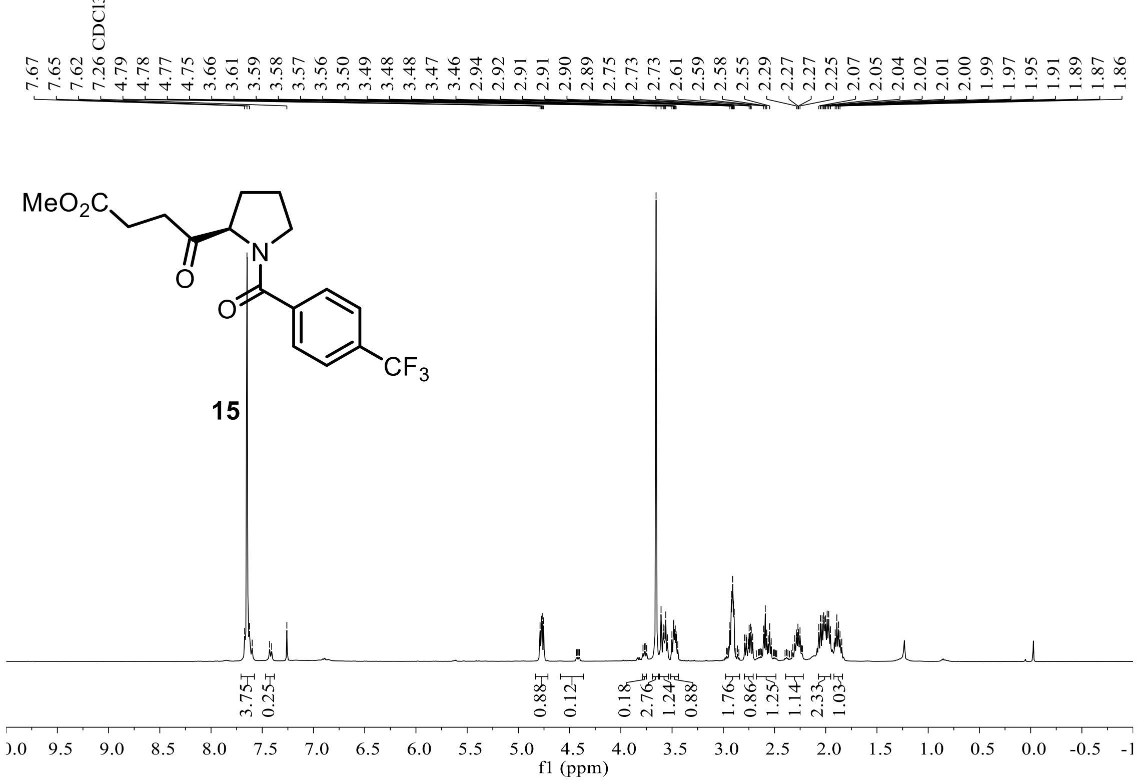
7.67 7.65 7.62 7.26 CDCl3 4.79 4.78 4.77 4.75 3.66 3.61 3.59 3.58 3.57 3.56 3.50 3.49 3.48 3.48 3.47 3.46 2.94 2.92 2.91 2.91 2.90 2.89 2.75 2.73 2.73 2.61 2.59 2.58 2.55 2.29 2.27 2.27 2.25 2.07 2.05 2.04 2.02 2.01 2.00 1.99 1.97 1.95 1.91 1.89 1.87 1.86
MeO2C
O
N
O
CF3
15
3.75 0.25 0.88 0.12 0.18 2.76 1.24 0.88 1.76 0.86 1.25 1.14 2.33 1.03
f1 (ppm)

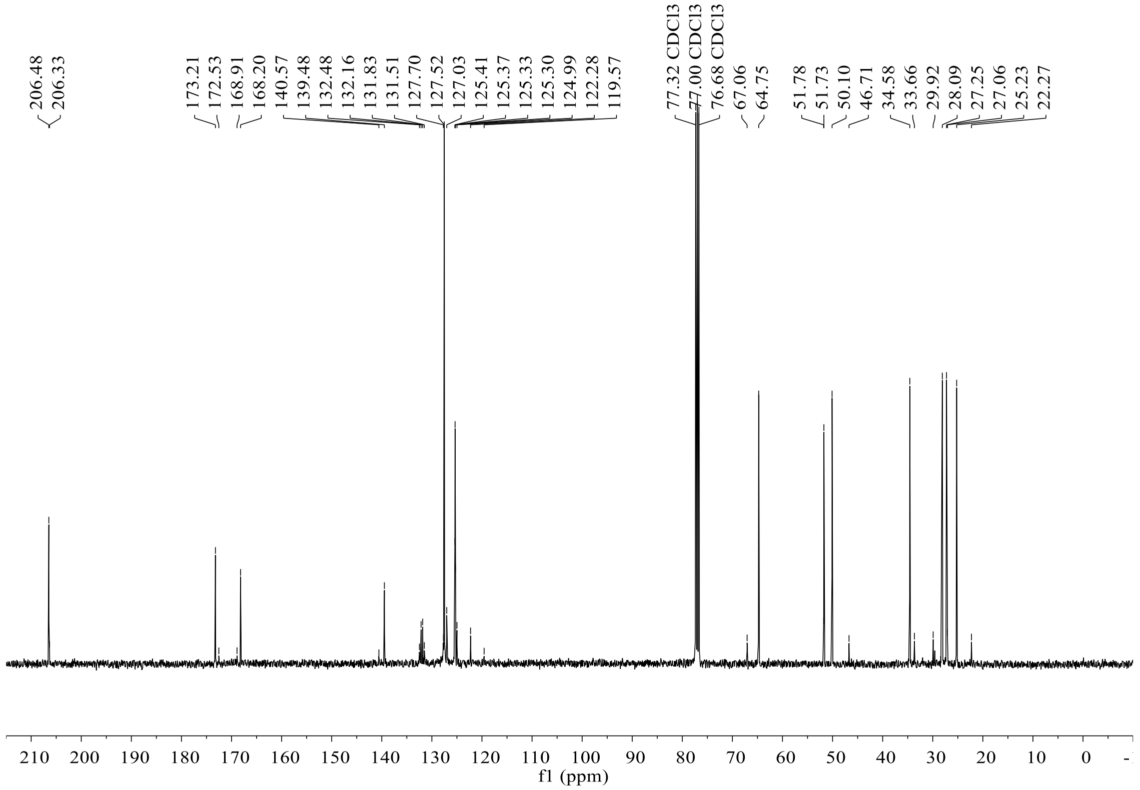
206.48 206.33 173.21 172.53 168.91 168.20 140.57 139.48 132.48 132.16 131.83 131.51 127.70 127.52 127.03 125.41 125.37 125.33 125.30 124.99 122.28 119.57 77.32 CDCl3 77.00 CDCl3 76.68 CDCl3 67.06 64.75 51.78 51.73 50.10 46.71 34.58 33.66 29.92 28.09 27.25 27.06 25.23 22.27
f1 (ppm)

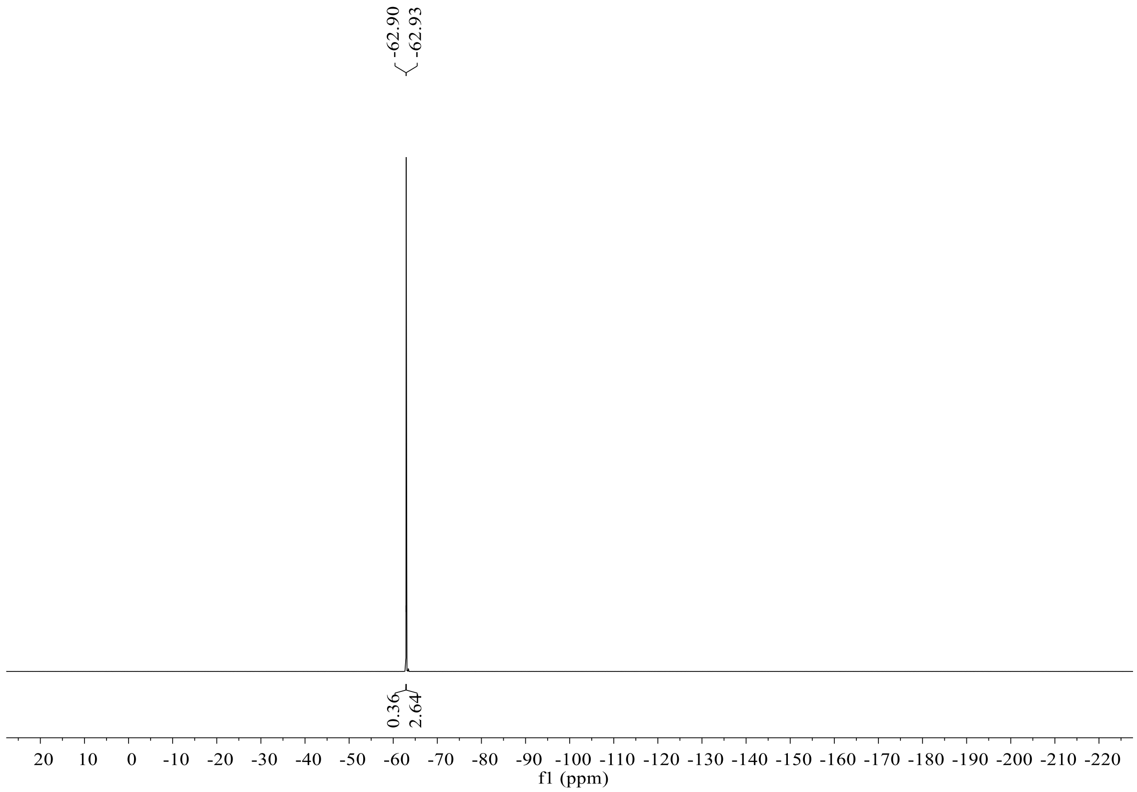

Supplementary Fig. 21 ^{1}H NMR (400 MHz, $CDCl_3$), ^{13}C NMR (101 MHz, $CDCl_3$) and ^{19}F NMR (376 MHz, $CDCl_3$) spectrum of **15**.

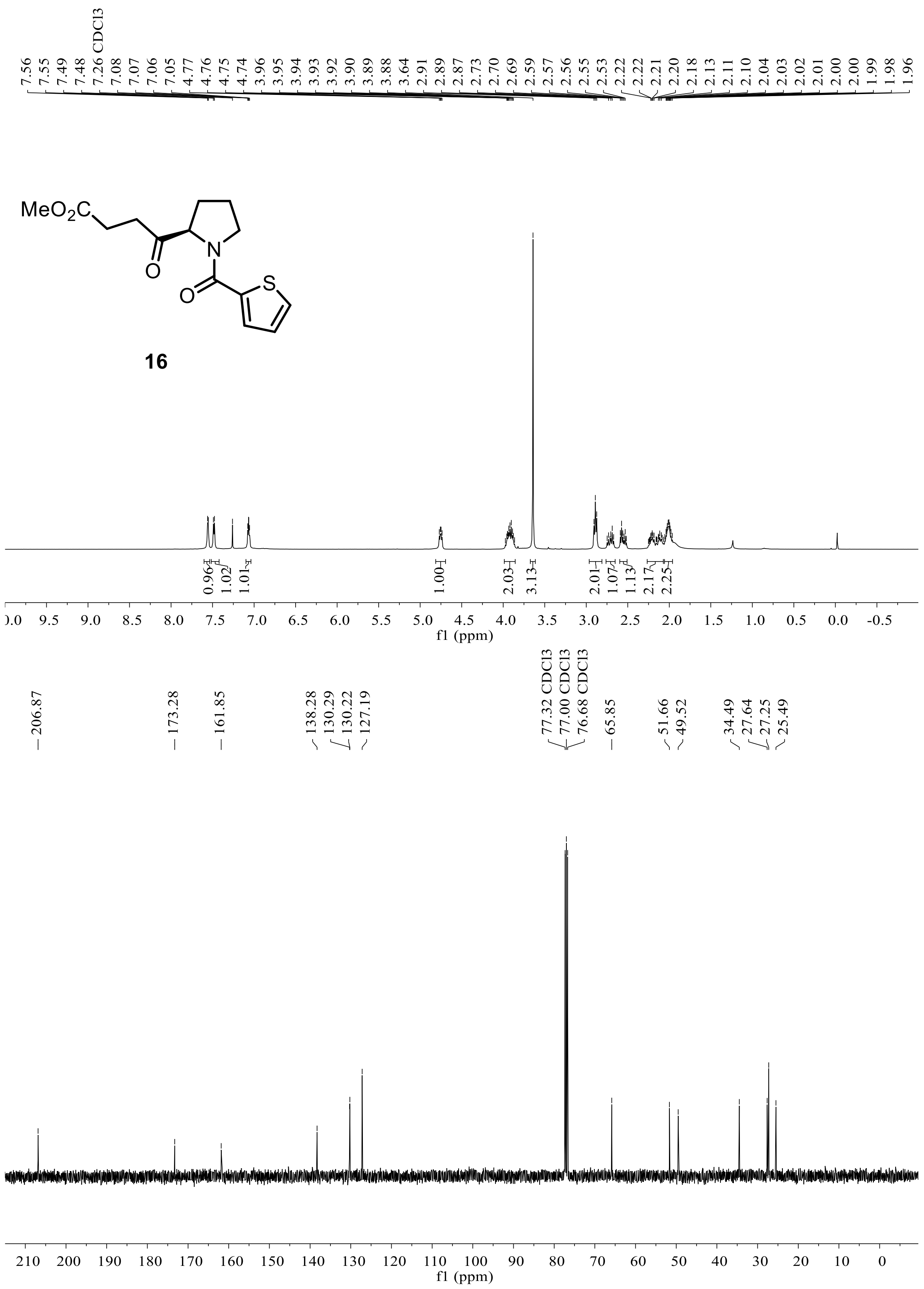

Supplementary Fig. 22 ^{1}H NMR (400 MHz, $CDCl_3$) and ^{13}C NMR (101 MHz, $CDCl_3$) spectrum of **16**.

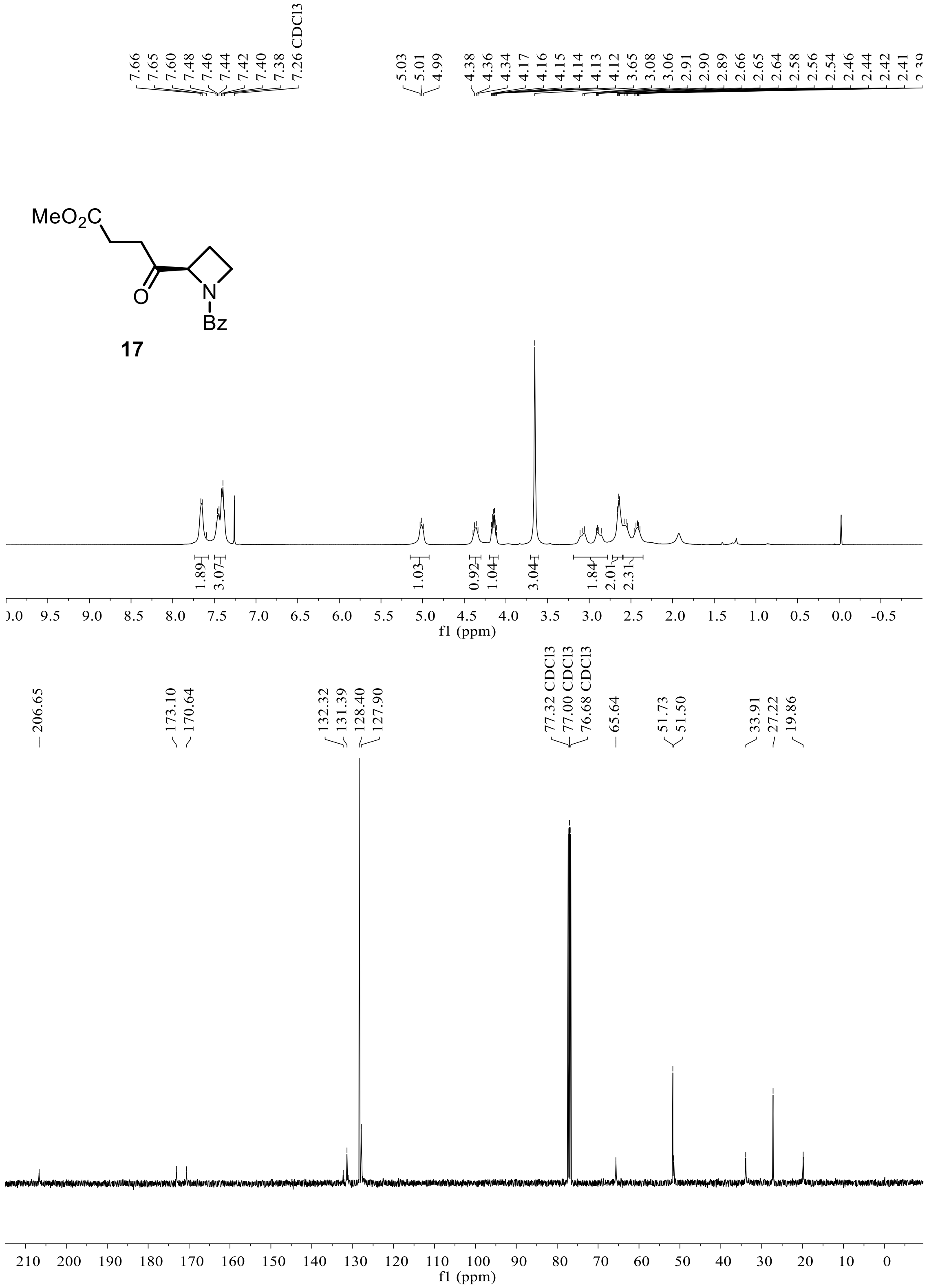

Supplementary Fig. 23 ^{1}H NMR (400 MHz, $CDCl_3$) and ^{13}C NMR (101 MHz, $CDCl_3$) spectrum of **17**.

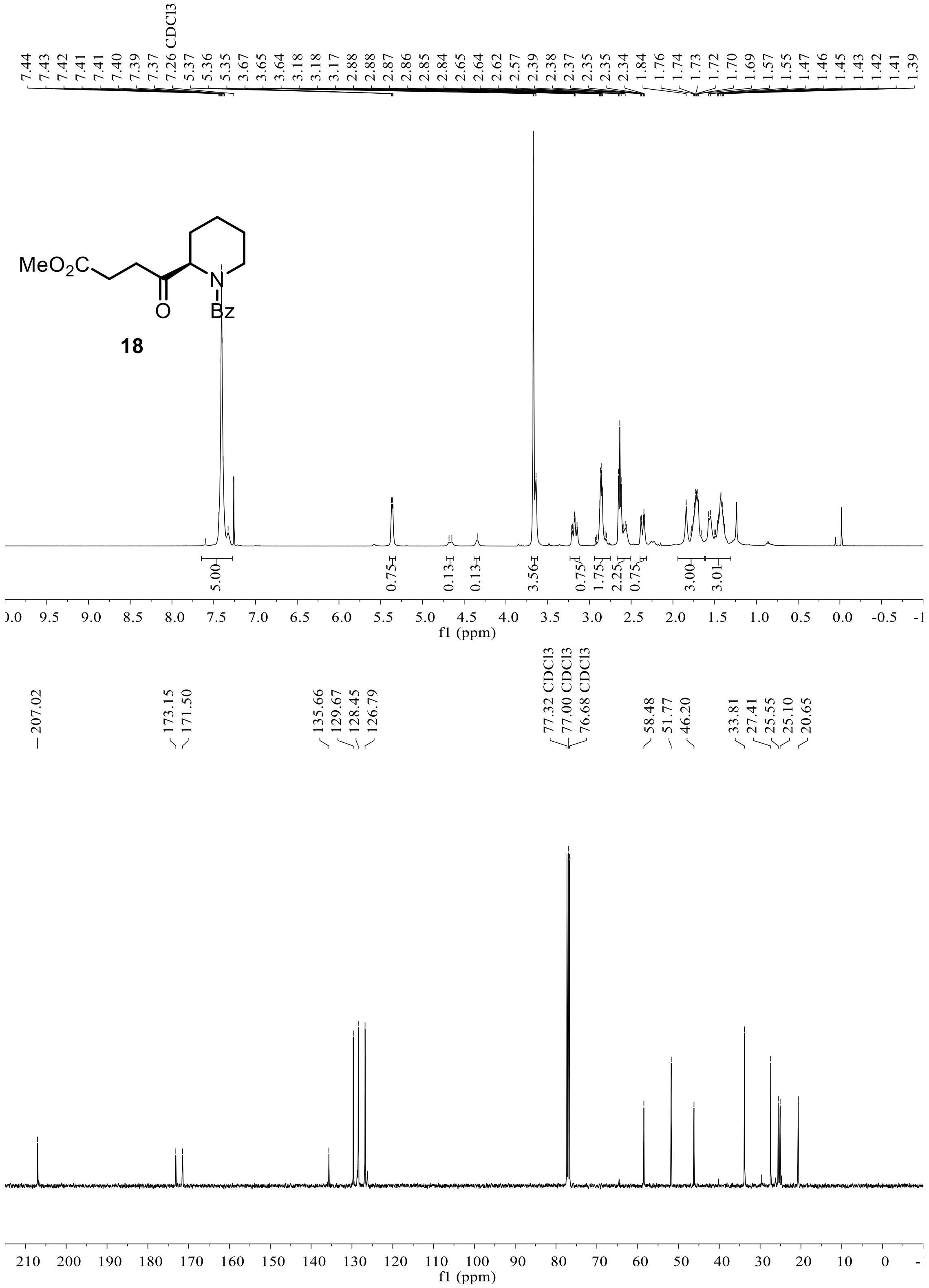

Supplementary Fig. 24 ^{1}H NMR (400 MHz, $CDCl_3$) and ^{13}C NMR (101 MHz, $CDCl_3$) spectrum of **18**.

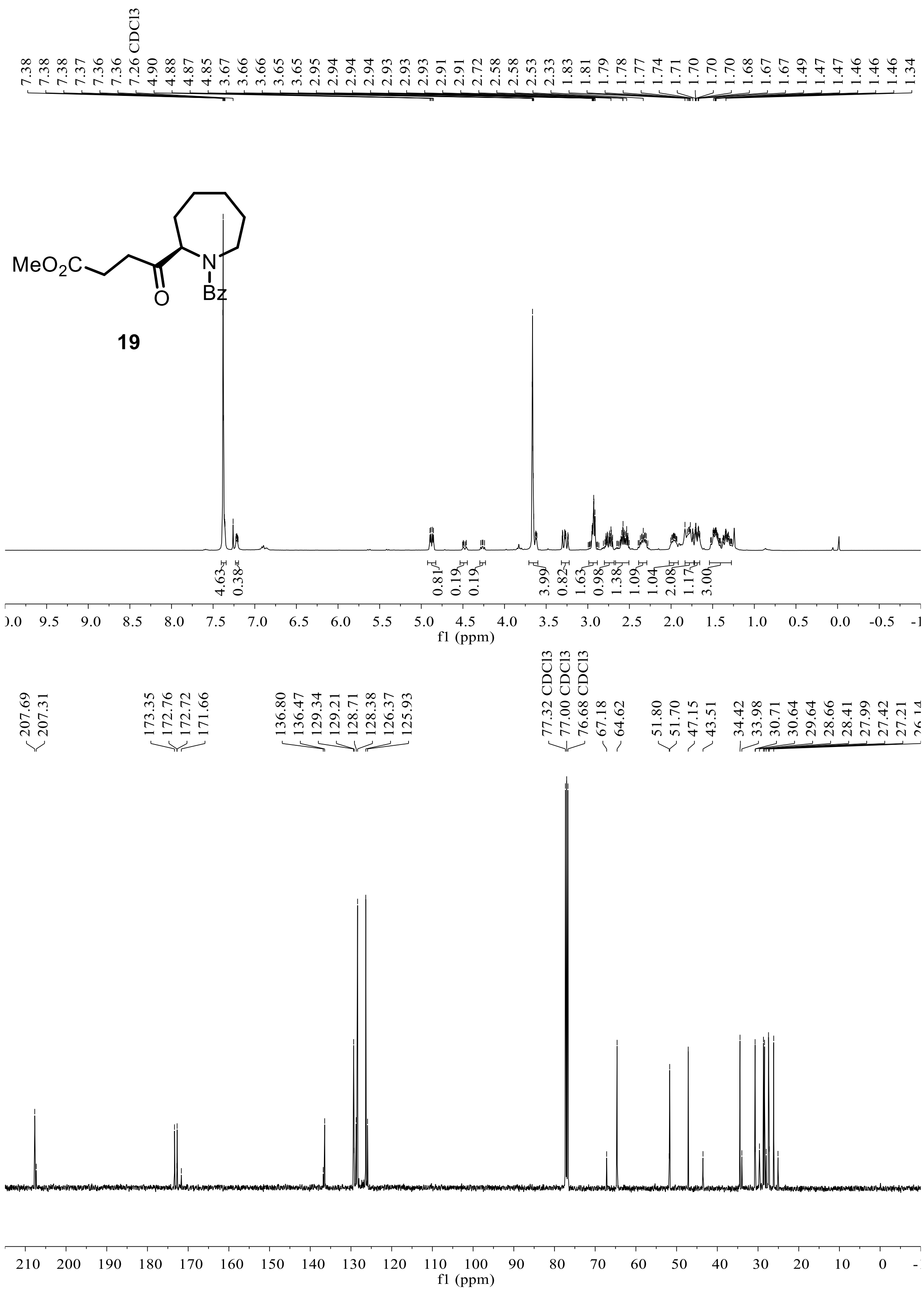

Supplementary Fig. 25 ^{1}H NMR (400 MHz, $CDCl_3$) and ^{13}C NMR (101 MHz, $CDCl_3$) spectrum of **19**.

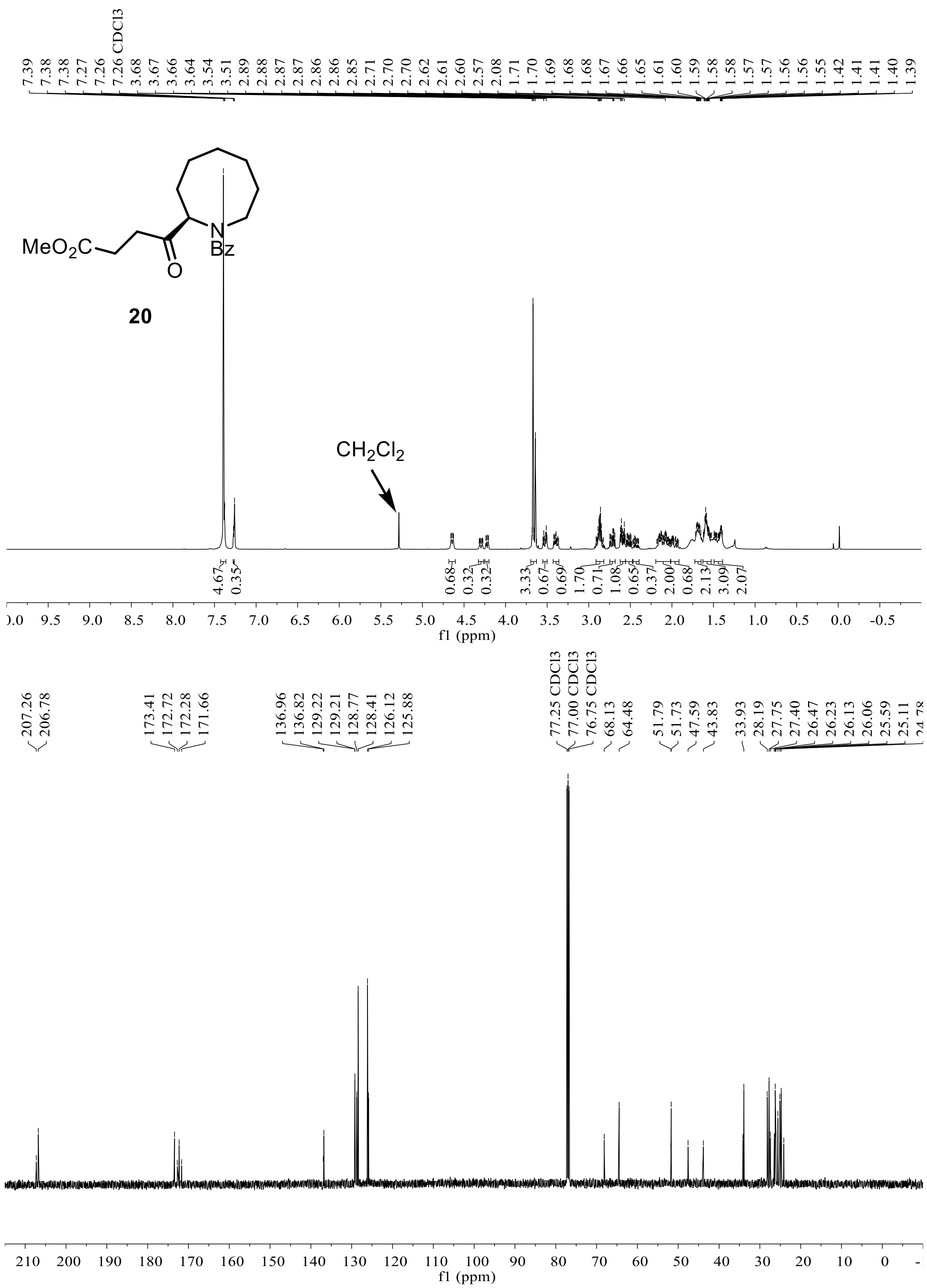

Supplementary Fig. 26 ^1H NMR (500 MHz, $CDCl_3$) and ^{13}C NMR (126 MHz, $CDCl_3$) spectrum of **20**.

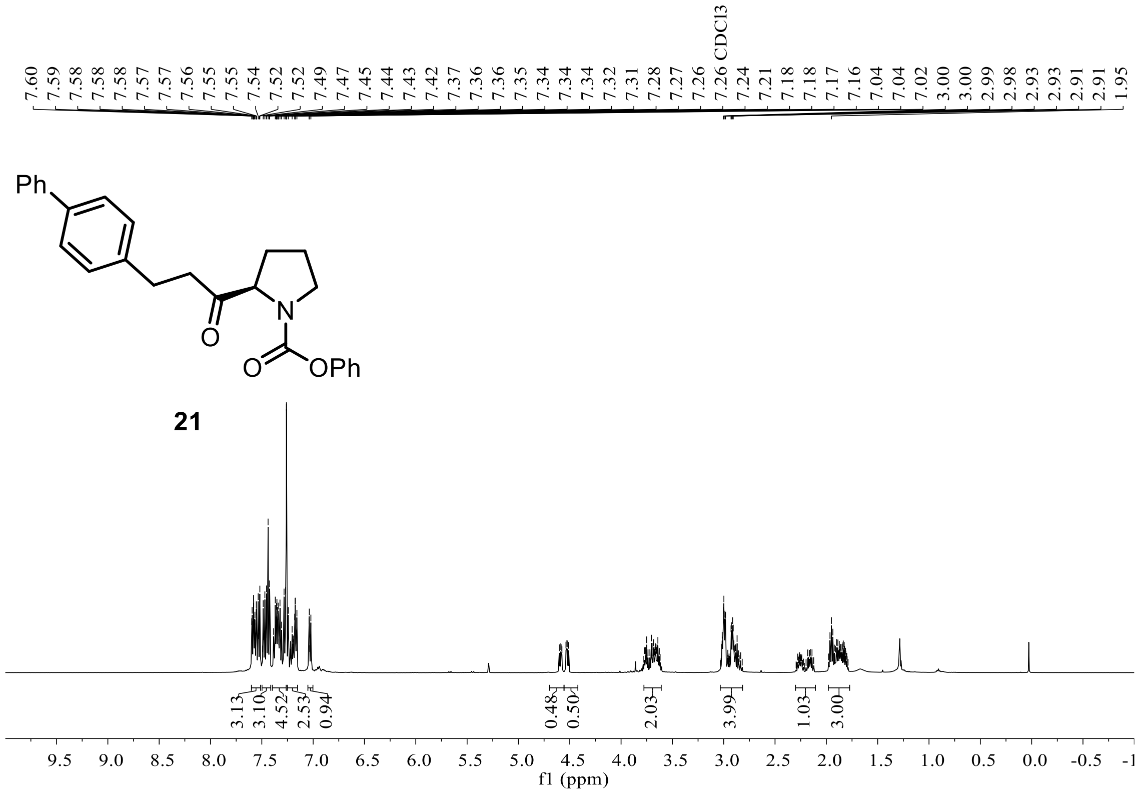

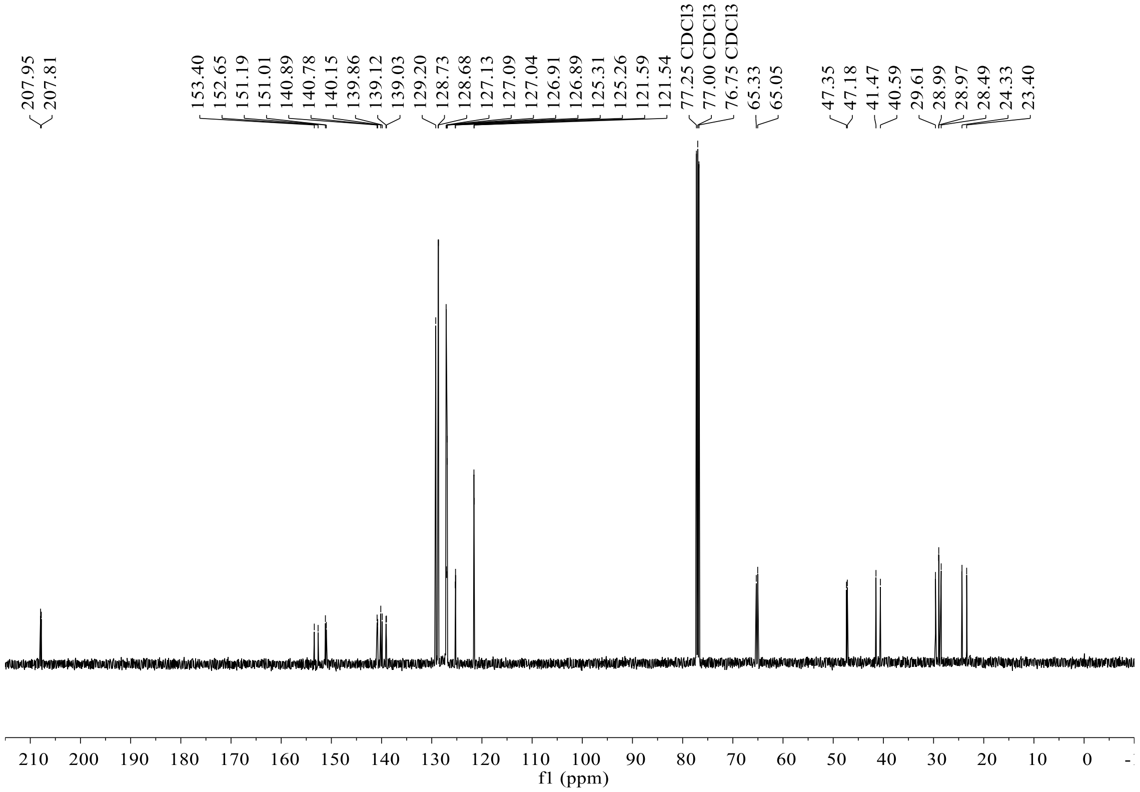

Supplementary Fig. 27 ^{1}H NMR (500 MHz, $CDCl_3$) and ^{13}C NMR (126 MHz, $CDCl_3$) spectrum of **21**.

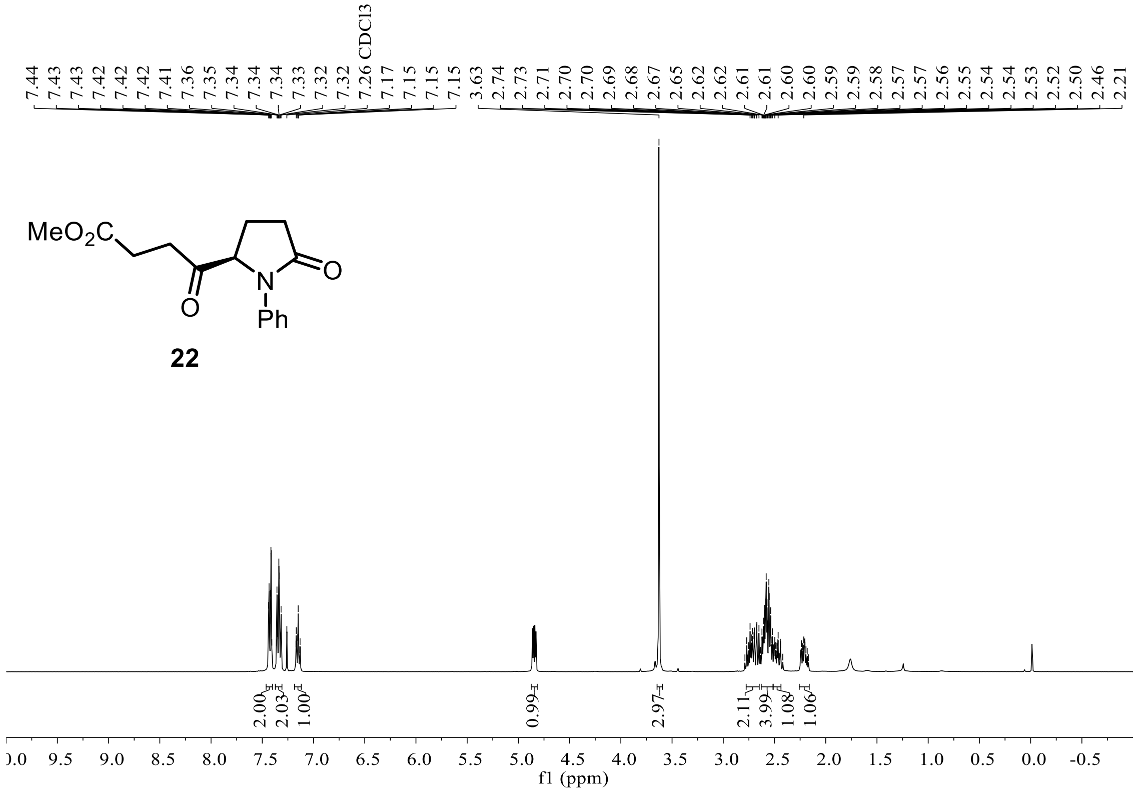

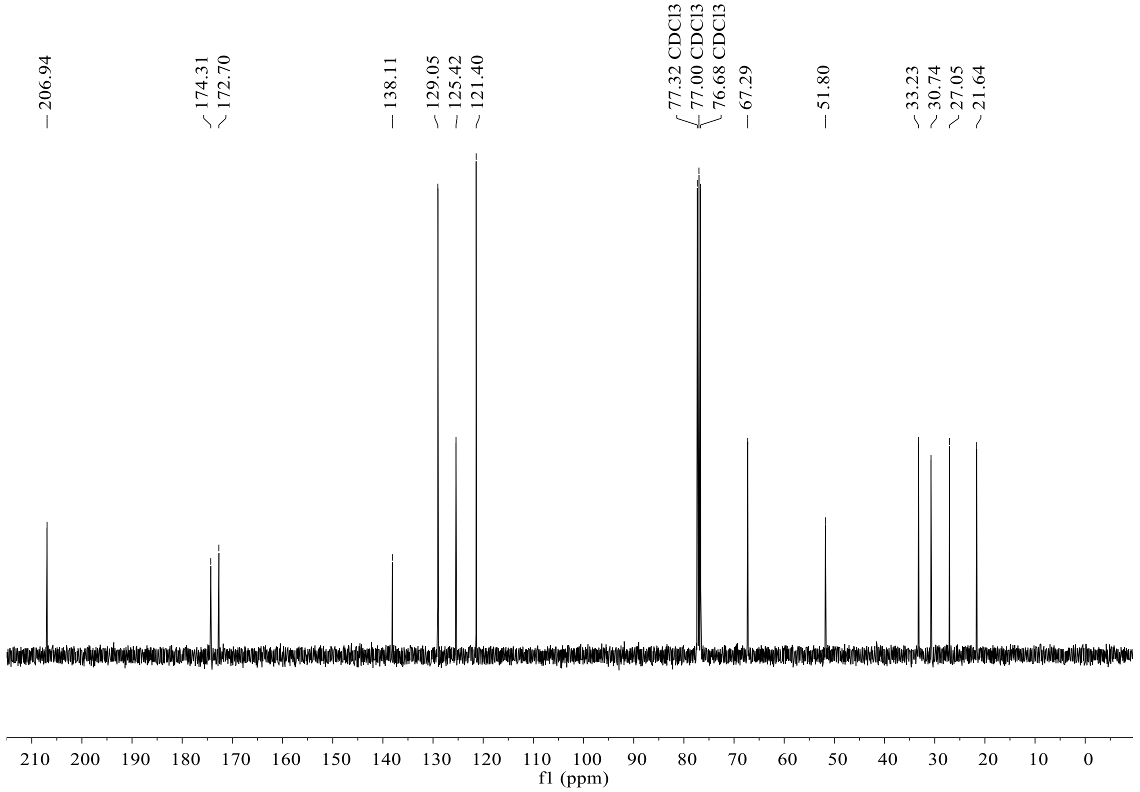

Supplementary Fig. 28 ^{1}H NMR (400 MHz, $CDCl_3$) and ^{13}C NMR (101 MHz, $CDCl_3$) spectrum of **22**.

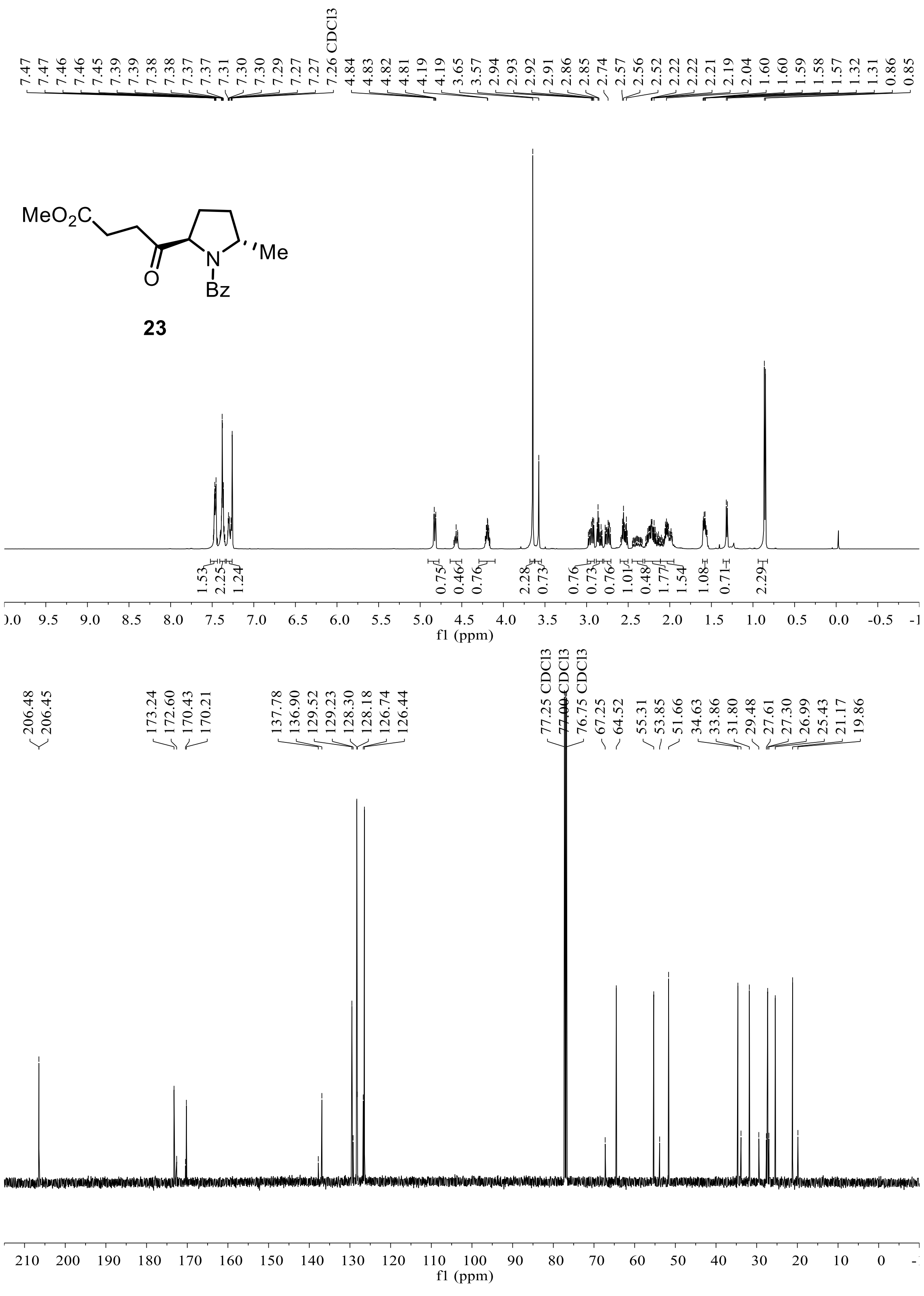

Supplementary Fig. 29 ^{1}H NMR (500 MHz, $CDCl_3$) and ^{13}C NMR (126 MHz, $CDCl_3$) spectrum of **23**.

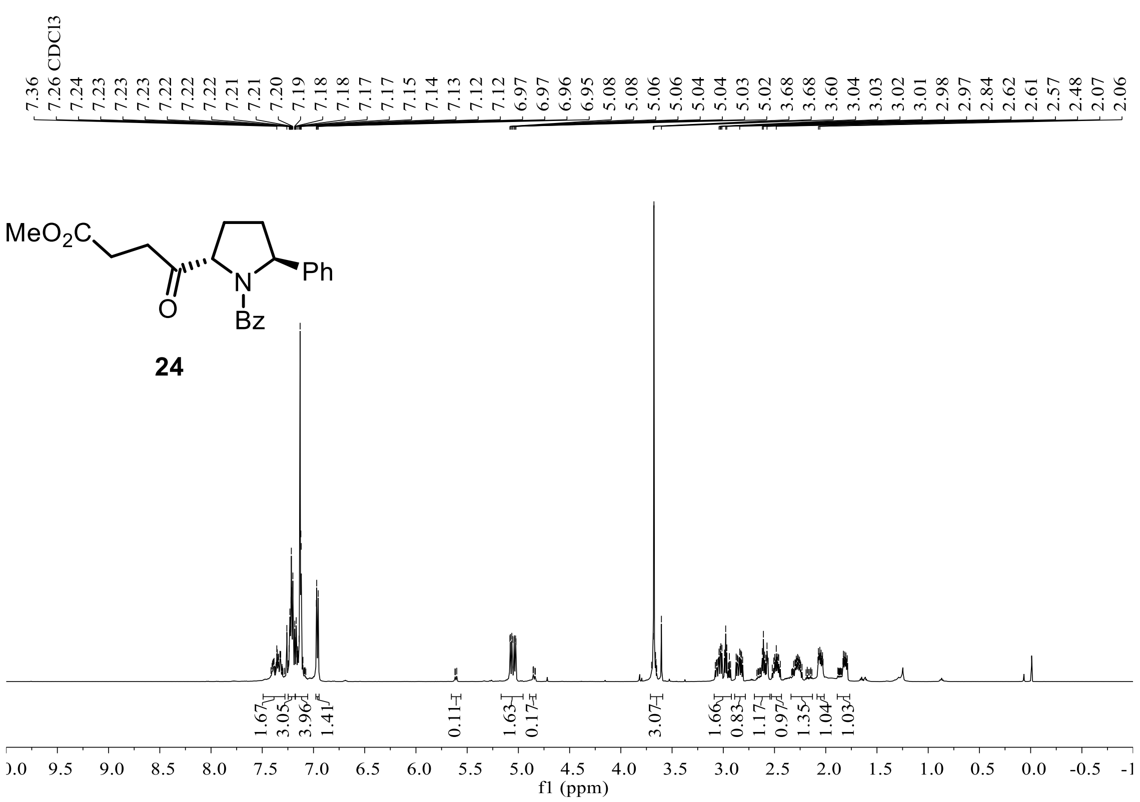

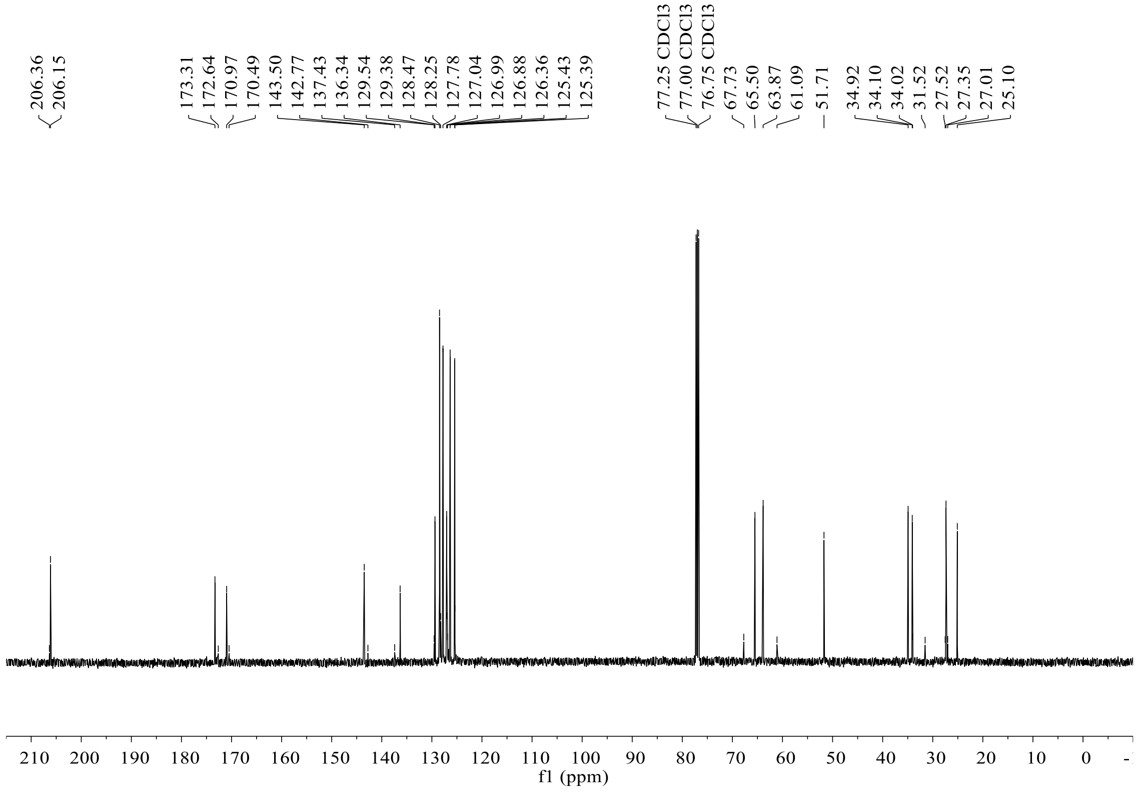

Supplementary Fig. 30 ^{1}H NMR (500 MHz, $CDCl_3$) and ^{13}C NMR (126 MHz, $CDCl_3$) spectrum of **24**.

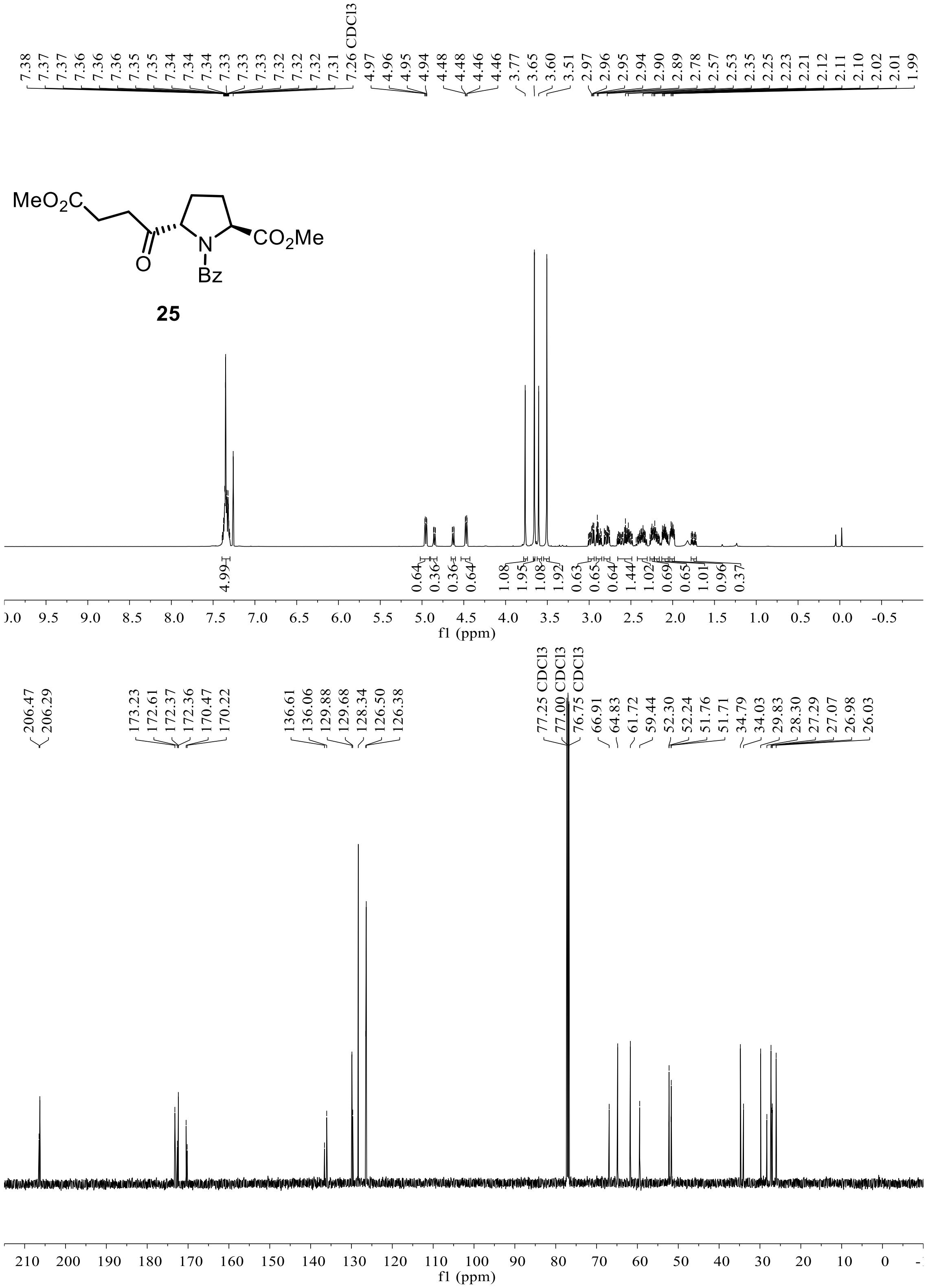

Supplementary Fig. 31 ^{1}H NMR (500 MHz, $CDCl_3$) and ^{13}C NMR (126 MHz, $CDCl_3$) spectrum of **25**.

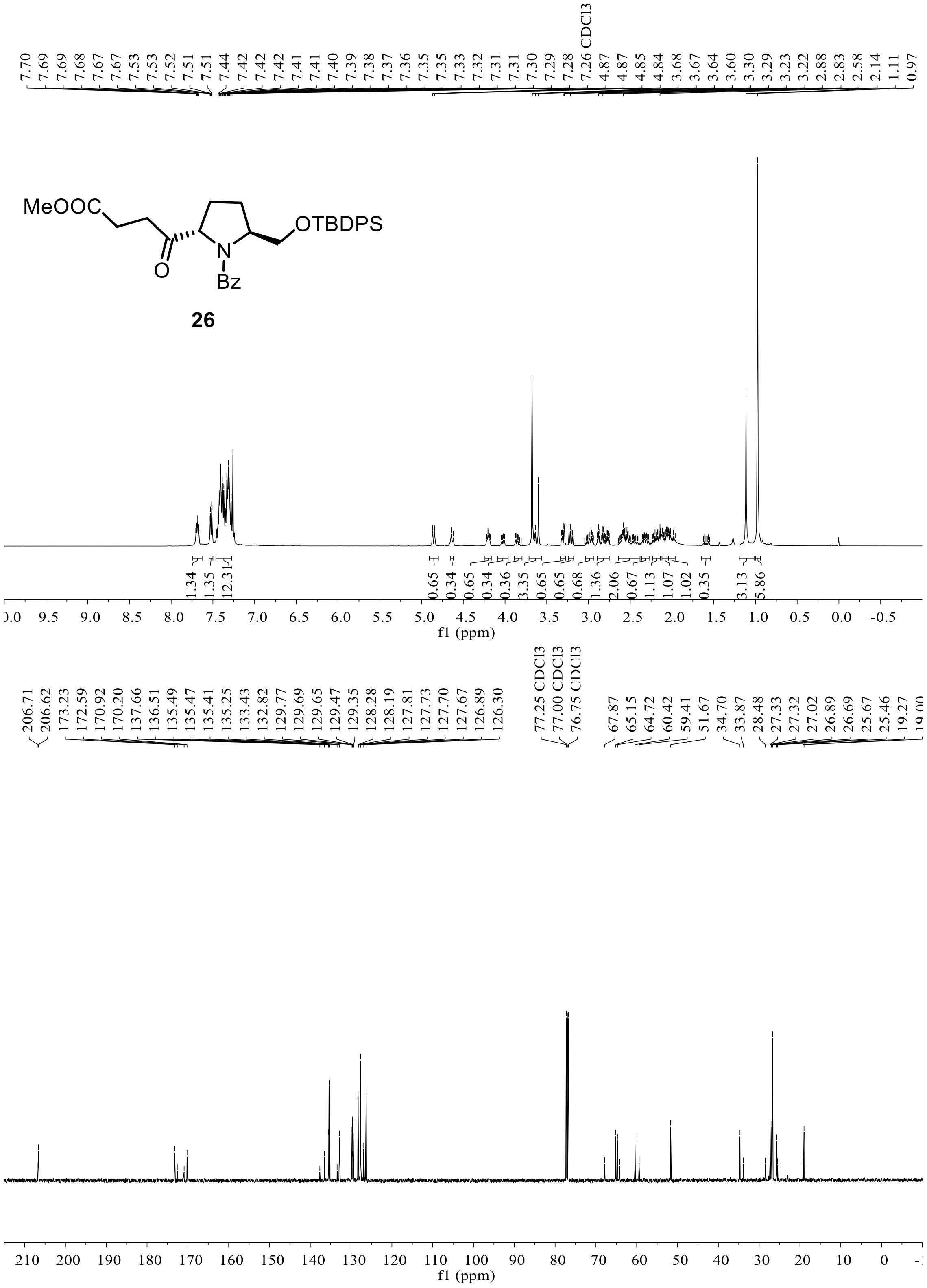

Supplementary Fig. 32 ^{1}H NMR (400 MHz, $CDCl_3$) and ^{13}C NMR (126 MHz, $CDCl_3$) spectrum of **26**.

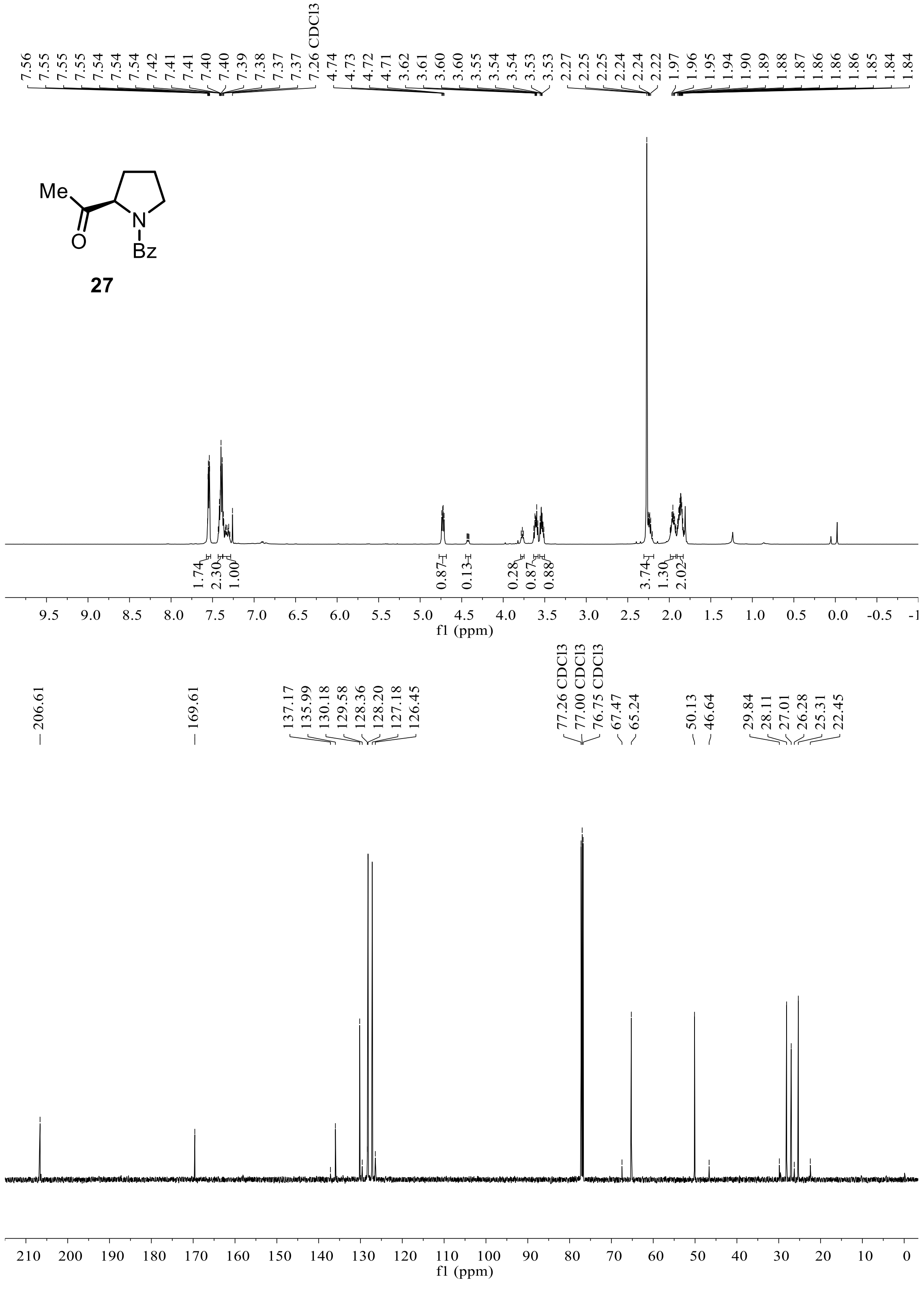

Supplementary Fig. 33 ^{1}H NMR (500 MHz, $CDCl_3$) and ^{13}C NMR (126 MHz, $CDCl_3$) spectrum of **27**.

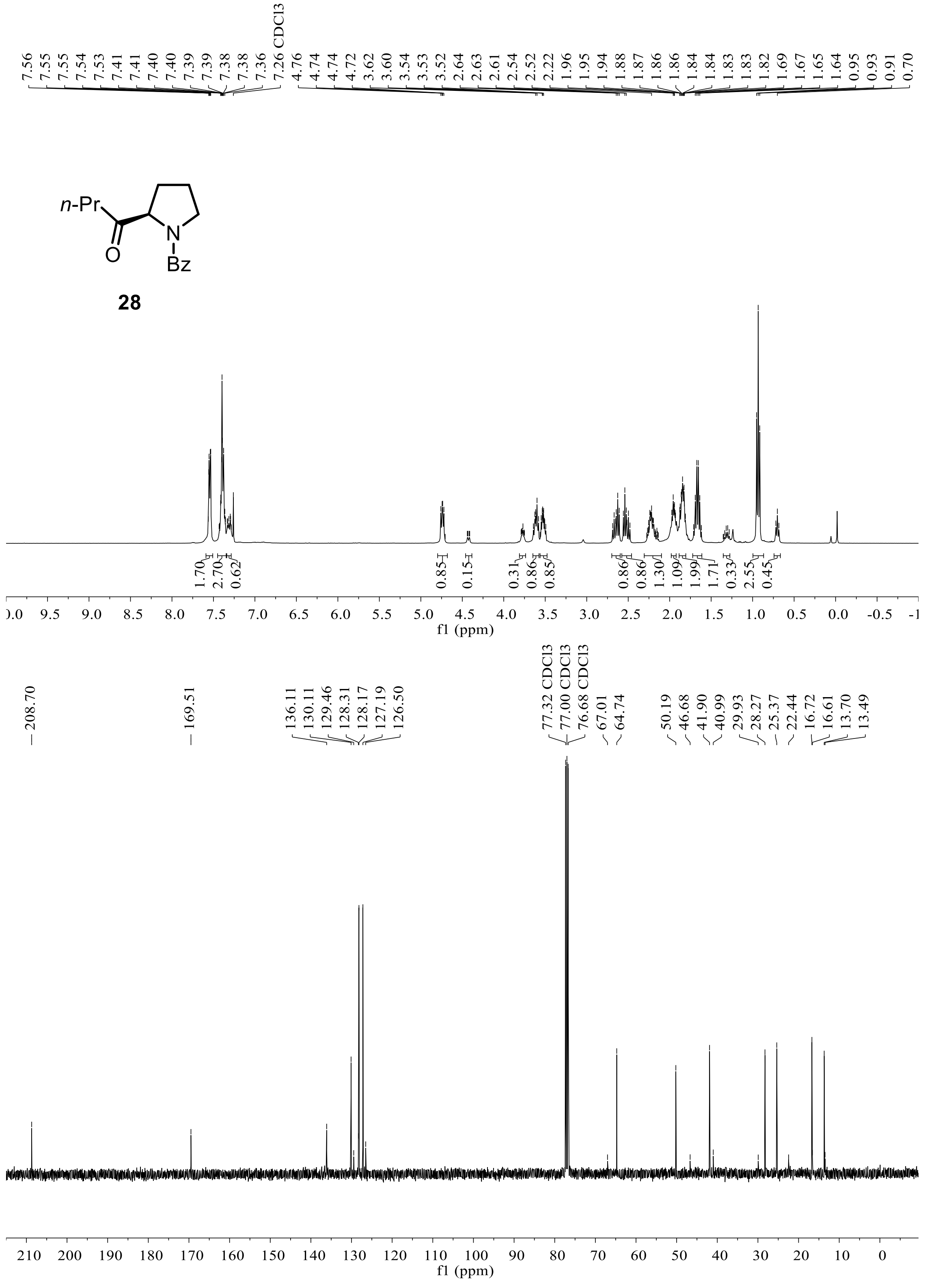

Supplementary Fig. 34 ^{1}H NMR (400 MHz, $CDCl_3$) and ^{13}C NMR (101 MHz, $CDCl_3$) spectrum of **28**.

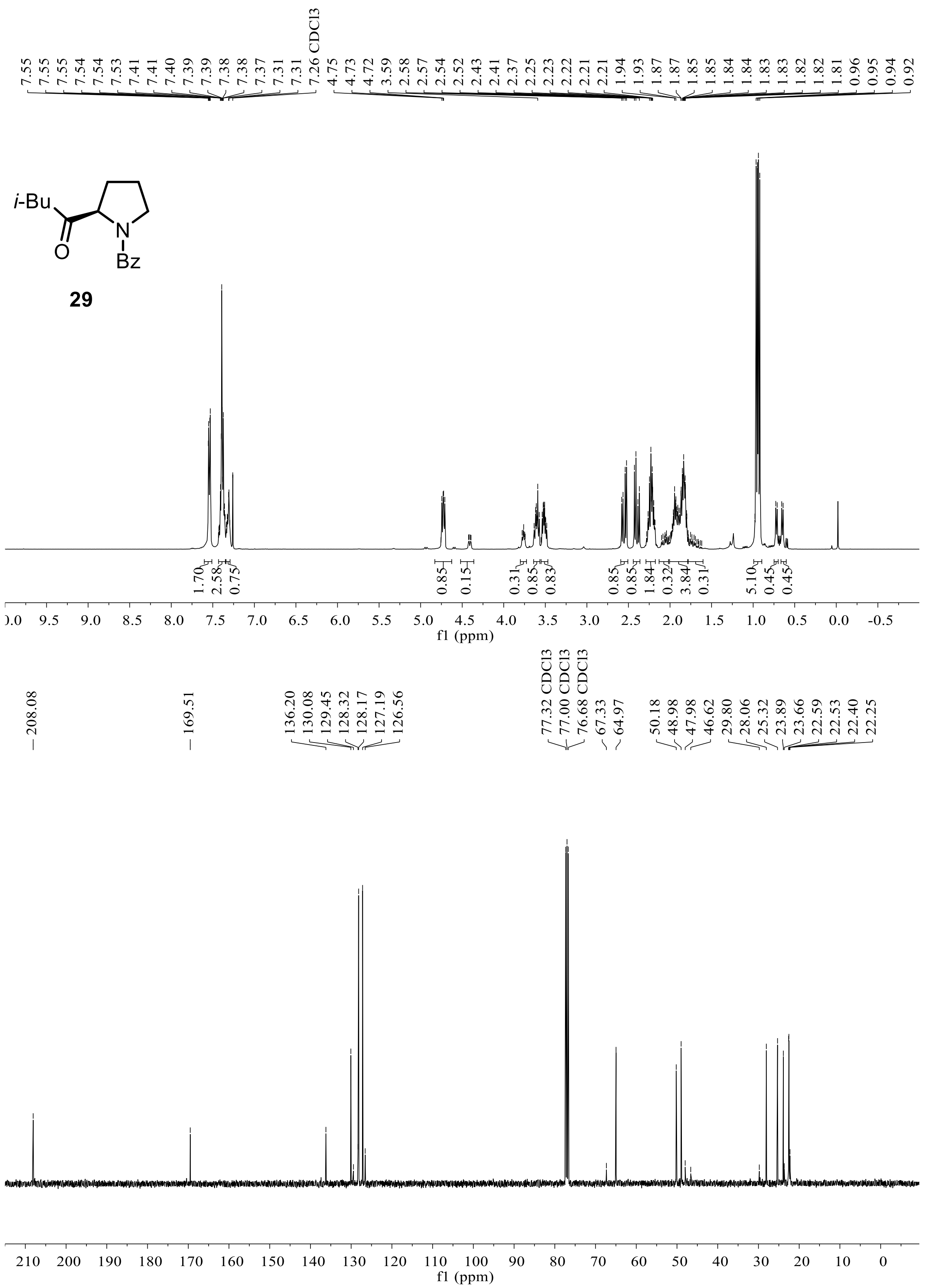

Supplementary Fig. 35 ^{1}H NMR (400 MHz, $CDCl_3$) and ^{13}C NMR (101 MHz, $CDCl_3$) spectrum of **29**.

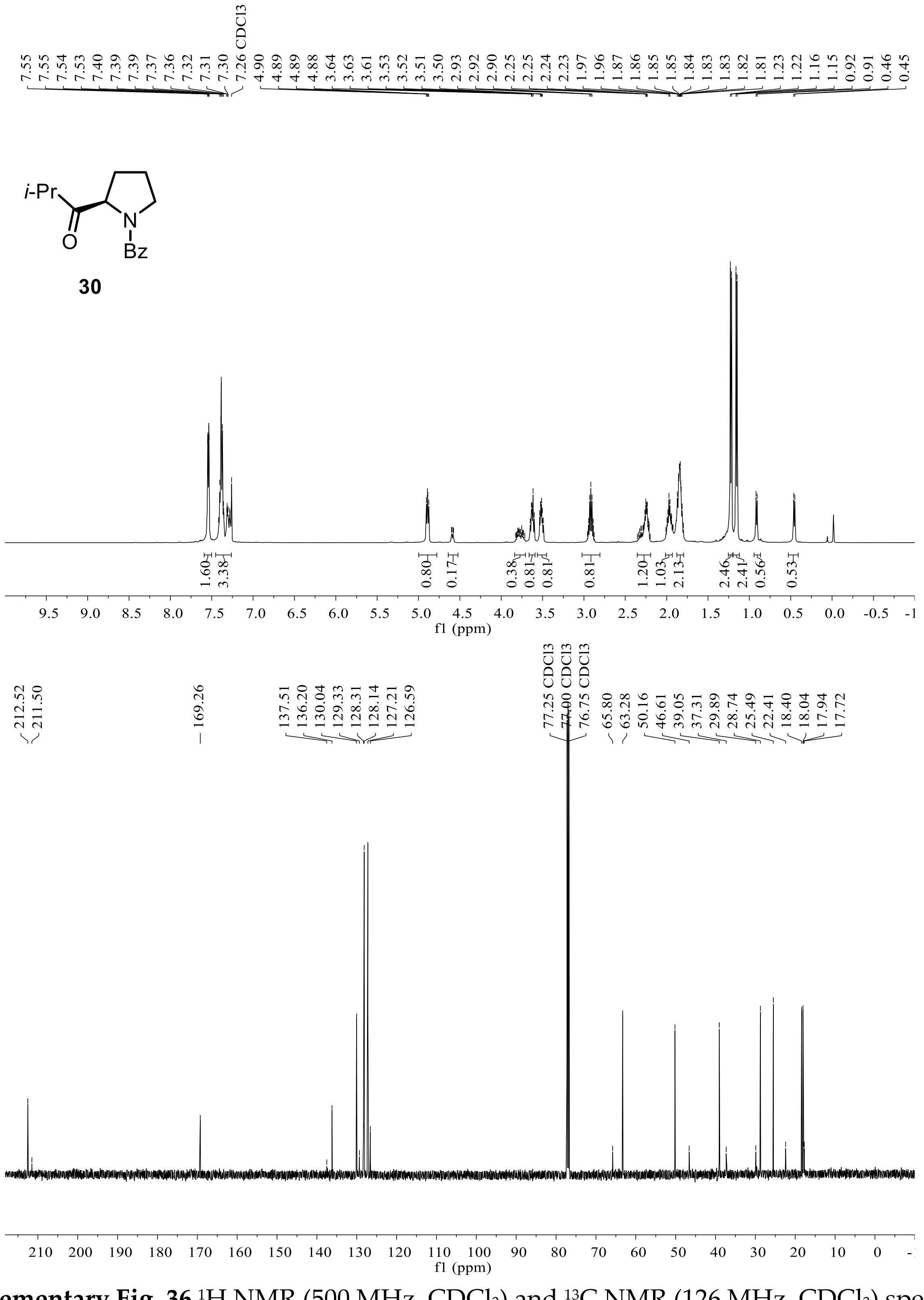

Supplementary Fig. 36 ^{1}H NMR (500 MHz, $CDCl_3$) and ^{13}C NMR (126 MHz, $CDCl_3$) spectrum of **30**.

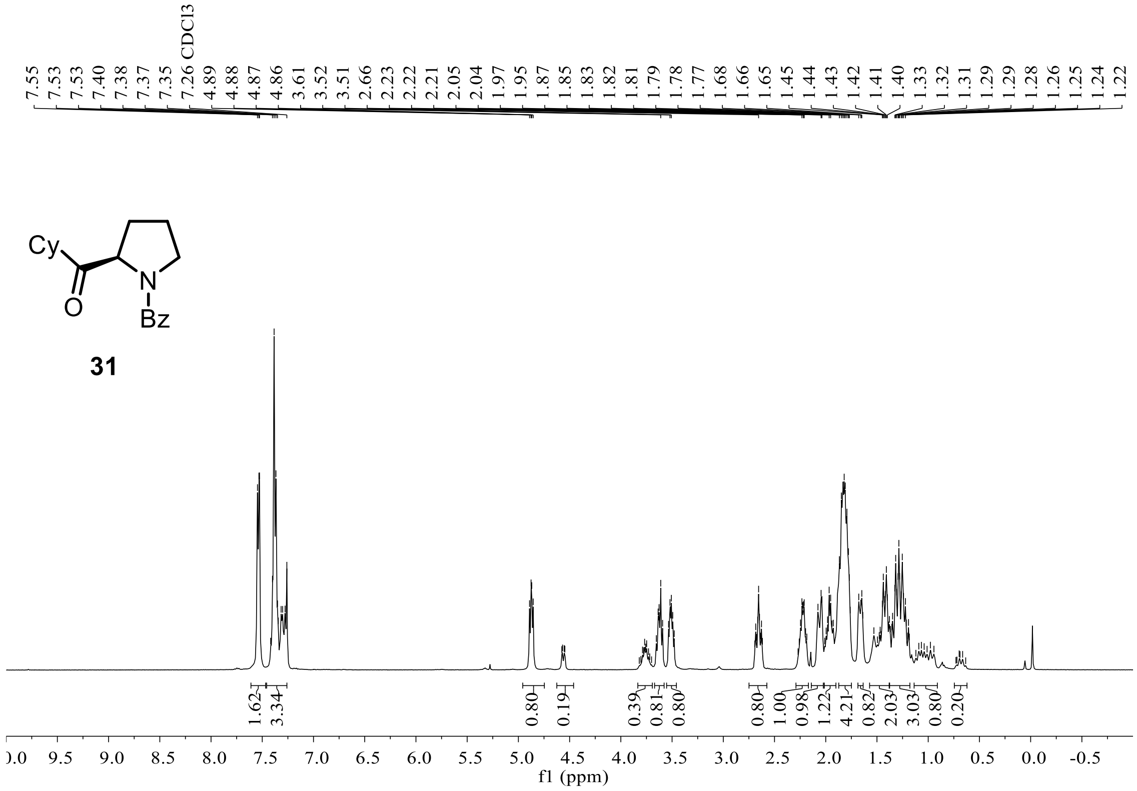

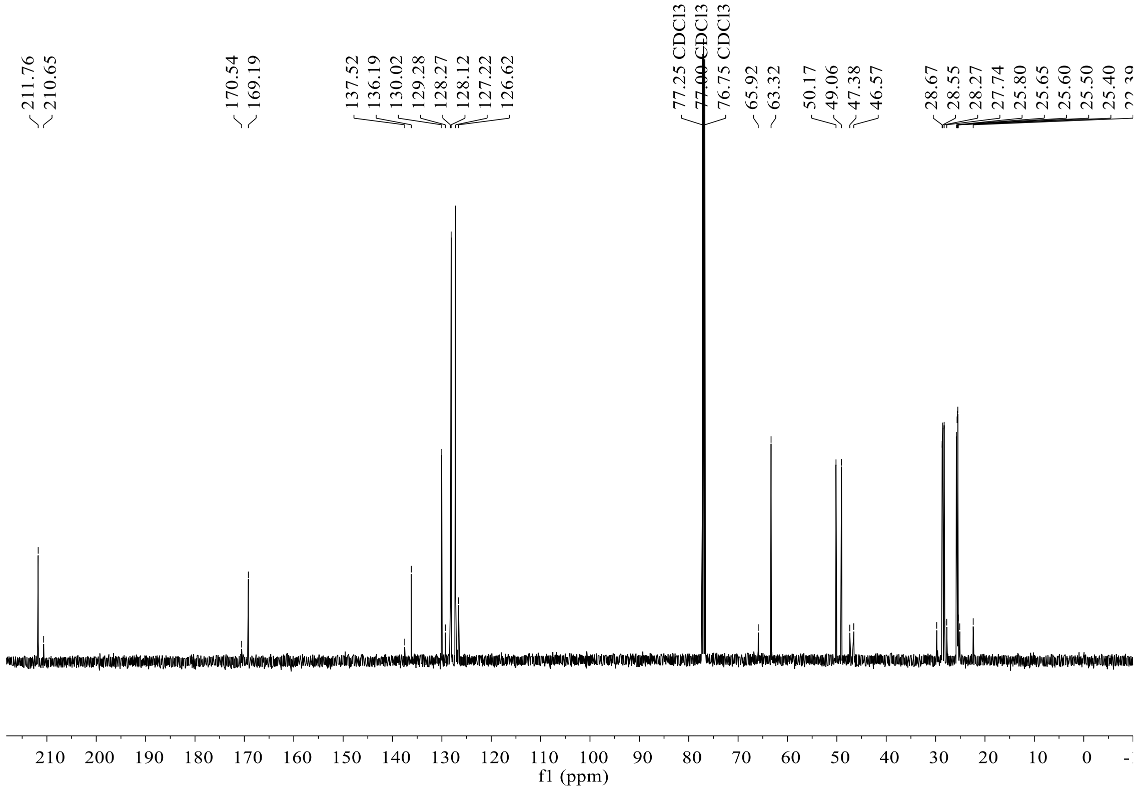

Supplementary Fig. 37 ^{1}H NMR (400 MHz, $CDCl_3$) and ^{13}C NMR (126 MHz, $CDCl_3$) spectrum of **31**.

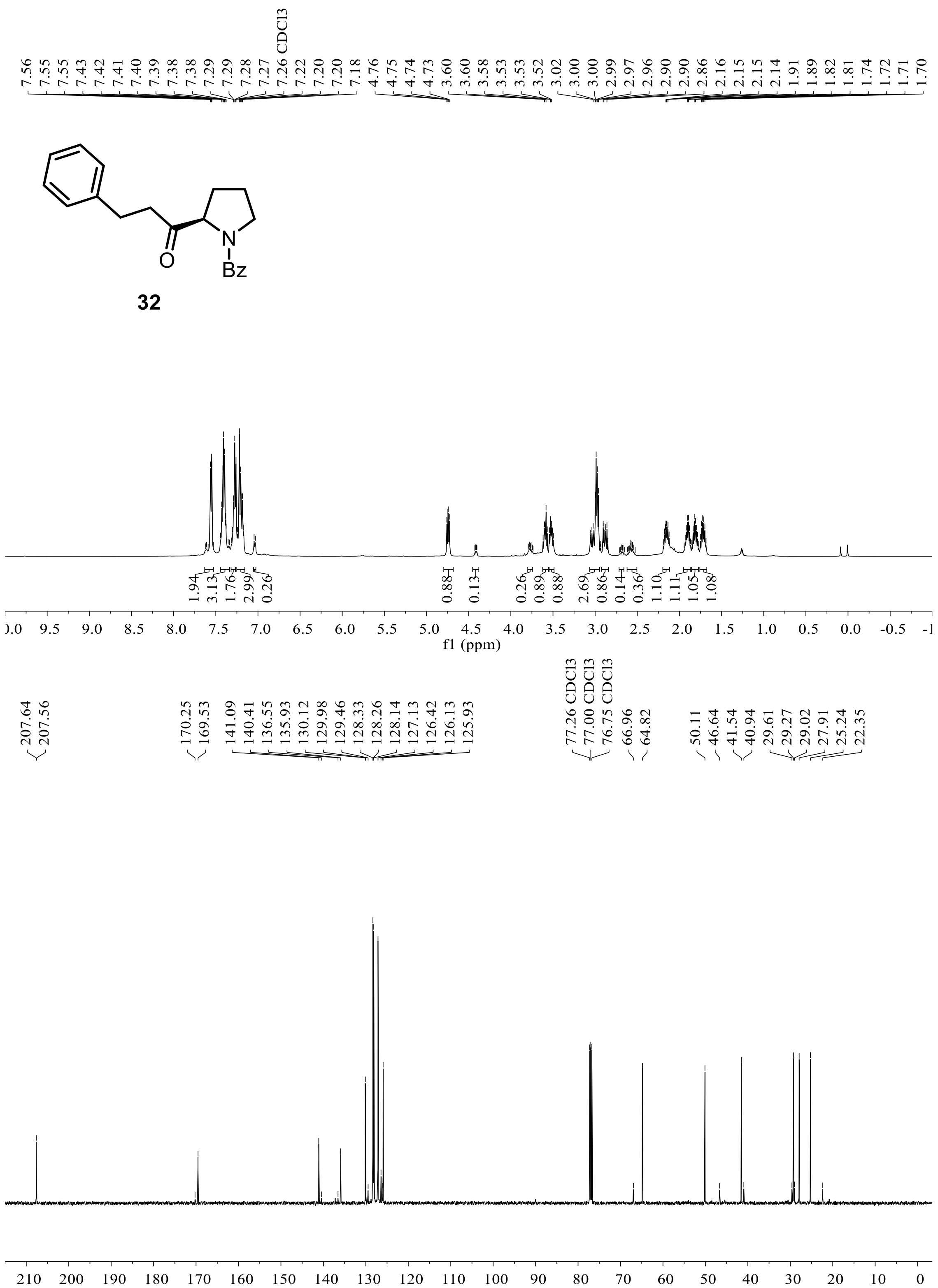

Supplementary Fig. 38 ^{1}H NMR (500 MHz, $CDCl_3$) and ^{13}C NMR (126 MHz, $CDCl_3$) spectrum of **32**.

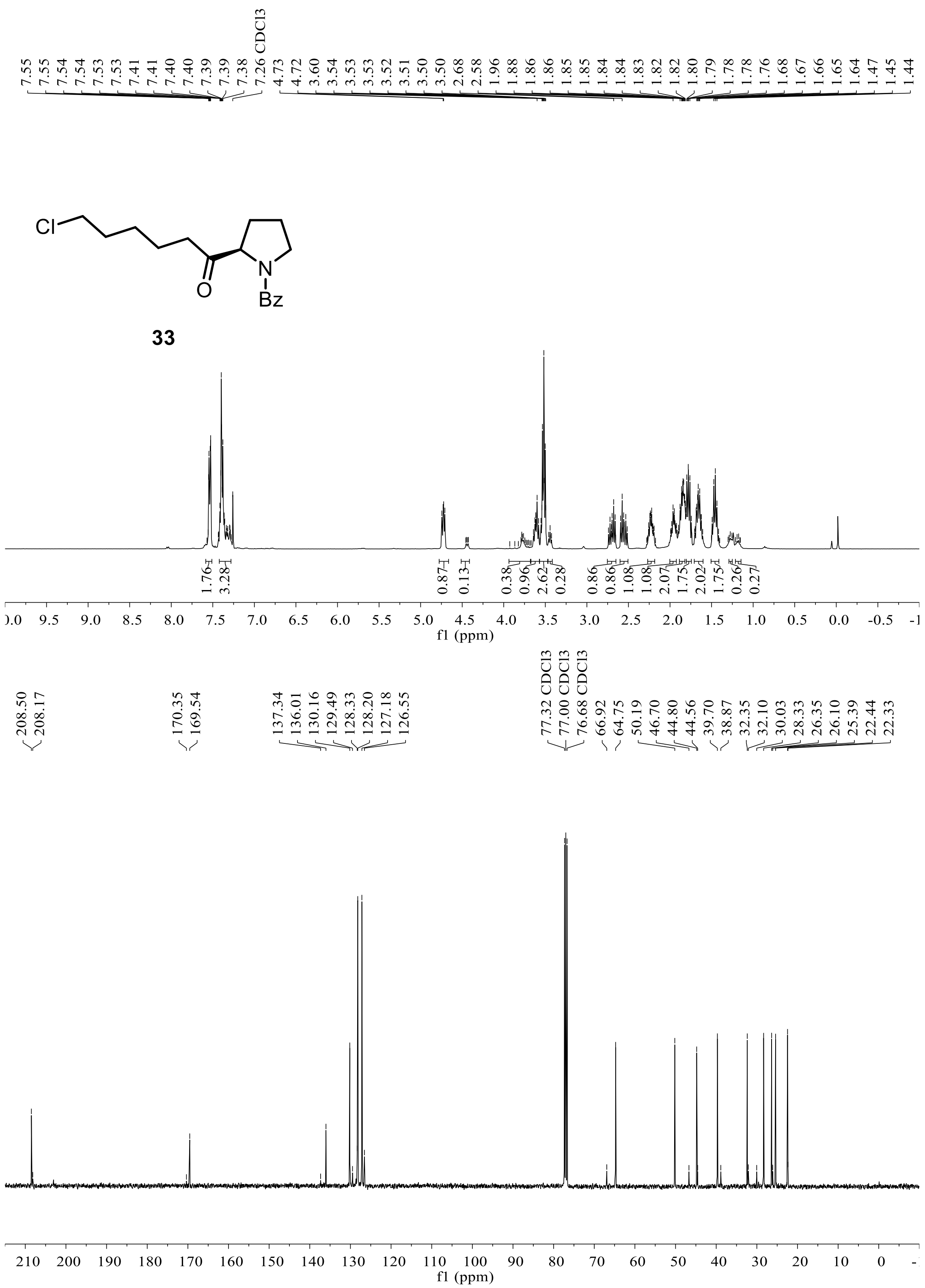

Supplementary Fig. 39 ^{1}H NMR (400 MHz, $CDCl_3$) and ^{13}C NMR (101 MHz, $CDCl_3$) spectrum of **33**.

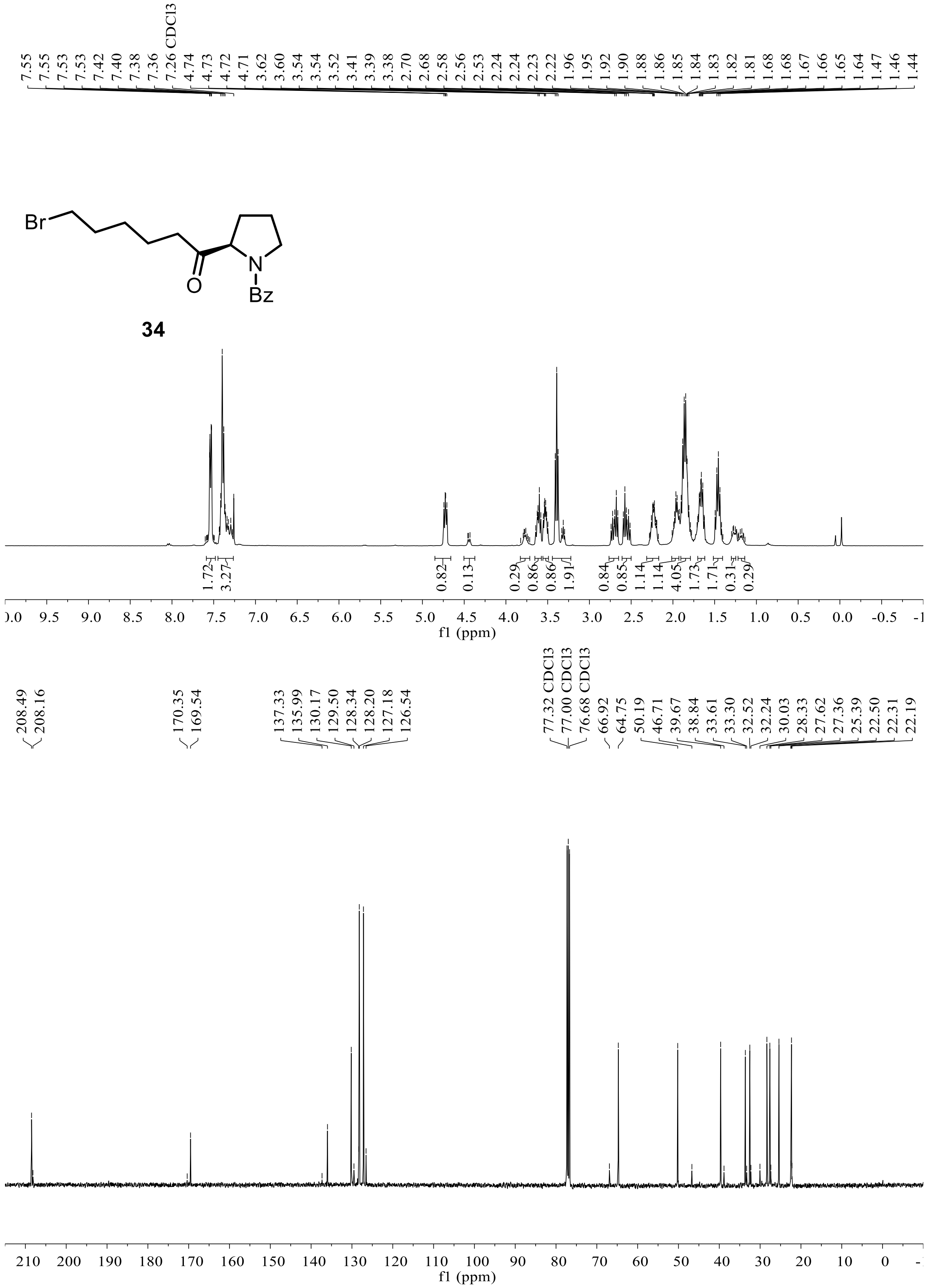

Supplementary Fig. 40 ^{1}H NMR (400 MHz, $CDCl_3$) and ^{13}C NMR (101 MHz, $CDCl_3$) spectrum of **34**.

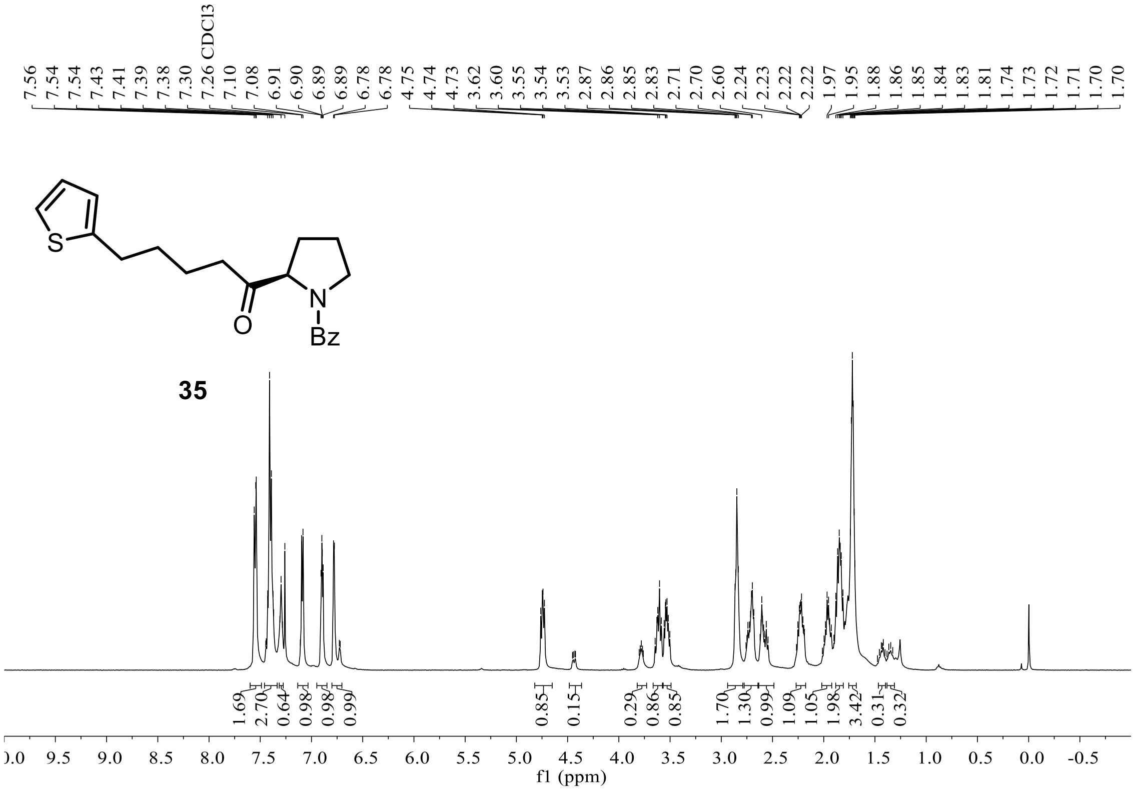

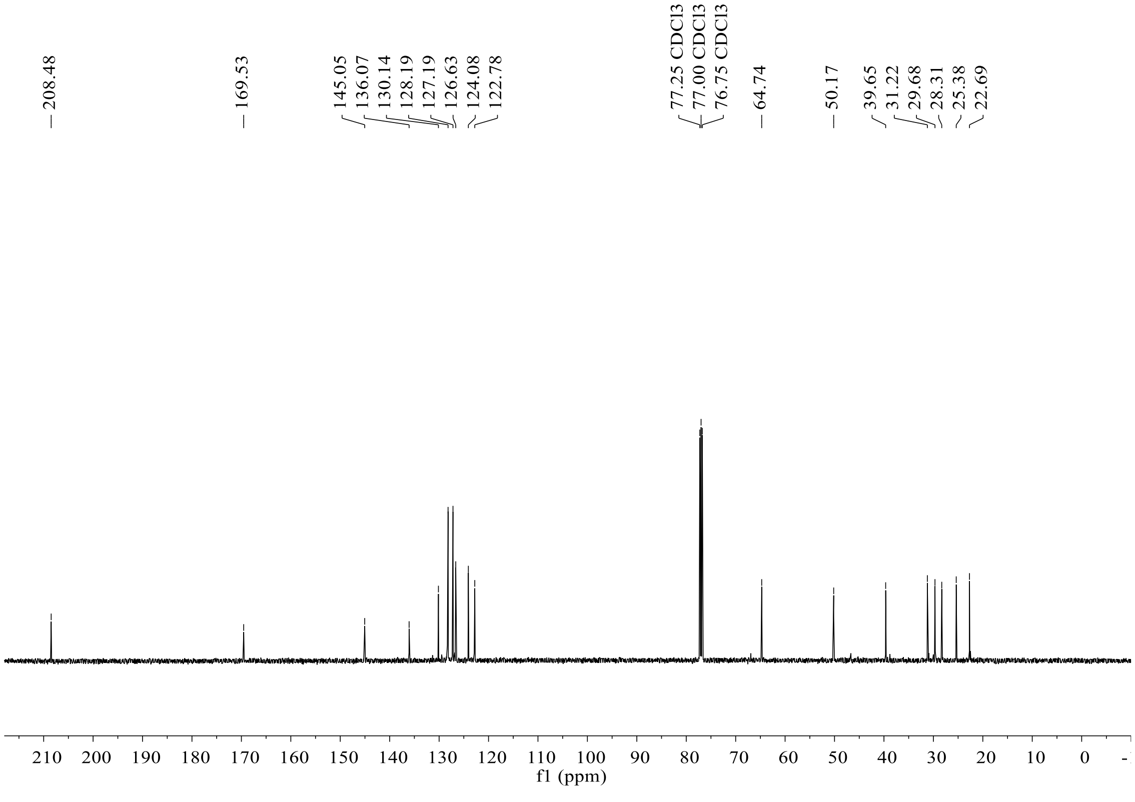

Supplementary Fig. 41 ^{1}H NMR (400 MHz, $CDCl_3$) and ^{13}C NMR (126 MHz, $CDCl_3$) spectrum of **35**.

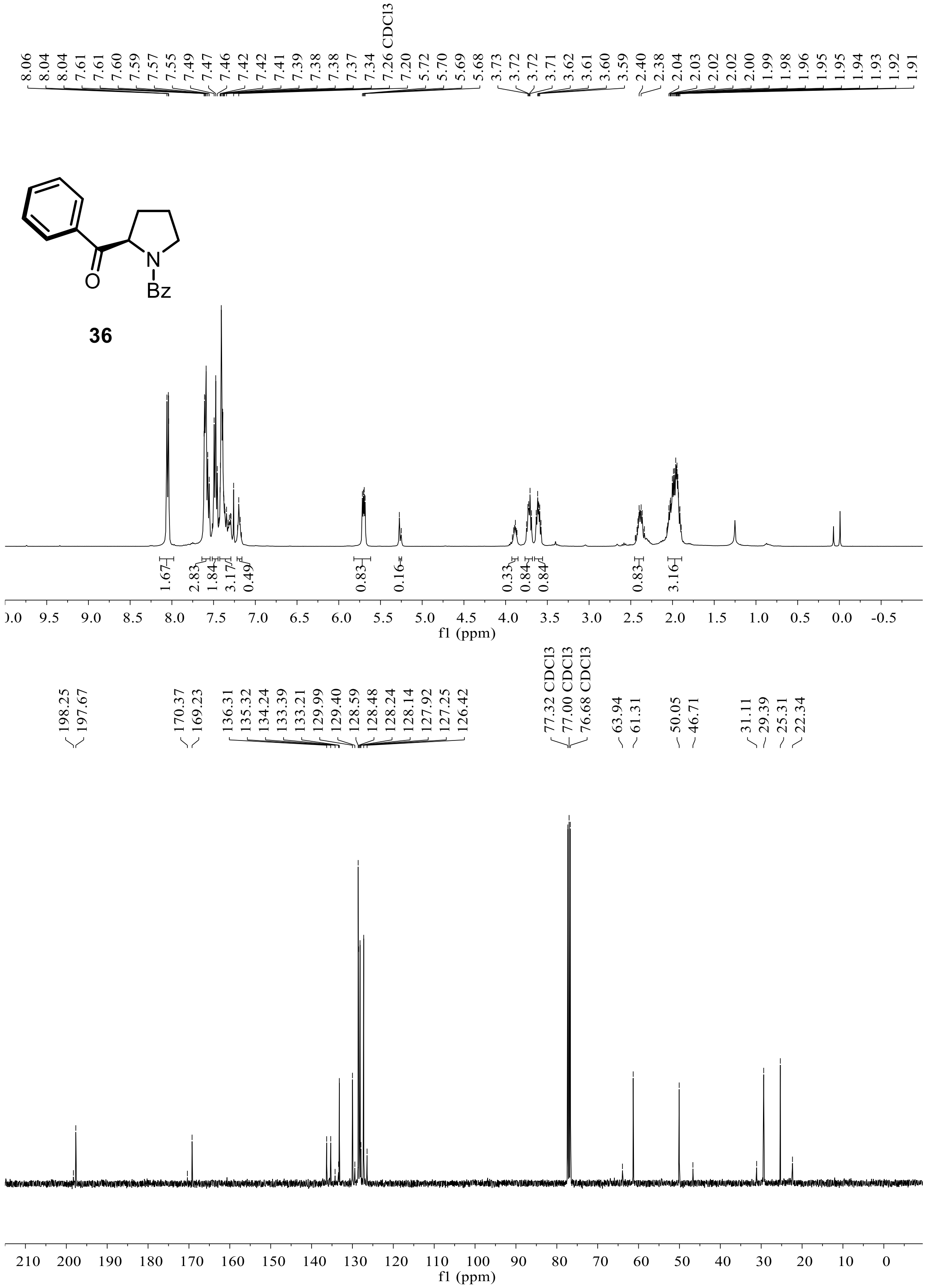

Supplementary Fig. 42 ^{1}H NMR (400 MHz, $CDCl_3$) and ^{13}C NMR (101 MHz, $CDCl_3$) spectrum of **36**.

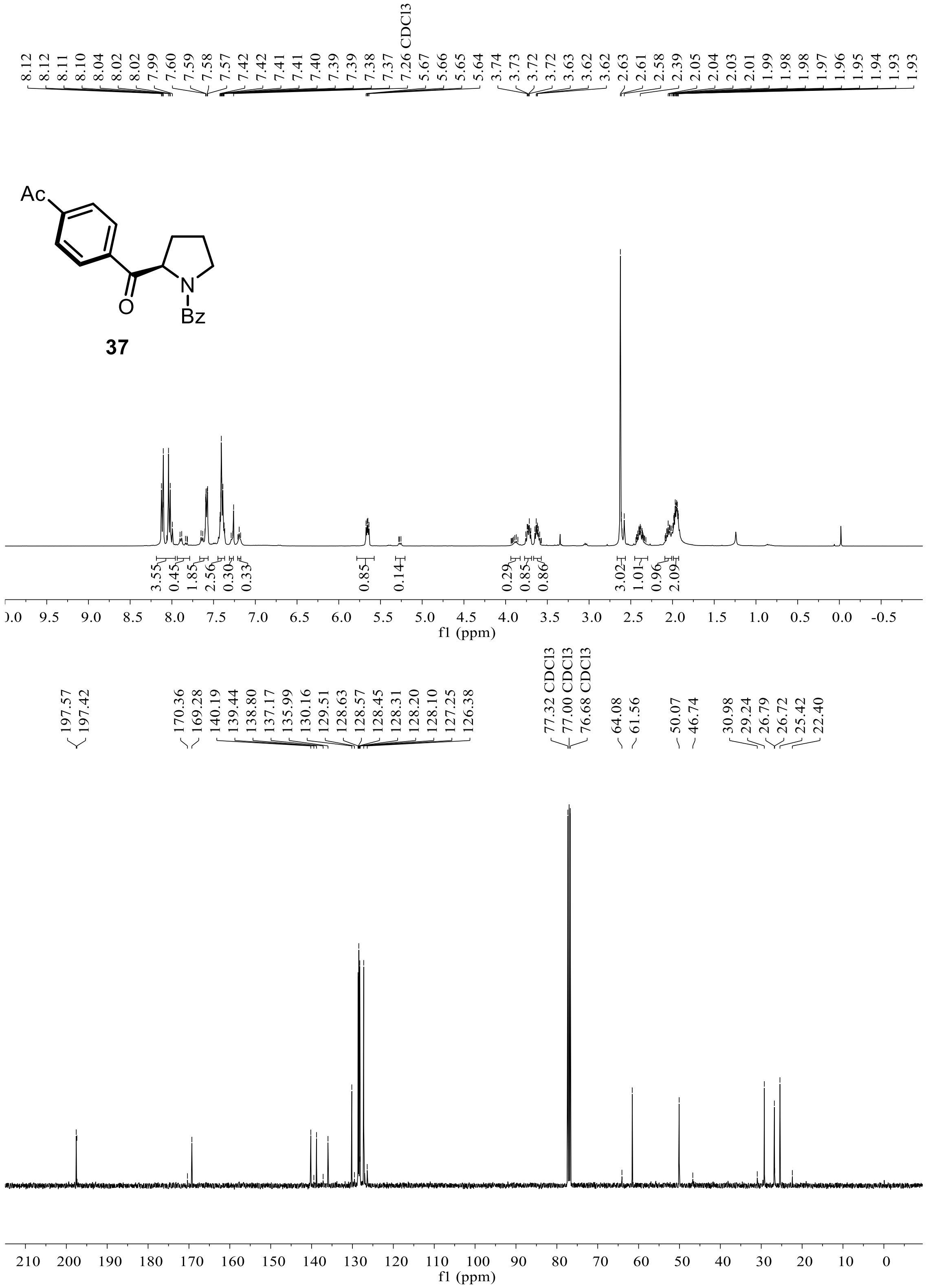

Supplementary Fig. 43 ^{1}H NMR (400 MHz, $CDCl_3$) and ^{13}C NMR (101 MHz, $CDCl_3$) spectrum of **37**.

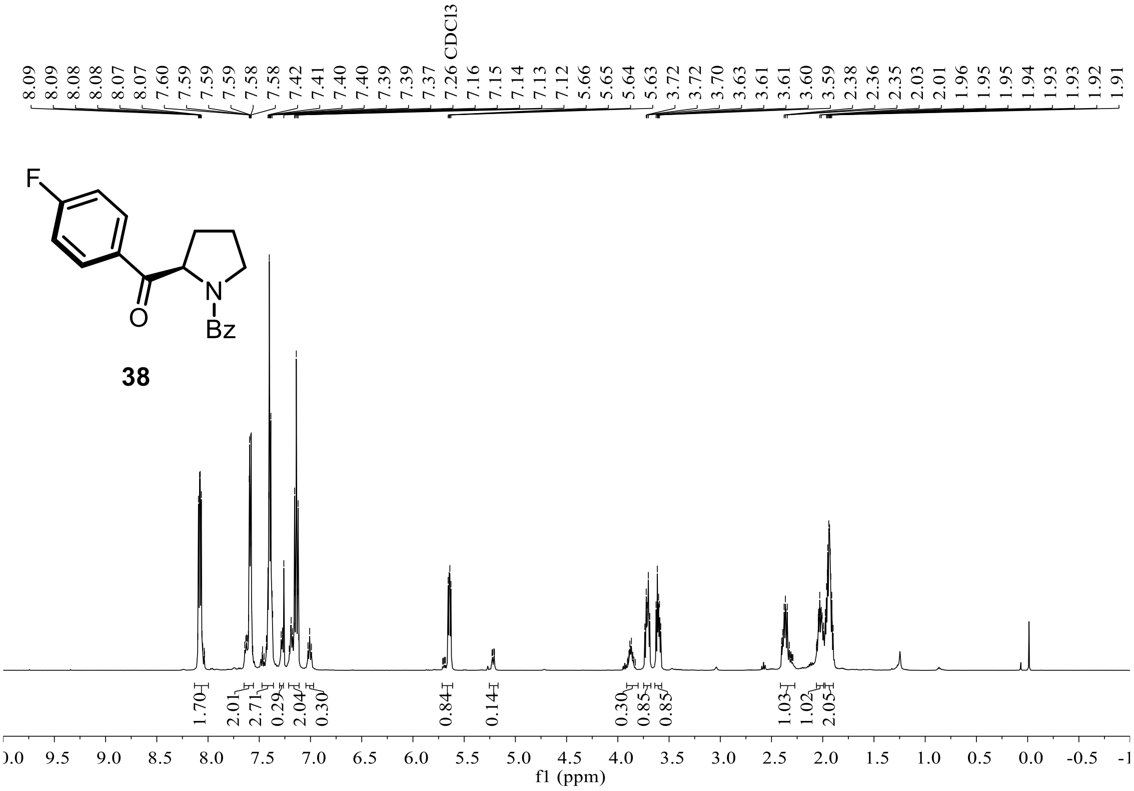
8.09 8.09 8.08 8.08 8.07 8.07 7.60 7.59 7.59 7.59 7.58 7.58 7.42 7.41 7.40 7.40 7.39 7.39 7.37 7.26 CDCl3 7.16 7.15 7.14 7.13 7.12 5.66 5.65 5.64 5.63 3.72 3.72 3.70 3.63 3.61 3.61 3.60 3.59 2.38 2.36 2.35 2.03 2.01 1.96 1.95 1.95 1.94 1.93 1.93 1.92 1.91
F
O
N
Bz
38
1.70 2.01 2.71 0.29 2.04 0.30 0.84 0.14 0.30 0.85 0.85 1.03 1.02 2.05
f1 (ppm)

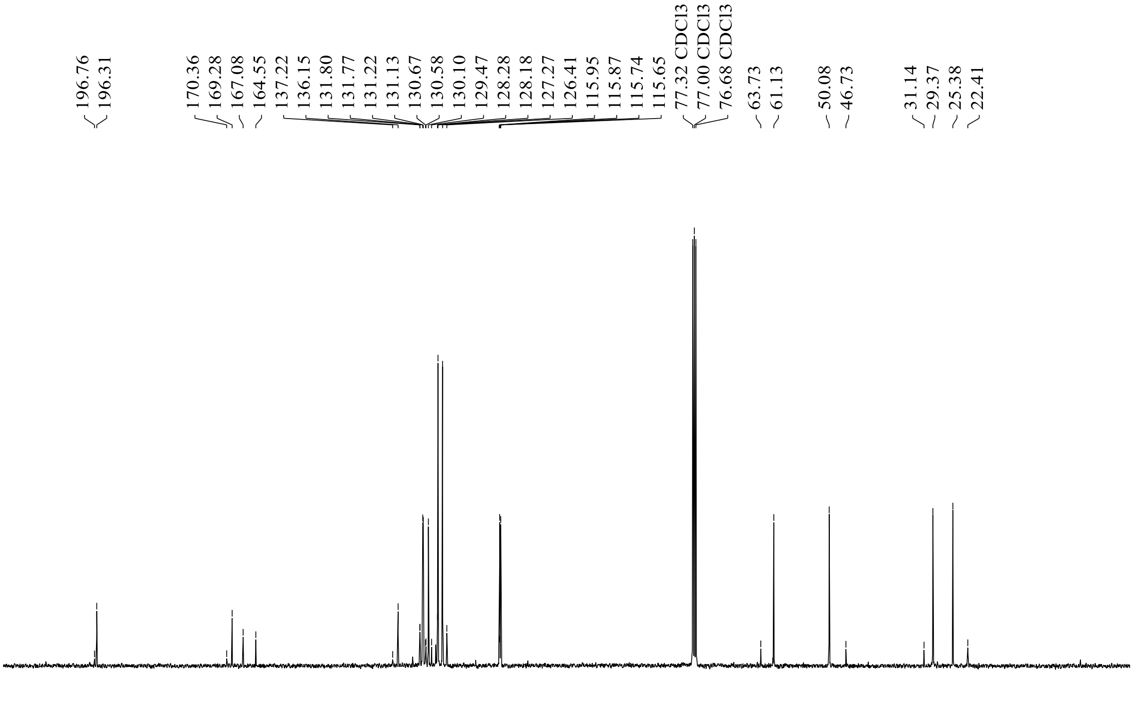
196.76 196.31 170.36 169.28 167.08 164.55 137.22 136.15 131.80 131.77 131.22 131.13 130.67 130.58 130.10 129.47 128.28 128.18 127.27 126.41 115.95 115.87 115.74 115.65 77.32 CDCl3 77.00 CDCl3 76.68 CDCl3 63.73 61.13 50.08 46.73 31.14 29.37 25.38 22.41
f1 (ppm)

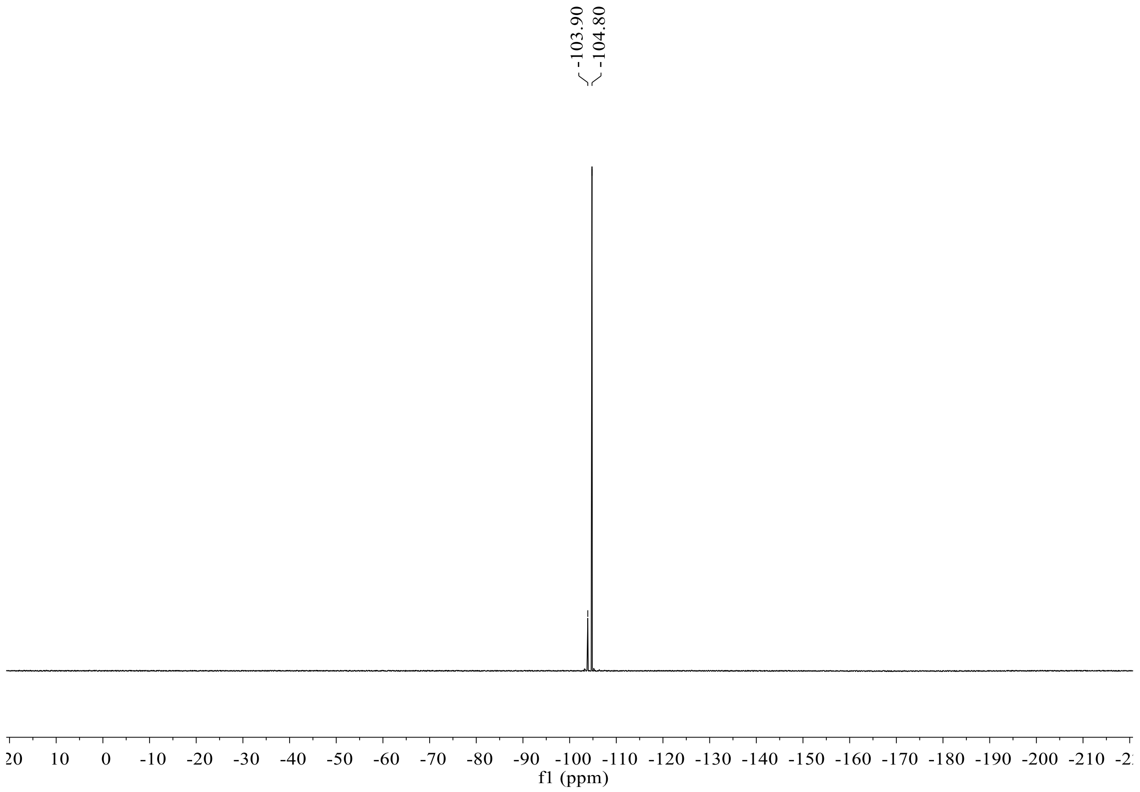

Supplementary Fig. 44 ^{1}H NMR (500 MHz, $CDCl_3$), ^{13}C NMR (101 MHz, $CDCl_3$) and ^{19}F NMR (471 MHz, $CDCl_3$) spectrum of **38**.

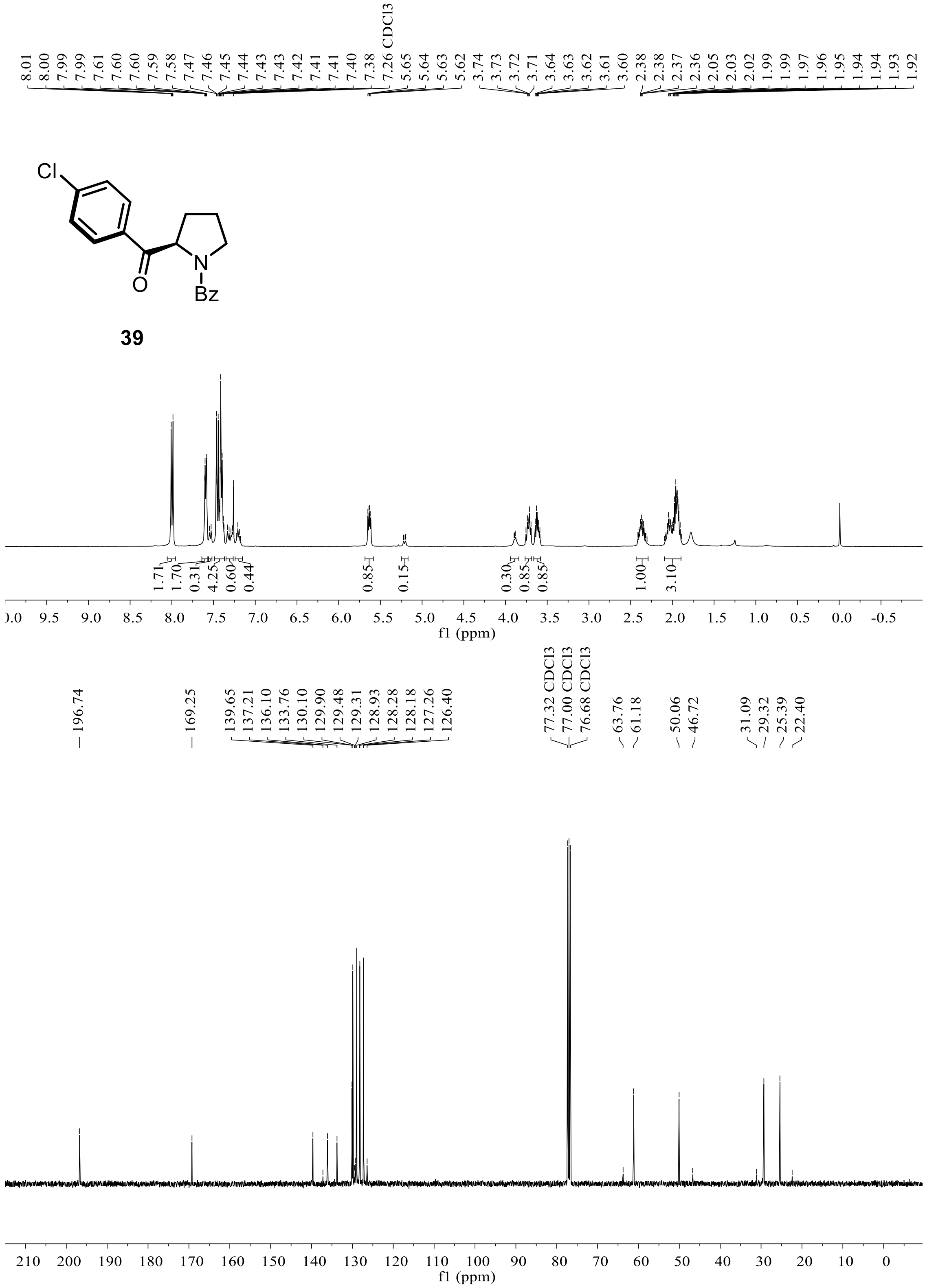

Supplementary Fig. 45 ^{1}H NMR (400 MHz, $CDCl_3$) and ^{13}C NMR (101 MHz, $CDCl_3$) spectrum of **39**.

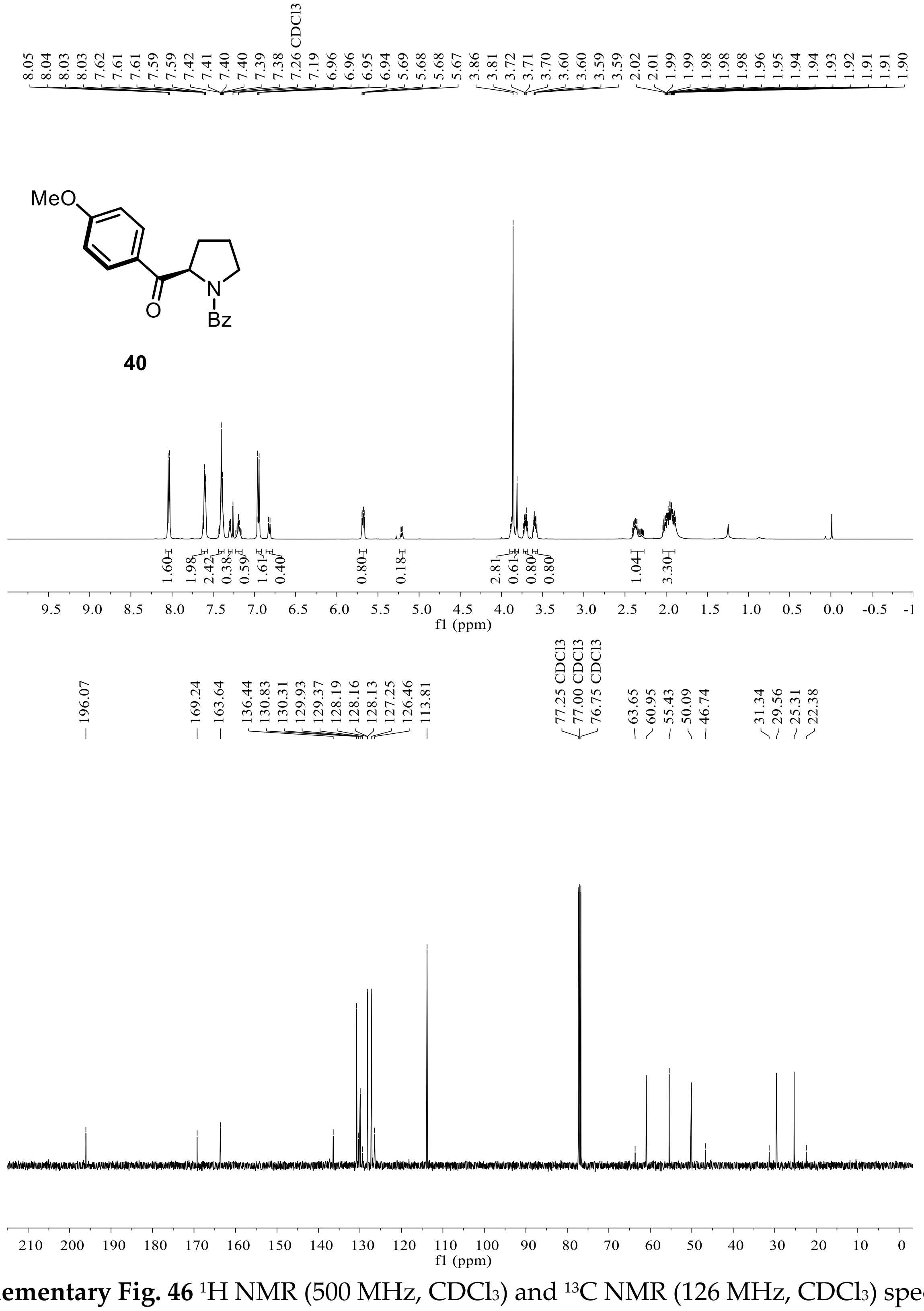

Supplementary Fig. 46 ^{1}H NMR (500 MHz, $CDCl_3$) and ^{13}C NMR (126 MHz, $CDCl_3$) spectrum of **40**.

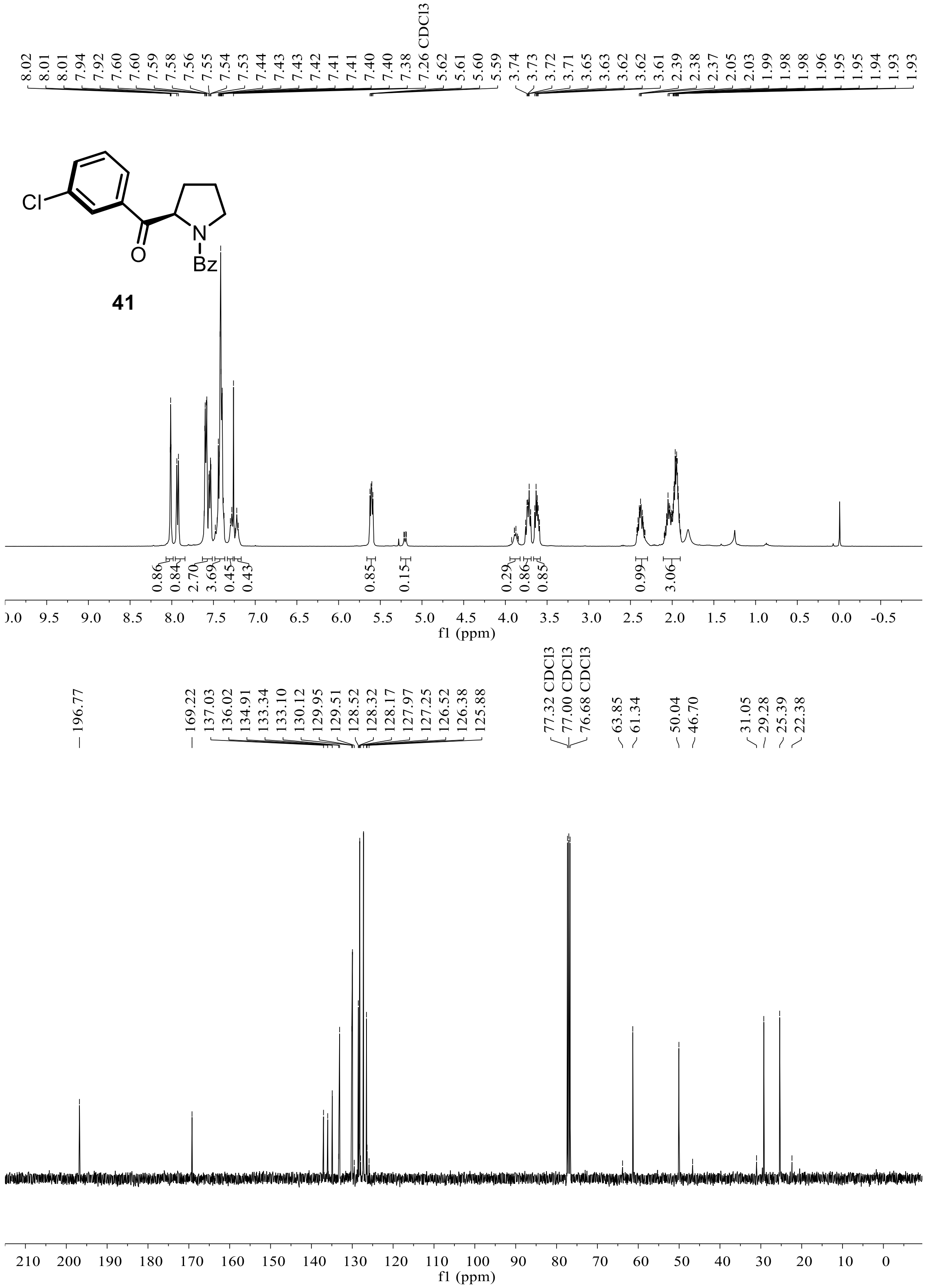

Supplementary Fig. 47 ^{1}H NMR (400 MHz, $CDCl_3$) and ^{13}C NMR (101 MHz, $CDCl_3$) spectrum of **41**.

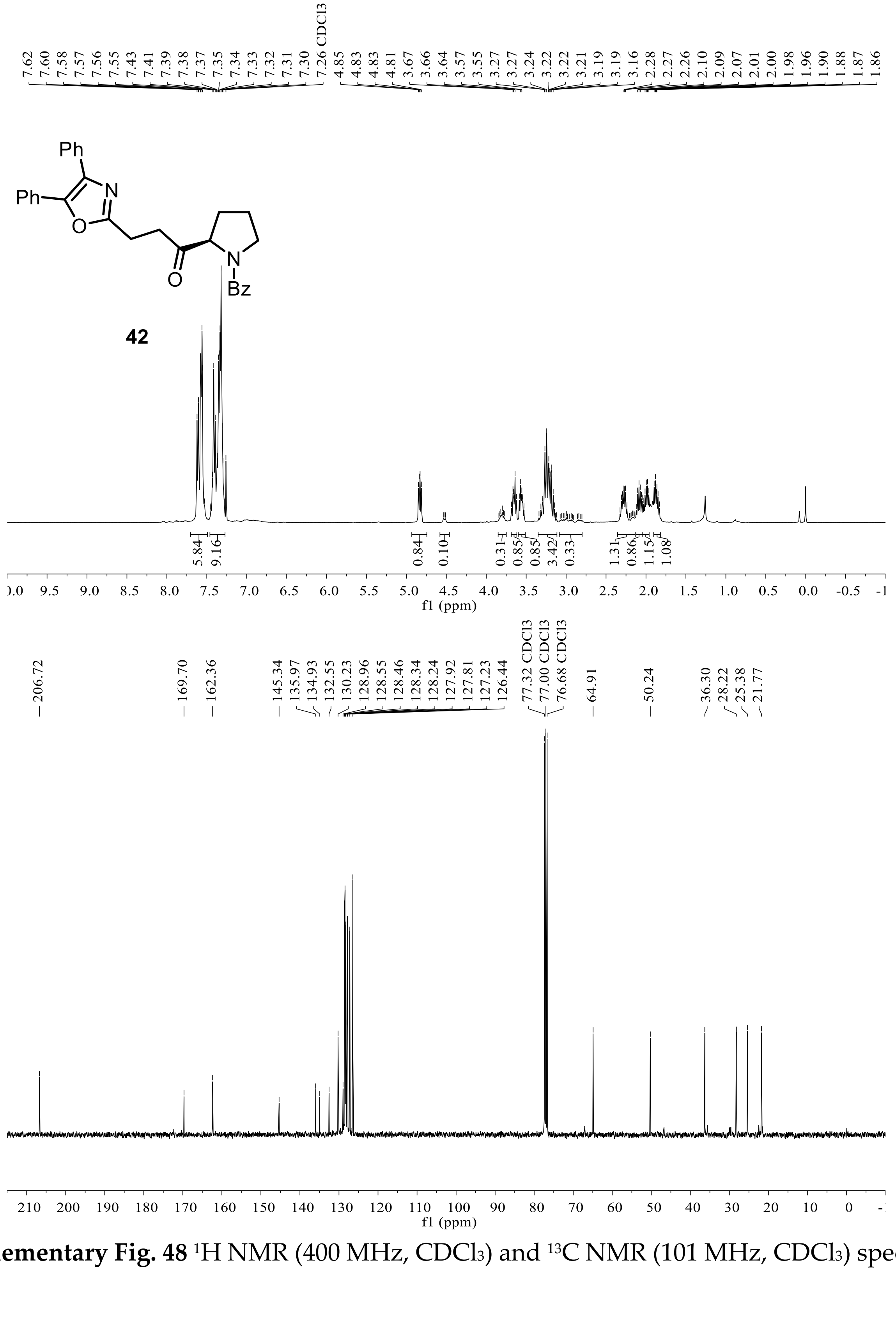

Supplementary Fig. 48 ^{1}H NMR (400 MHz, $CDCl_3$) and ^{13}C NMR (101 MHz, $CDCl_3$) spectrum of **42**.

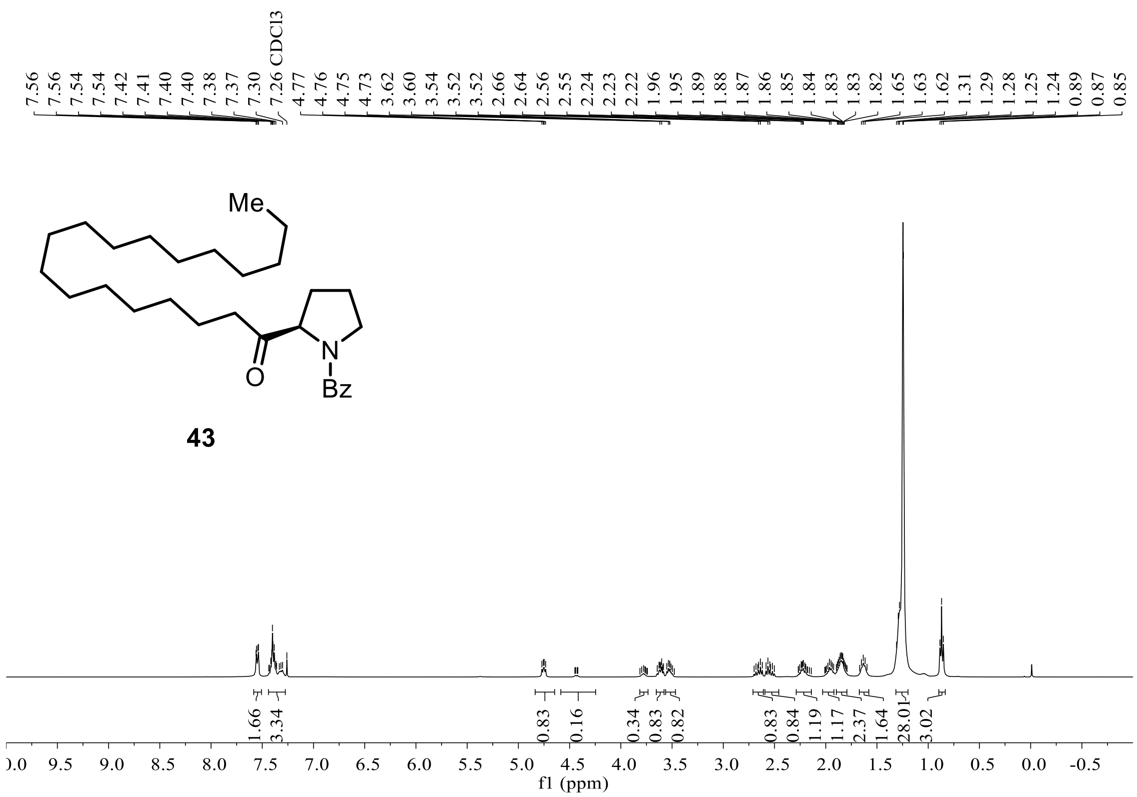

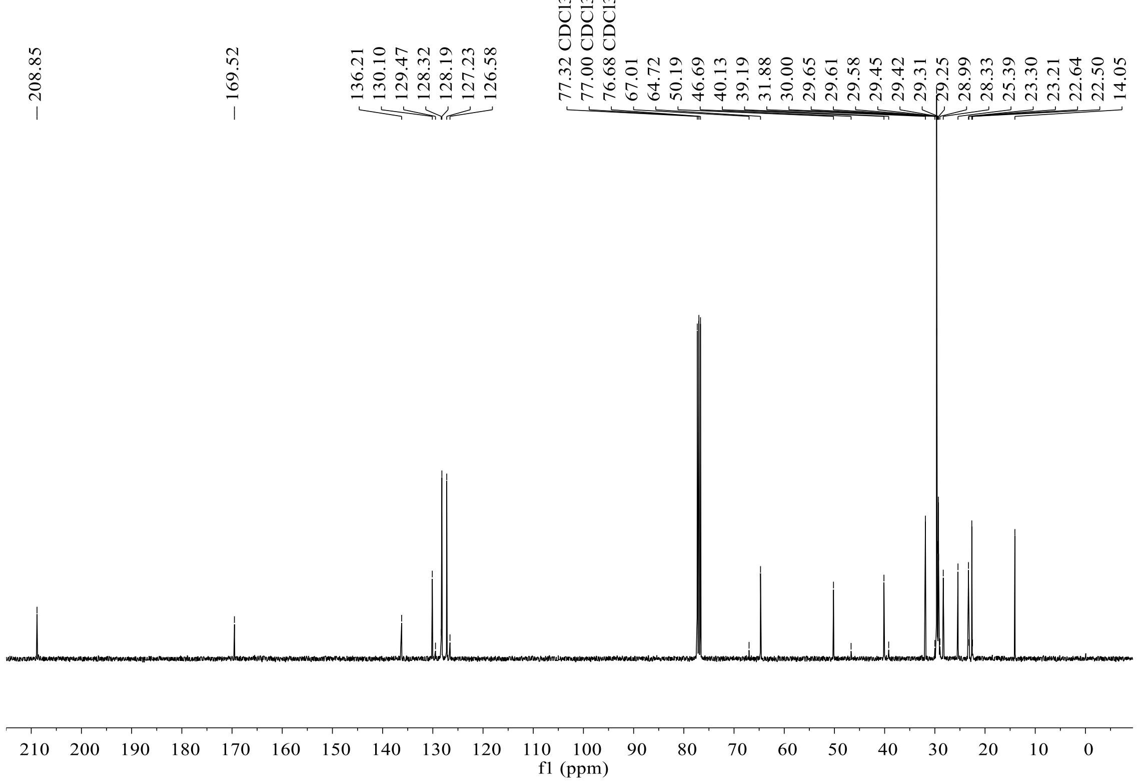

Supplementary Fig. 49 ^{1}H NMR (400 MHz, $CDCl_3$) and ^{13}C NMR (101 MHz, $CDCl_3$) spectrum of **43**.

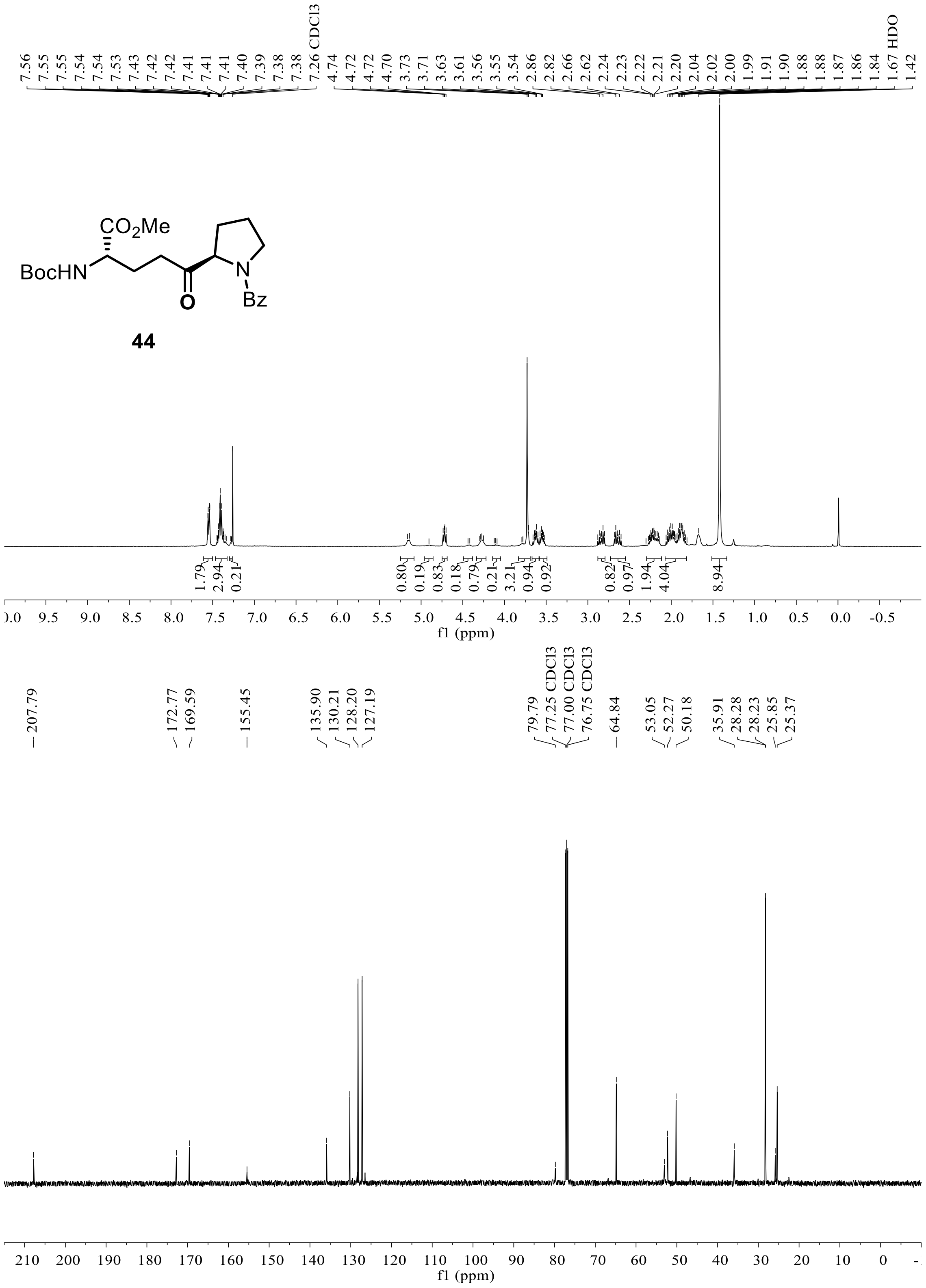

Supplementary Fig. 50 ¹H NMR (400 MHz, CDCl3) and ¹³C NMR (126 MHz, CDCl3) spectrum of **44**.

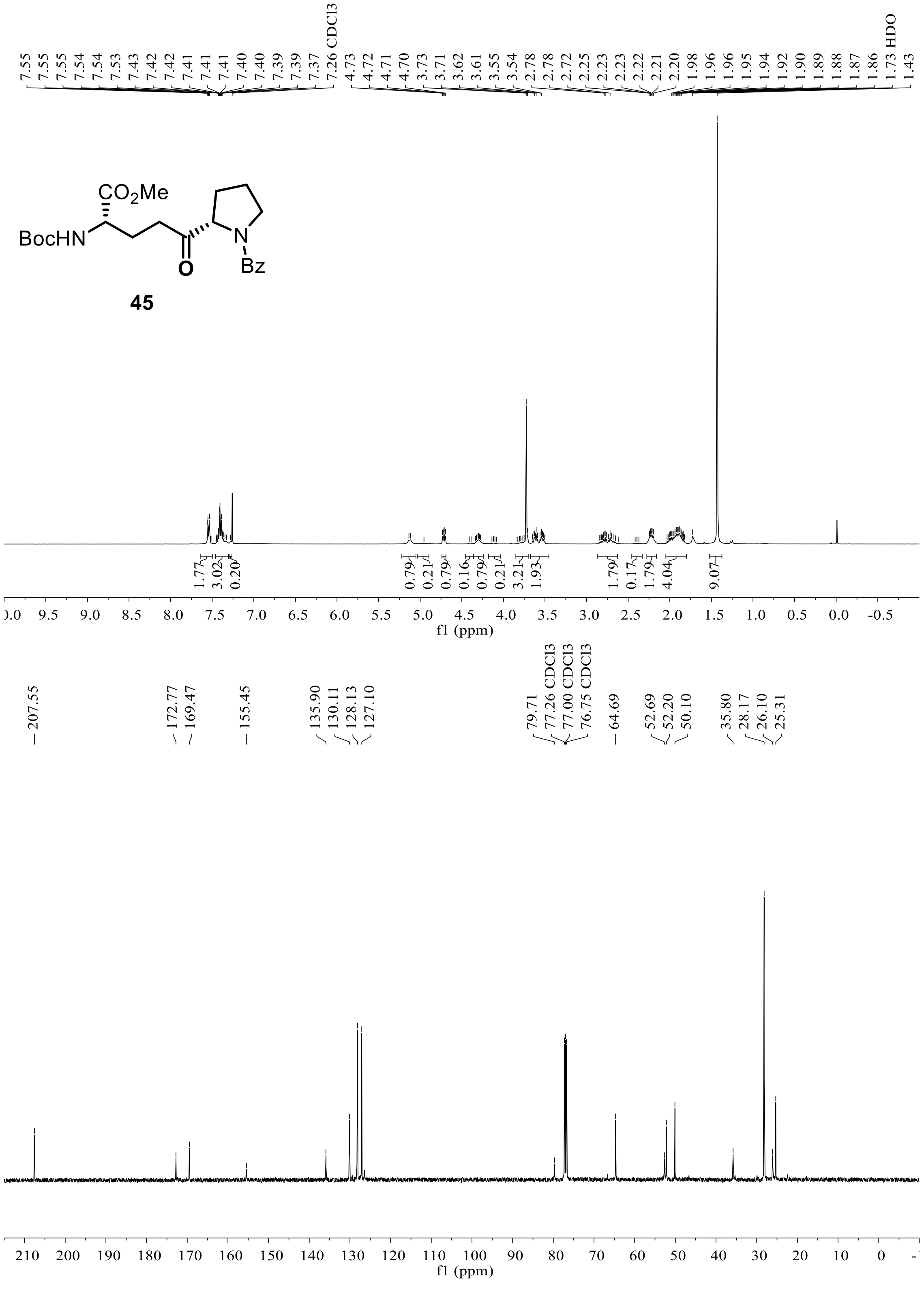

Supplementary Fig. 51 ^{1}H NMR (400 MHz, $CDCl_3$) and ^{13}C NMR (126 MHz, $CDCl_3$) spectrum of **45**.

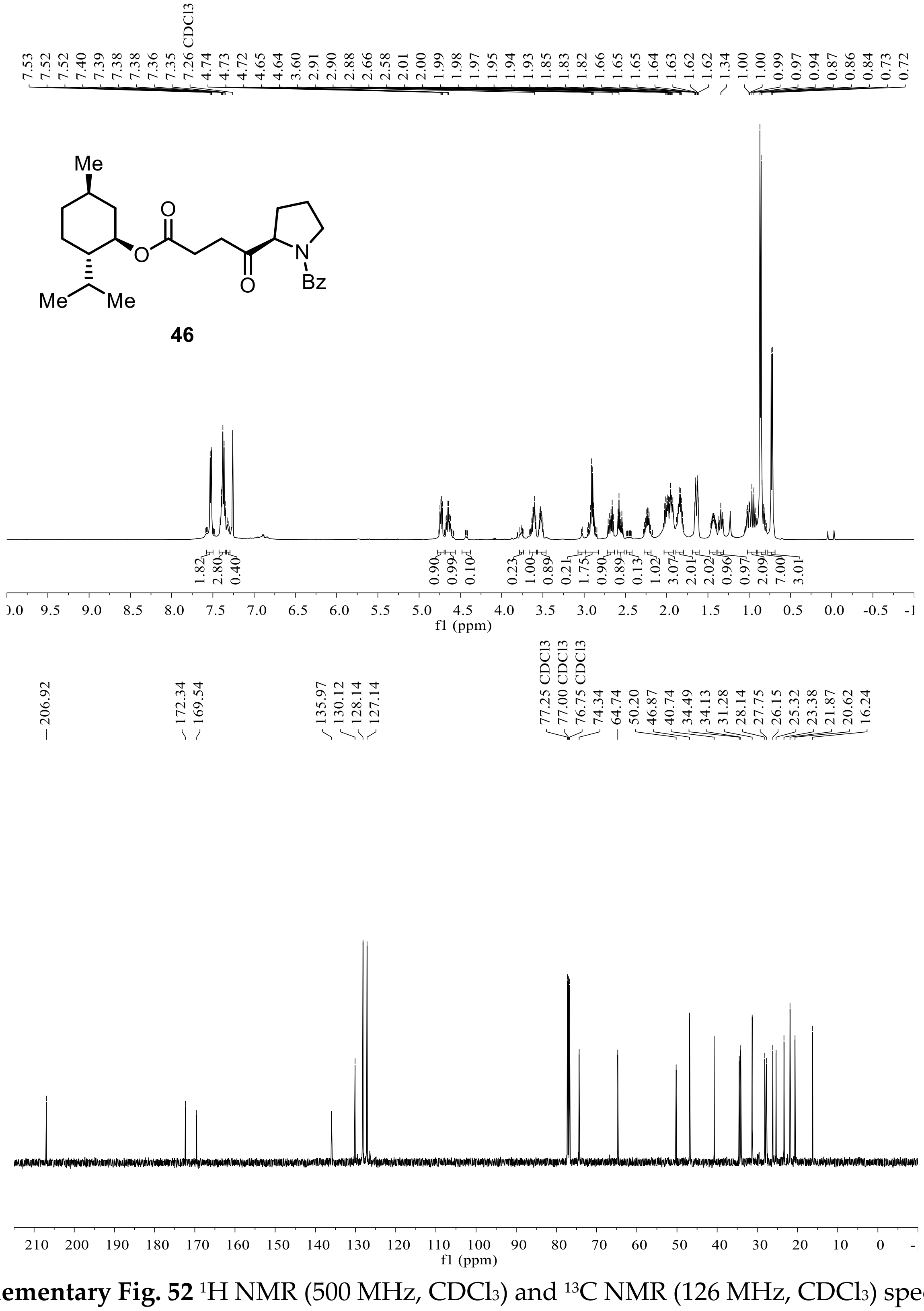

Supplementary Fig. 52 ^{1}H NMR (500 MHz, $CDCl_3$) and ^{13}C NMR (126 MHz, $CDCl_3$) spectrum of **46**.

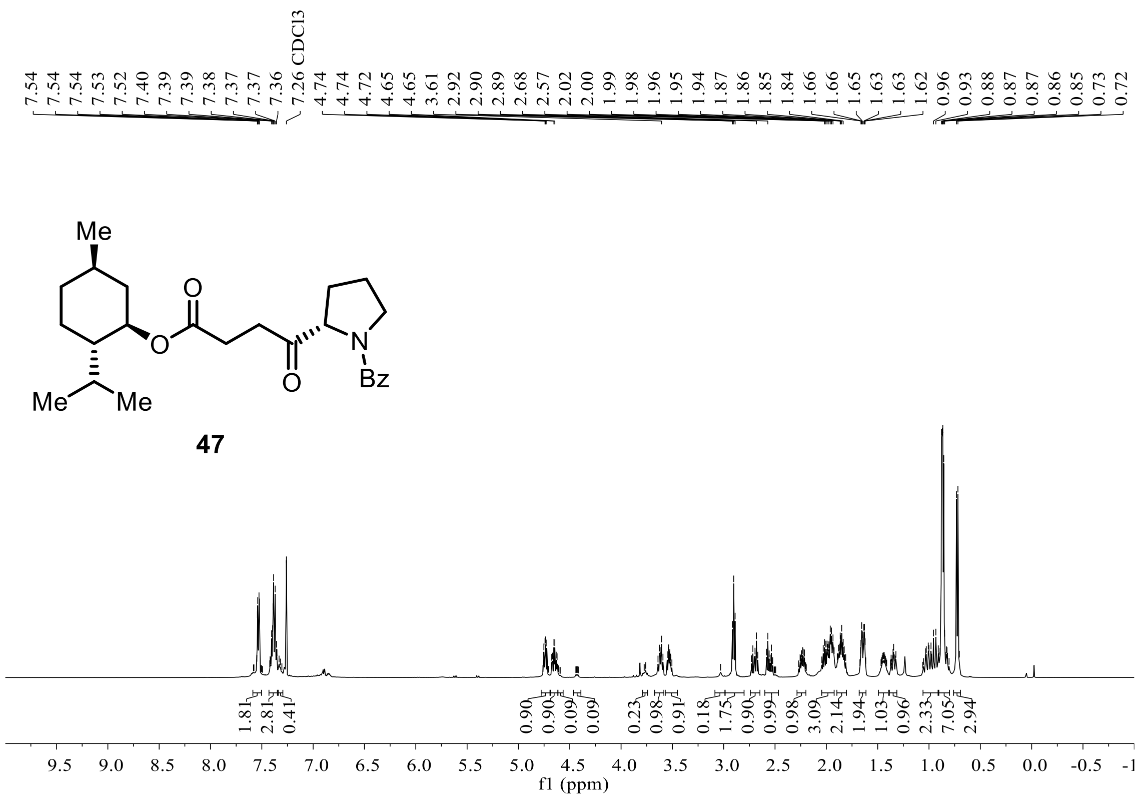

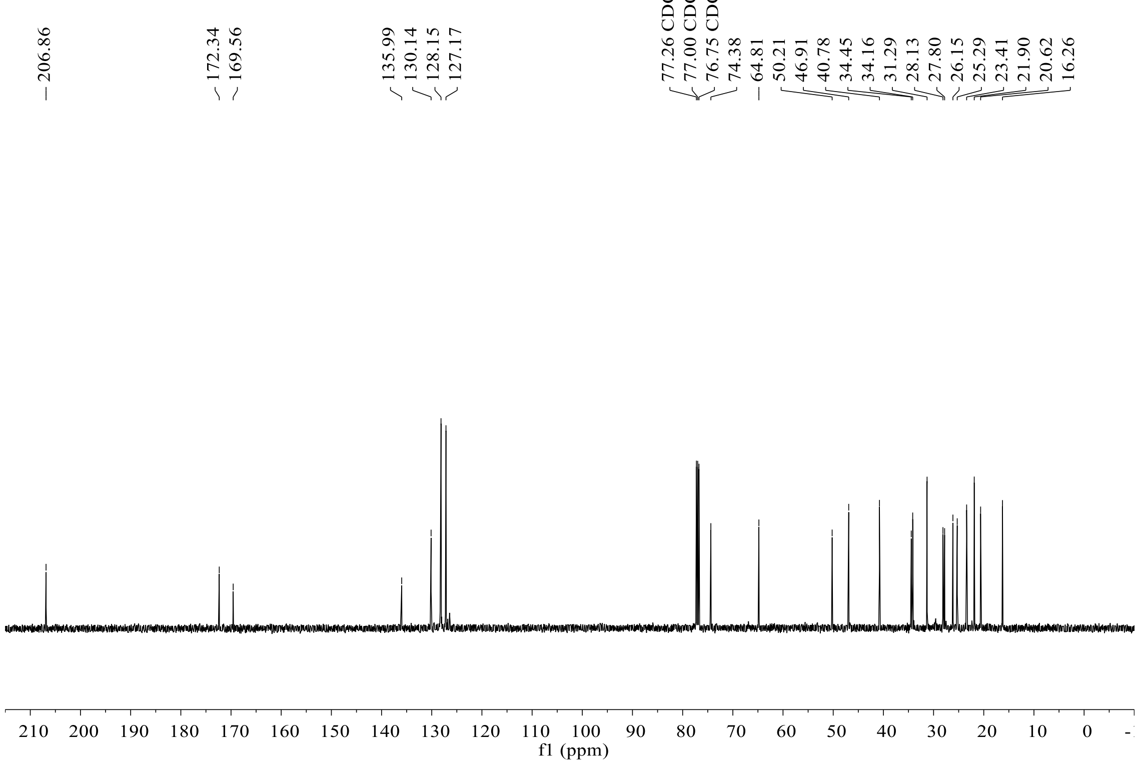

Supplementary Fig. 53 ^{1}H NMR (500 MHz, $CDCl_3$) and ^{13}C NMR (126 MHz, $CDCl_3$) spectrum of **47**.

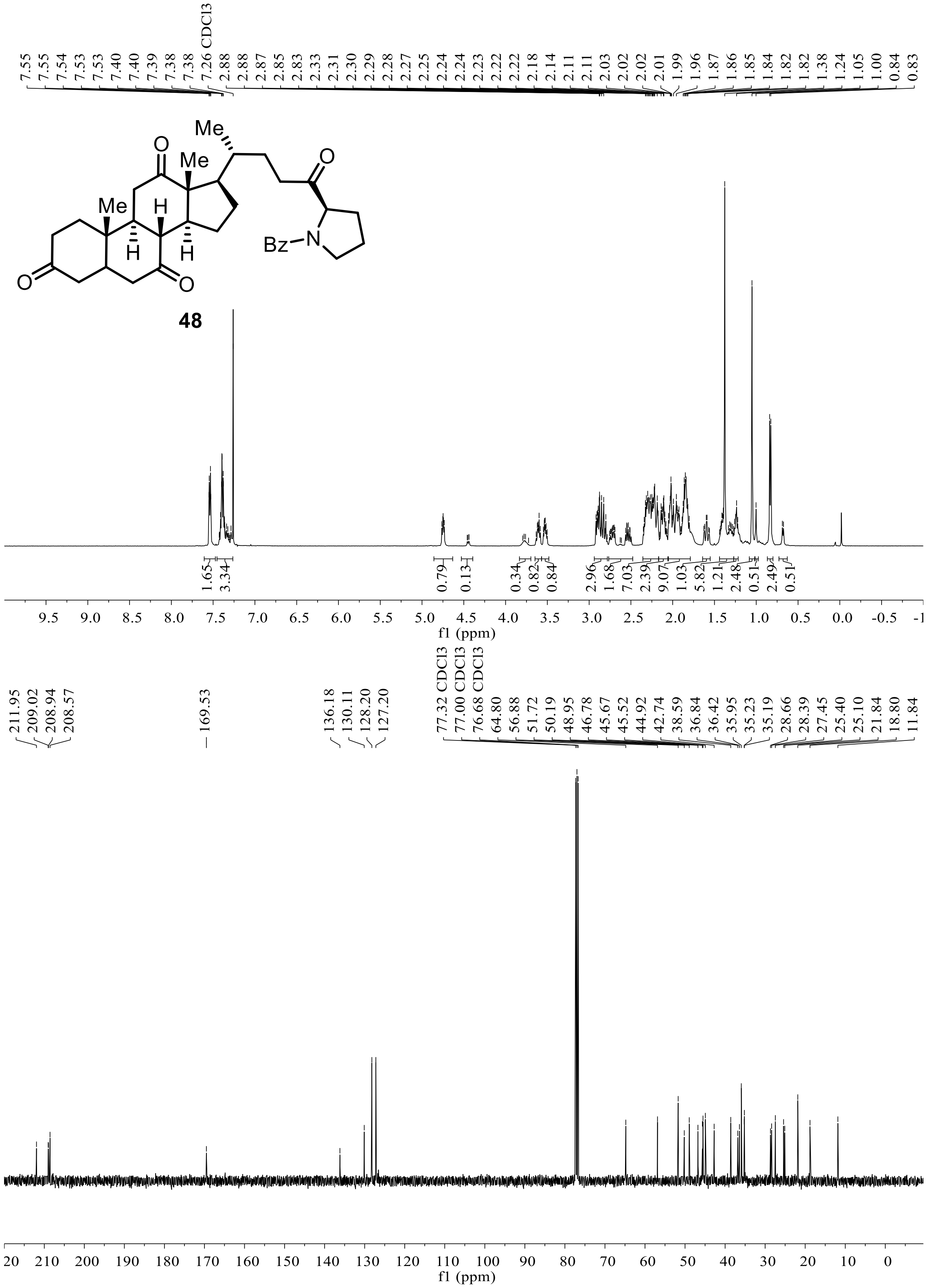

Supplementary Fig. 54 ^{1}H NMR (500 MHz, $CDCl_3$) and ^{13}C NMR (101 MHz, $CDCl_3$) spectrum of **48**.

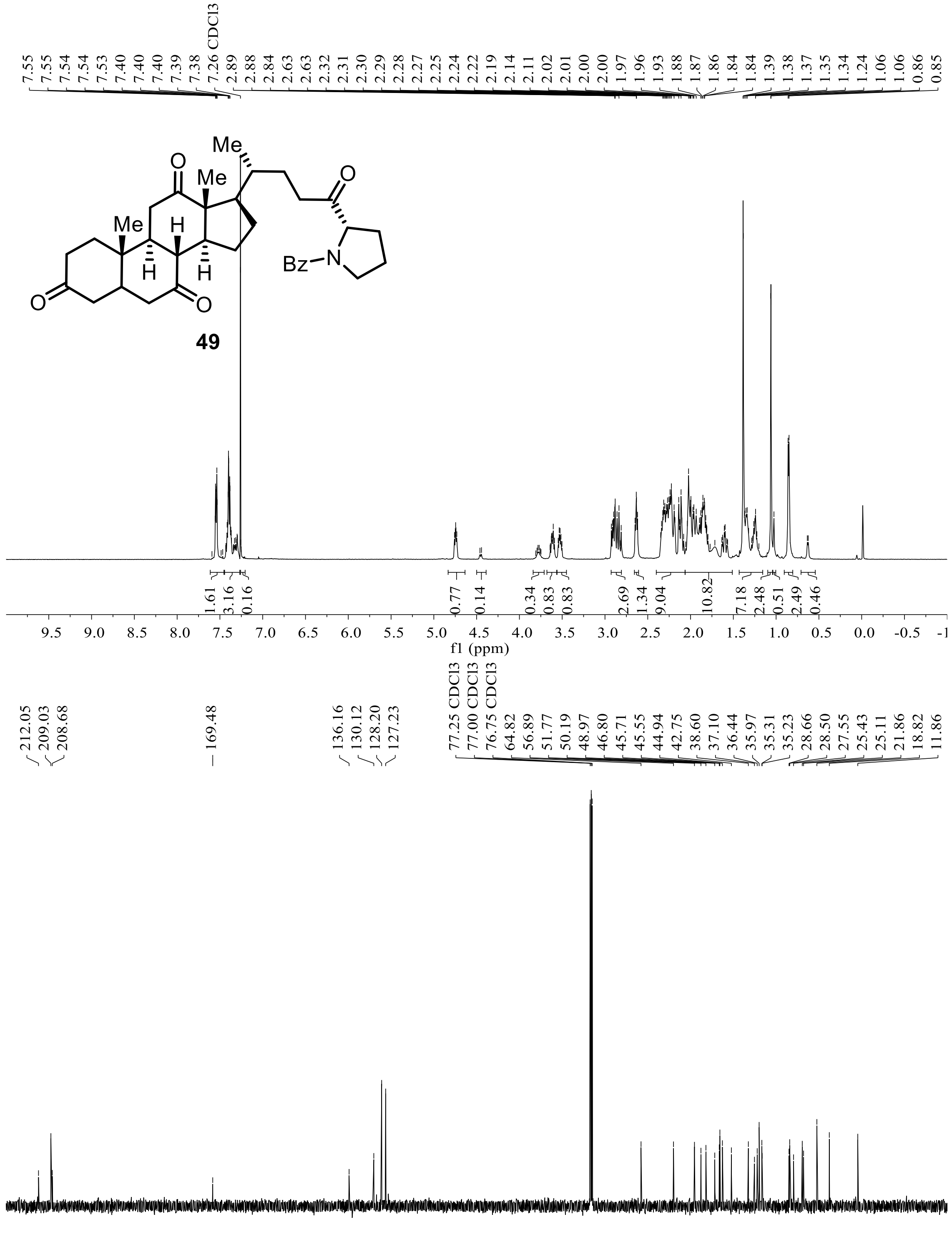

Supplementary Fig. 55 ^{1}H NMR (500 MHz, $CDCl_3$) and ^{13}C NMR (126 MHz, $CDCl_3$) spectrum of **49**.

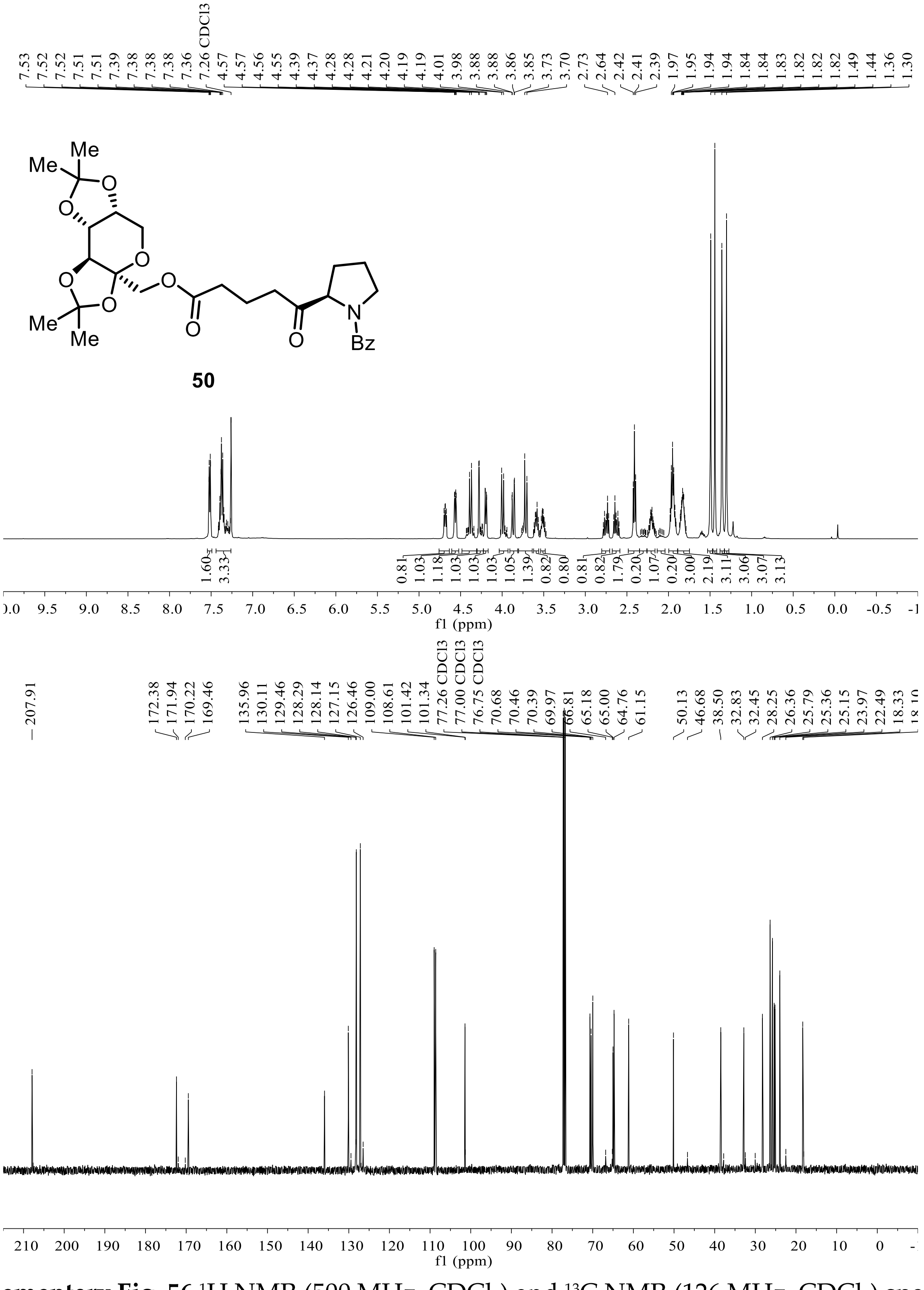

Supplementary Fig. 56 ^{1}H NMR (500 MHz, $CDCl_3$) and ^{13}C NMR (126 MHz, $CDCl_3$) spectrum of **50**.

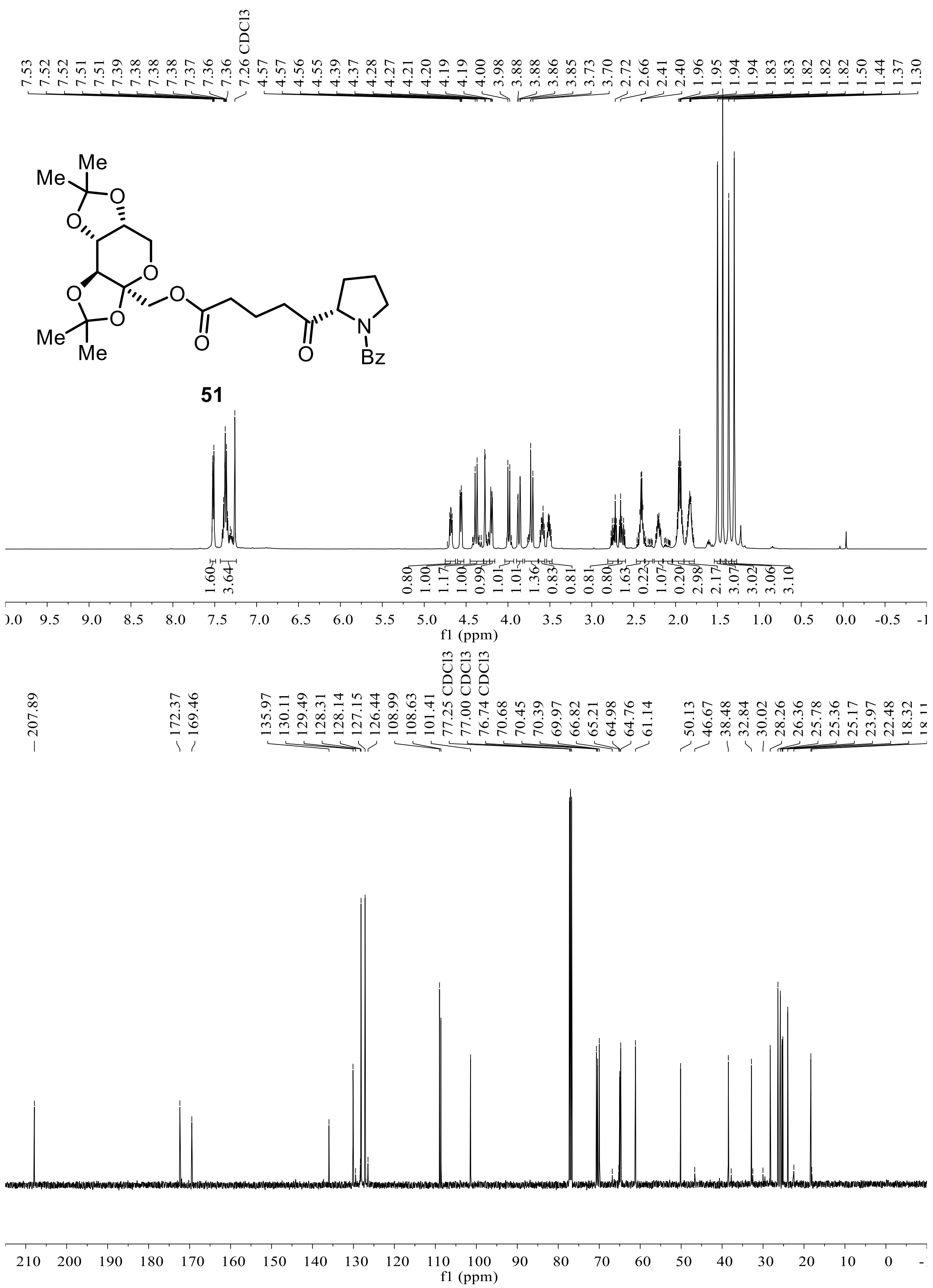

Supplementary Fig. 57 ^{1}H NMR (500 MHz, $CDCl_3$) and ^{13}C NMR (126 MHz, $CDCl_3$) spectrum of **51**.

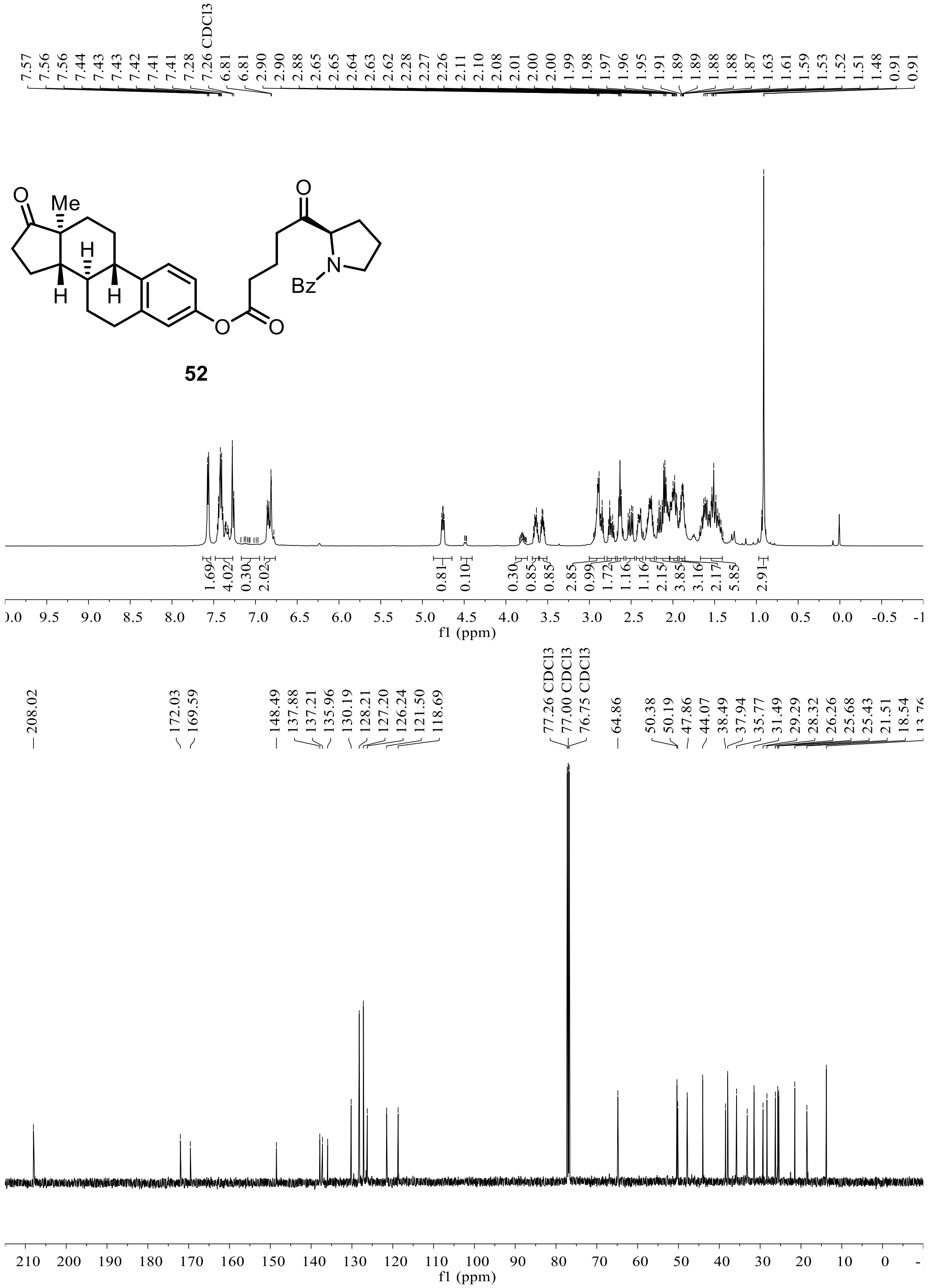

Supplementary Fig. 58 ^{1}H NMR (500 MHz, $CDCl_3$) and ^{13}C NMR (126 MHz, $CDCl_3$) spectrum of **52**.

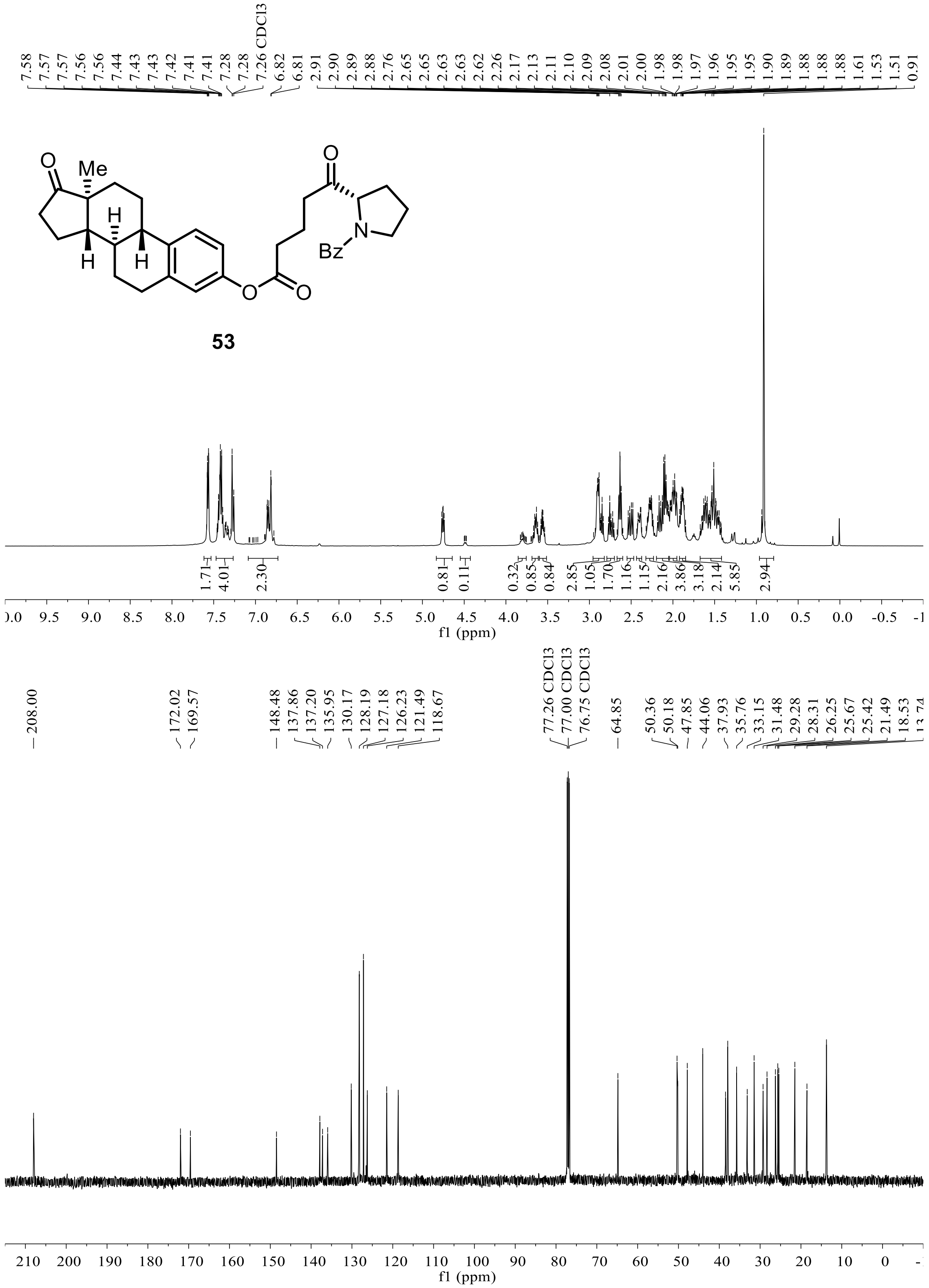

Supplementary Fig. 59 ^{1}H NMR (500 MHz, $CDCl_3$) and ^{13}C NMR (126 MHz, $CDCl_3$) spectrum of **53**.

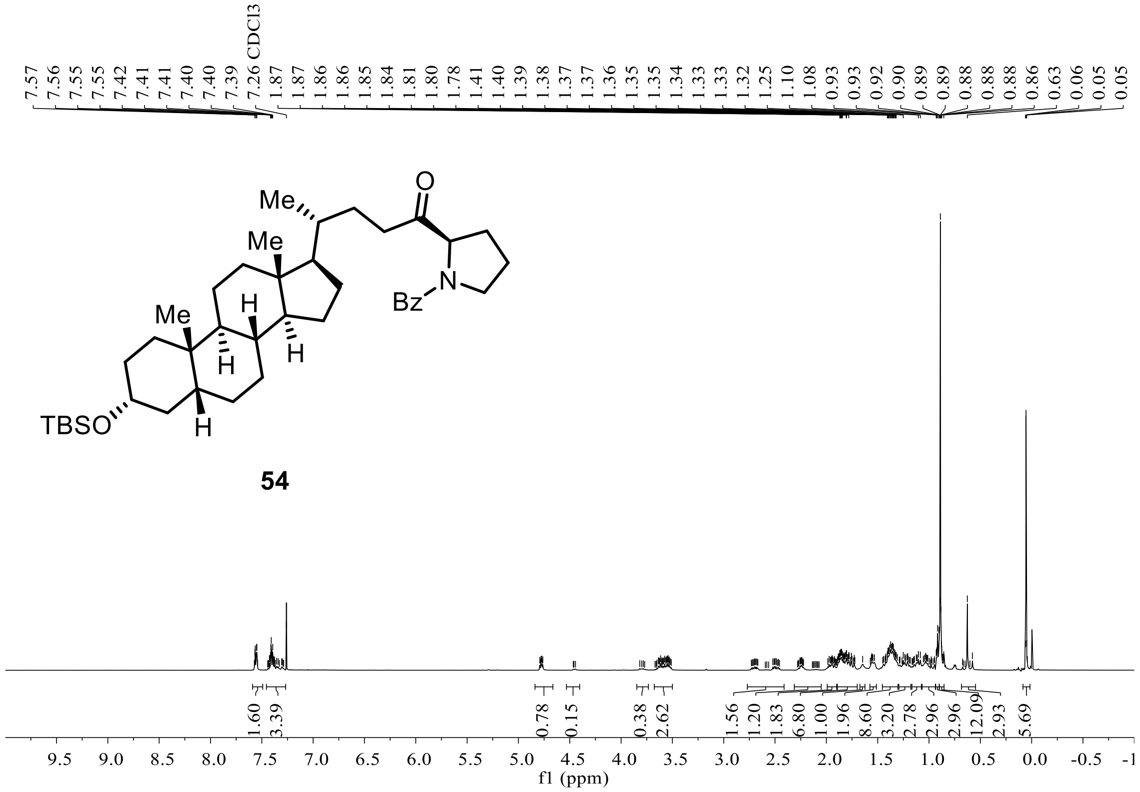

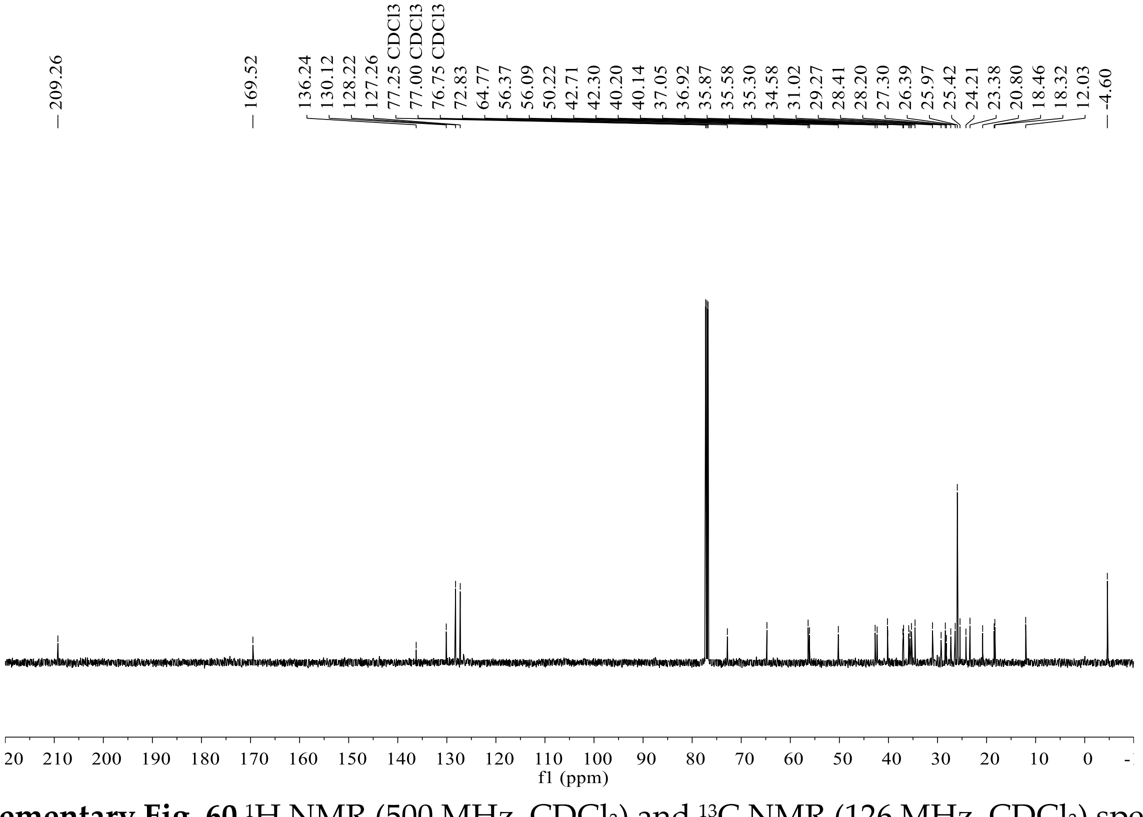

Supplementary Fig. 60 ^{1}H NMR (500 MHz, $CDCl_3$) and ^{13}C NMR (126 MHz, $CDCl_3$) spectrum of **54**.

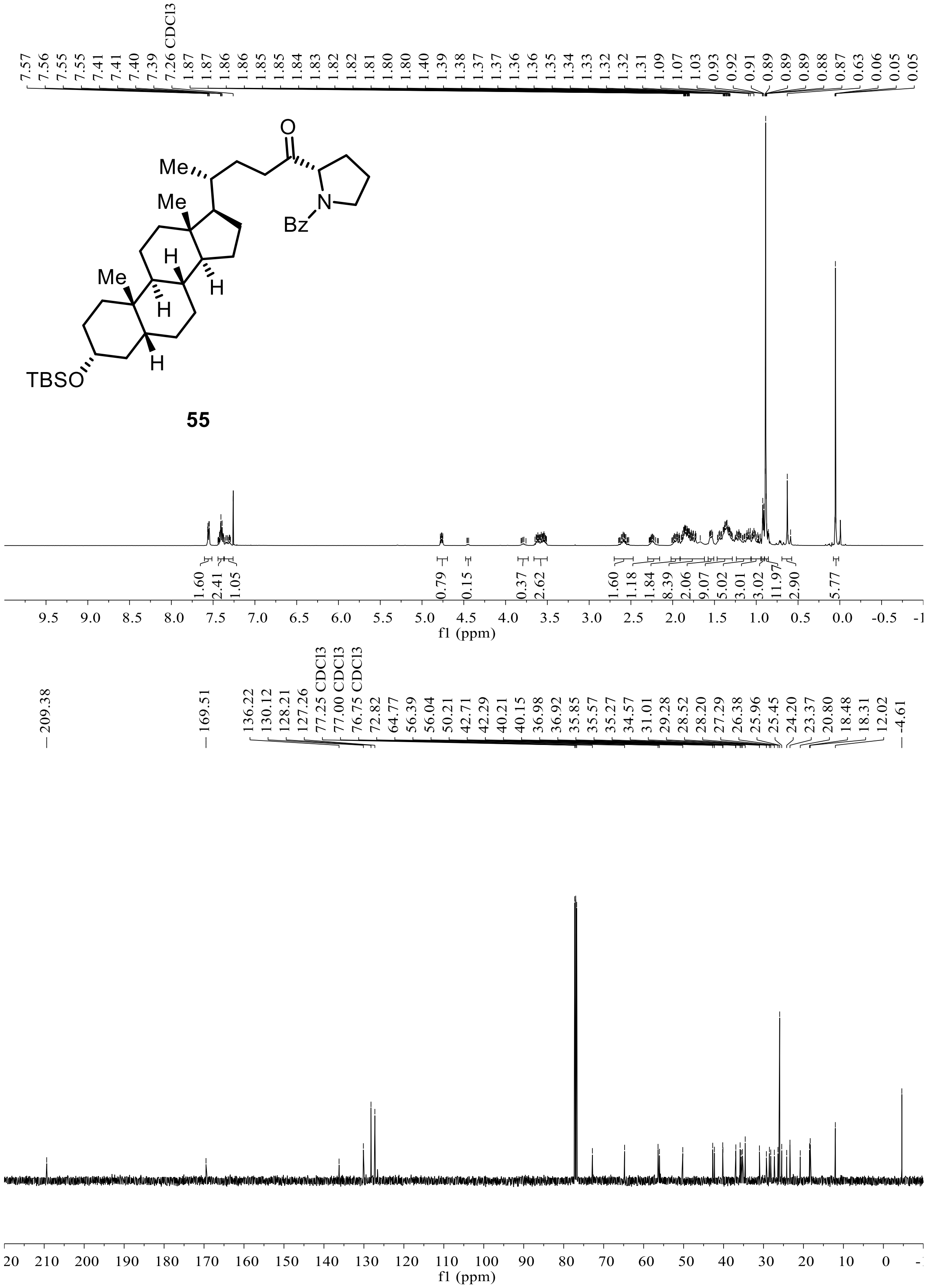

Supplementary Fig. 61 ^{1}H NMR (500 MHz, $CDCl_3$) and ^{13}C NMR (126 MHz, $CDCl_3$) spectrum of **55**.

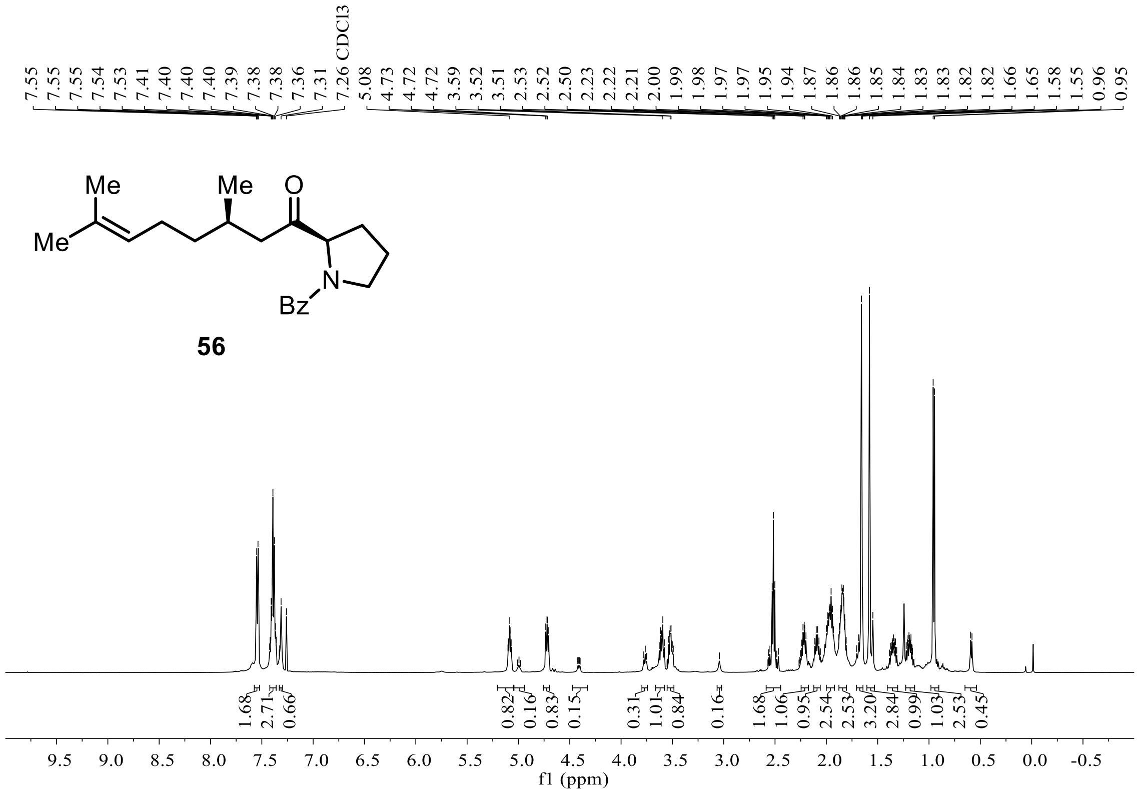

56

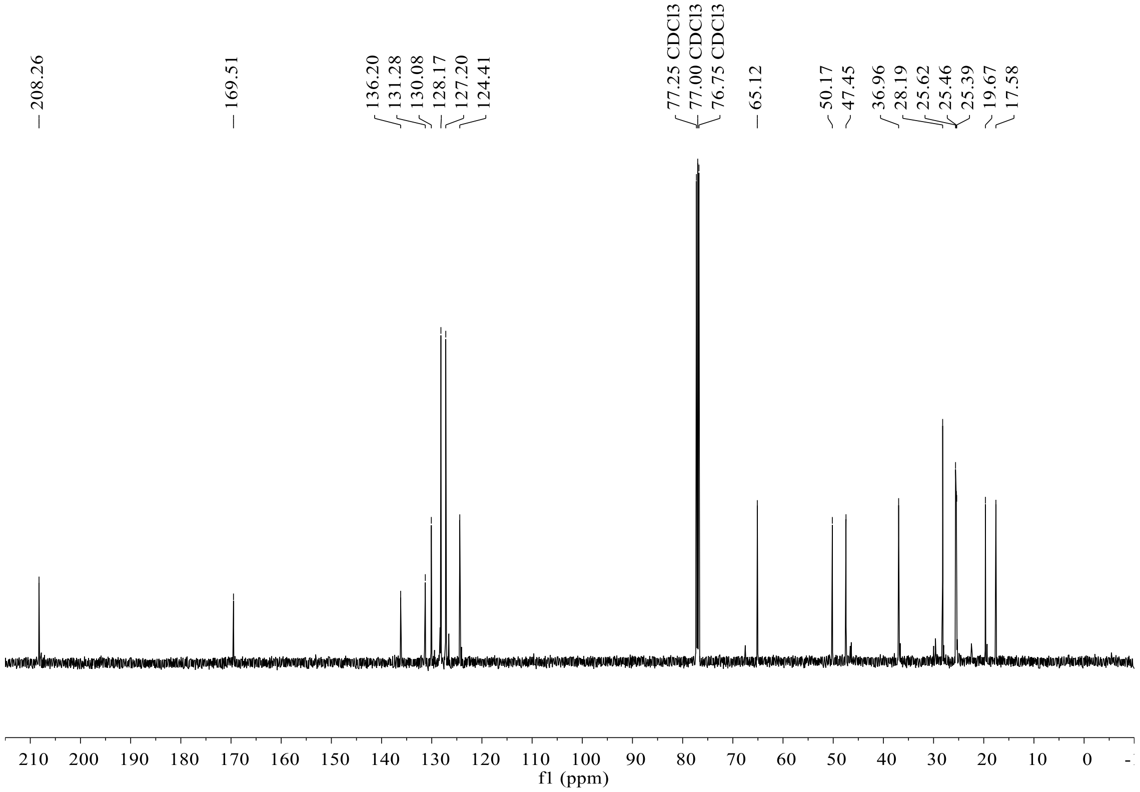

Supplementary Fig. 62 ^{1}H NMR (500 MHz, $CDCl_3$) and ^{13}C NMR (126 MHz, $CDCl_3$) spectrum of **56**.

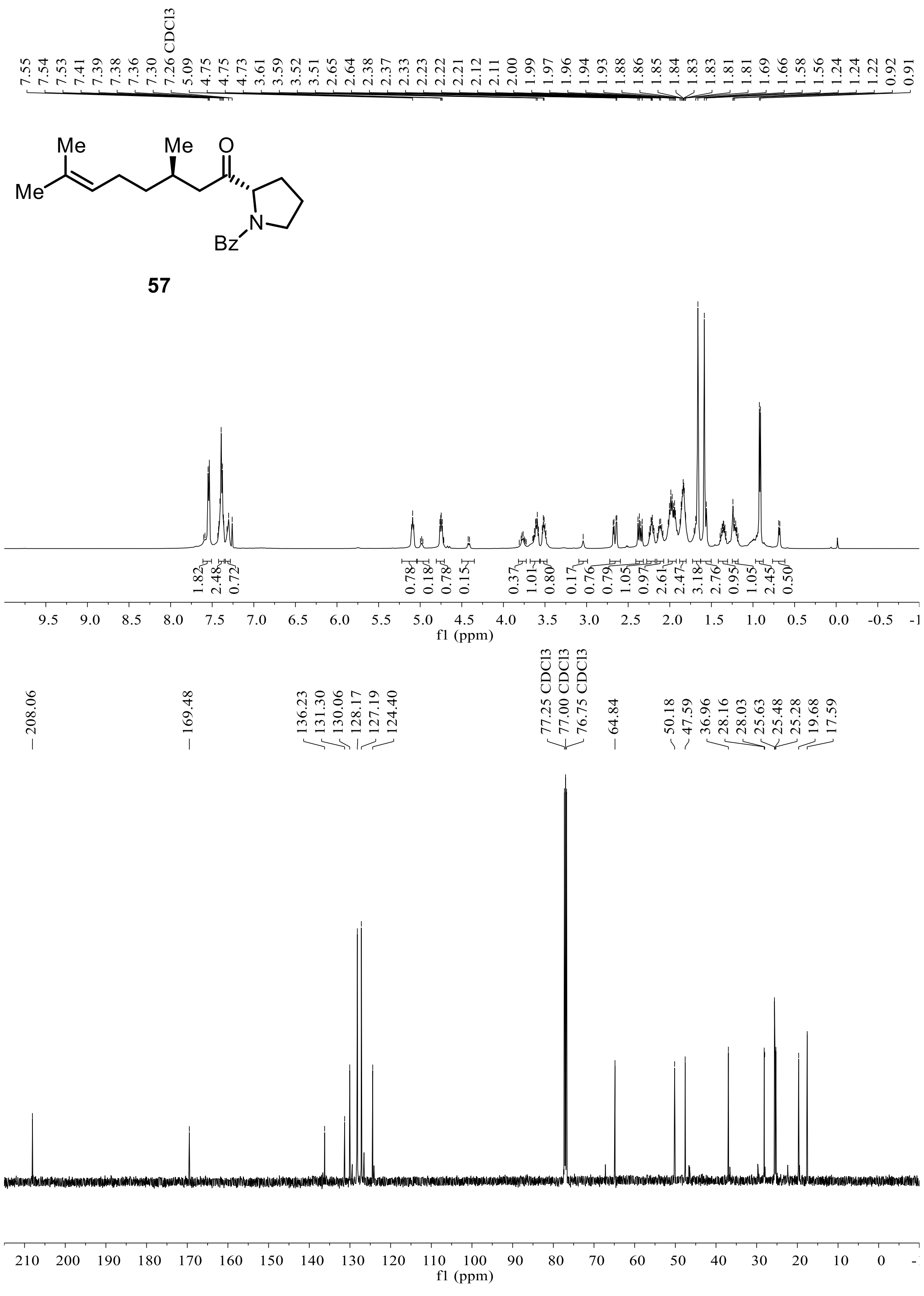

Supplementary Fig. 63 ^{1}H NMR (500 MHz, $CDCl_3$) and ^{13}C NMR (126 MHz, $CDCl_3$) spectrum of **57**.

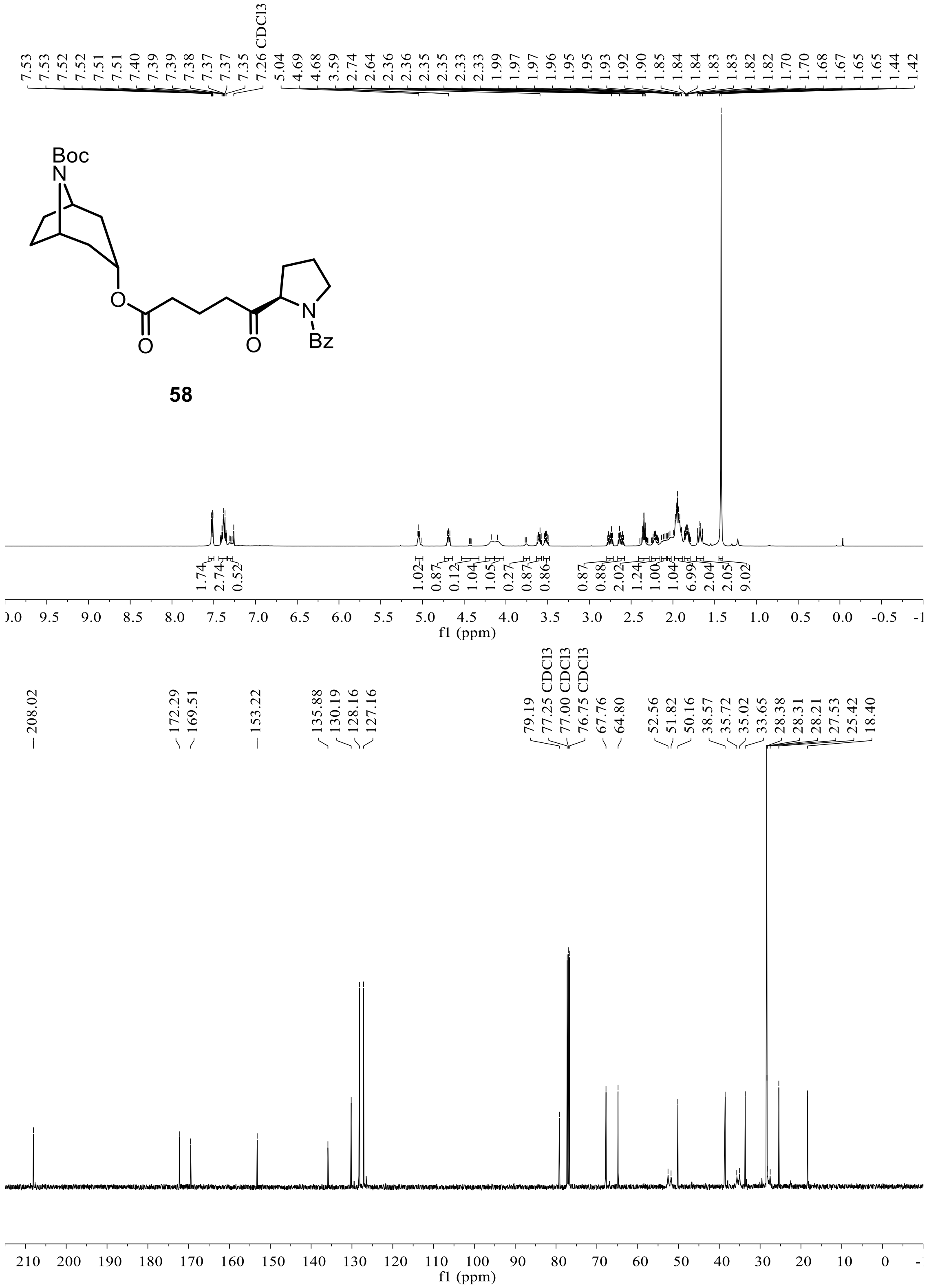

Supplementary Fig. 64 ¹H NMR (500 MHz, CDCl₃) and ¹³C NMR (126 MHz, CDCl₃) spectrum of 58.

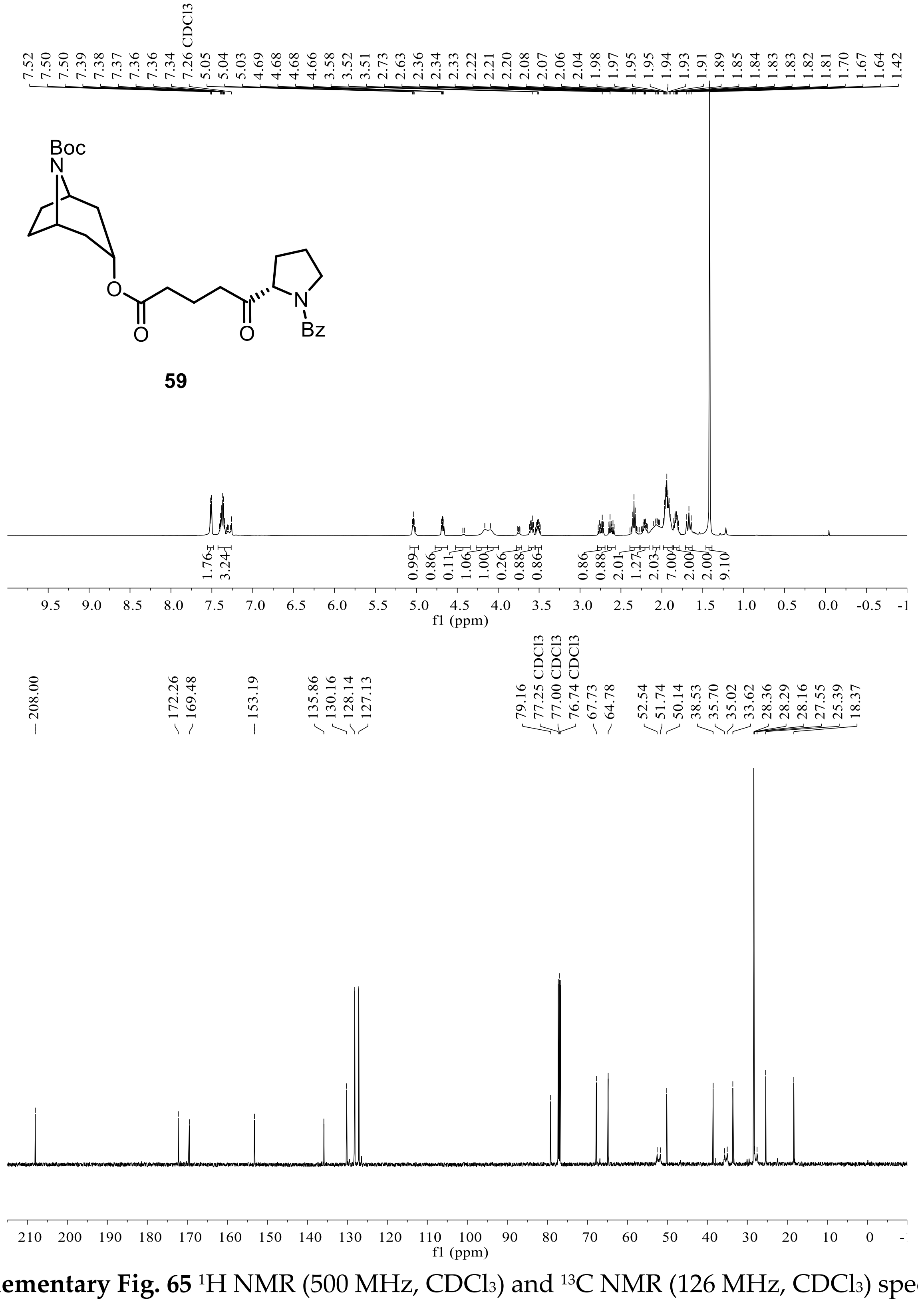

Supplementary Fig. 65 ^{1}H NMR (500 MHz, $CDCl_3$) and ^{13}C NMR (126 MHz, $CDCl_3$) spectrum of **59**.

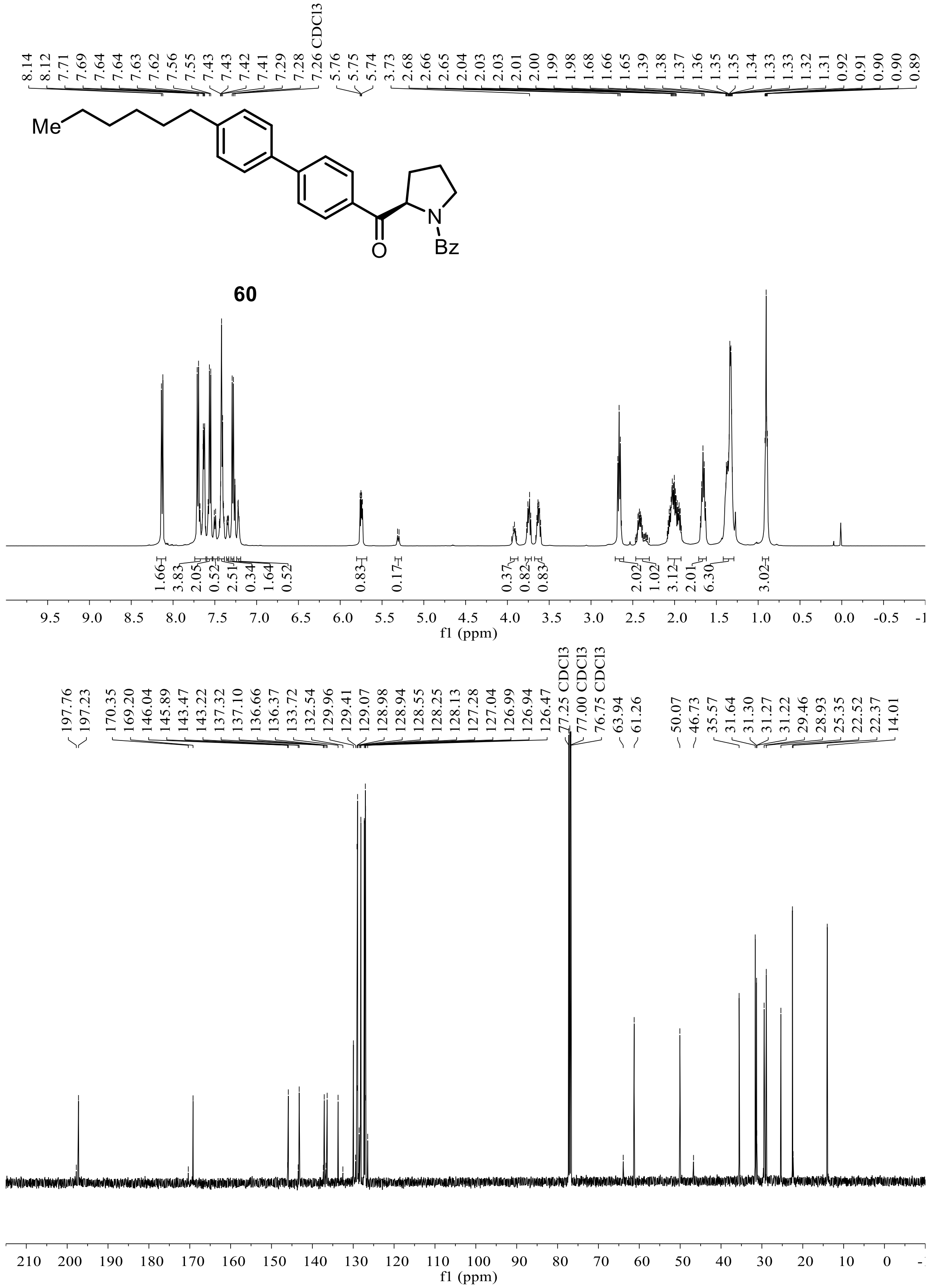

Supplementary Fig. 66 ^{1}H NMR (500 MHz, $CDCl_3$) and ^{13}C NMR (126 MHz, $CDCl_3$) spectrum of **60**.

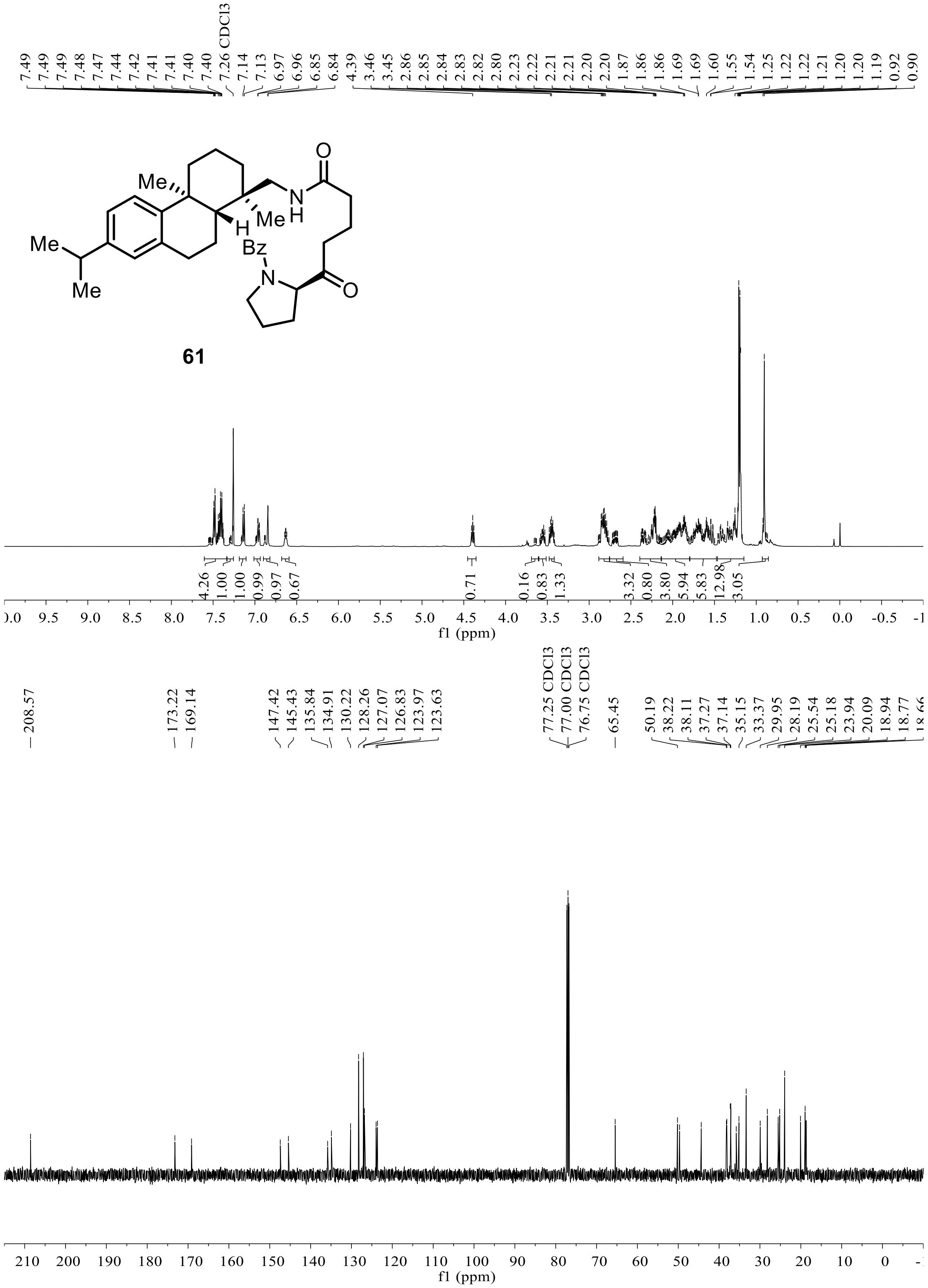

Supplementary Fig. 67 ^{1}H NMR (500 MHz, $CDCl_3$) and ^{13}C NMR (126 MHz, $CDCl_3$) spectrum of **61**.

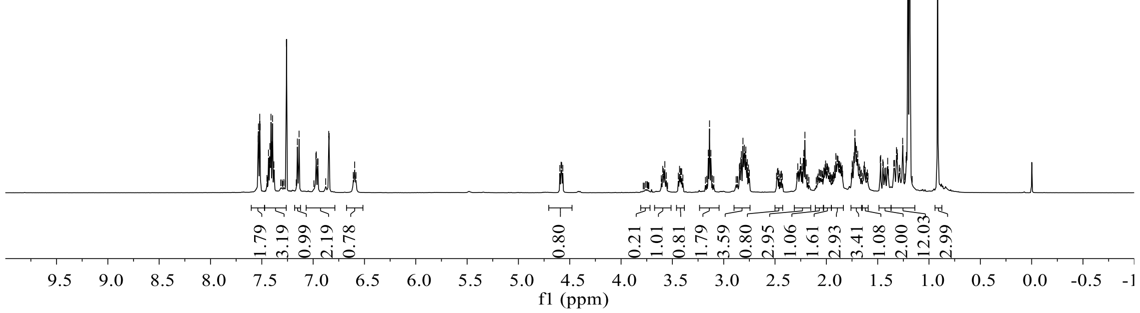

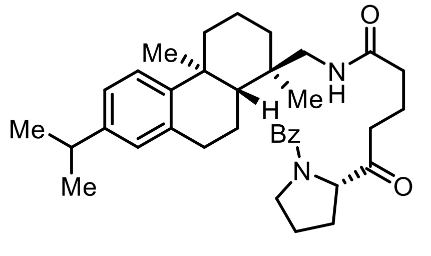

62

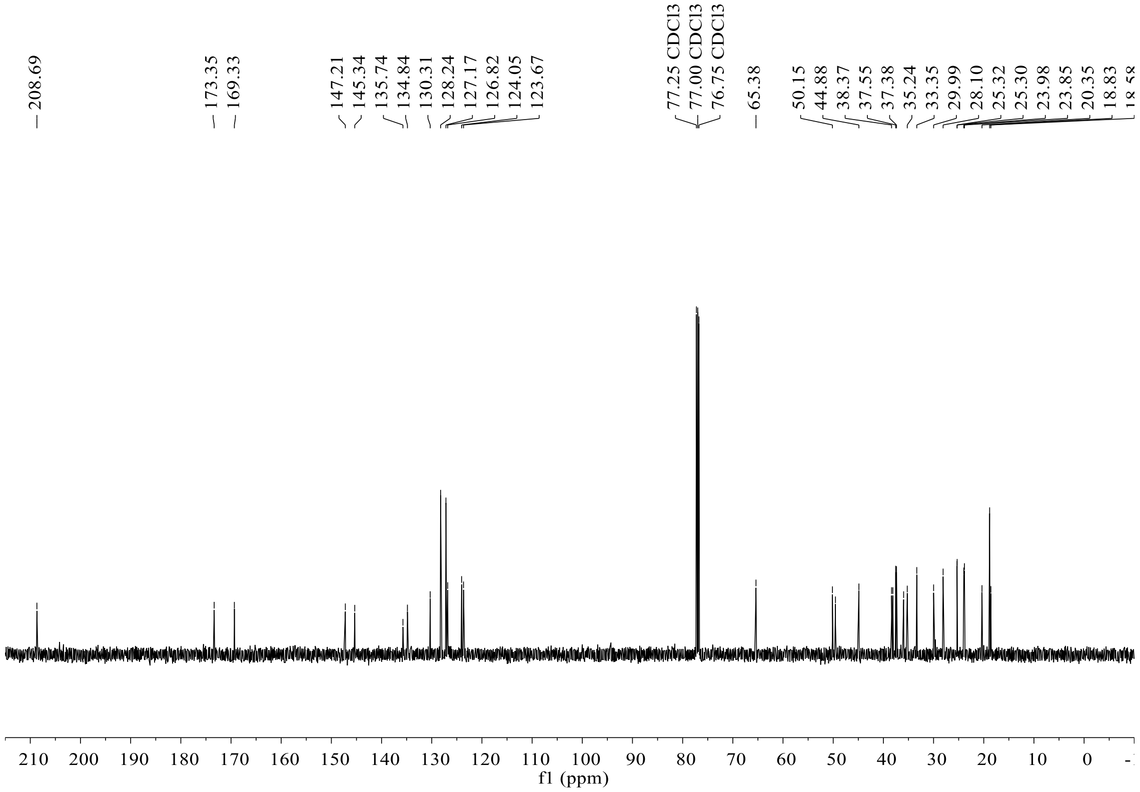

Supplementary Fig. 68 ^{1}H NMR (500 MHz, $CDCl_3$) and ^{13}C NMR (126 MHz, $CDCl_3$) spectrum of **62**.

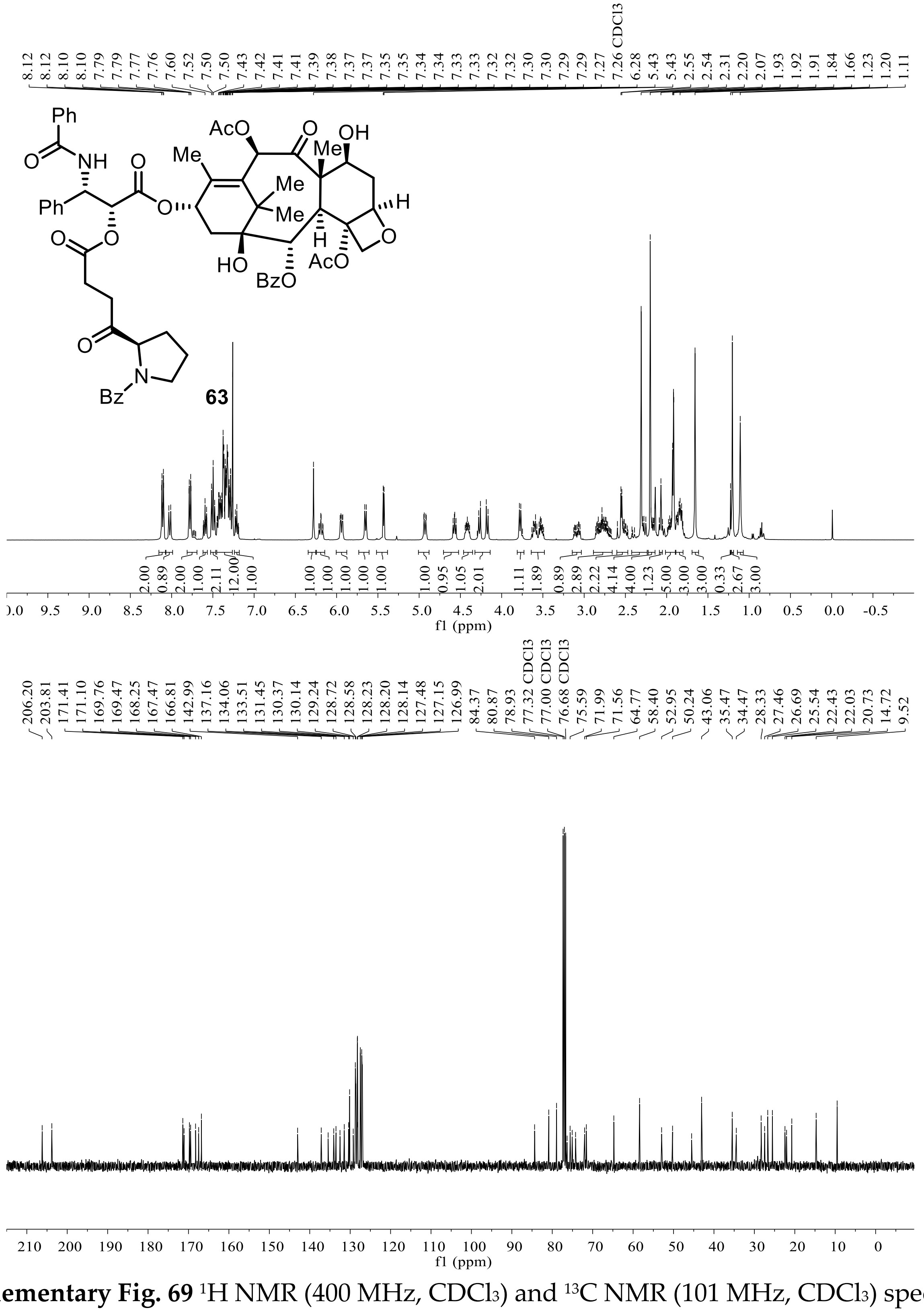

Supplementary Fig. 69 ^{1}H NMR (400 MHz, $CDCl_3$) and ^{13}C NMR (101 MHz, $CDCl_3$) spectrum of **63**.

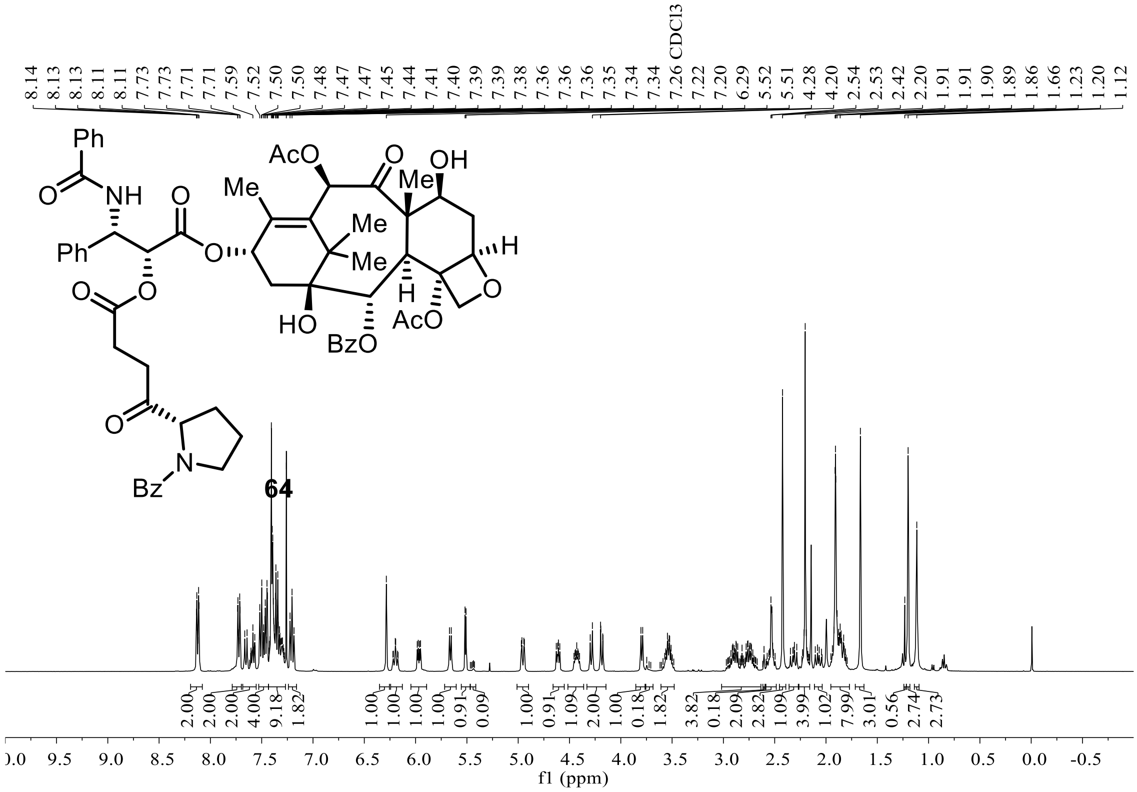

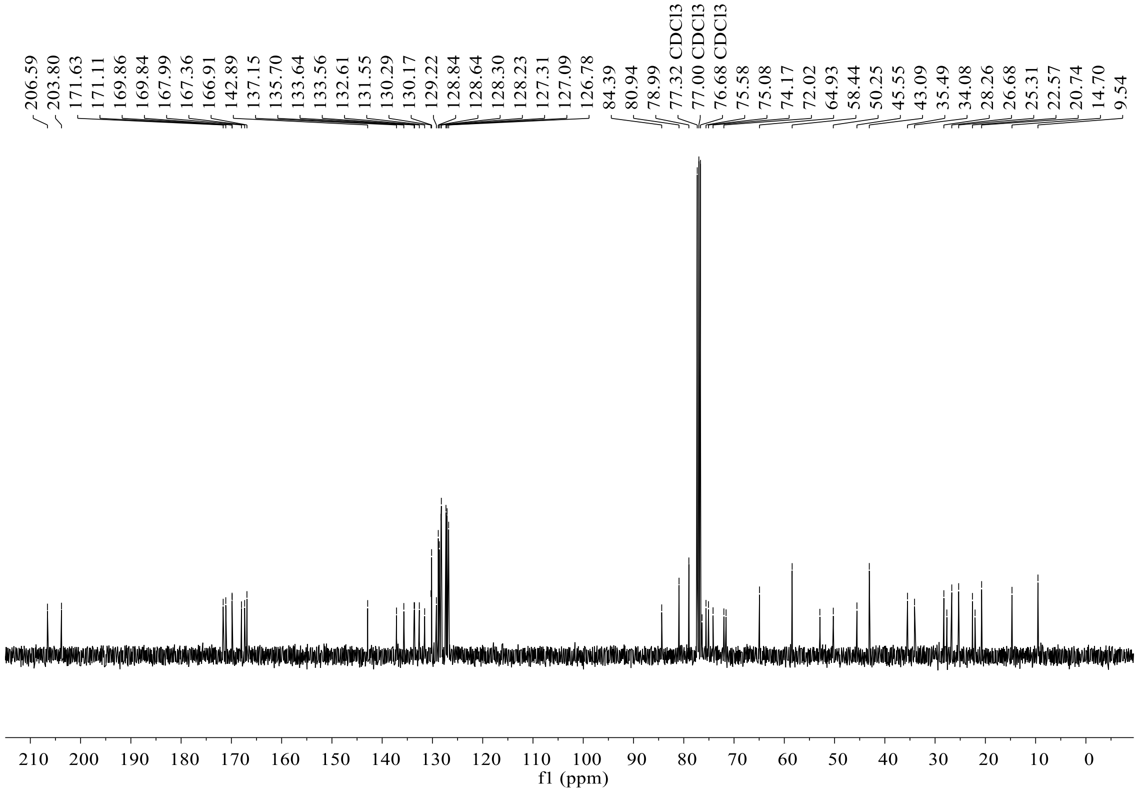

Supplementary Fig. 70 ^{1}H NMR (400 MHz, $CDCl_3$) and ^{13}C NMR (101 MHz, $CDCl_3$) spectrum of **64**.

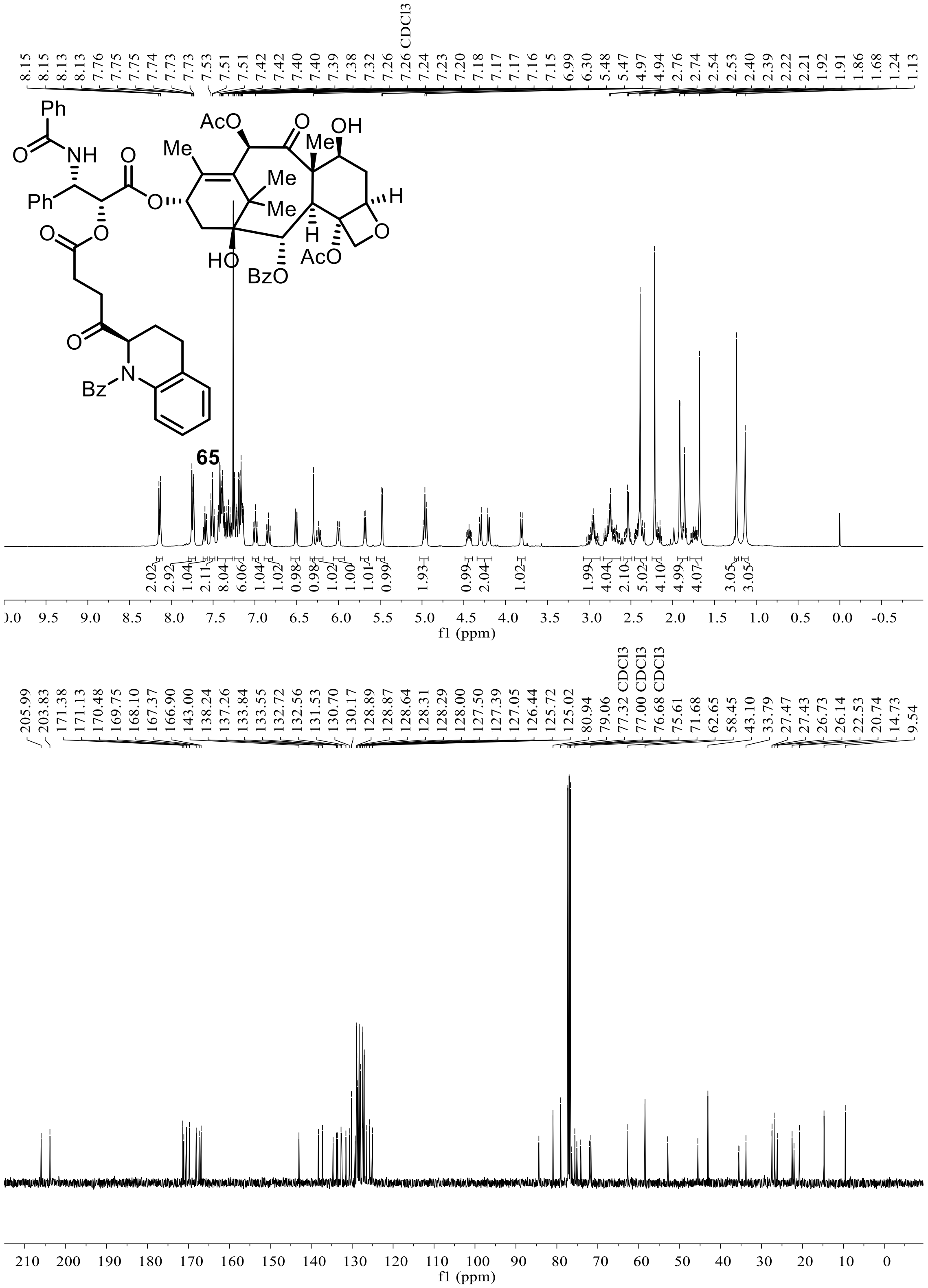

Supplementary Fig. 71 ^{1}H NMR (400 MHz, $CDCl_3$) and ^{13}C NMR (101 MHz, $CDCl_3$) spectrum of **65**.

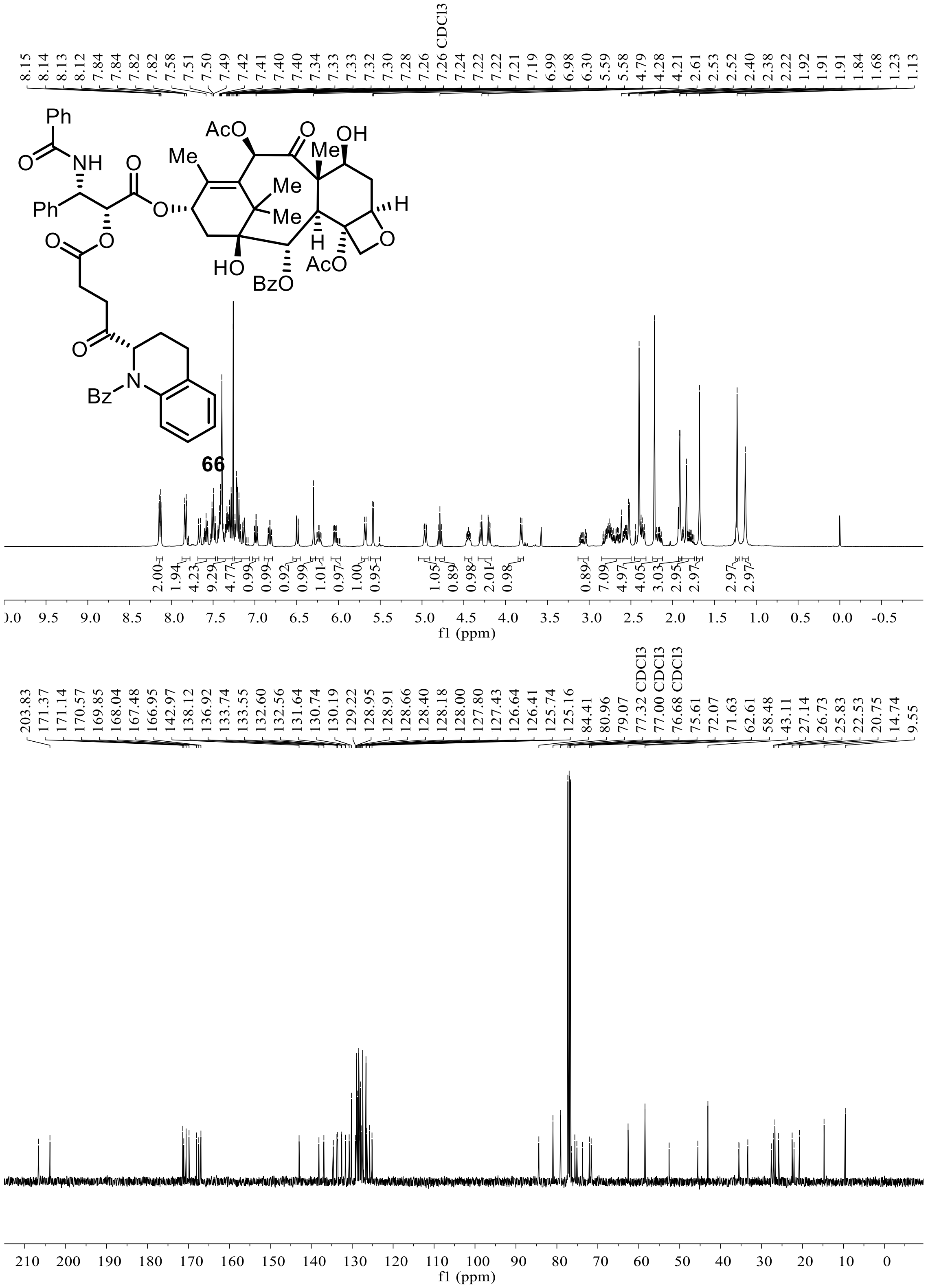

Supplementary Fig. 72 ^{1}H NMR (400 MHz, $CDCl_3$) and ^{13}C NMR (101 MHz, $CDCl_3$) spectrum of **66**.

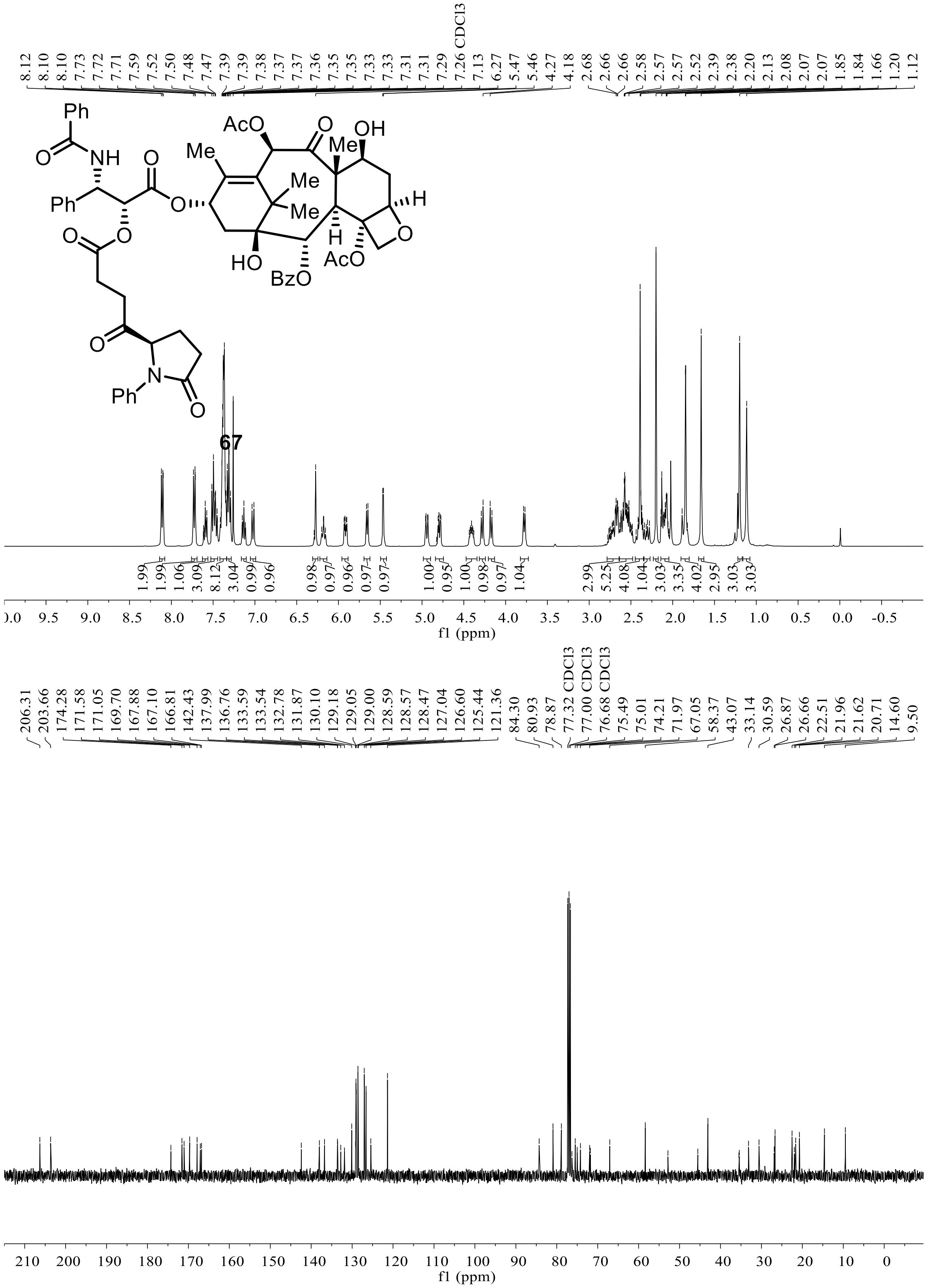

Supplementary Fig. 73 ^{1}H NMR (400 MHz, $CDCl_3$) and ^{13}C NMR (101 MHz, $CDCl_3$) spectrum of **67**.

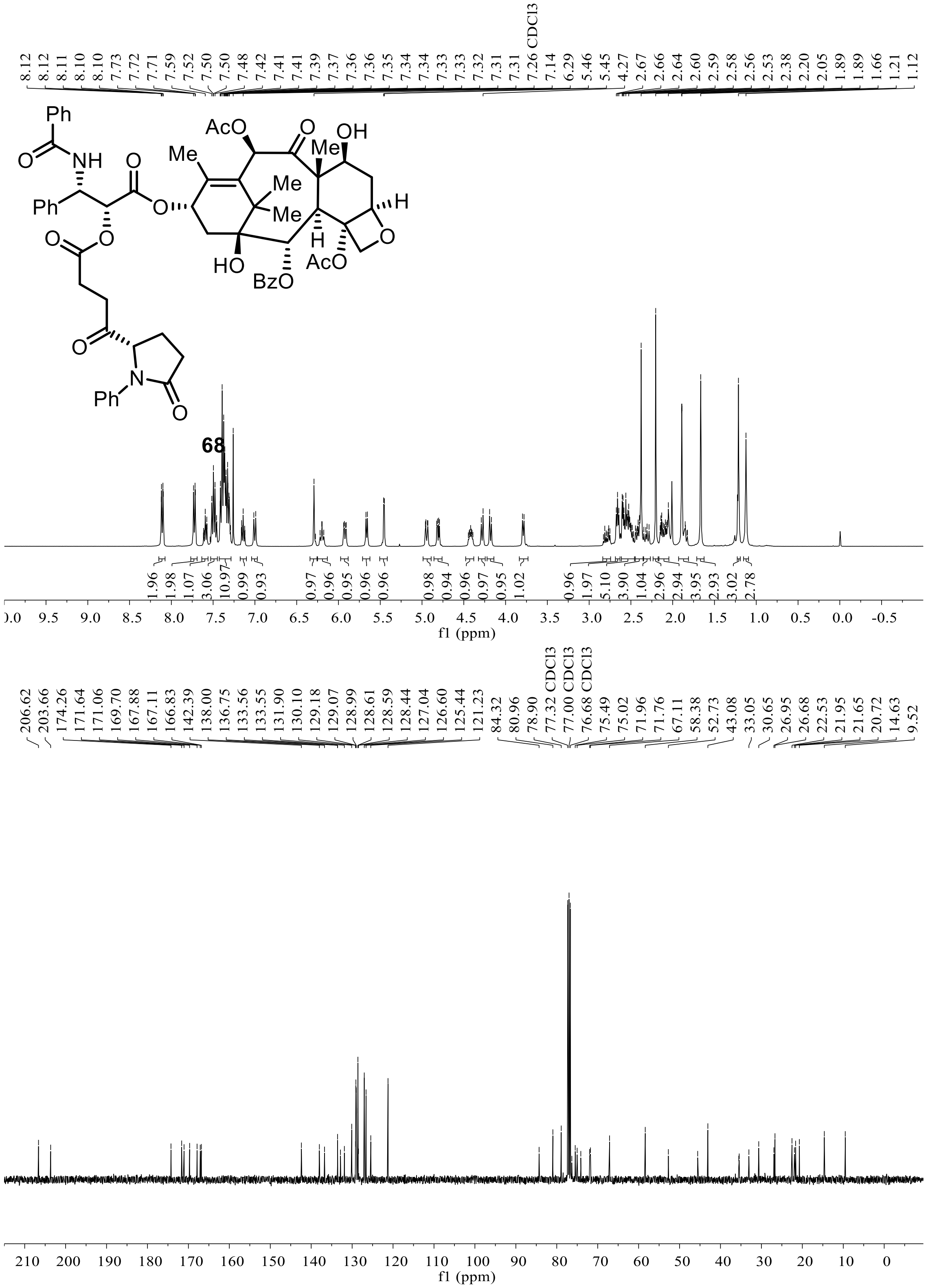

Supplementary Fig. 74 ^{1}H NMR (400 MHz, $CDCl_3$) and ^{13}C NMR (101 MHz, $CDCl_3$) spectrum of **68**.

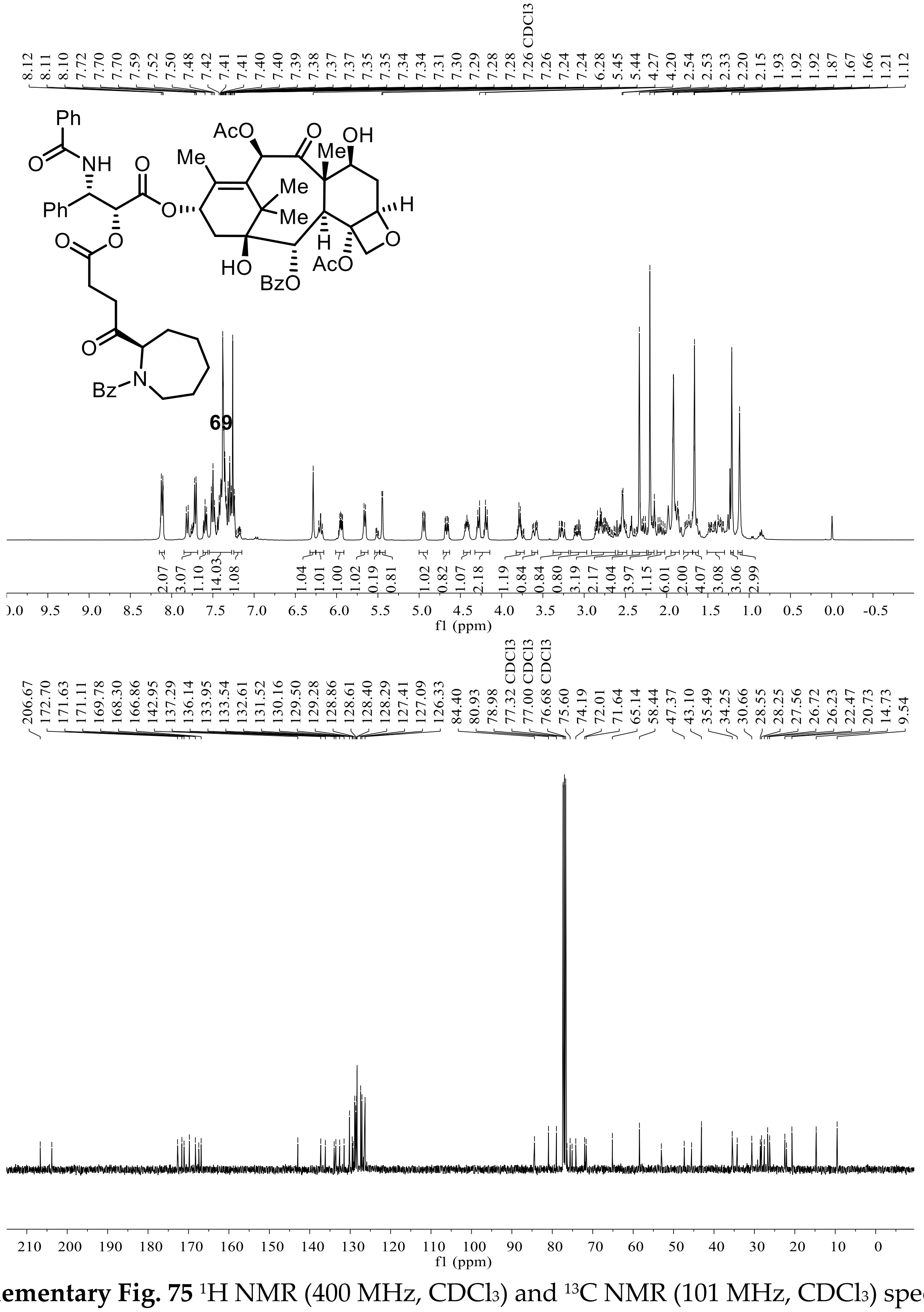

Supplementary Fig. 75 ^{1}H NMR (400 MHz, $CDCl_3$) and ^{13}C NMR (101 MHz, $CDCl_3$) spectrum of **69**.

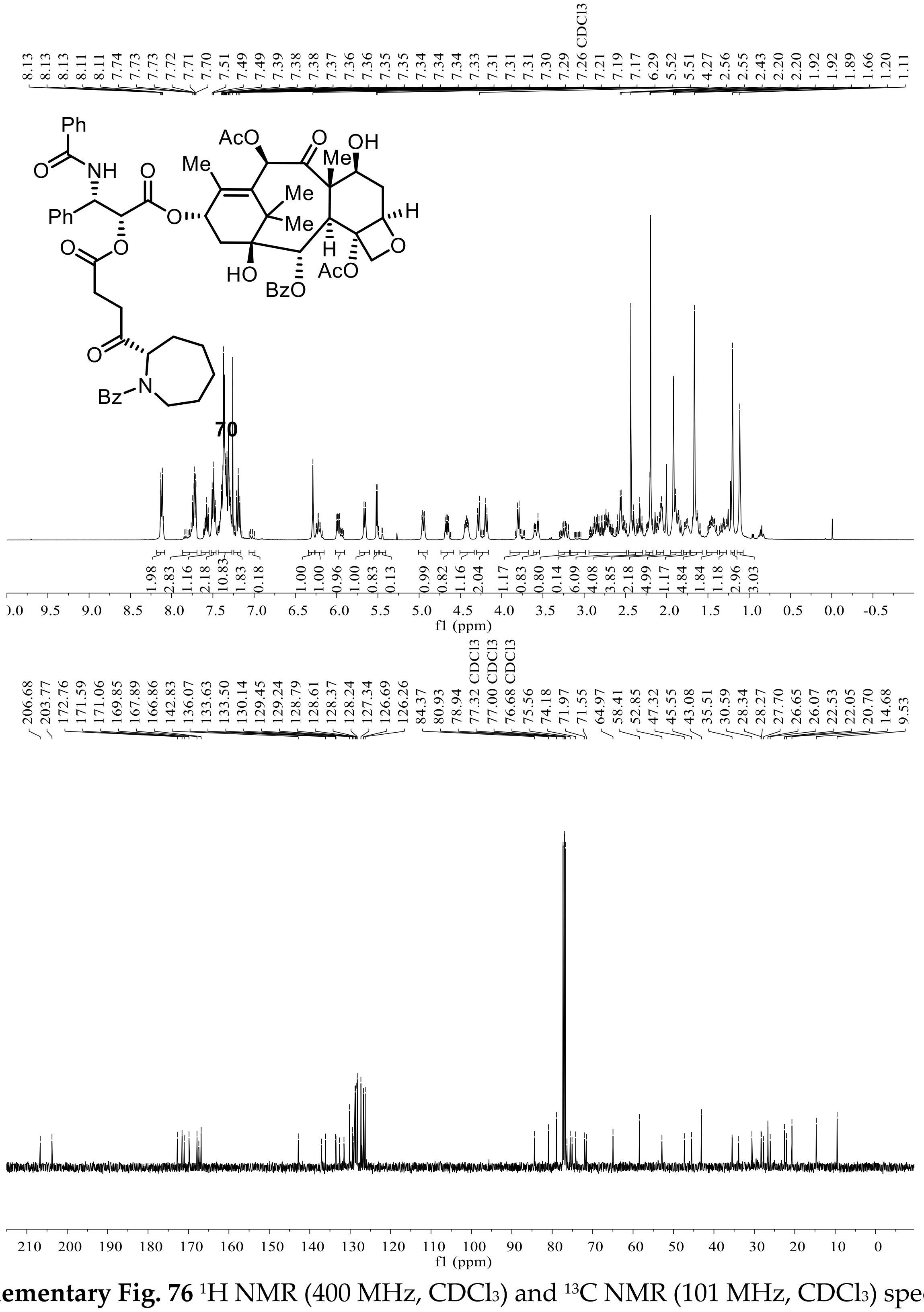

Supplementary Fig. 76 ^{1}H NMR (400 MHz, $CDCl_3$) and ^{13}C NMR (101 MHz, $CDCl_3$) spectrum of **70**.

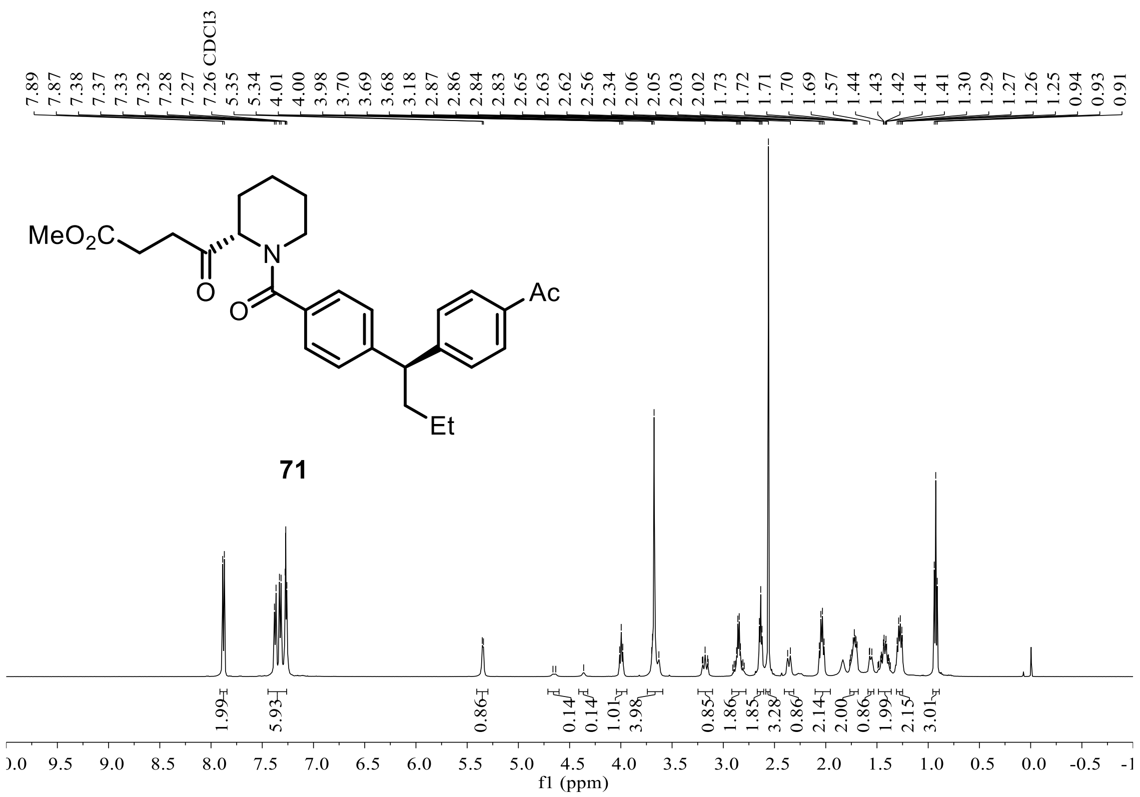

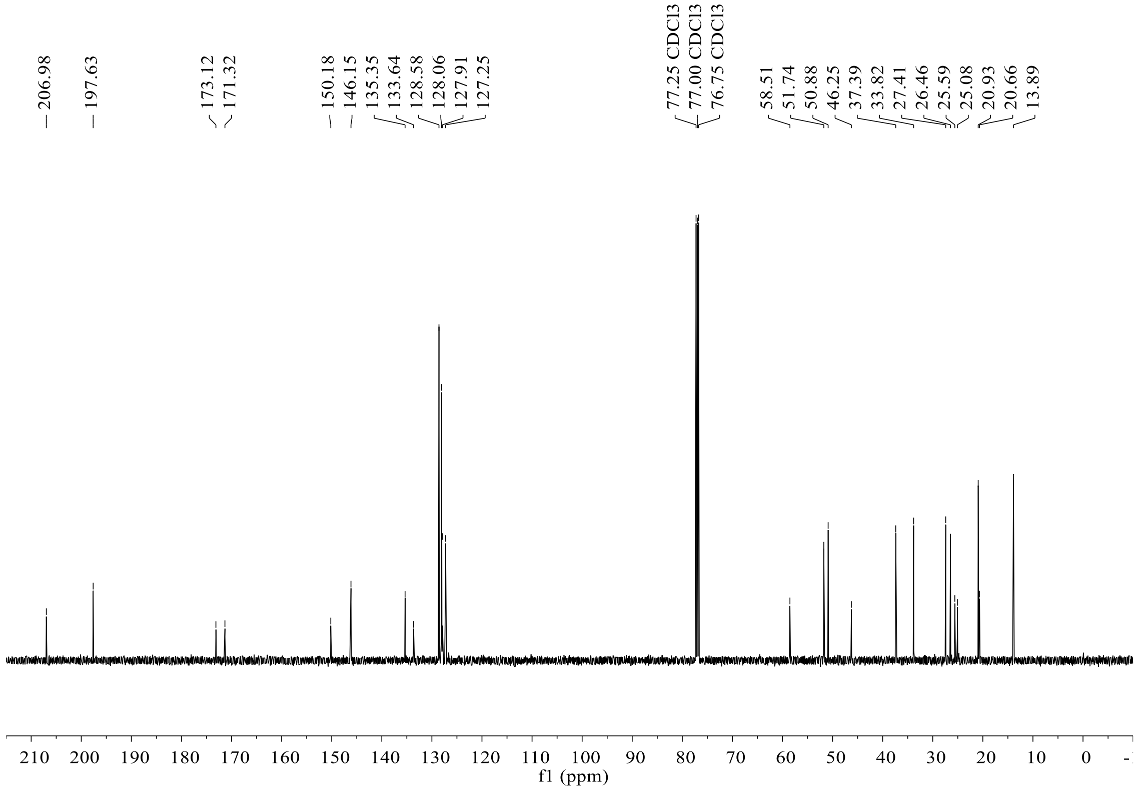

Supplementary Fig. 77 ^{1}H NMR (500 MHz, $CDCl_3$) and ^{13}C NMR (126 MHz, $CDCl_3$) spectrum of **71**.

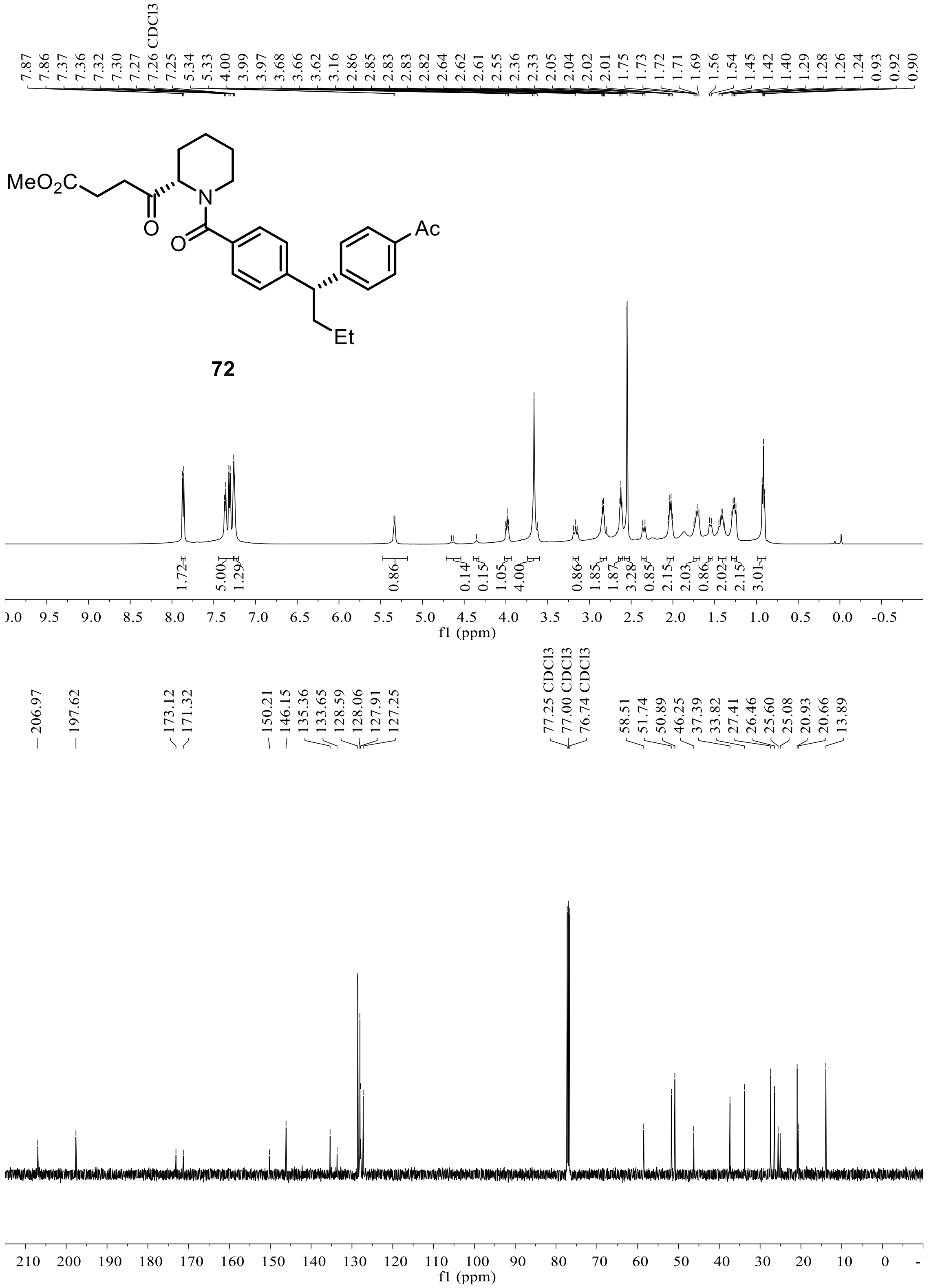

Supplementary Fig. 78 ^{1}H NMR (500 MHz, $CDCl_3$) and ^{13}C NMR (126 MHz, $CDCl_3$) spectrum of **72**.

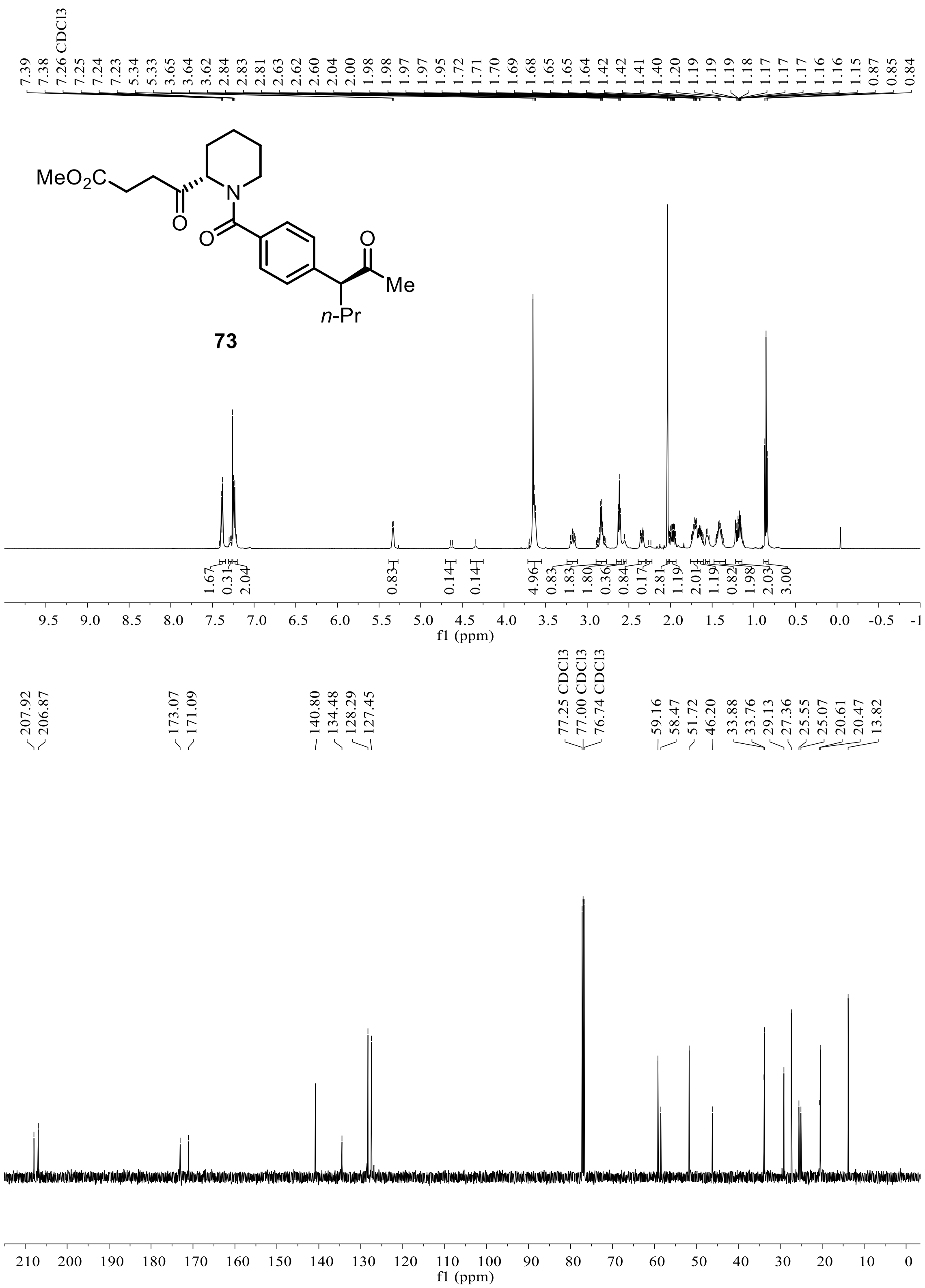

Supplementary Fig. 79 ^{1}H NMR (500 MHz, $CDCl_3$) and ^{13}C NMR (126 MHz, $CDCl_3$) spectrum of **73**.

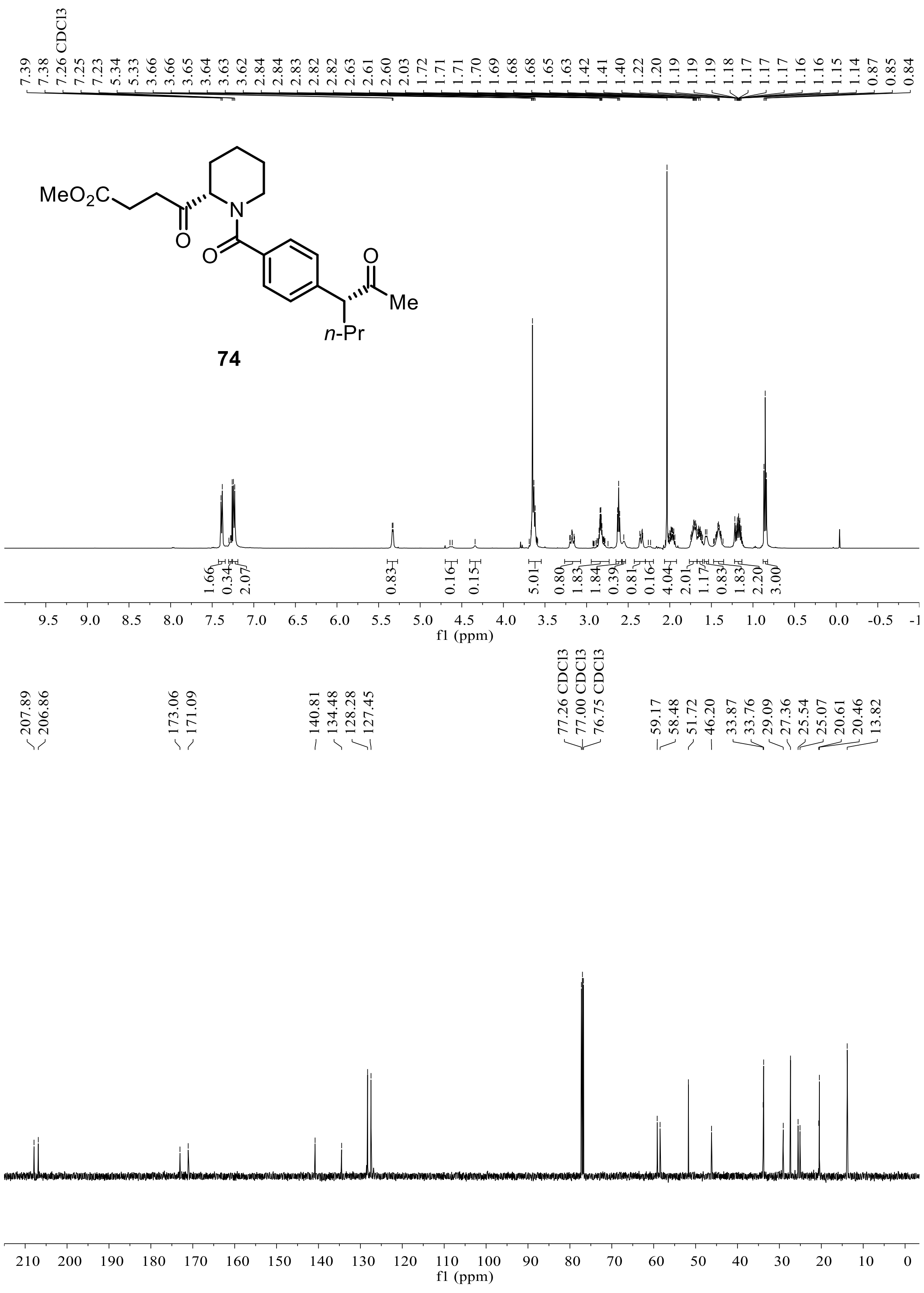

Supplementary Fig. 80 ^{1}H NMR (500 MHz, $CDCl_3$) and ^{13}C NMR (126 MHz, $CDCl_3$) spectrum of **74**.

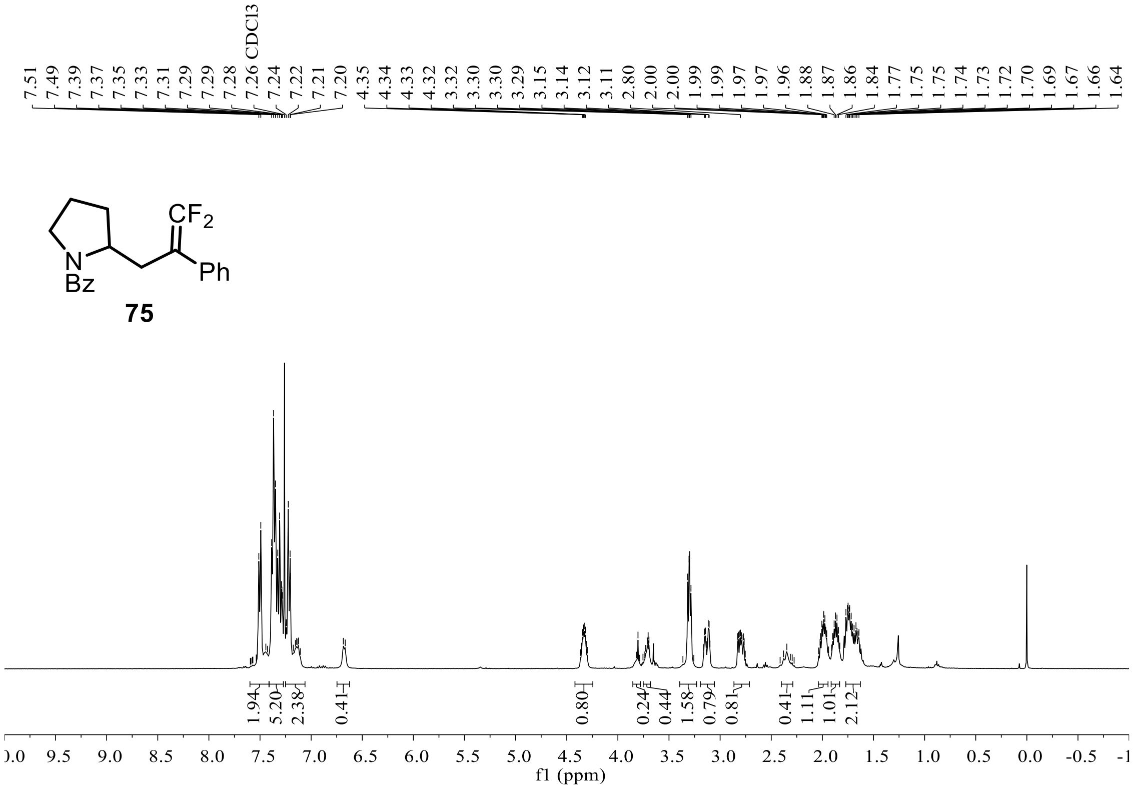
CF2
N
Bz
Ph
75
7.51 7.49 7.39 7.37 7.35 7.33 7.31 7.29 7.29 7.28 7.26 CDCl3 7.24 7.22 7.21 7.20 4.35 4.34 4.33 4.32 3.32 3.30 3.30 3.29 3.15 3.14 3.12 3.11 2.80 2.00 2.00 1.99 1.99 1.97 1.97 1.96 1.88 1.87 1.86 1.84 1.77 1.75 1.75 1.74 1.73 1.72 1.70 1.69 1.67 1.66 1.64
1.94 5.20 2.38 0.41 0.80 0.24 0.44 1.58 0.79 0.81 0.41 1.11 1.01 2.12
f1 (ppm)

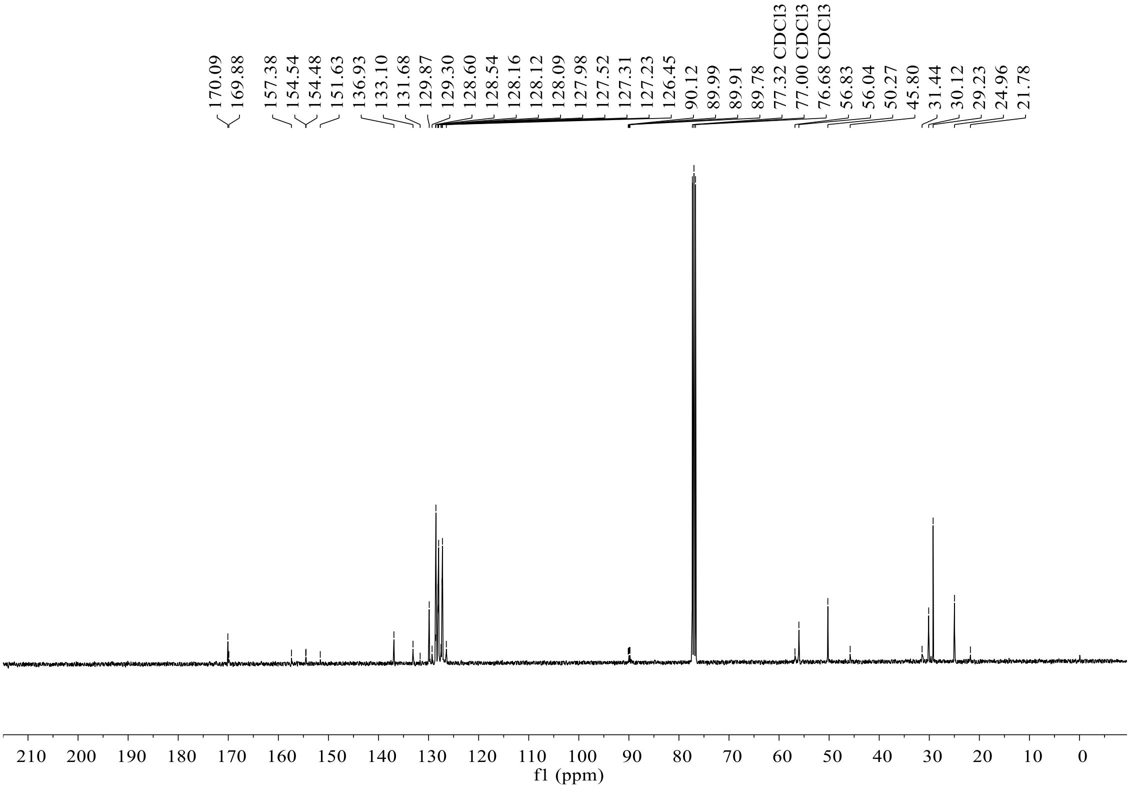
170.09 169.88 157.38 154.54 154.48 151.63 136.93 133.10 131.68 129.87 129.30 128.60 128.54 128.16 128.12 128.09 127.98 127.52 127.31 127.23 126.45 90.12 89.99 89.91 89.78 77.32 CDCl3 77.00 CDCl3 76.68 CDCl3 56.83 56.04 50.27 45.80 31.44 30.12 29.23 24.96 21.78
f1 (ppm)

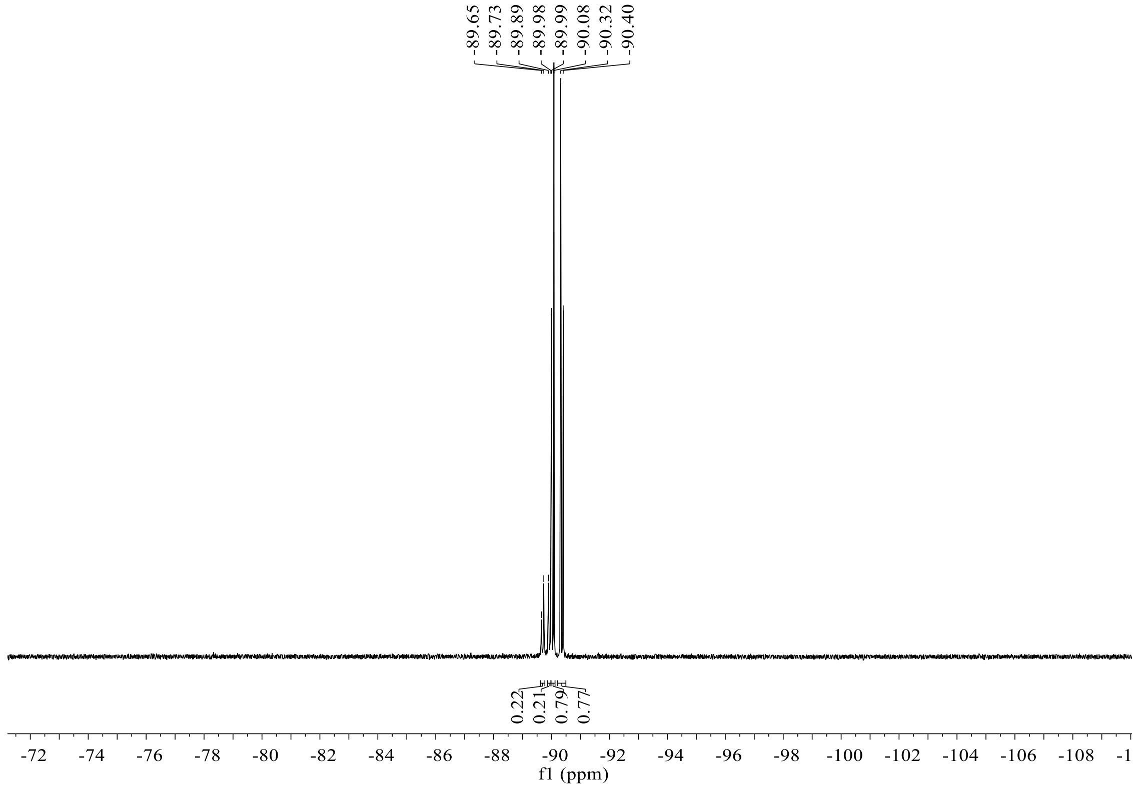

Supplementary Fig. 81 ^{1}H NMR (400 MHz, $CDCl_3$), ^{13}C NMR (101 MHz, $CDCl_3$) and ^{19}F NMR (471 MHz, $CDCl_3$) spectrum of **75**.

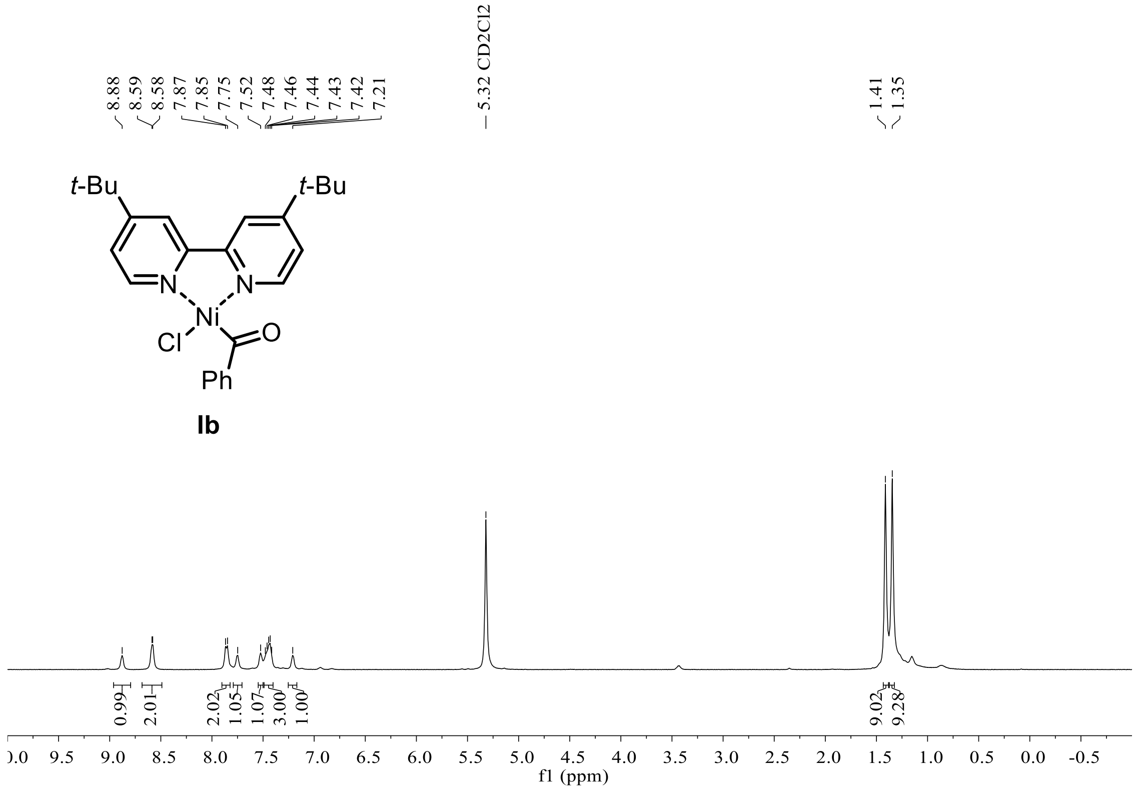

Supplementary Fig. 82 ^{1}H NMR (500 MHz, CD_2Cl_2) spectrum of **Ib**.

1.9 Stereoselectivity Analysis

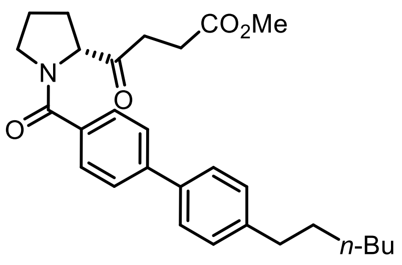

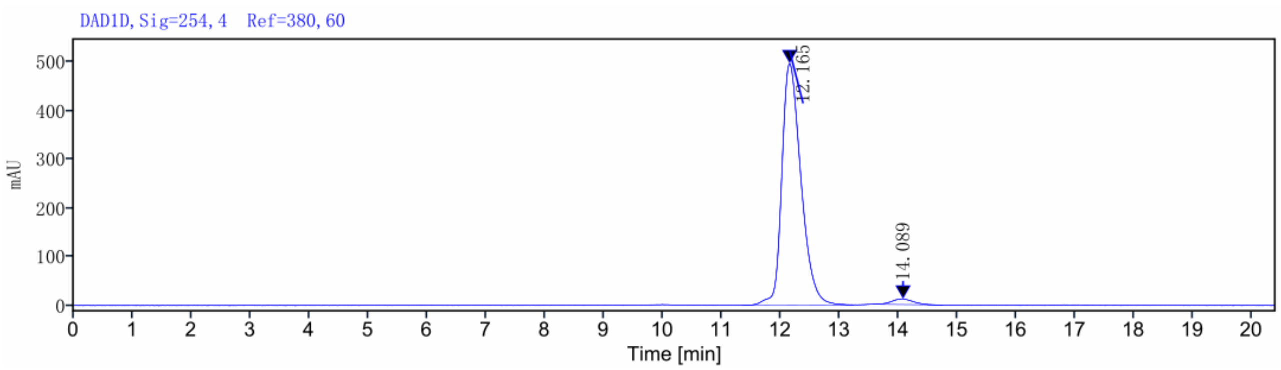

Signal: DAD1D, Sig=254, 4 Ref=380, 60

RetTime [min]	Type	Width [min]	Area [mAU*s]	Height [mAU]	Area %
12.165	MM m	1.90471	11237.85680	496.80463	97.5217
14.089	MM m	1.19044	285.58194	11.88619	2.4783
		Totals	11523.43874		

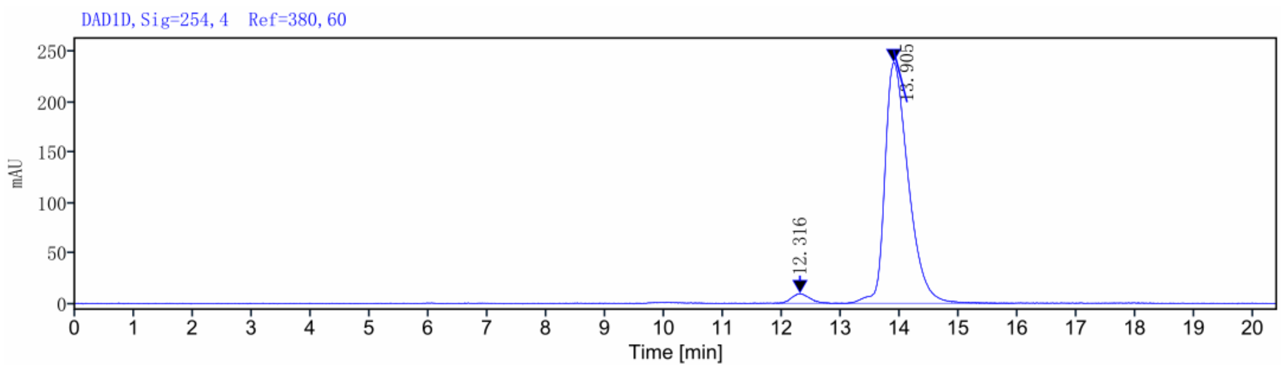

Signal: DAD1D, Sig=254, 4 Ref=380, 60

RetTime [min]	Type	Width [min]	Area [mAU*s]	Height [mAU]	Area %
12.316	MM m	0.87302	199.20733	9.83313	2.9154
13.905	MM m	3.17460	6633.72606	239.74617	97.0846
		Totals	6832.93339		

Supplementary Fig. 83 HPLC data of **3a**.

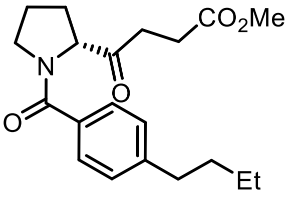

(*S*, *R*)-**L1**: 96% ee

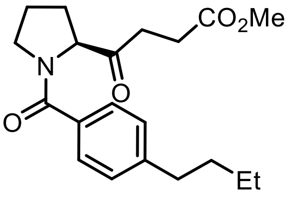

(*R*, *S*)-**L1**: 95% ee

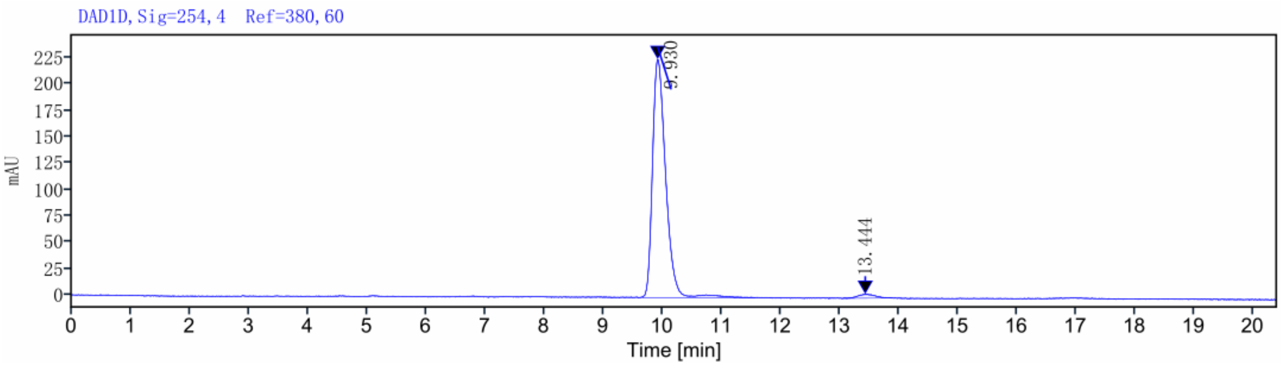

Signal: DAD1D, Sig=254, 4 Ref=380, 60

RetTime [min]	Type	Width [min]	Area [mAU*s]	Height [mAU]	Area %
9.930	VM m	1.90198	3511.80761	226.63921	97.9172
13.444	MM m	0.82254	74.70115	3.98812	2.0828
		Totals	3586.50875		

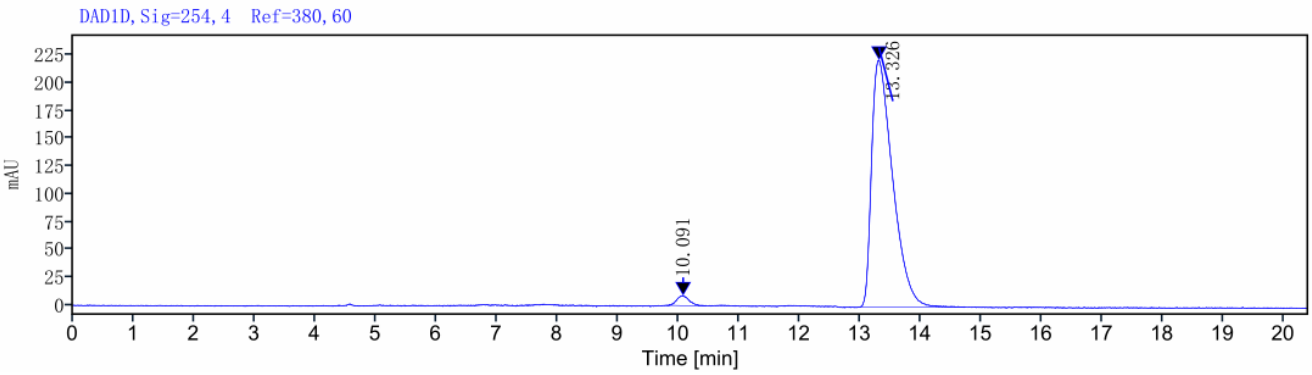

Signal: DAD1D, Sig=254, 4 Ref=380, 60

RetTime [min]	Type	Width [min]	Area [mAU*s]	Height [mAU]	Area %
10.091	MM m	0.64995	129.41212	8.96702	2.3581
13.326	MM m	1.72574	5358.55727	222.09704	97.6419
		Totals	5487.96939		

Supplementary Fig. 84 HPLC data of **4**.

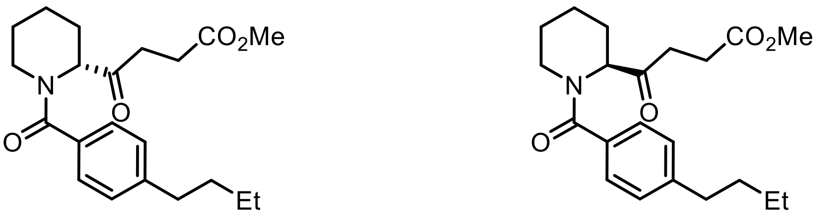

(*S*, *R*)-**L2**: 94% ee (*R*, *S*)-**L2**: 93% ee

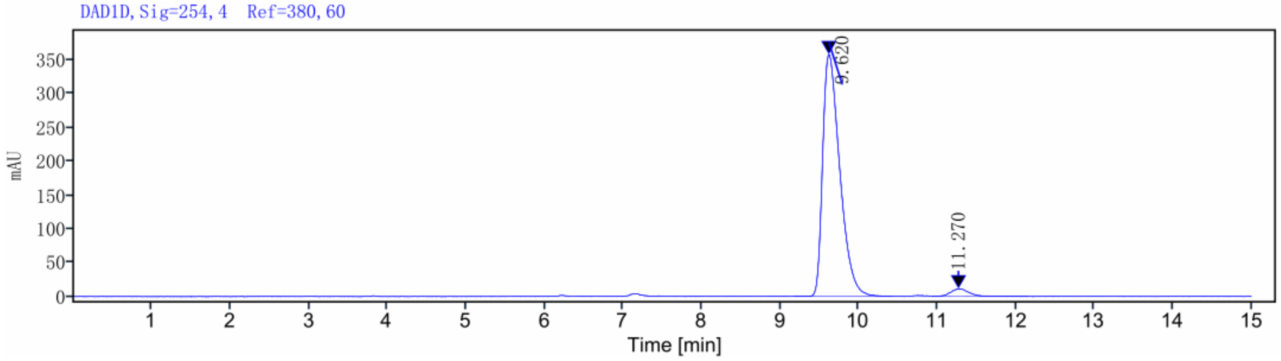

Signal: DAD1D, Sig=254, 4 Ref=380, 60

RetTime [min]	Type	Width [min]	Area [mAU*s]	Height [mAU]	Area %
9.620	MM m	1.25666	5424.61431	358.34073	97.0091
11.270	MM m	1.21097	167.24429	11.28308	2.9909
		Totals	5591.85860		

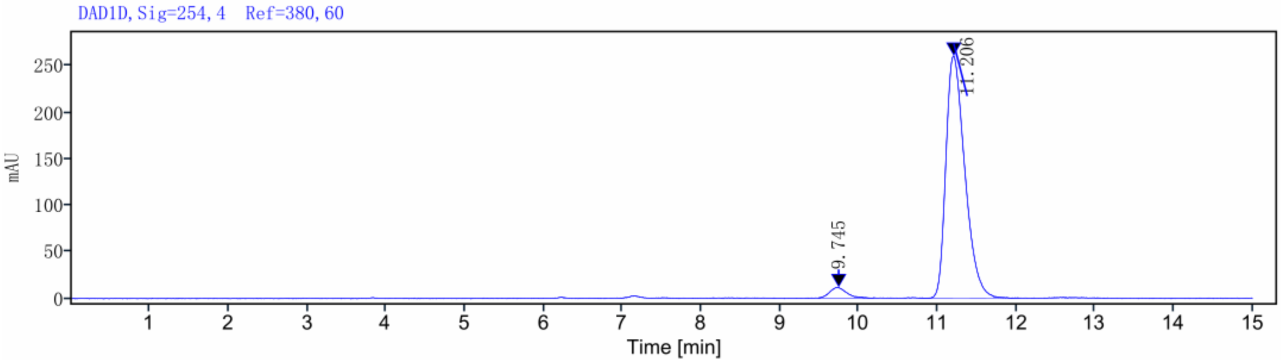

Signal: DAD1D, Sig=254, 4 Ref=380, 60

RetTime [min]	Type	Width [min]	Area [mAU*s]	Height [mAU]	Area %
9.745	MM m	0.71969	166.96413	11.52825	3.6859
11.206	MM m	1.26802	4362.79822	260.46774	96.3141
		Totals	4529.76235		

Supplementary Fig. 85 HPLC data of **5**.

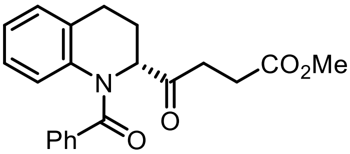

(*S*, *R*)-**L1**: 92% ee

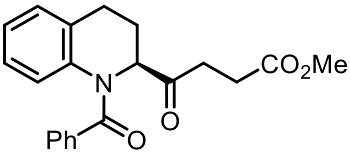

(*R*, *S*)-**L1**: 90% ee

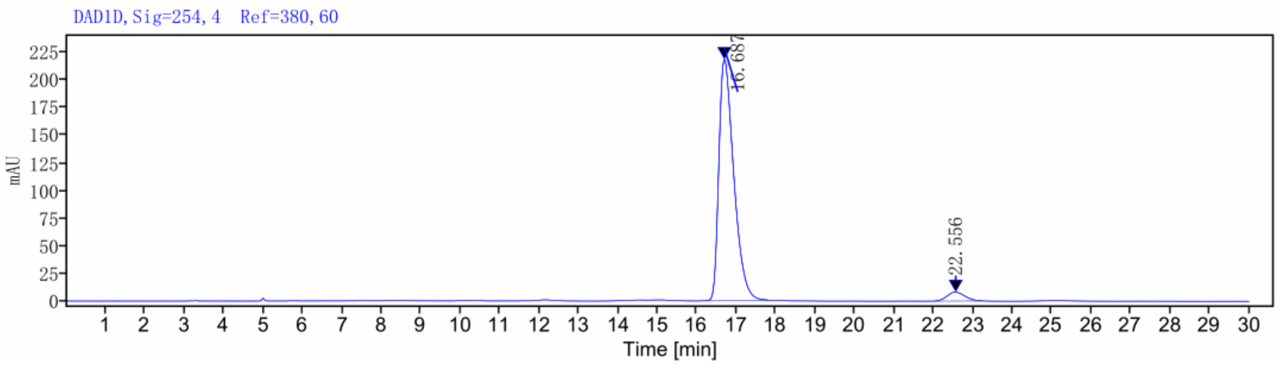

Signal: DAD1D, Sig=254, 4 Ref=380, 60

RetTime [min]	Type	Width [min]	Area [mAU*s]	Height [mAU]	Area %
16.687	VV	1.58608	5756.94178	219.22308	95.7772
22.556	VM m	1.19427	253.82010	8.11515	4.2228
		Totals	6010.76188		

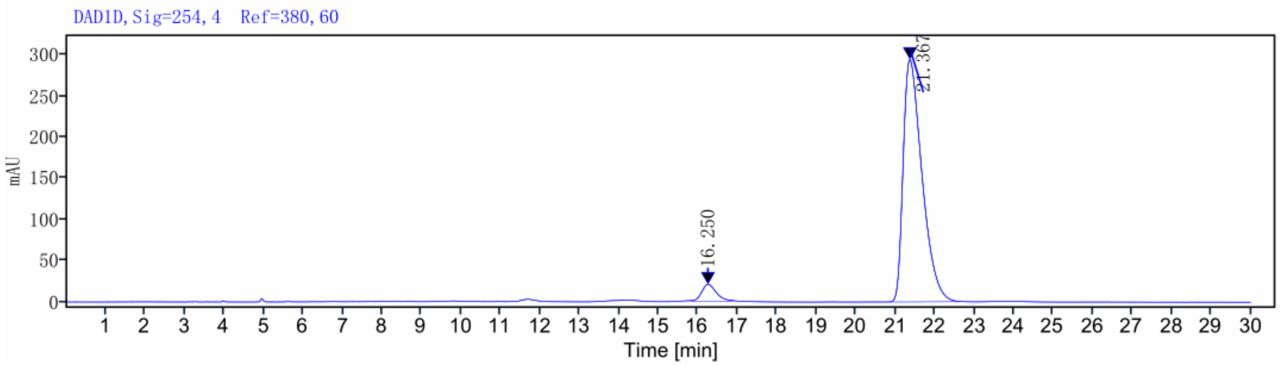

Signal: DAD1D, Sig=254, 4 Ref=380, 60

RetTime [min]	Type	Width [min]	Area [mAU*s]	Height [mAU]	Area %
16.250	VV	1.06499	522.20974	20.34943	5.0856
21.367	VV	1.78230	9746.09313	294.01410	94.9144
		Totals	10268.30288		

Supplementary Fig. 86 HPLC data of **6**.

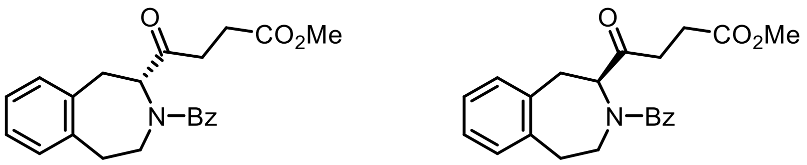

(*S*, *R*)-**L1**: 81% ee   (*R*, *S*)-**L1**: 78% ee

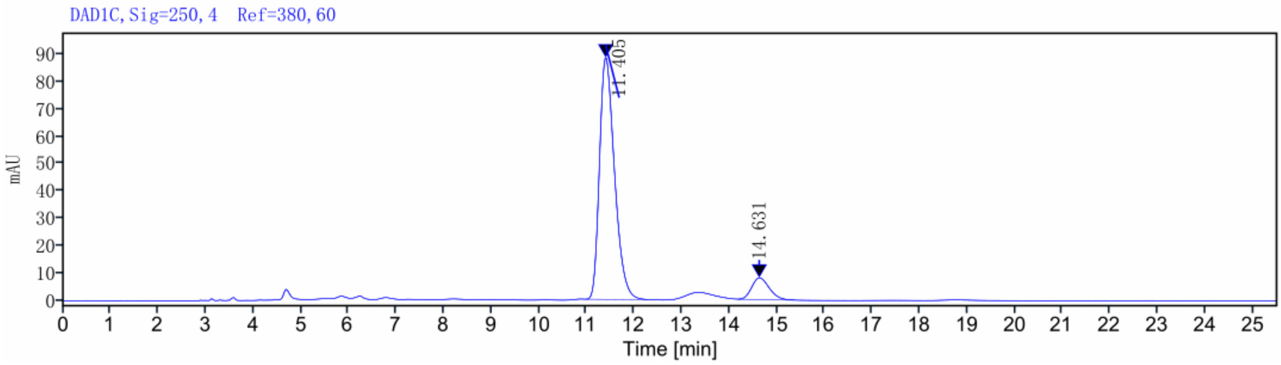

Signal: DAD1C, Sig=250, 4 Ref=380, 60

RetTime [min]	Type	Width [min]	Area [mAU*s]	Height [mAU]	Area %
11.405	VV	1.27050	1939.73961	88.39925	90.4782
14.631	BV	1.17109	204.13621	7.97735	9.5218
		Totals	2143.87582		

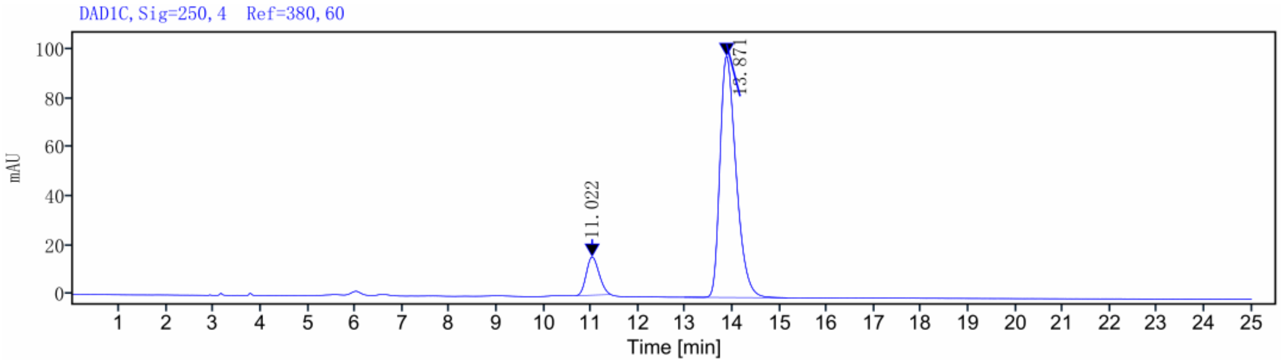

Signal: DAD1C, Sig=250, 4 Ref=380, 60

RetTime [min]	Type	Width [min]	Area [mAU*s]	Height [mAU]	Area %
11.022	MM m	0.73879	295.12774	15.65912	11.1431
13.871	MM m	2.17645	2353.38942	98.96361	88.8569
		Totals	2648.51716		

Supplementary Fig. 87 HPLC data of **7**.

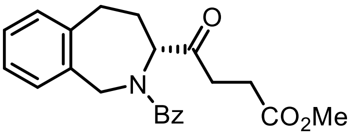

(*S*, *R*)-**L1**: 84% ee

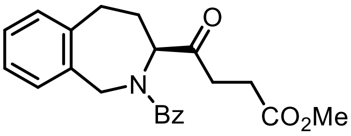

(*R*, *S*)-**L1**: 83% ee

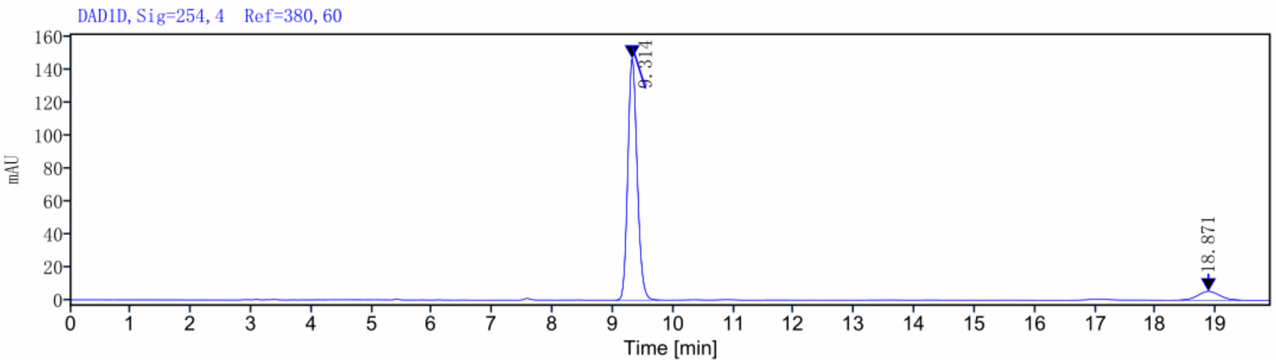

Signal: DAD1D, Sig=254, 4 Ref=380, 60

RetTime [min]	Type	Width [min]	Area [mAU*s]	Height [mAU]	Area %
9.314	BV	0.72387	1582.80447	146.60395	92.1700
18.871	VV	0.99039	134.46132	5.33534	7.8300
		Totals	1717.26579		

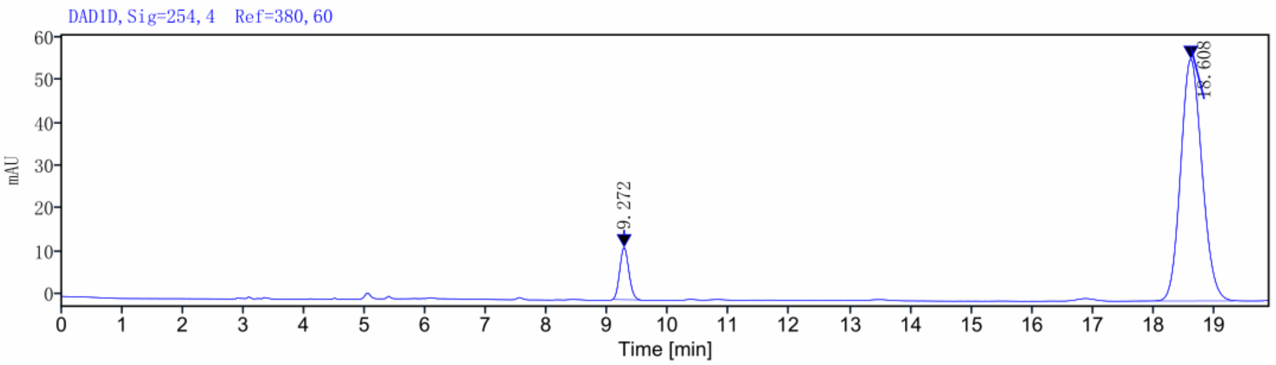

Signal: DAD1D, Sig=254, 4 Ref=380, 60

RetTime [min]	Type	Width [min]	Area [mAU*s]	Height [mAU]	Area %
9.272	MM m	0.45526	126.98902	12.12637	8.4354
18.608	VV	1.29350	1378.43941	56.21567	91.5646
		Totals	1505.42843		

Supplementary Fig. 88 HPLC data of **8**.

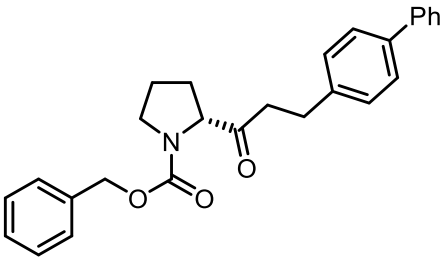

(*S*, *R*)-**L1**: 88% ee

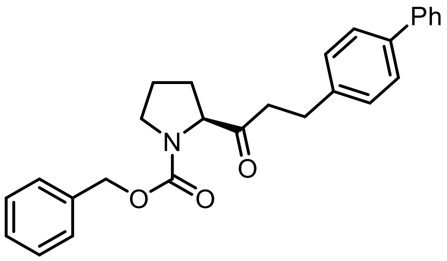

(*R*, *S*)-**L1**: 89% ee

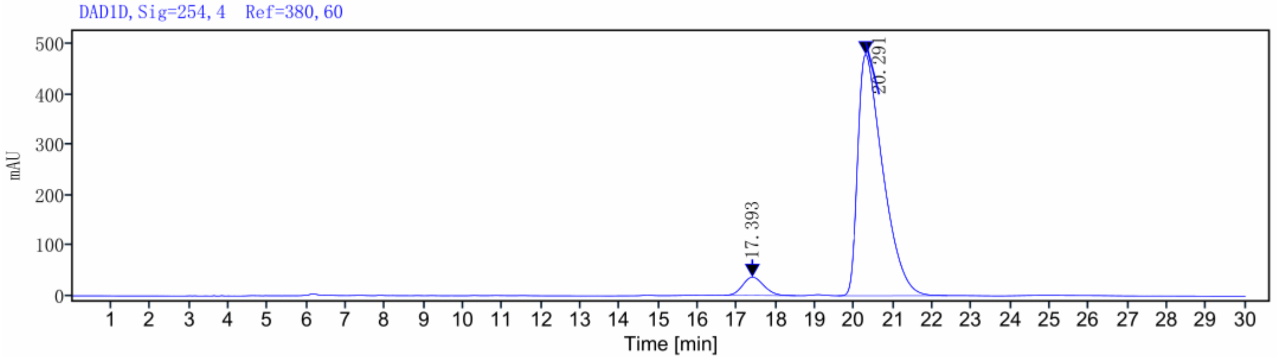

Signal: DAD1D, Sig=254, 4 Ref=380, 60

RetTime [min]	Type	Width [min]	Area [mAU*s]	Height [mAU]	Area %
17.393	BV	1.83765	1362.20774	36.33406	5.9885
20.291	BV	2.79345	21384.99775	479.54380	94.0115
		Totals	22747.20549		

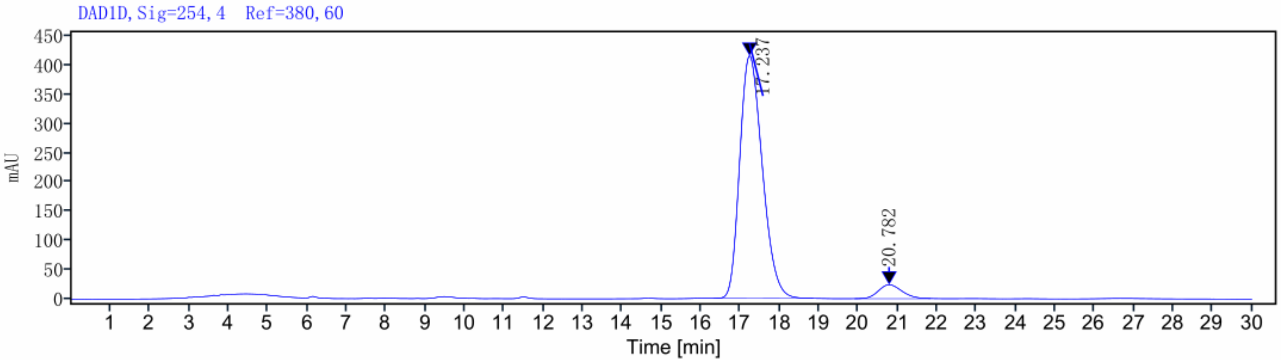

Signal: DAD1D, Sig=254, 4 Ref=380, 60

RetTime [min]	Type	Width [min]	Area [mAU*s]	Height [mAU]	Area %
17.237	VV	2.53045	16577.03073	415.18418	94.2772
20.782	VV	1.92823	1006.26169	23.95452	5.7228
		Totals	17583.29243		

Supplementary Fig. 89 HPLC data of **9**.

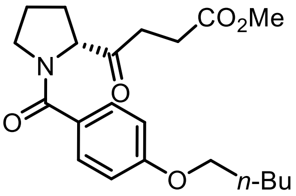

(*S, R*)-**L1**: 94% ee

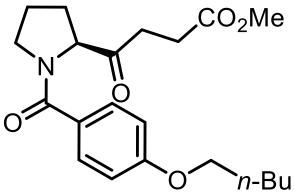

(*R, S*)-**L1**: 94% ee

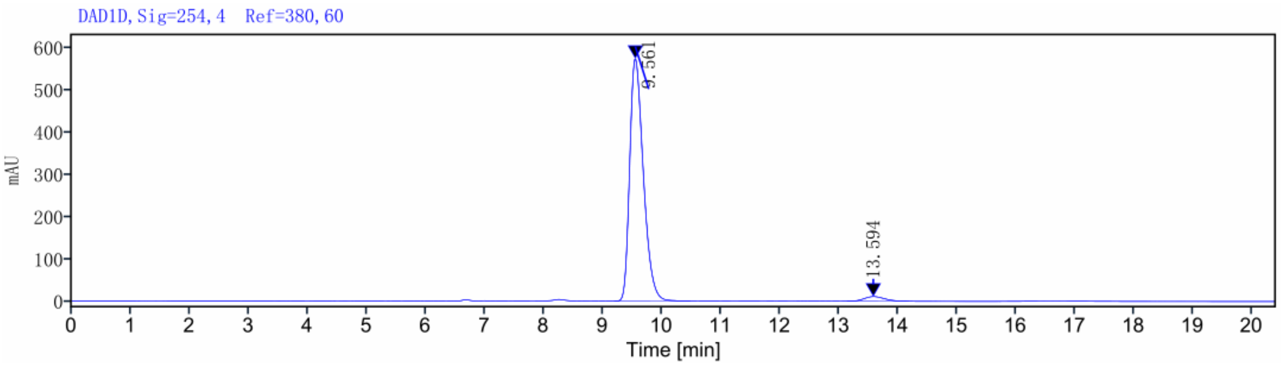

Signal: DAD1D, Sig=254, 4 Ref=380, 60

RetTime [min]	Type	Width [min]	Area [mAU*s]	Height [mAU]	Area %
9.561	BV	1.29750	9232.45809	574.14134	97.1588
13.594	BV	1.04084	269.98113	11.14164	2.8412
		Totals	9502.43922		

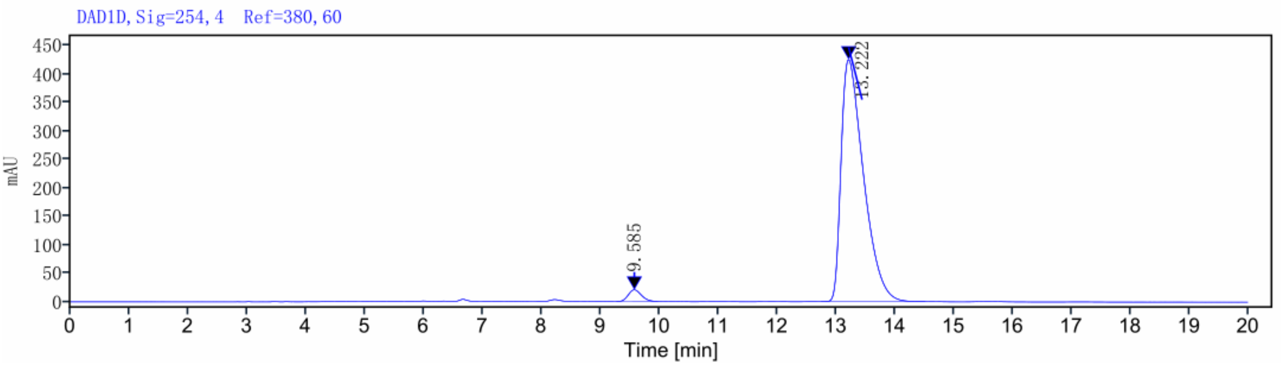

Signal: DAD1D, Sig=254, 4 Ref=380, 60

RetTime [min]	Type	Width [min]	Area [mAU*s]	Height [mAU]	Area %
9.585	BV	0.70401	320.11285	20.65124	2.7736
13.222	BB	1.76250	11221.41116	425.22644	97.2264
		Totals	11541.52401		

Supplementary Fig. 90 HPLC data of **10**.

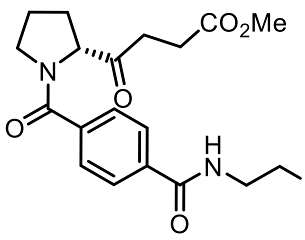

(*S*, *R*)-**L1**: 88% ee

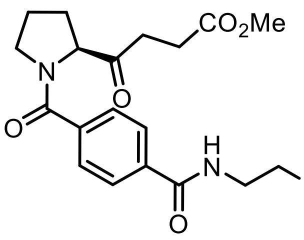

(*R*, *S*)-**L1**: 89% ee

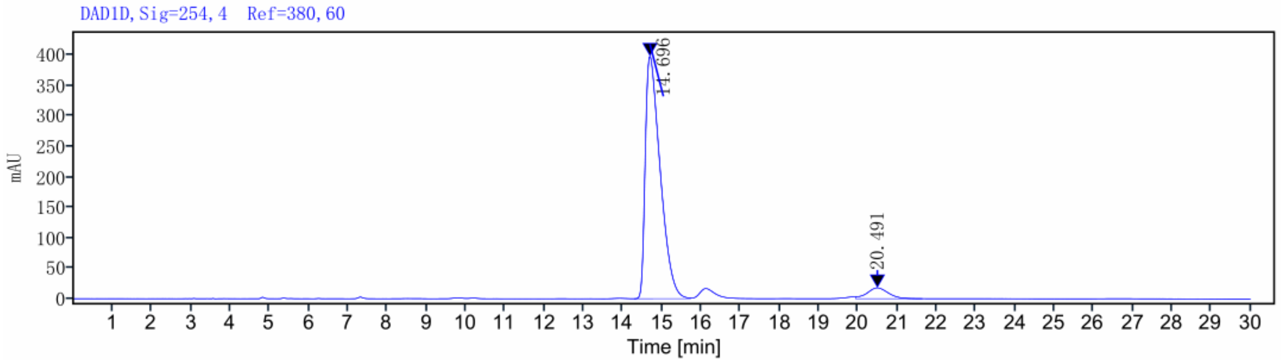

Signal: DAD1D, Sig=254, 4 Ref=380, 60

RetTime [min]	Type	Width [min]	Area [mAU*s]	Height [mAU]	Area %
14.696	BV	1.44159	10521.59115	398.65081	94.0450
20.491	MM m	1.70123	666.23225	17.78140	5.9550
		Totals	11187.82340		

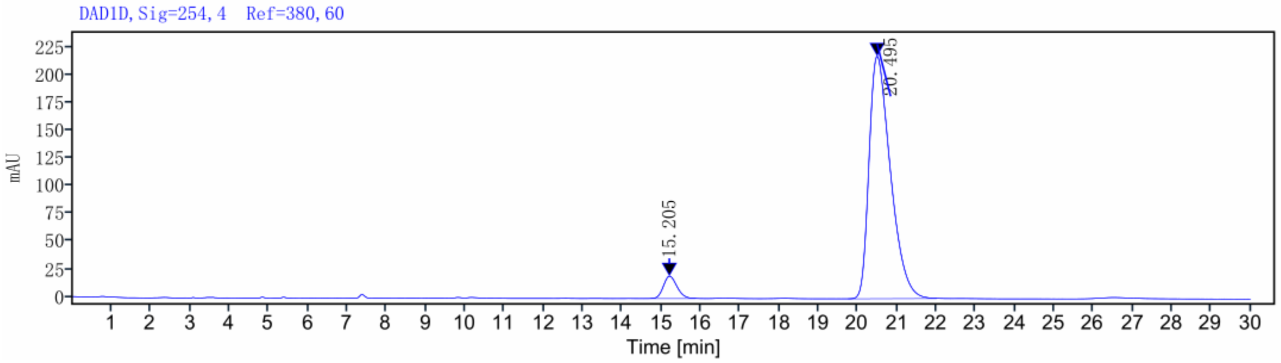

Signal: DAD1D, Sig=254, 4 Ref=380, 60

RetTime [min]	Type	Width [min]	Area [mAU*s]	Height [mAU]	Area %
15.205	VV	1.14637	488.97772	20.25677	5.5703
20.495	BV	2.25065	8289.36065	218.93323	94.4297
		Totals	8778.33837		

Supplementary Fig. 91 HPLC data of **11**.

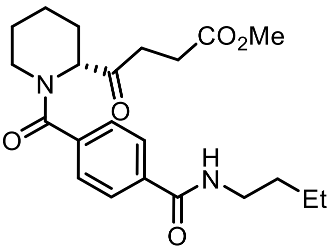

(*S*, *R*)-**L2**: 93% ee

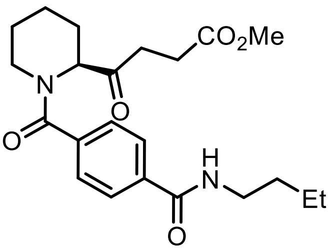

(*R*, *S*)-**L2**: 92% ee

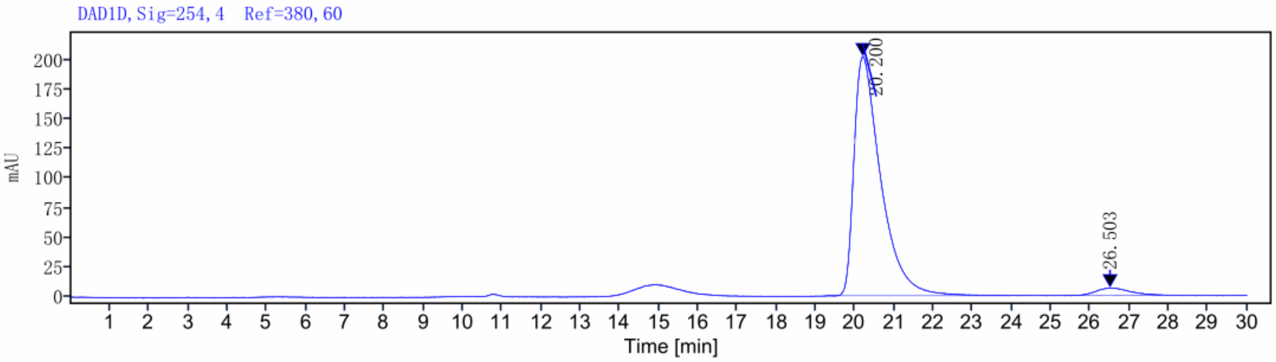

Signal: DAD1D, Sig=254, 4 Ref=380, 60

RetTime [min]	Type	Width [min]	Area [mAU*s]	Height [mAU]	Area %
20.200	MM m	4.70886	9573.98993	201.22933	96.6016
26.503	MM m	2.46025	336.80467	6.40423	3.3984
		Totals	9910.79460		

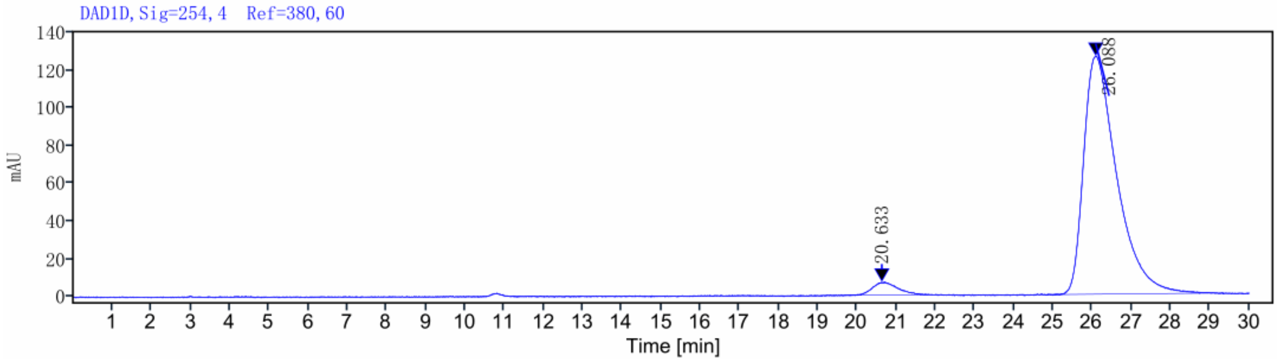

Signal: DAD1D, Sig=254, 4 Ref=380, 60

RetTime [min]	Type	Width [min]	Area [mAU*s]	Height [mAU]	Area %
20.633	MM m	2.01053	292.60311	6.93260	3.8128
26.088	MM m	4.17978	7381.53419	125.91464	96.1872
		Totals	7674.13730		

Supplementary Fig. 92 HPLC data of **12.**

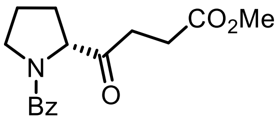

(*S*, *R*)-**L1**: 96% ee

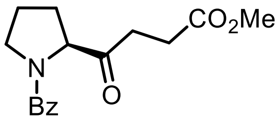

(*R*, *S*)-**L1**: 97% ee

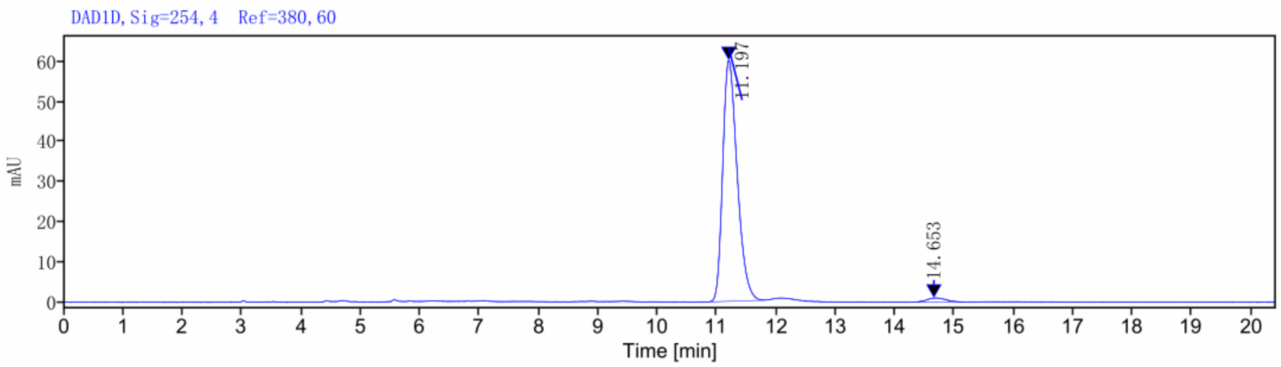

Signal: DAD1D, Sig=254, 4 Ref=380, 60

RetTime [min]	Type	Width [min]	Area [mAU*s]	Height [mAU]	Area %
11.197	VM m	0.88012	1017.68103	60.22257	97.9896
14.653	MM m	0.77776	20.87935	1.03752	2.0104
		Totals	1038.56038		

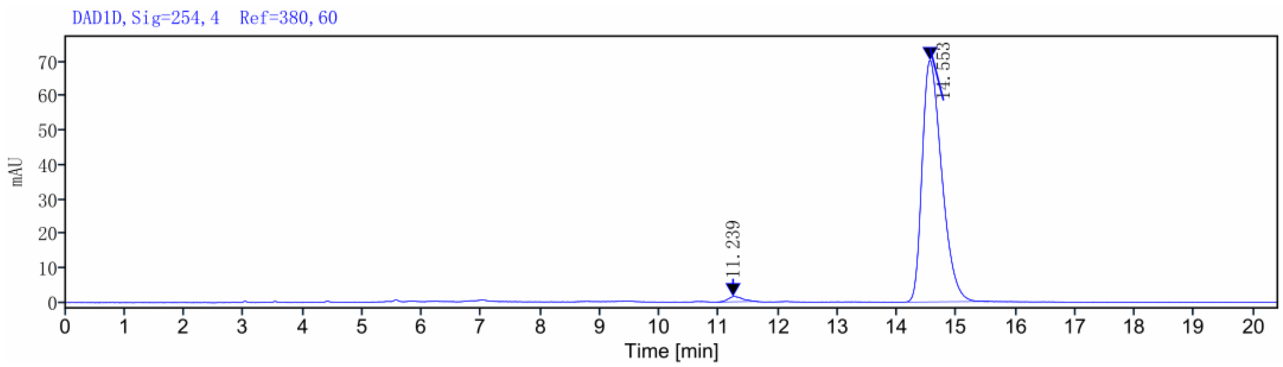

Signal: DAD1D, Sig=254, 4 Ref=380, 60

RetTime [min]	Type	Width [min]	Area [mAU*s]	Height [mAU]	Area %
11.239	MM m	0.66665	26.21681	1.55574	1.6013
14.553	VM m	1.37419	1610.98588	70.23717	98.3987
		Totals	1637.20269		

Supplementary Fig. 93 HPLC data of **13.**

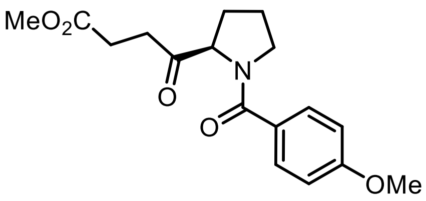

(*S*, *R*)-**L1**: 97% ee

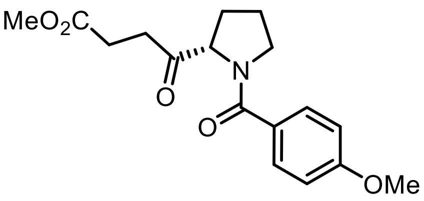

(*R*, *S*)-**L1**: 97% ee

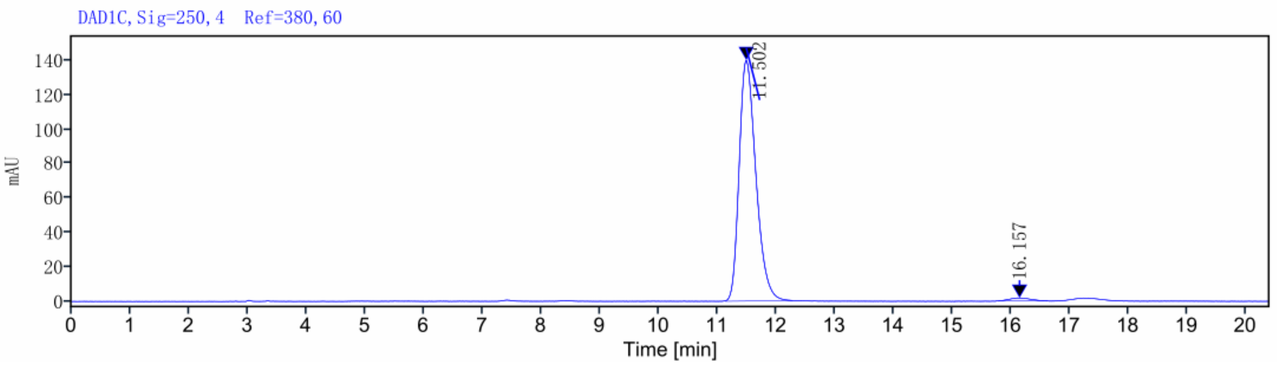

Signal: DAD1C, Sig=250, 4 Ref=380, 60

RetTime [min]	Type	Width [min]	Area [mAU*s]	Height [mAU]	Area %
11.502	VM m	1.14763	2769.16118	139.94796	98.4951
16.157	MM m	0.83532	42.31065	1.77759	1.5049
		Totals	2811.47183		

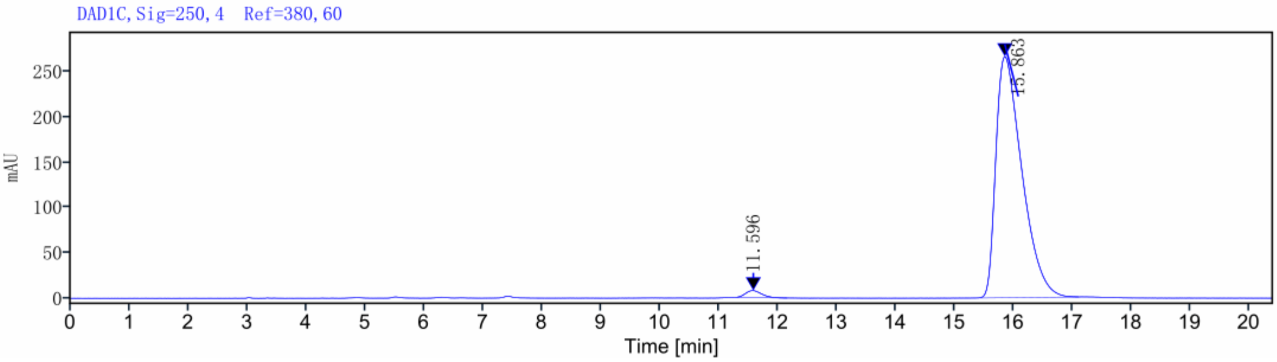

Signal: DAD1C, Sig=250, 4 Ref=380, 60

RetTime [min]	Type	Width [min]	Area [mAU*s]	Height [mAU]	Area %
11.596	MM m	0.85919	143.94305	7.75212	1.7276
15.863	VM m	1.66737	8188.23525	266.95307	98.2724
		Totals	8332.17830		

Supplementary Fig. 94 HPLC data of **14.**

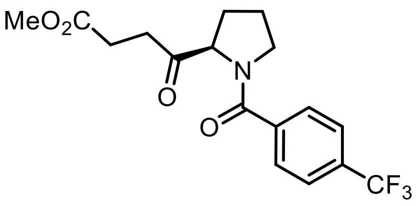

(*S*, *R*)-**L1**: 96% ee

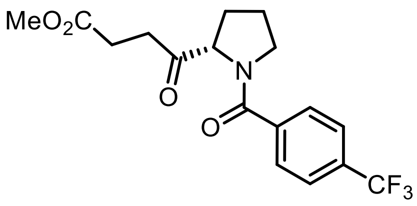

(*R*, *S*)-**L1**: 96% ee

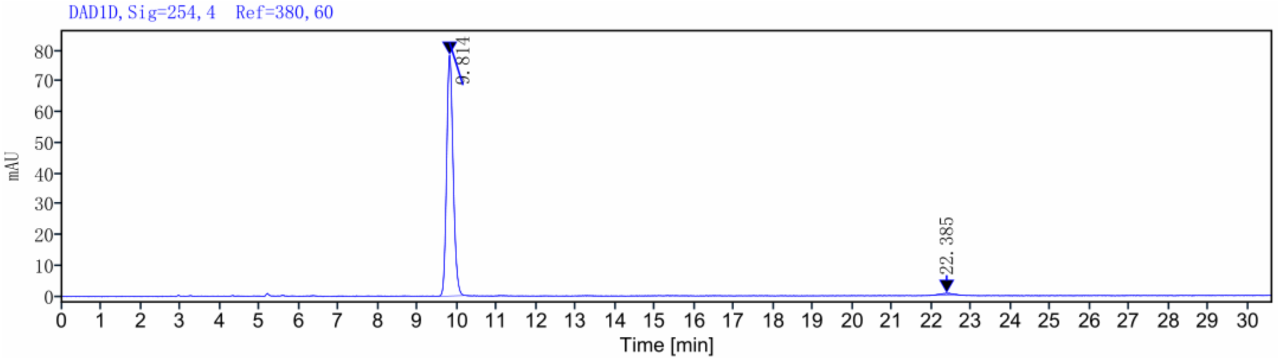

Signal: DAD1D, Sig=254, 4 Ref=380, 60

RetTime [min]	Type	Width [min]	Area [mAU*s]	Height [mAU]	Area %
9.814	VV	0.58531	897.51794	78.57721	98.1537
22.385	MM m	0.84471	16.88267	0.77112	1.8463
		Totals	914.40061		

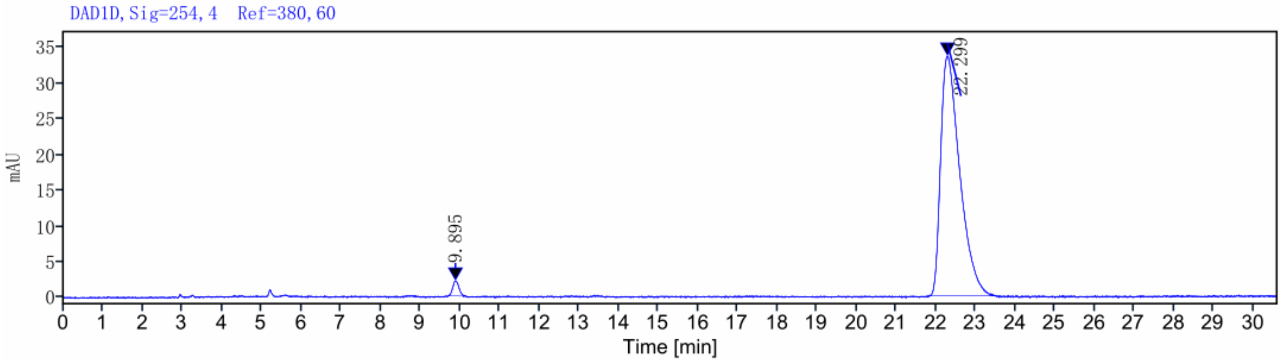

Signal: DAD1D, Sig=254, 4 Ref=380, 60

RetTime [min]	Type	Width [min]	Area [mAU*s]	Height [mAU]	Area %
9.895	MM m	0.42236	22.34273	2.11907	1.9514
22.299	MM m	1.67488	1122.63434	33.56514	98.0486
		Totals	1144.97707		

Supplementary Fig. 95 HPLC data of **15.**

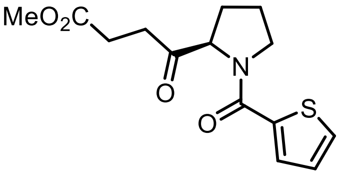

(*S*, *R*)-**L1**: 97% ee

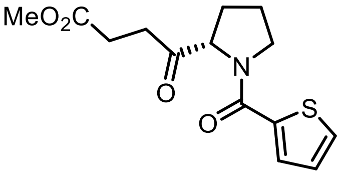

(*R*, *S*)-**L1**: 97% ee

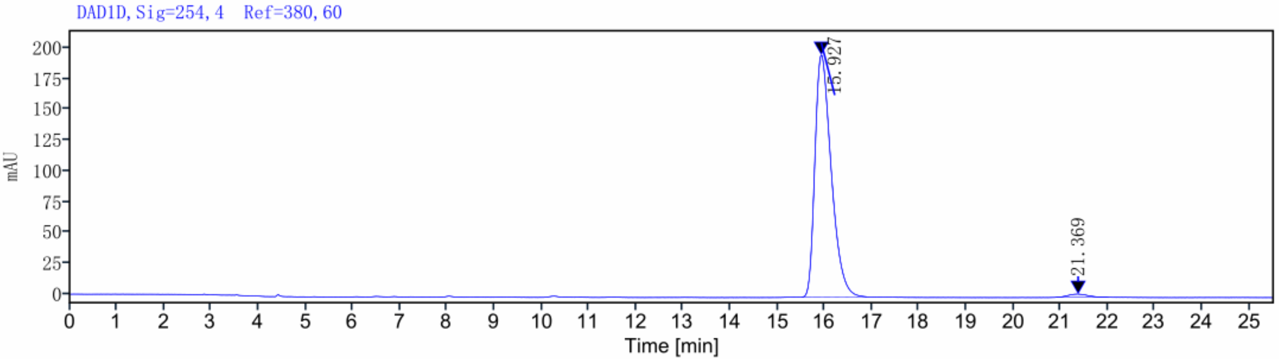

Signal: DAD1D, Sig=254, 4 Ref=380, 60

RetTime [min]	Type	Width [min]	Area [mAU*s]	Height [mAU]	Area %
15.927	VM m	1.37166	4729.27595	196.66464	98.3866
21.369	MM m	1.11019	77.55251	2.69487	1.6134
		Totals	4806.82847		

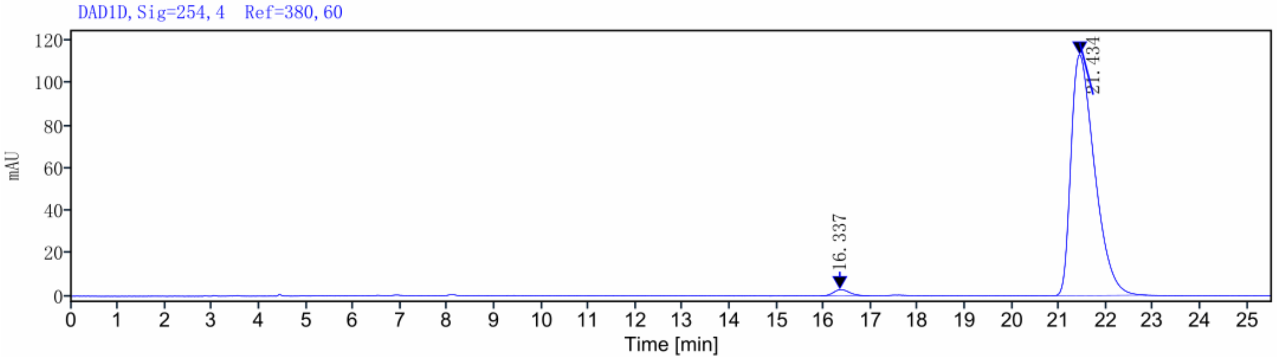

Signal: DAD1D, Sig=254, 4 Ref=380, 60

RetTime [min]	Type	Width [min]	Area [mAU*s]	Height [mAU]	Area %
16.337	MM m	1.03778	68.46514	3.01906	1.7052
21.434	VM m	1.90358	3946.51254	113.12180	98.2948
		Totals	4014.97768		

Supplementary Fig. 96 HPLC data of **16.**

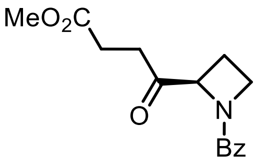

(*S*, *R*)-**L1**: 85% ee

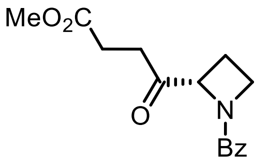

(*R*, *S*)-**L1**: 84% ee

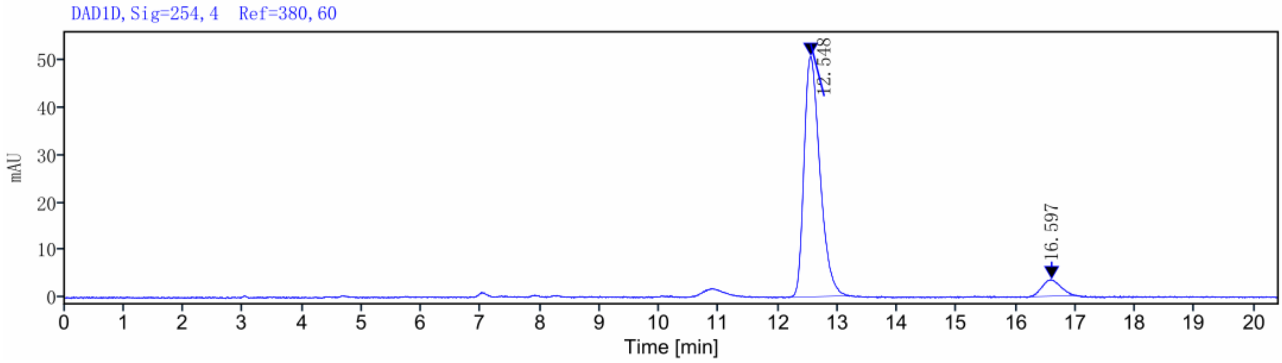

Signal: DAD1D, Sig=254, 4 Ref=380, 60

RetTime [min]	Type	Width [min]	Area [mAU*s]	Height [mAU]	Area %
12.548	VM m	0.95841	939.30118	50.63576	92.6329
16.597	MM m	0.77233	74.70300	3.52059	7.3671
		Totals	1014.00419		

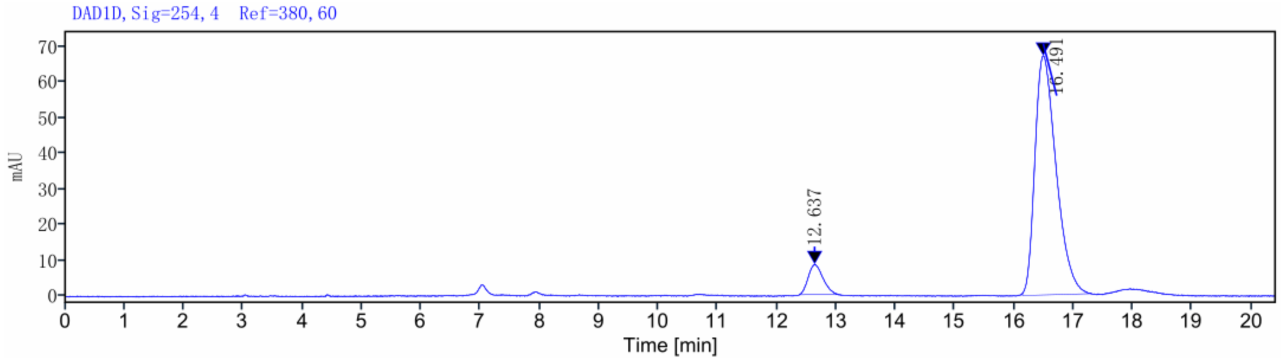

Signal: DAD1D, Sig=254, 4 Ref=380, 60

RetTime [min]	Type	Width [min]	Area [mAU*s]	Height [mAU]	Area %
12.637	MM m	0.65165	143.96919	8.47829	7.9479
16.491	VM m	1.16430	1667.43798	67.04094	92.0521
		Totals	1811.40717		

Supplementary Fig. 97 HPLC data of **17.**

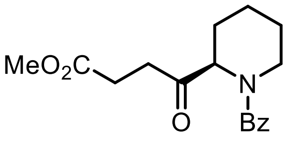

(*S*, *R*)-**L2**: 94% ee

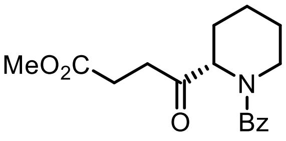

(*R*, *S*)-**L2**: 95% ee

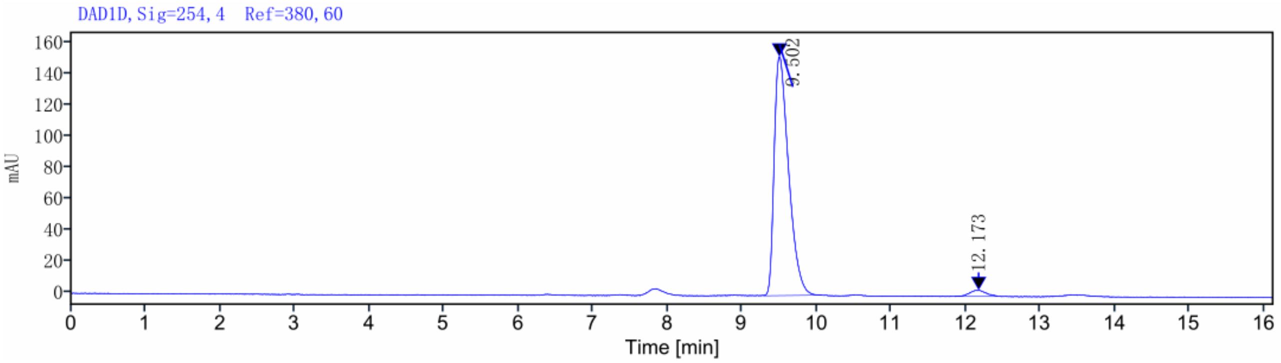

Signal: DAD1D, Sig=254, 4 Ref=380, 60

RetTime [min]	Type	Width [min]	Area [mAU*s]	Height [mAU]	Area %
9.502	VM m	0.76845	2052.56184	152.38892	97.2078
12.173	MM m	0.57923	58.95752	3.96462	2.7922
		Totals	2111.51936		

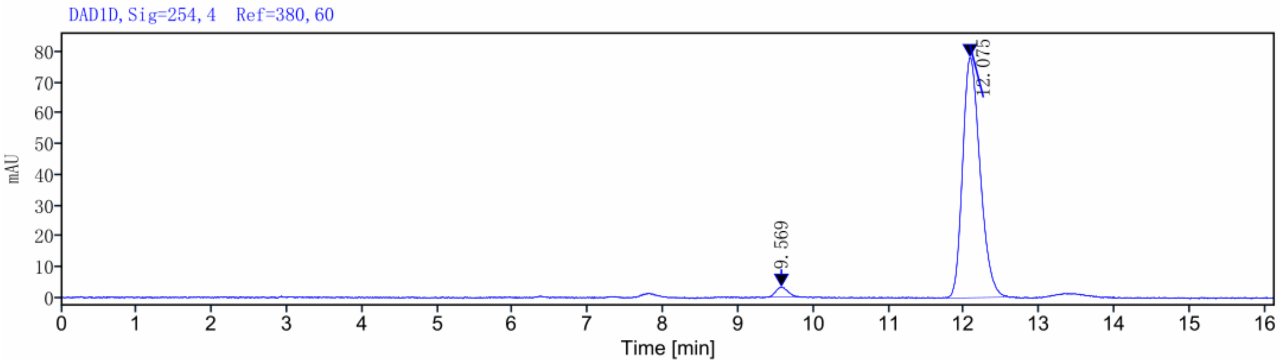

Signal: DAD1D, Sig=254, 4 Ref=380, 60

RetTime [min]	Type	Width [min]	Area [mAU*s]	Height [mAU]	Area %
9.569	MM m	0.41029	34.01206	3.18478	2.6189
12.075	BM m	0.75830	1264.71796	78.27704	97.3811
		Totals	1298.73002		

Supplementary Fig. 98 HPLC data of **18.**

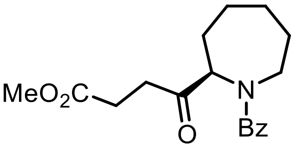

(*S*, *R*)-**L1**: 88% ee

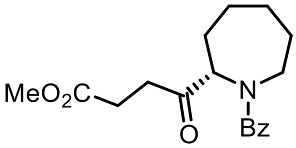

(*R*, *S*)-**L1**: 89% ee

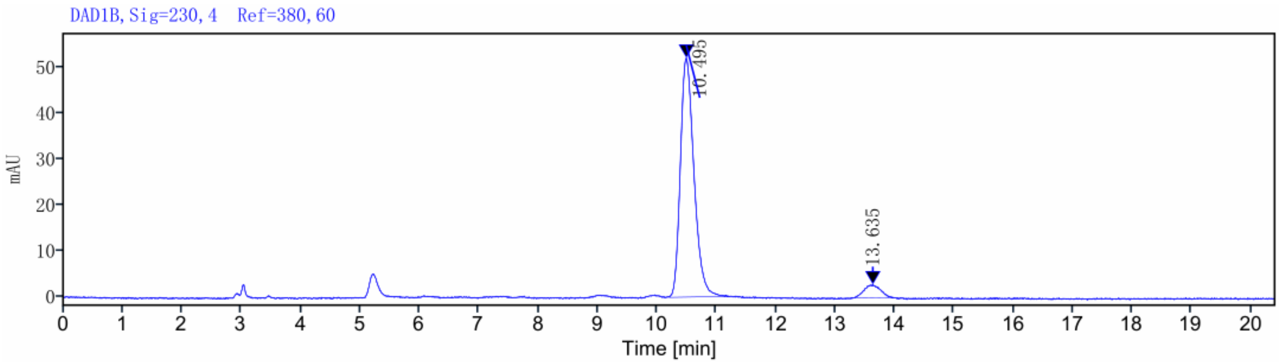

Signal: DAD1B, Sig=230, 4 Ref=380, 60

RetTime [min]	Type	Width [min]	Area [mAU*s]	Height [mAU]	Area %
10.495	VM m	1.01422	829.27266	52.23380	93.8102
13.635	MM m	0.65537	54.71766	2.92034	6.1898
		Totals	883.99032		

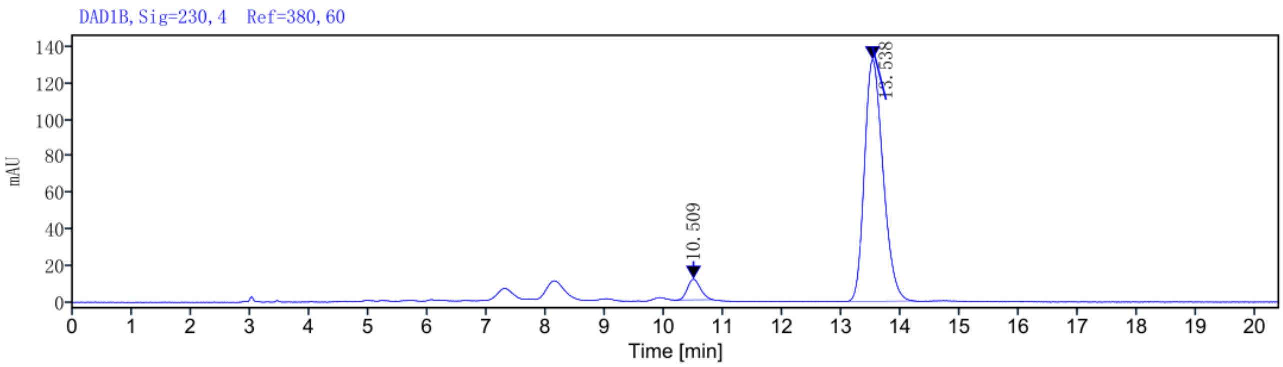

Signal: DAD1B, Sig=230, 4 Ref=380, 60

RetTime [min]	Type	Width [min]	Area [mAU*s]	Height [mAU]	Area %
10.509	MM m	0.66574	171.64600	11.44068	5.5905
13.538	VM m	1.10488	2898.67702	132.61787	94.4095
		Totals	3070.32301		

Supplementary Fig. 99 HPLC data of **19.**

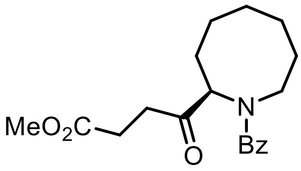

(*S*, *R*)-**L1**: 80% ee

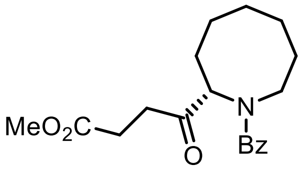

(*R*, *S*)-**L1**: 80% ee

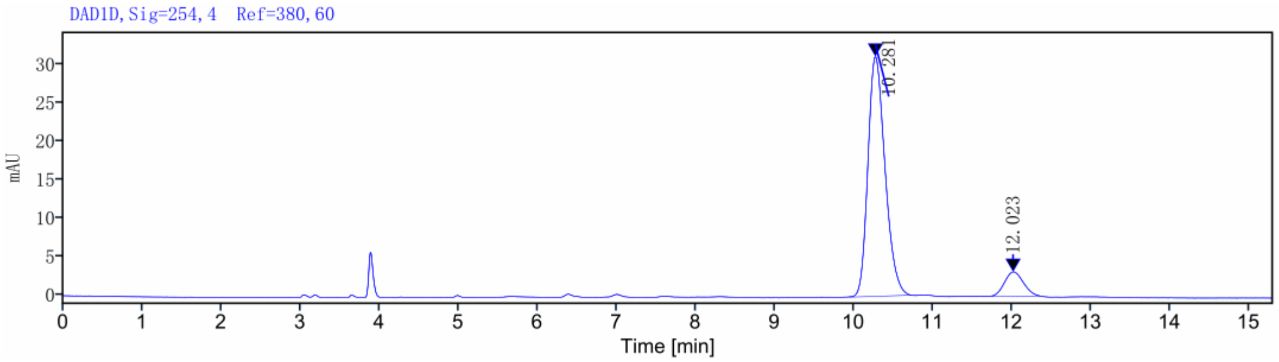

Signal: DAD1D, Sig=254, 4 Ref=380, 60

RetTime [min]	Type	Width [min]	Area [mAU*s]	Height [mAU]	Area %
10.281	VM m	0.77171	470.95717	31.30208	89.9130
12.023	VM m	0.58971	52.83495	3.18298	10.0870
		Totals	523.79212		

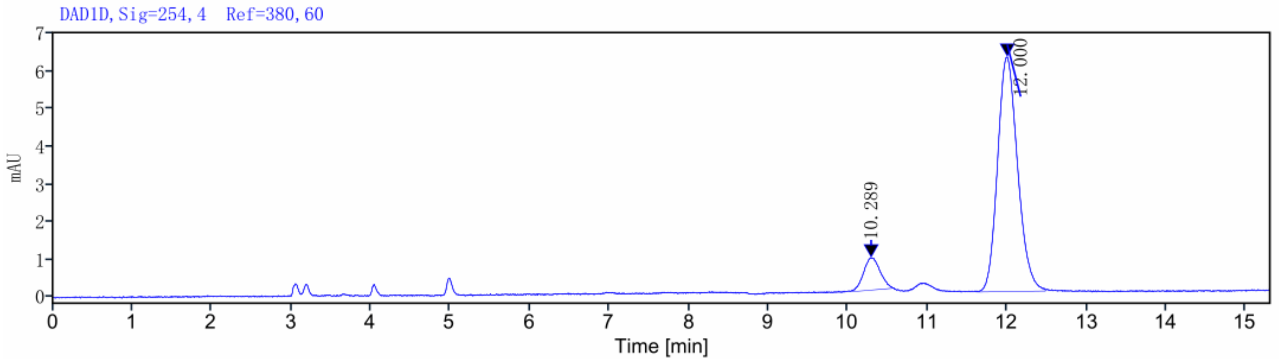

Signal: DAD1D, Sig=254, 4 Ref=380, 60

RetTime [min]	Type	Width [min]	Area [mAU*s]	Height [mAU]	Area %
10.289	VM m	0.50557	12.37051	0.86164	10.1994
12.000	VV	0.85780	108.91631	6.22194	89.8006
		Totals	121.28682		

Supplementary Fig. 100 HPLC data of **20.**

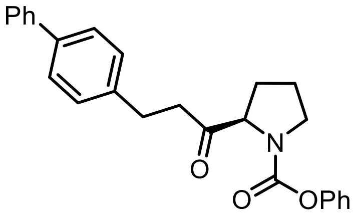

(*S*, *R*)-**L1**: 89% ee

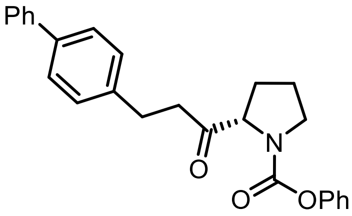

(*R*, *S*)-**L1**: 90% ee

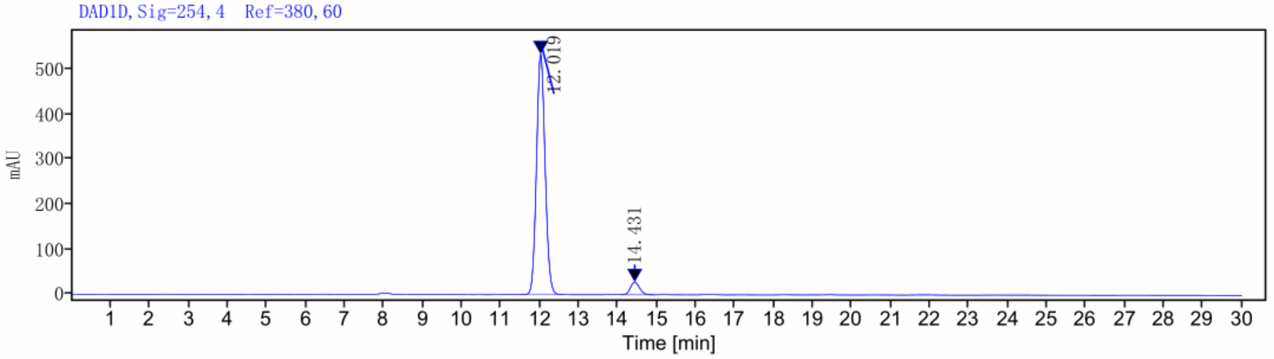

Signal: DAD1D, Sig=254, 4 Ref=380, 60

RetTime [min]	Type	Width [min]	Area [mAU*s]	Height [mAU]	Area %
12.019	VV	1.08752	8193.76331	534.07784	94.6944
14.431	VV	0.83854	459.08969	28.13462	5.3056
		Totals	8652.85300		

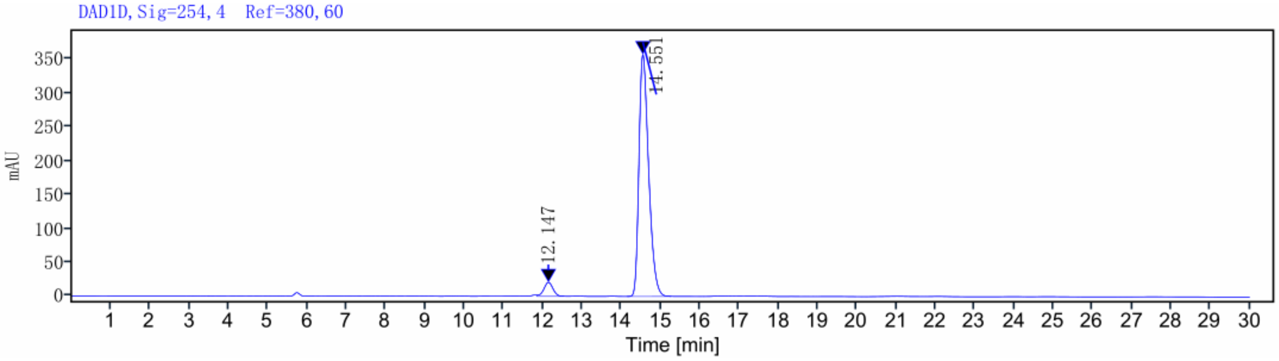

Signal: DAD1D, Sig=254, 4 Ref=380, 60

RetTime [min]	Type	Width [min]	Area [mAU*s]	Height [mAU]	Area %
12.147	VV	0.69351	334.93597	20.78001	5.1880
14.551	VV	1.11700	6121.05251	357.51652	94.8120
		Totals	6455.98848		

Supplementary Fig. 101 HPLC data of **21.**

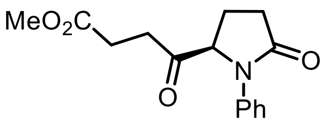

(*S*, *R*)-**L2**: 88% ee

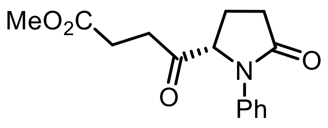

(*R*, *S*)-**L2**: 88% ee

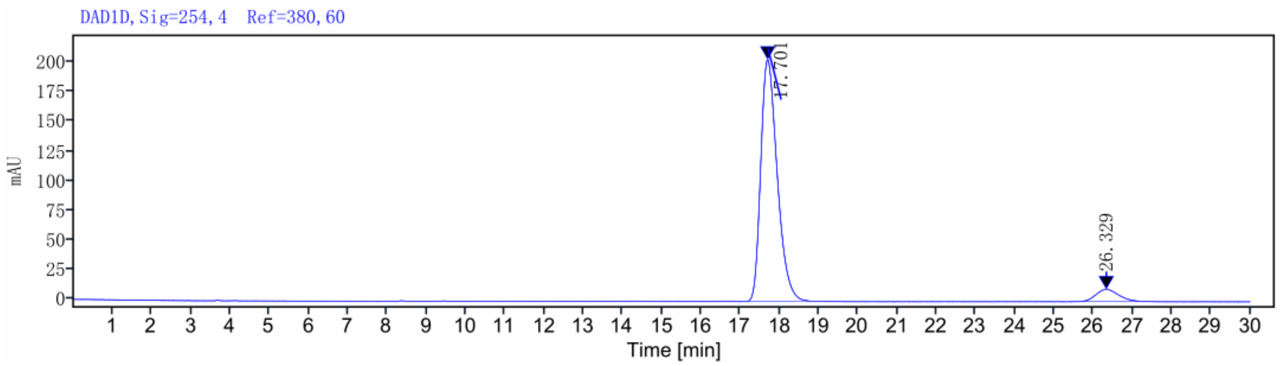

Signal: DAD1D, Sig=254, 4 Ref=380, 60

RetTime [min]	Type	Width [min]	Area [mAU*s]	Height [mAU]	Area %
17.701	VM m	1.53215	5923.53078	203.63630	93.8830
26.329	MM m	1.44807	385.94900	10.03229	6.1170
		Totals	6309.47978		

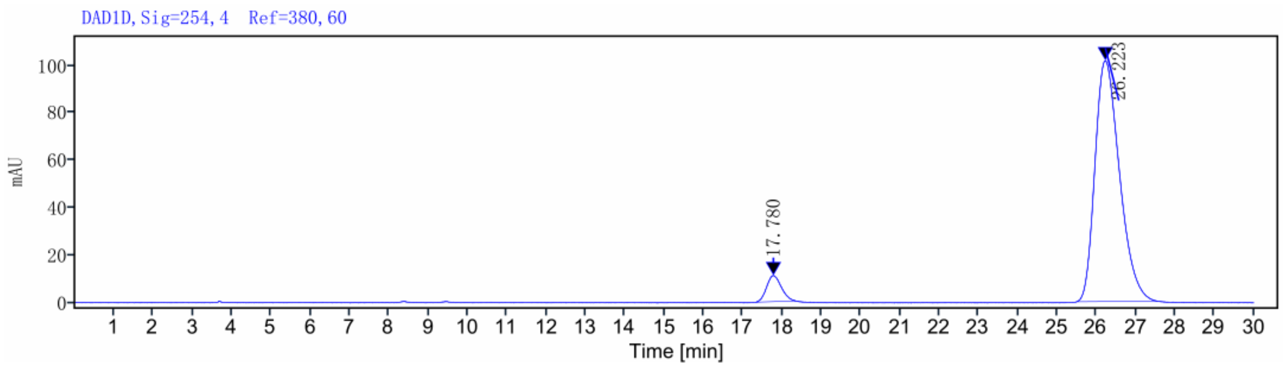

Signal: DAD1D, Sig=254, 4 Ref=380, 60

RetTime [min]	Type	Width [min]	Area [mAU*s]	Height [mAU]	Area %
17.780	MM m	1.01368	283.60878	11.06339	6.0565
26.223	MM m	2.02735	4399.13368	101.67522	93.9435
		Totals	4682.74246		

Supplementary Fig. 102 HPLC data of **22.**

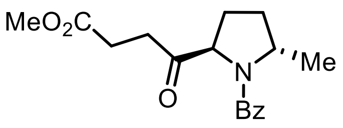

(*S*, *R*)-**L2**: 99:1 dr

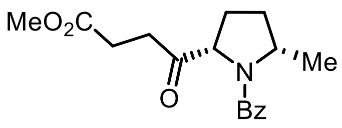

(*R*, *S*)-**L2**: 34:66 dr

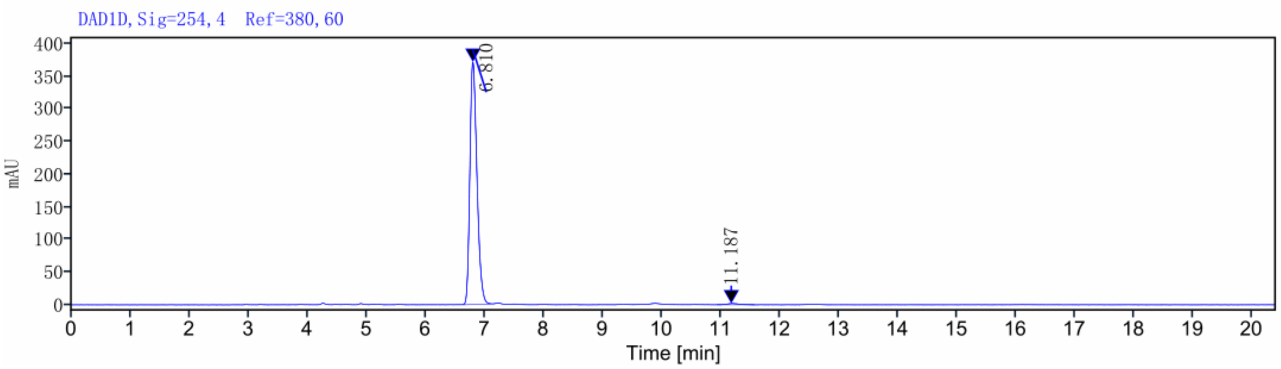

Signal: DAD1D, Sig=254, 4 Ref=380, 60

RetTime [min]	Type	Width [min]	Area [mAU*s]	Height [mAU]	Area %
6.810	VV	0.45353	3087.88920	371.32551	99.2172
11.187	MM m	0.63974	24.36418	1.97725	0.7828
		Totals	3112.25338		

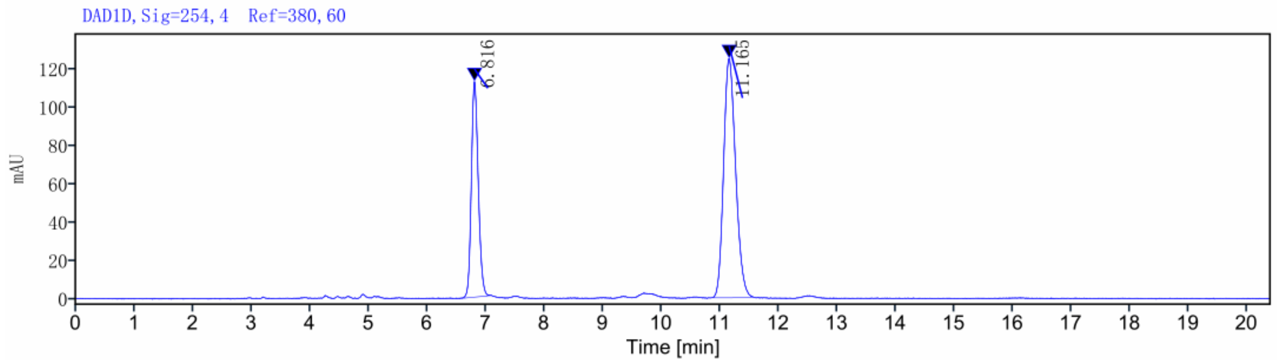

Signal: DAD1D, Sig=254, 4 Ref=380, 60

RetTime [min]	Type	Width [min]	Area [mAU*s]	Height [mAU]	Area %
6.816	VV	0.41081	922.68171	113.15263	34.0736
11.165	VV	0.64798	1785.22930	125.56824	65.9264
		Totals	2707.91101		

Supplementary Fig. 103 HPLC data of **23.**

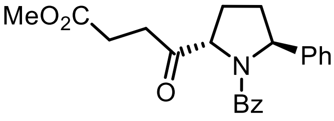

(*S*, *R*)-**L2**: 96:4 dr

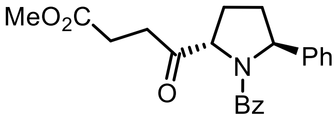

(*R*, *S*)-**L2**: > 99:1 dr

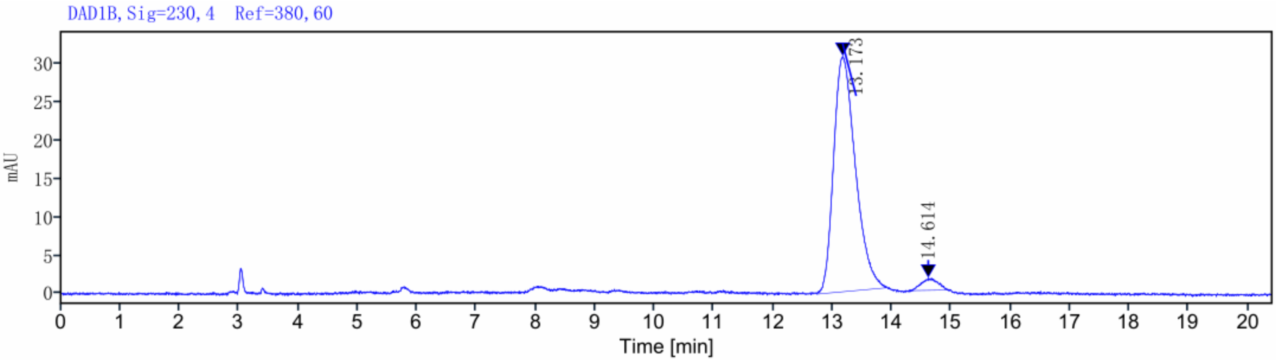

Signal: DAD1B, Sig=230, 4 Ref=380, 60

RetTime [min]	Type	Width [min]	Area [mAU*s]	Height [mAU]	Area %
13.173	MM m	1.15512	786.57900	30.87831	96.3095
14.614	MM m	0.66007	30.14148	1.64486	3.6905
		Totals	816.72047		

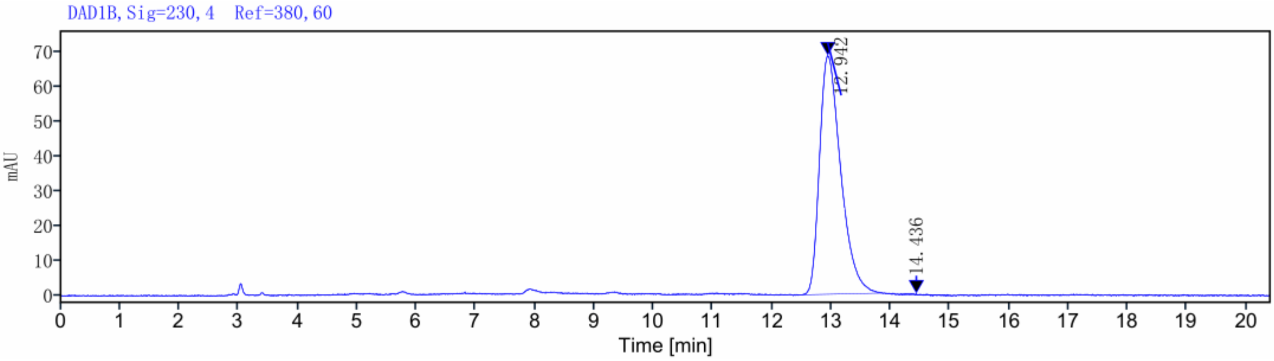

Signal: DAD1B, Sig=230, 4 Ref=380, 60

RetTime [min]	Type	Width [min]	Area [mAU*s]	Height [mAU]	Area %
12.942	VM m	1.31245	1710.33564	68.82133	99.7642
14.436	MM m	0.82508	4.04175	0.41934	0.2358
		Totals	1714.37739		

Supplementary Fig. 104 HPLC data of **24.**

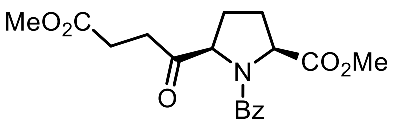

(*S*, *R*)-**L2**: 52:48 dr

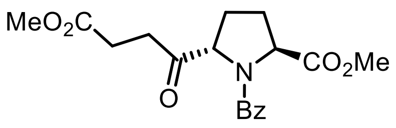

(*R*, *S*)-**L2**: 1:99 dr

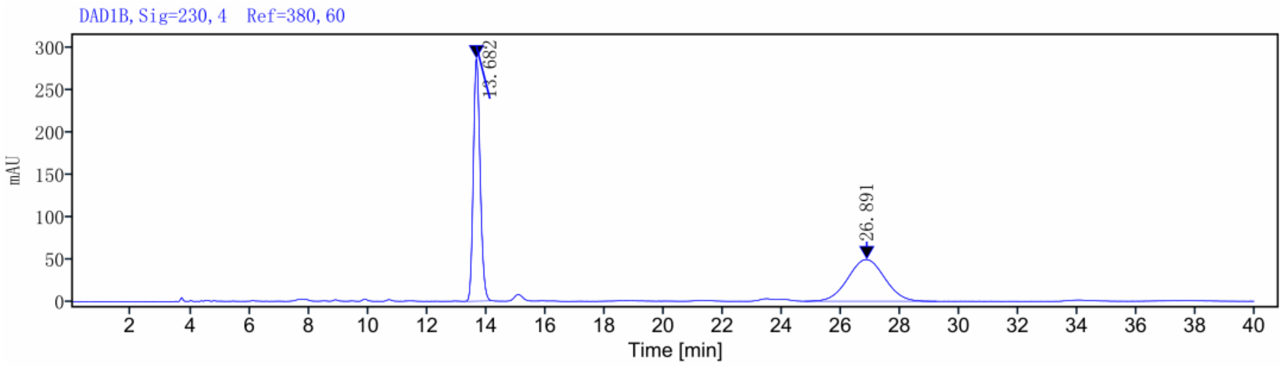

Signal: DAD1B, Sig=230, 4 Ref=380, 60

RetTime [min]	Type	Width [min]	Area [mAU*s]	Height [mAU]	Area %
13.682	VV	0.80128	4616.36806	287.83168	51.8670
26.891	MM m	4.44774	4284.02483	49.70564	48.1330
		Totals	8900.39289		

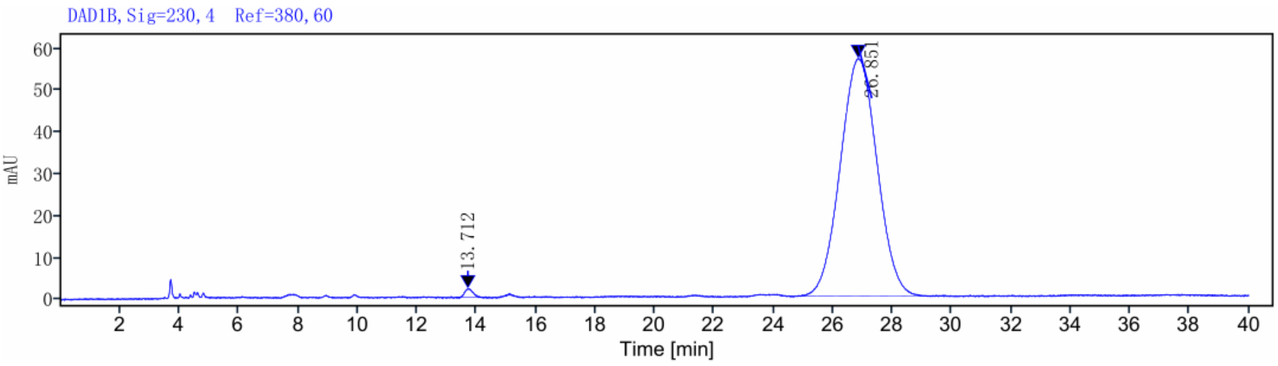

Signal: DAD1B, Sig=230, 4 Ref=380, 60

RetTime [min]	Type	Width [min]	Area [mAU*s]	Height [mAU]	Area %
13.712	MM m	1.06624	39.88872	2.14313	0.8090
26.851	MM m	4.90470	4890.49399	56.84360	99.1910
		Totals	4930.38271		

Supplementary Fig. 105 HPLC data of **25.**

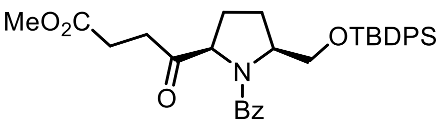

(*S, R*)-**L2**: 67:33 dr

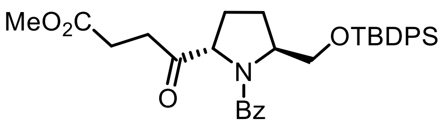

(*R, S*)-**L2**: 1:99 dr

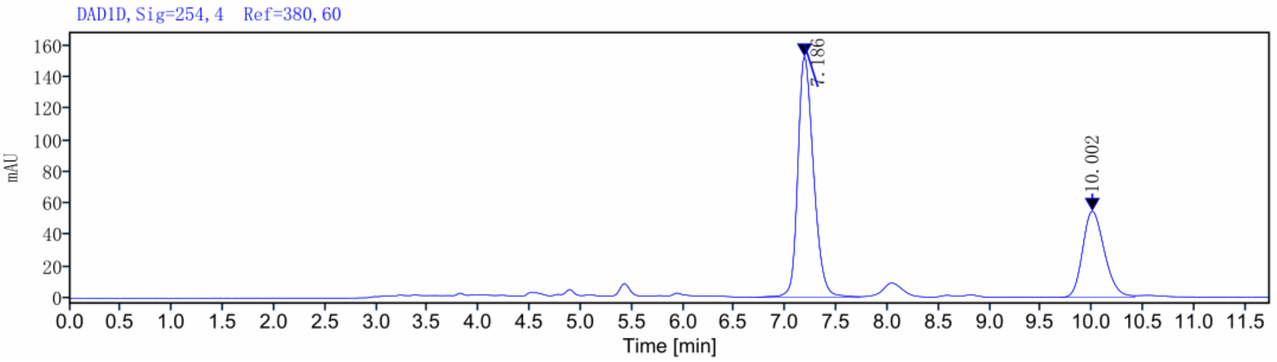

Signal: DAD1D, Sig=254, 4 Ref=380, 60

RetTime [min]	Type	Width [min]	Area [mAU*s]	Height [mAU]	Area %
7.186	BV	1.00983	1633.70195	152.72679	66.9166
10.002	BV	0.73074	807.69875	54.66054	33.0834
		Totals	2441.40071		

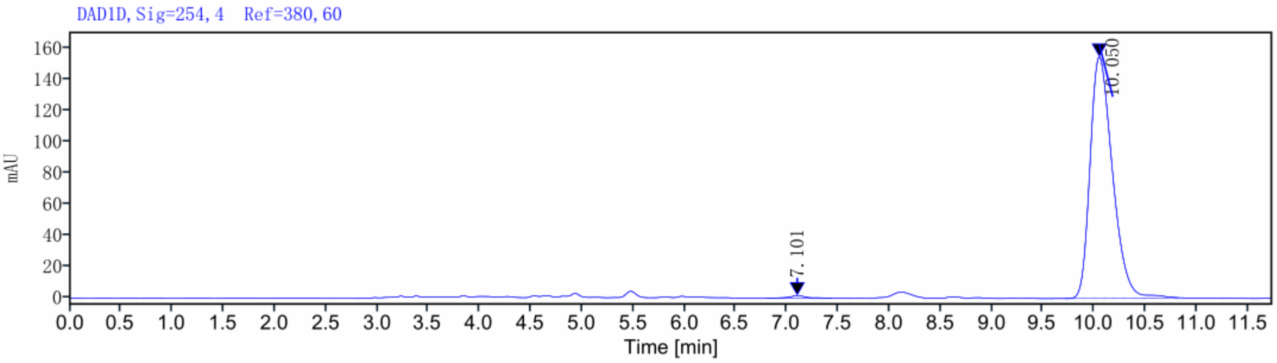

Signal: DAD1D, Sig=254, 4 Ref=380, 60

RetTime [min]	Type	Width [min]	Area [mAU*s]	Height [mAU]	Area %
7.101	MM m	0.65216	18.94590	1.67288	0.8017
10.050	BM m	1.10096	2344.33552	154.43907	99.1983
		Totals	2363.28142		

Supplementary Fig. 106 HPLC data of **26.**

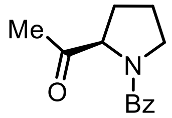

(*S*, *R*)-**L1**: 94% ee

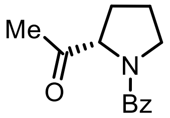

(*R*, *S*)-**L1**: 94% ee

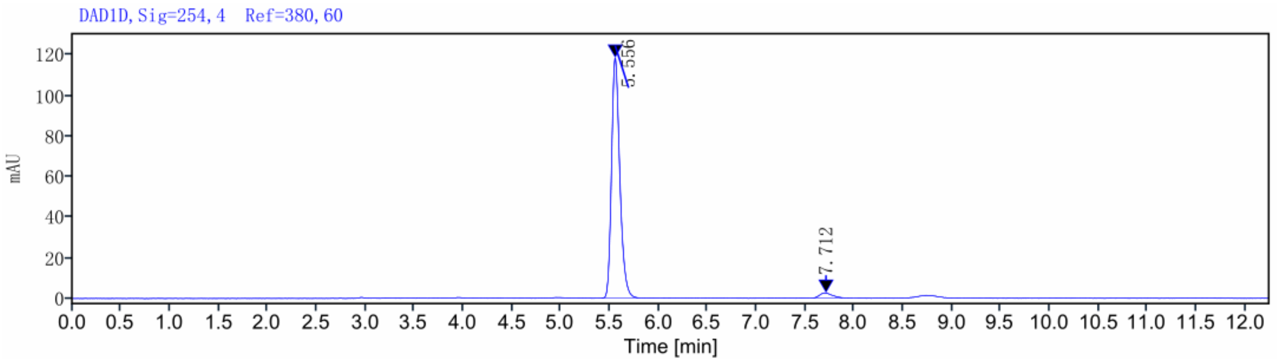

Signal: DAD1D, Sig=254, 4 Ref=380, 60

RetTime [min]	Type	Width [min]	Area [mAU*s]	Height [mAU]	Area %
5.556	VV	0.37425	698.81678	118.69527	96.7589
7.712	VV	0.29021	23.40839	2.60067	3.2411
		Totals	722.22517		

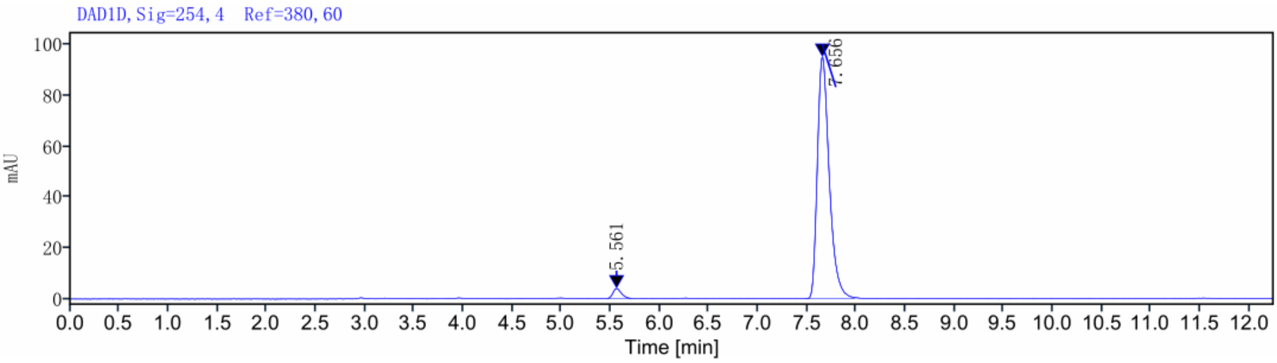

Signal: DAD1D, Sig=254, 4 Ref=380, 60

RetTime [min]	Type	Width [min]	Area [mAU*s]	Height [mAU]	Area %
5.561	VV	0.24457	24.78084	4.04285	3.0078
7.656	BV	0.50747	799.10025	95.19649	96.9922
		Totals	823.88109		

Supplementary Fig. 107 HPLC data of **27.**

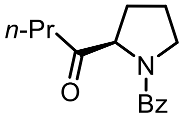

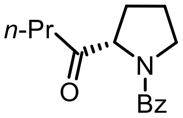

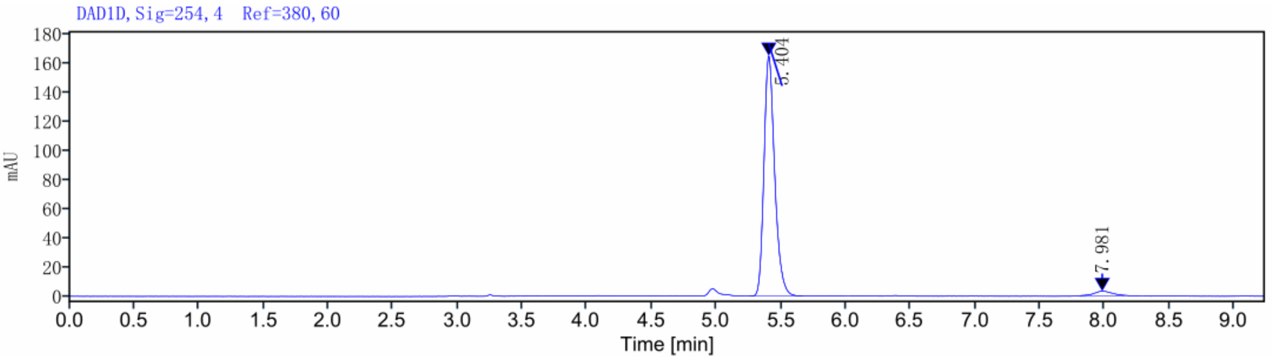

Signal: DAD1D, Sig=254, 4 Ref=380, 60

RetTime [min]	Type	Width [min]	Area [mAU*s]	Height [mAU]	Area %
5.404	BV	0.40709	952.27391	165.26652	96.3349
7.981	VV	0.33758	36.22923	3.43901	3.6651
		Totals	988.50314		

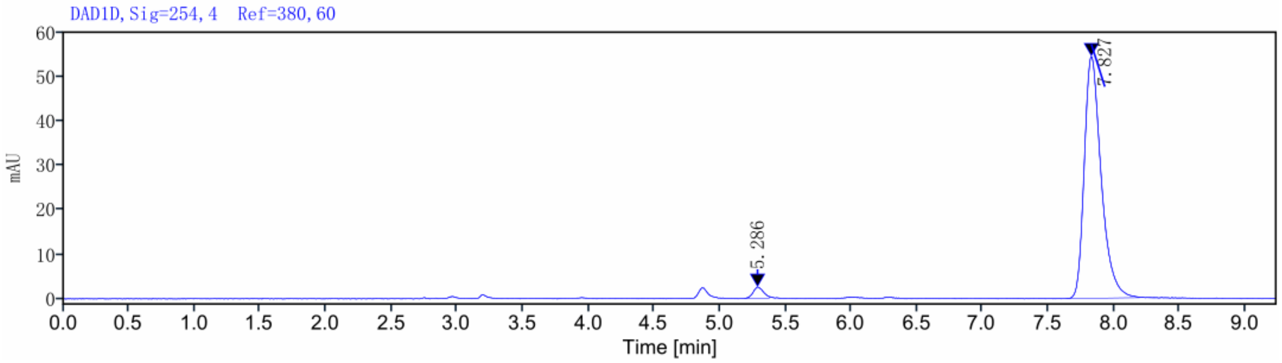

Signal: DAD1D, Sig=254, 4 Ref=380, 60

RetTime [min]	Type	Width [min]	Area [mAU*s]	Height [mAU]	Area %
5.286	BV	0.18995	14.05016	2.56539	2.7967
7.827	BV	0.55768	488.33038	54.45936	97.2033
		Totals	502.38054		

Supplementary Fig. 108 HPLC data of **28.**

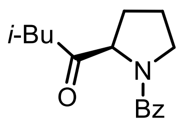

(*S*, *R*)-**L1**: 93% ee

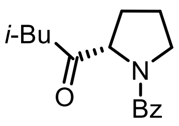

(*R*, *S*)-**L1**: 94% ee

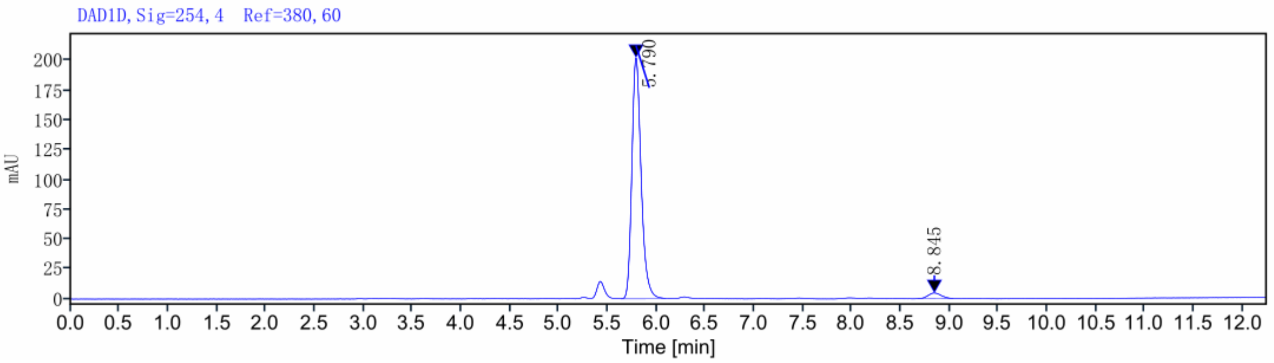

Signal: DAD1D, Sig=254, 4 Ref=380, 60

RetTime [min]	Type	Width [min]	Area [mAU*s]	Height [mAU]	Area %
5.790	BV	0.45248	1335.50494	202.44672	96.6269
8.845	BB m	0.36801	46.62059	4.83386	3.3731
		Totals	1382.12552		

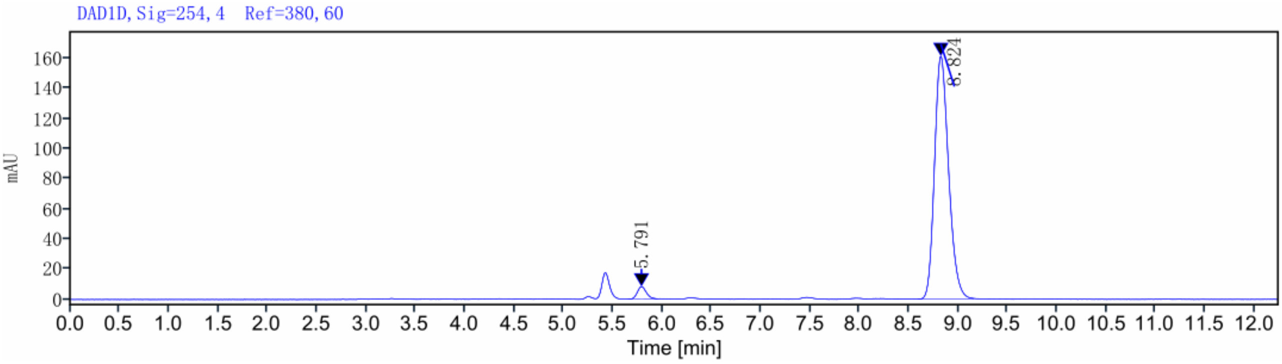

Signal: DAD1D, Sig=254, 4 Ref=380, 60

RetTime [min]	Type	Width [min]	Area [mAU*s]	Height [mAU]	Area %
5.791	VV	0.29554	53.13112	8.27021	3.2137
8.824	VV	0.56145	1600.13445	161.01261	96.7863
		Totals	1653.26558		

Supplementary Fig. 109 HPLC data of **29.**

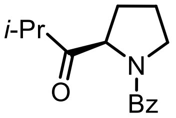

(*S*, *R*)-**L1**: 86% ee

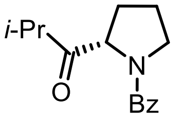

(*R*, *S*)-**L1**: 87% ee

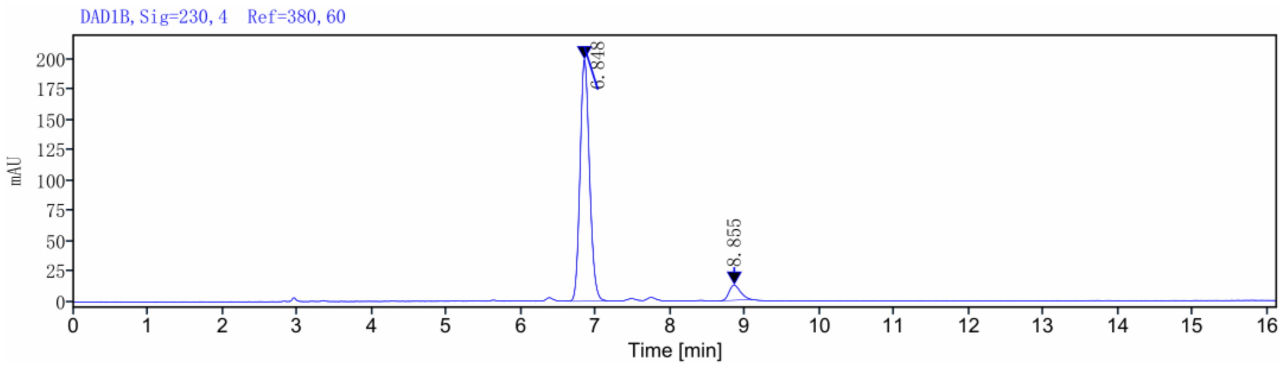

Signal: DAD1B, Sig=230, 4 Ref=380, 60

RetTime [min]	Type	Width [min]	Area [mAU*s]	Height [mAU]	Area %
6.848	VV	0.49419	1763.46906	199.45028	93.0427
8.855	VM m	0.43063	131.86318	12.63707	6.9573
		Totals	1895.33224		

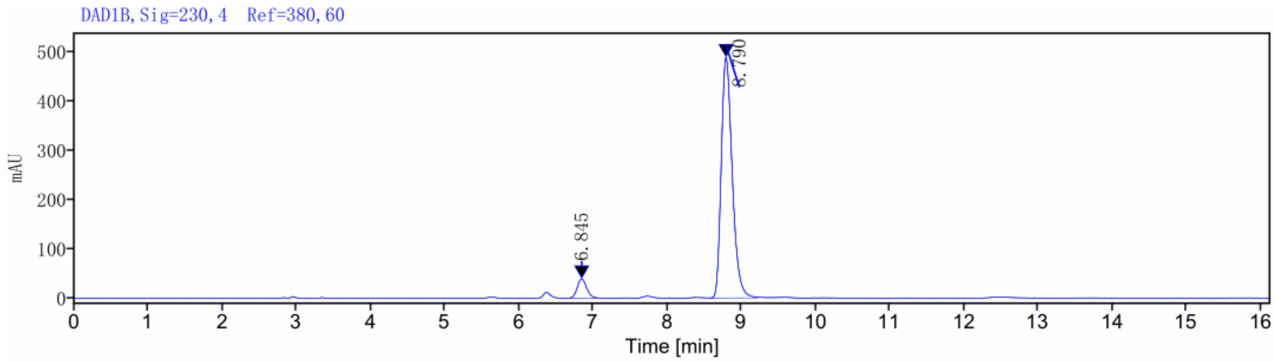

Signal: DAD1B, Sig=230, 4 Ref=380, 60

RetTime [min]	Type	Width [min]	Area [mAU*s]	Height [mAU]	Area %
6.845	VV	0.36608	344.48634	39.32366	6.4378
8.790	BV	0.63397	5006.49717	487.79384	93.5622
		Totals	5350.98351		

Supplementary Fig. 110 HPLC data of **30.**

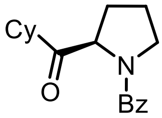

(*S*, *R*)-**L1**: 81% ee

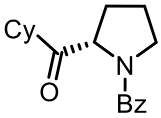

(*R*, *S*)-**L1**: 85% ee

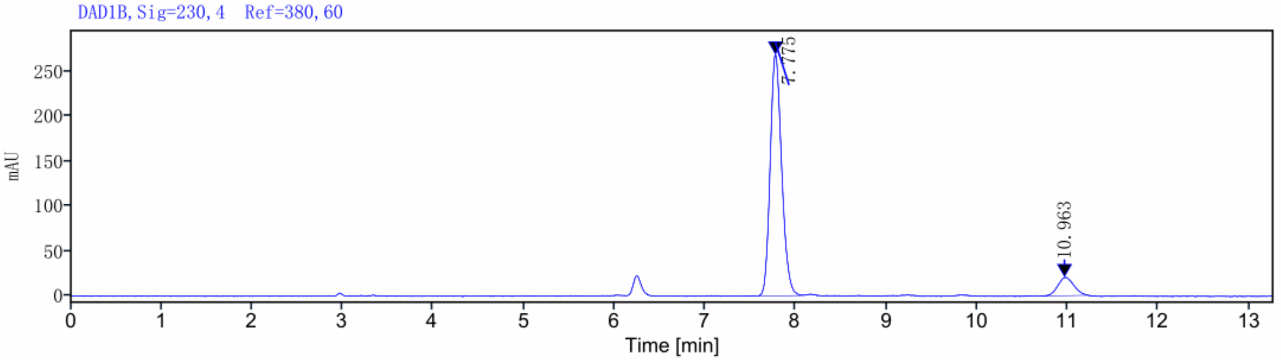

Signal: DAD1B, Sig=230, 4 Ref=380, 60

RetTime [min]	Type	Width [min]	Area [mAU*s]	Height [mAU]	Area %
7.775	VB	0.46081	2367.60883	268.86470	90.4596
10.963	MM m	0.51809	249.70310	20.58129	9.5404
		Totals	2617.31194		

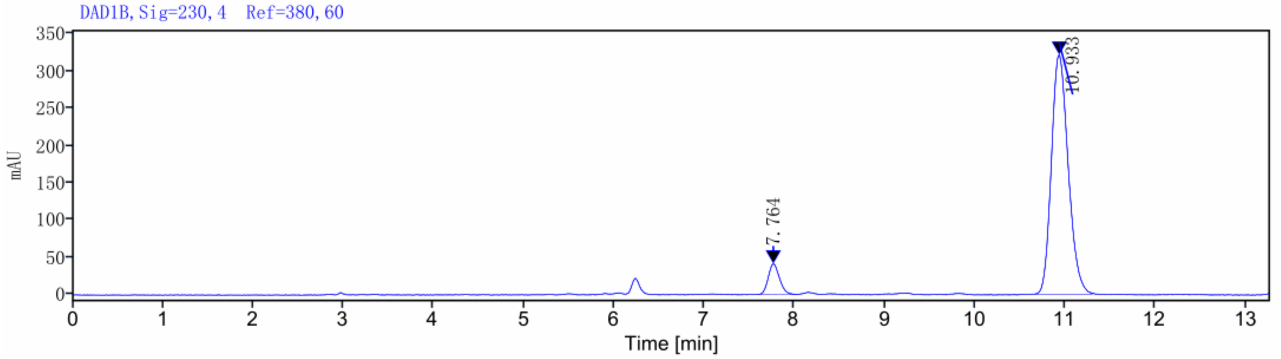

Signal: DAD1B, Sig=230, 4 Ref=380, 60

RetTime [min]	Type	Width [min]	Area [mAU*s]	Height [mAU]	Area %
7.764	MM m	0.46874	345.51462	41.08350	7.6845
10.933	VV	0.62636	4150.73763	320.77381	92.3155
		Totals	4496.25226		

Supplementary Fig. 111 HPLC data of **31.**

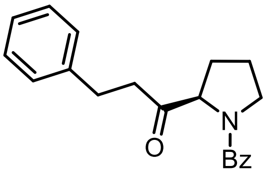

(*S*, *R*)-**L1**: 96% ee

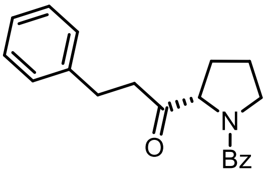

(*R*, *S*)-**L1**: 97% ee

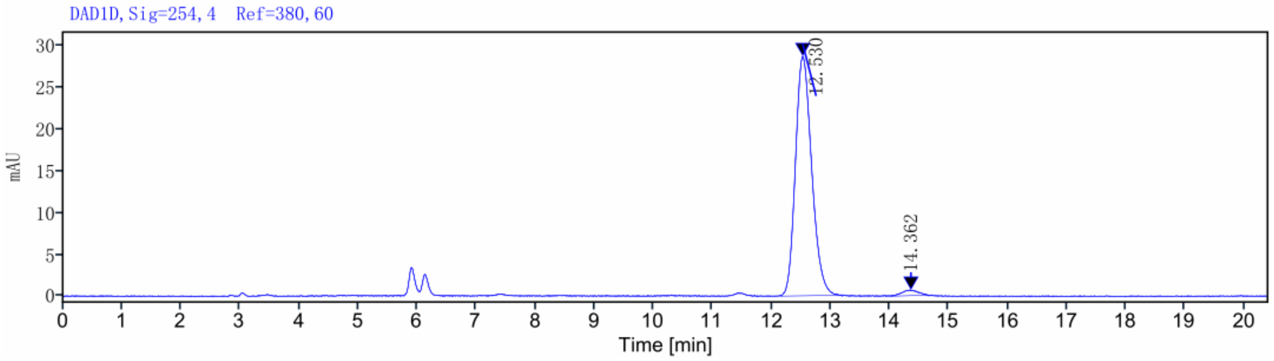

Signal: DAD1D, Sig=254, 4 Ref=380, 60

RetTime [min]	Type	Width [min]	Area [mAU*s]	Height [mAU]	Area %
12.530	VM m	1.02390	549.18363	28.73067	97.7729
14.362	MM m	0.70392	12.50952	0.68577	2.2271
		Totals	561.69315		

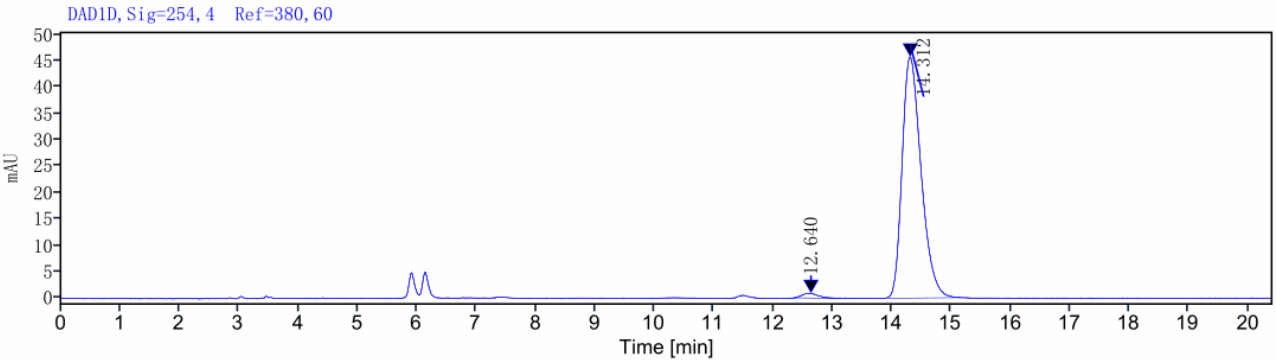

Signal: DAD1D, Sig=254, 4 Ref=380, 60

RetTime [min]	Type	Width [min]	Area [mAU*s]	Height [mAU]	Area %
12.640	MM m	0.76425	17.24428	0.94034	1.6672
14.312	VM m	1.22106	1017.05903	45.81104	98.3328
		Totals	1034.30331		

Supplementary Fig. 112 HPLC data of **32.**

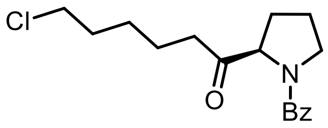

(*S, R*)-**L1**: 97% ee

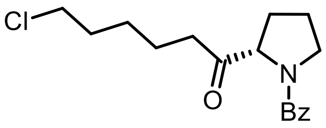

(*R, S*)-**L1**: 96% ee

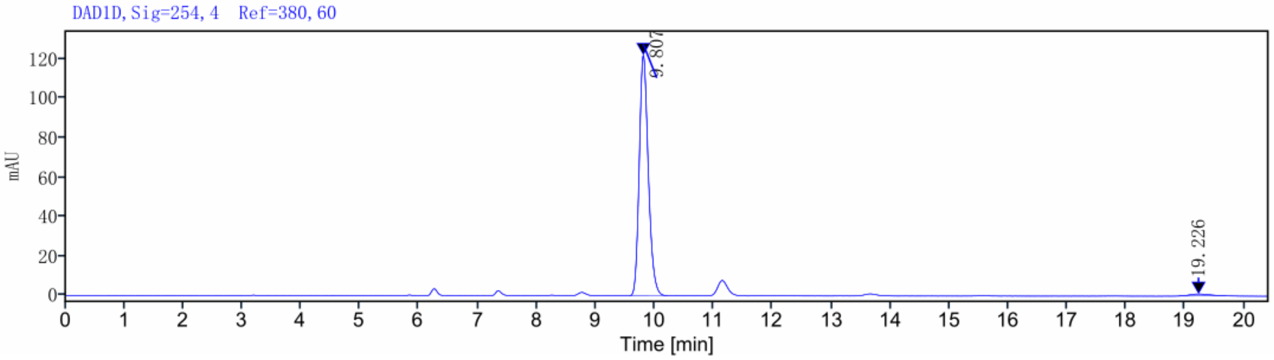

Signal: DAD1D, Sig=254, 4 Ref=380, 60

RetTime [min]	Type	Width [min]	Area [mAU*s]	Height [mAU]	Area %
9.807	VV	0.63791	1304.98878	122.07805	98.2818
19.226	MM m	0.80128	22.81411	1.00421	1.7182
		Totals	1327.80289		

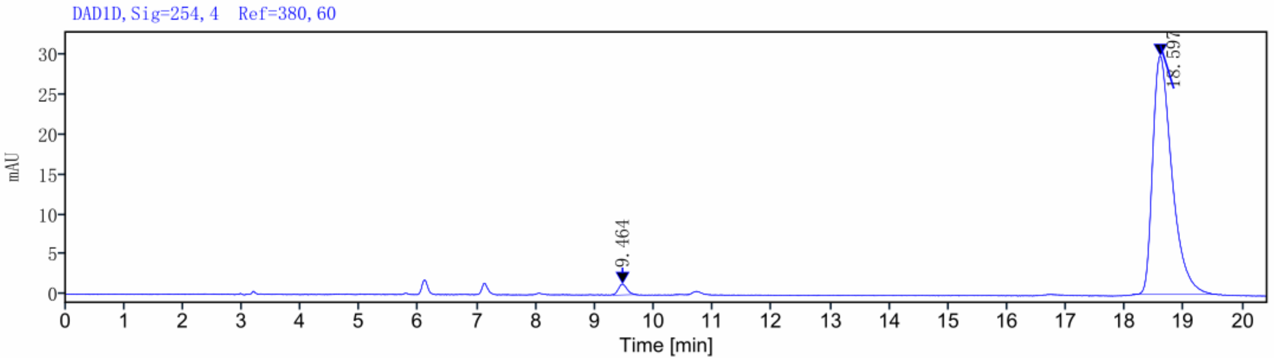

Signal: DAD1D, Sig=254, 4 Ref=380, 60

RetTime [min]	Type	Width [min]	Area [mAU*s]	Height [mAU]	Area %
9.464	VM m	0.40185	13.66942	1.36043	1.9669
18.597	MM m	1.42445	681.30845	29.89889	98.0331
		Totals	694.97787		

Supplementary Fig. 113 HPLC data of **33.**

Supplementary Fig. 114 HPLC data of **34**.

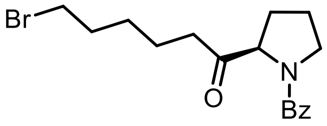

(*S*, *R*)-**L1**: 96% ee

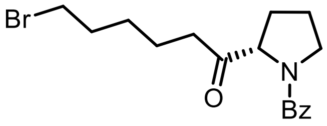

(*R*, *S*)-**L1**: 96% ee

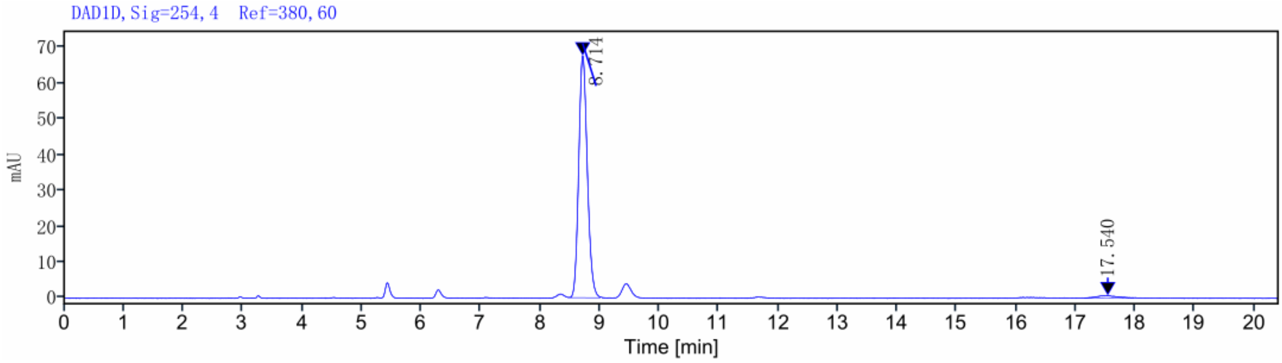

Signal: DAD1D, Sig=254, 4 Ref=380, 60

RetTime [min]	Type	Width [min]	Area [mAU*s]	Height [mAU]	Area %
8.714	VV	0.49550	676.57072	67.54477	97.9818
17.540	MM m	0.84473	13.93612	0.70084	2.0182
		Totals	690.50683		

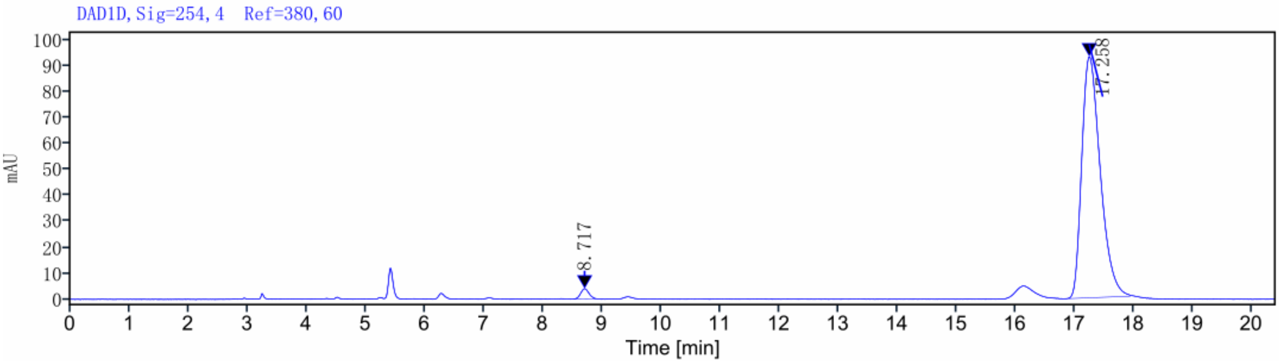

Signal: DAD1D, Sig=254, 4 Ref=380, 60

RetTime [min]	Type	Width [min]	Area [mAU*s]	Height [mAU]	Area %
8.717	BV	0.35232	40.00116	4.09344	1.9368
17.258	VV	1.03740	2025.32609	93.09732	98.0632
		Totals	2065.32726		

Supplementary Fig. 114 HPLC data of **34.**

Supplementary Fig. 115 HPLC data of **35**.

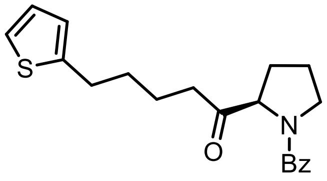

(*S*, *R*)-**L1**: 97% ee

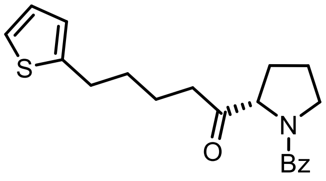

(*R*, *S*)-**L1**: 97% ee

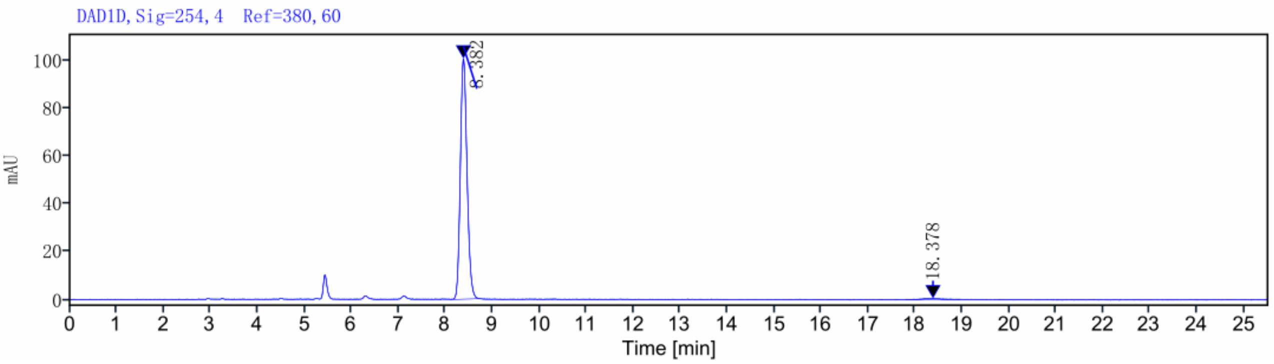

Signal: DAD1D, Sig=254, 4 Ref=380, 60

RetTime [min]	Type	Width [min]	Area [mAU*s]	Height [mAU]	Area %
8.382	VM m	0.56663	994.32686	100.64328	98.7112
18.378	MM m	1.18262	12.98222	0.48938	1.2888
		Totals	1007.30908		

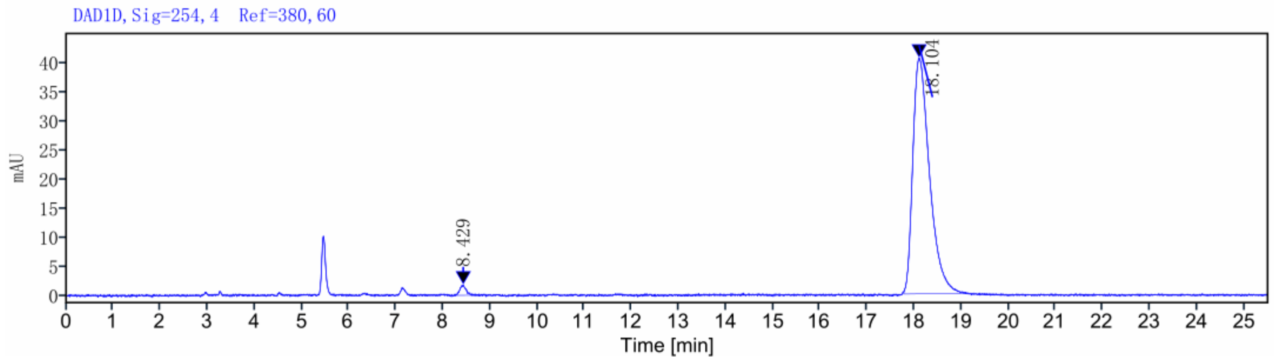

Signal: DAD1D, Sig=254, 4 Ref=380, 60

RetTime [min]	Type	Width [min]	Area [mAU*s]	Height [mAU]	Area %
8.429	MM m	0.45856	16.51513	1.86178	1.6352
18.104	VM m	1.33631	993.48399	40.42462	98.3648
		Totals	1009.99913		

Supplementary Fig. 115 HPLC data of **35**.

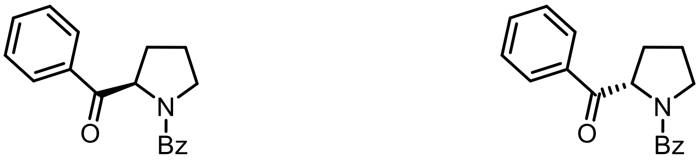

(*S*, *R*)-**L2**: 96% ee (*R*, *S*)-**L2**: 96% ee

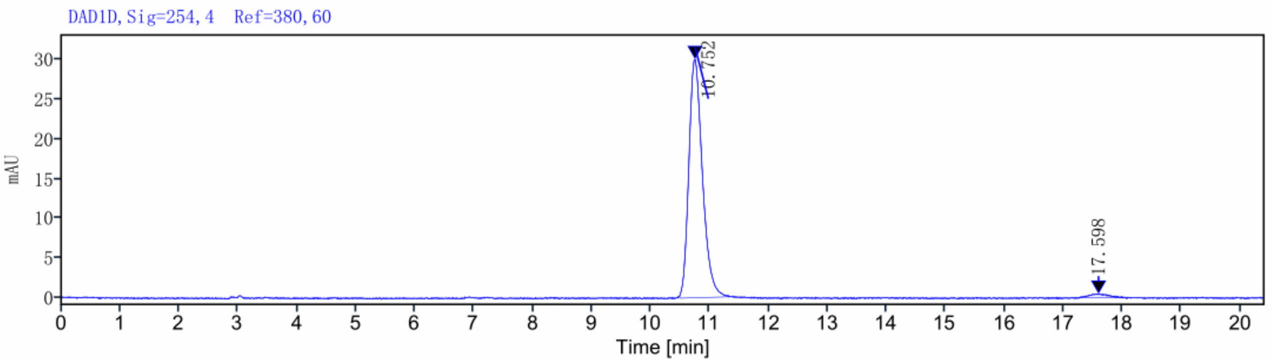

Signal: DAD1D, Sig=254, 4 Ref=380, 60

RetTime [min]	Type	Width [min]	Area [mAU*s]	Height [mAU]	Area %
10.752	VM m	0.85327	482.59662	30.14648	97.7820
17.598	MM m	0.86884	10.94660	0.50110	2.2180
		Totals	493.54323		

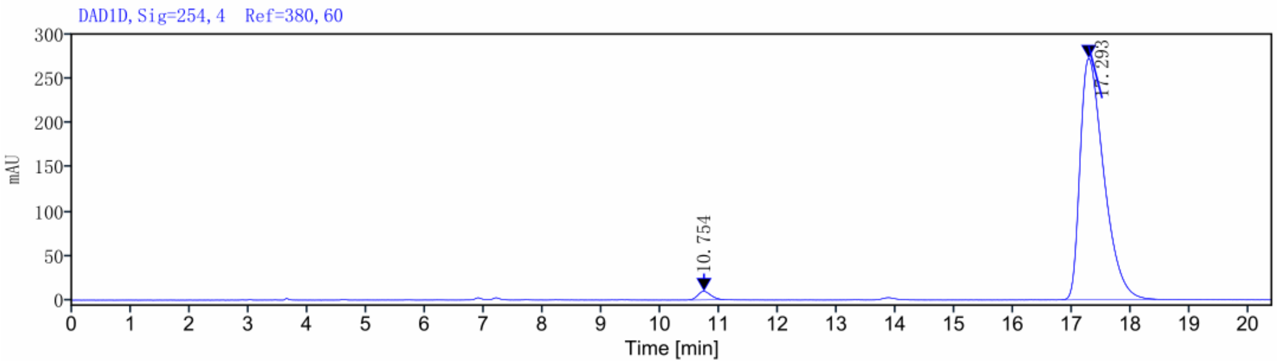

Signal: DAD1D, Sig=254, 4 Ref=380, 60

RetTime [min]	Type	Width [min]	Area [mAU*s]	Height [mAU]	Area %
10.754	VM m	0.59643	150.07360	9.75568	1.9207
17.293	BM m	1.60708	7663.47998	273.35980	98.0793
		Totals	7813.55358		

Supplementary Fig. 116 HPLC data of **36.**

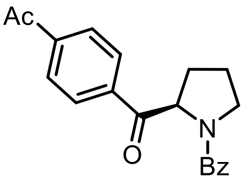

(*S*, *R*)-**L2**: 98% ee

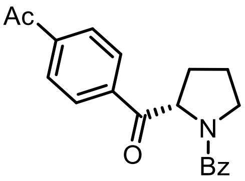

(*R*, *S*)-**L2**: 98% ee

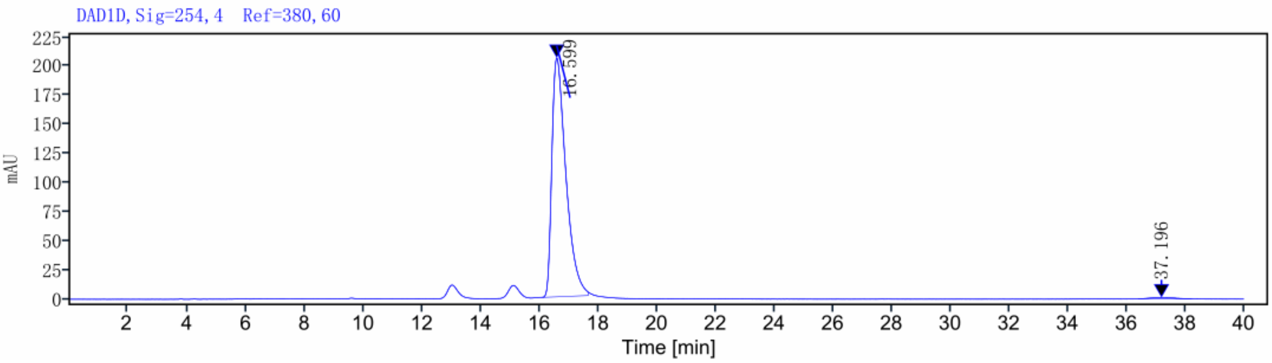

Signal: DAD1D, Sig=254, 4 Ref=380, 60

RetTime [min]	Type	Width [min]	Area [mAU*s]	Height [mAU]	Area %
16.599	VV	1.54775	6816.08412	205.29648	99.0429
37.196	MM m	1.83423	65.86657	1.20273	0.9571
		Totals	6881.95069		

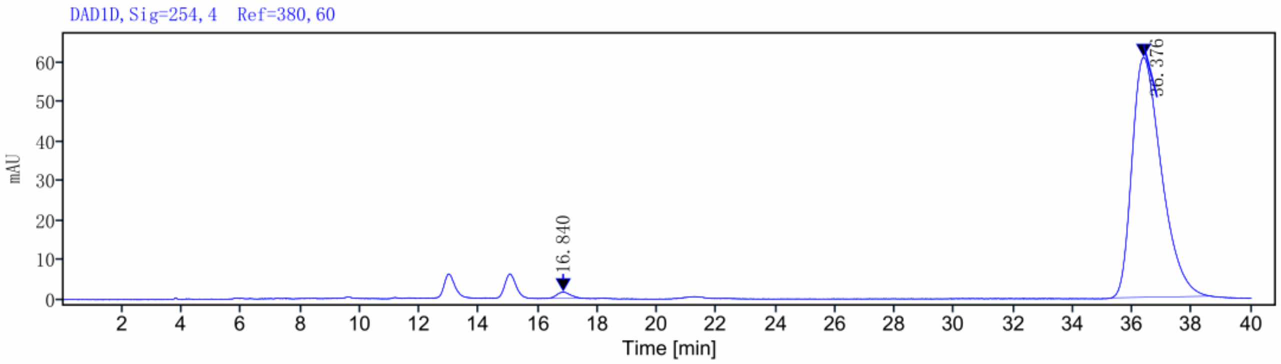

Signal: DAD1D, Sig=254, 4 Ref=380, 60

RetTime [min]	Type	Width [min]	Area [mAU*s]	Height [mAU]	Area %
16.840	MM m	1.09410	45.08716	1.60855	1.0661
36.376	MM m	3.37885	4184.14714	60.72781	98.9339
		Totals	4229.23430		

Supplementary Fig. 117 HPLC data of **37.**

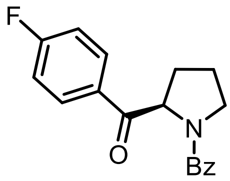

(*S*, *R*)-**L2**: 96% ee

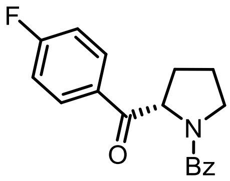

(*R*, *S*)-**L2**: 97% ee

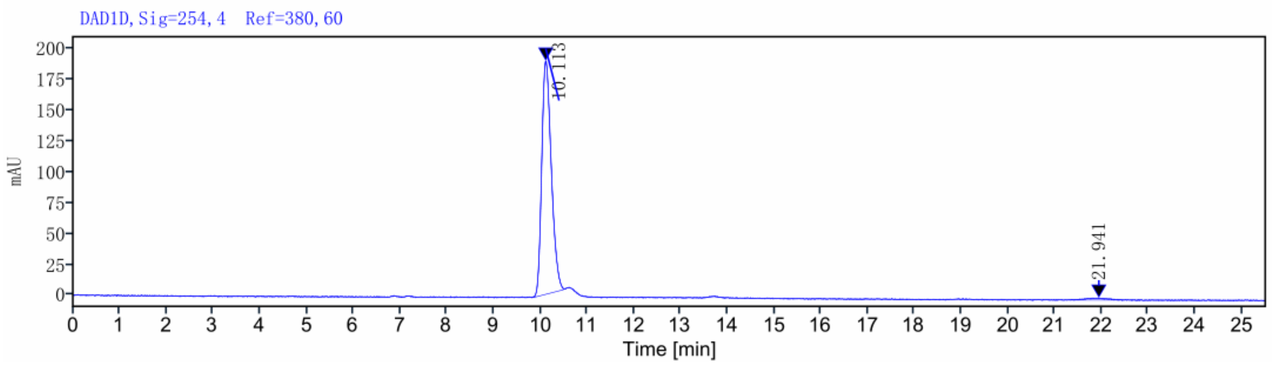

Signal: DAD1D, Sig=254, 4 Ref=380, 60

RetTime [min]	Type	Width [min]	Area [mAU*s]	Height [mAU]	Area %
10.113	VV	0.63371	2690.48132	189.43929	98.1090
21.941	MM m	1.06083	51.85848	1.78839	1.8910
		Totals	2742.33980		

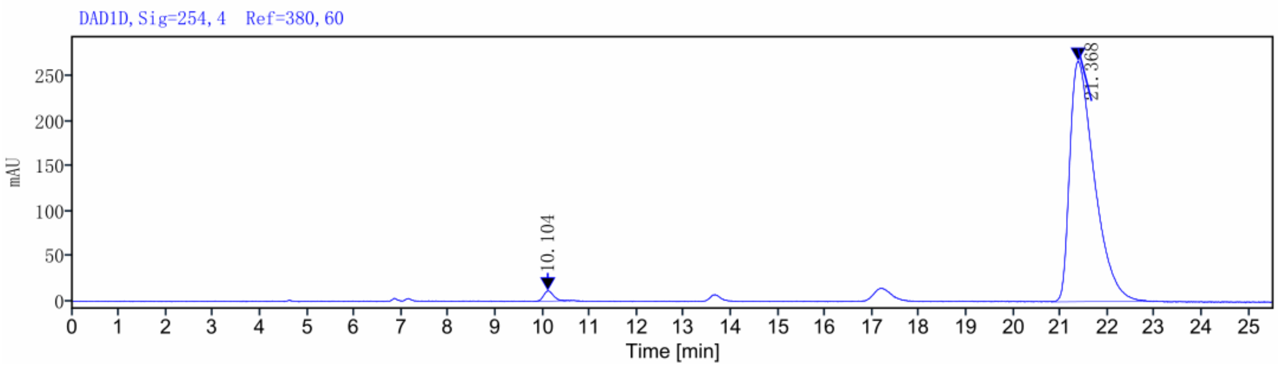

Signal: DAD1D, Sig=254, 4 Ref=380, 60

RetTime [min]	Type	Width [min]	Area [mAU*s]	Height [mAU]	Area %
10.104	MM m	0.69077	166.97333	11.74276	1.6893
21.368	MM m	2.12165	9717.01877	268.19721	98.3107
		Totals	9883.99211		

Supplementary Fig. 118 HPLC data of **38.**

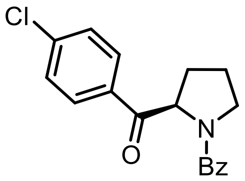

(*S*, *R*)-**L2**: 97% ee

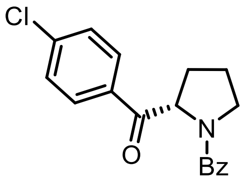

(*R*, *S*)-**L2**: 97% ee

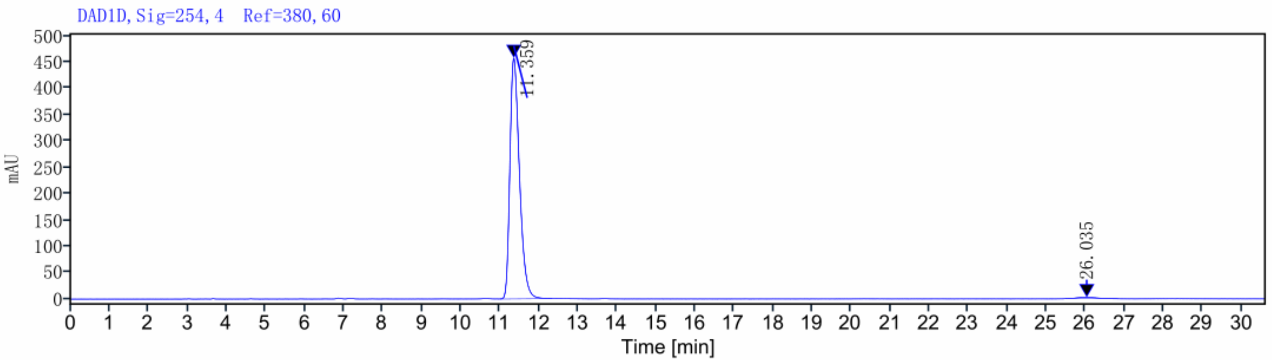

Signal: DAD1D, Sig=254, 4 Ref=380, 60

RetTime [min]	Type	Width [min]	Area [mAU*s]	Height [mAU]	Area %
11.359	BM m	1.32019	7788.64812	457.68220	98.6987
26.035	MM m	1.35154	102.69081	2.81060	1.3013
		Totals	7891.33893		

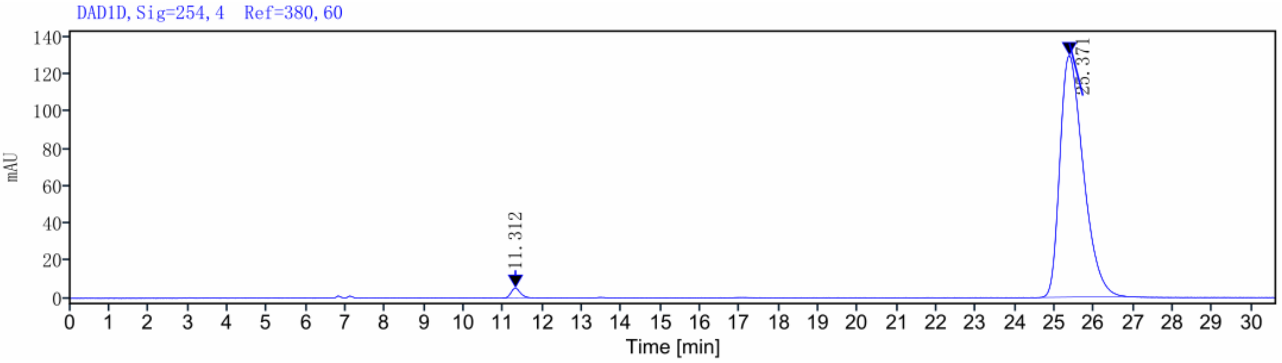

Signal: DAD1D, Sig=254, 4 Ref=380, 60

RetTime [min]	Type	Width [min]	Area [mAU*s]	Height [mAU]	Area %
11.312	MM m	0.64761	81.47725	5.18887	1.4967
25.371	VM m	2.11427	5362.34417	129.77239	98.5033
		Totals	5443.82141		

Supplementary Fig. 119 HPLC data of **39.**

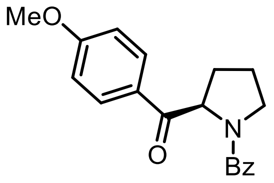

(*S*, *R*)-**L2**: 97% ee

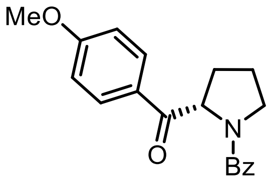

(*R*, *S*)-**L2**: 96% ee

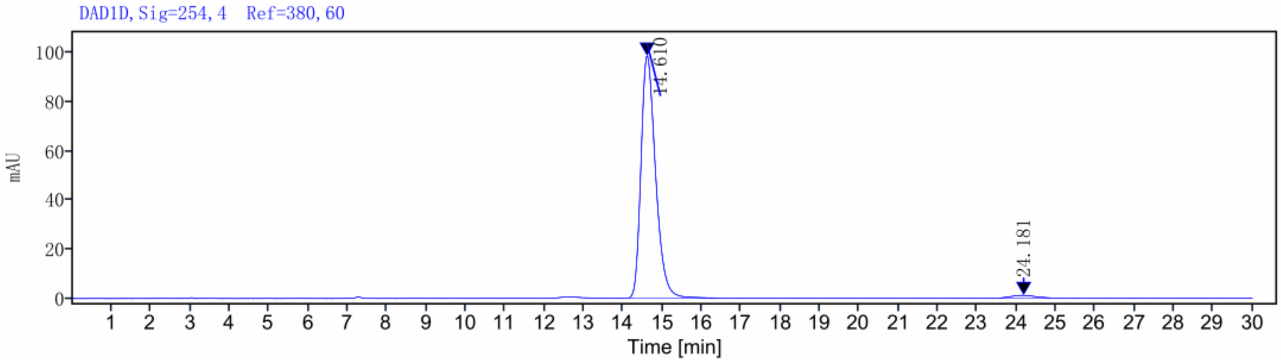

Signal: DAD1D, Sig=254, 4 Ref=380, 60

RetTime [min]	Type	Width [min]	Area [mAU*s]	Height [mAU]	Area %
14.610	VM m	2.21891	2549.28403	98.91125	98.3248
24.181	MM m	1.34371	43.43287	1.10879	1.6752
		Totals	2592.71690		

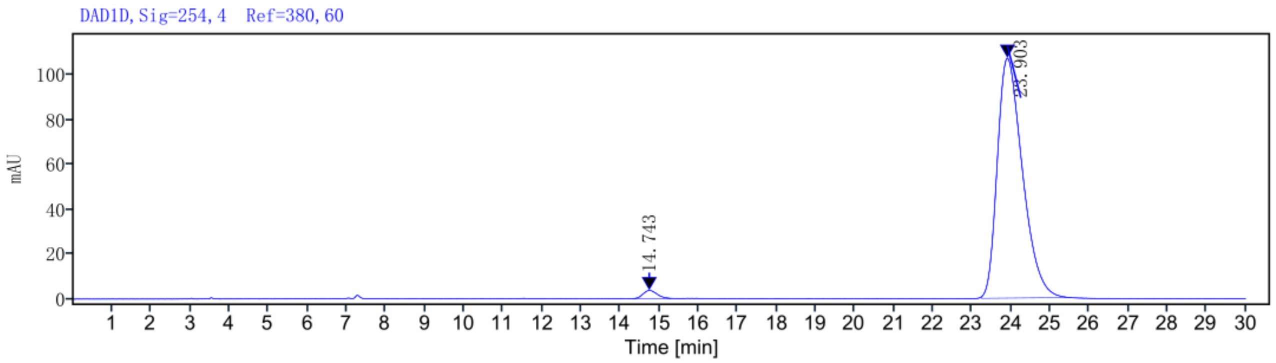

Signal: DAD1D, Sig=254, 4 Ref=380, 60

RetTime [min]	Type	Width [min]	Area [mAU*s]	Height [mAU]	Area %
14.743	MM m	0.96538	88.79961	3.67924	1.8177
23.903	VM m	2.29624	4796.57299	107.33720	98.1823
		Totals	4885.37259		

Supplementary Fig. 120 HPLC data of **40.**

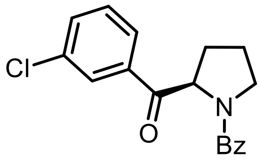

(*S*, *R*)-**L2**: 97% ee

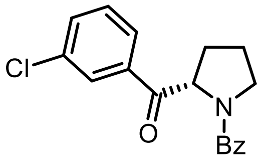

(*R*, *S*)-**L2**: 97% ee

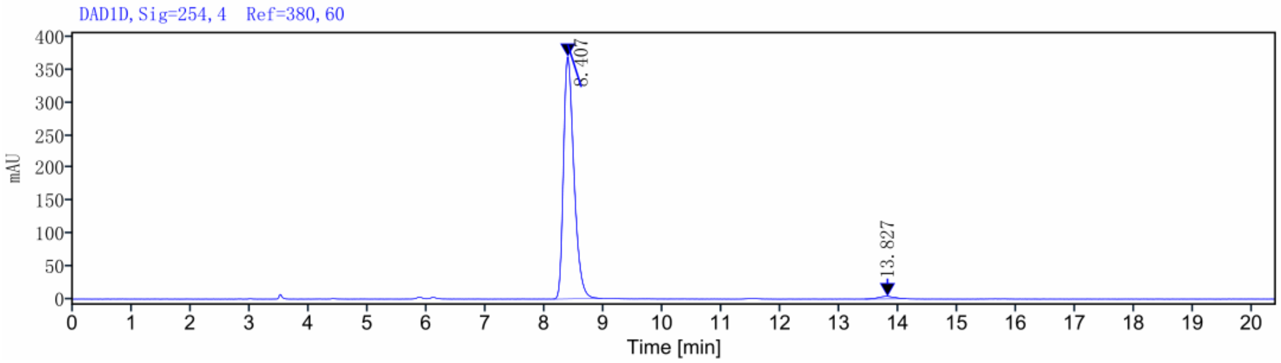

Signal: DAD1D, Sig=254, 4 Ref=380, 60

RetTime [min]	Type	Width [min]	Area [mAU*s]	Height [mAU]	Area %
8.407	VM m	0.82502	4554.08616	369.61203	98.4033
13.827	MM m	0.69992	73.89686	3.87288	1.5967
		Totals	4627.98303		

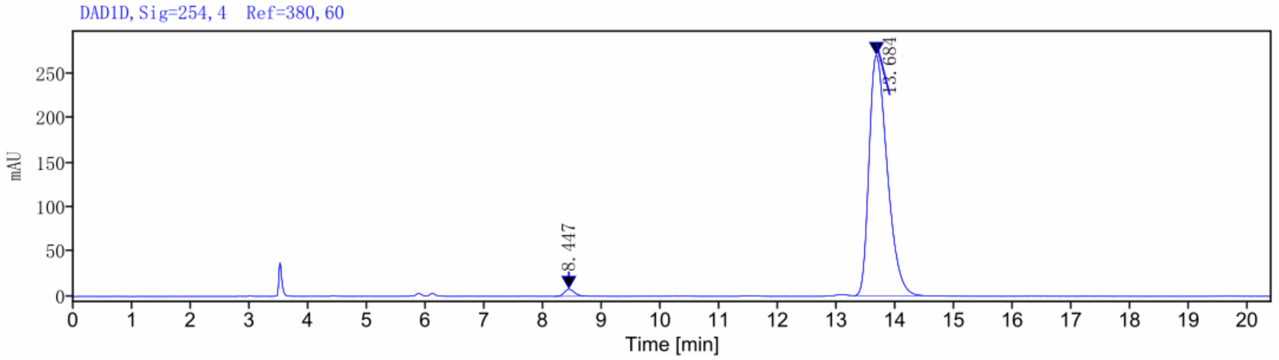

Signal: DAD1D, Sig=254, 4 Ref=380, 60

RetTime [min]	Type	Width [min]	Area [mAU*s]	Height [mAU]	Area %
8.447	MM m	0.53096	95.40458	8.04617	1.6149
13.684	VV	1.13962	5812.28794	270.11958	98.3851
		Totals	5907.69252		

Supplementary Fig. 121 HPLC data of **41.**

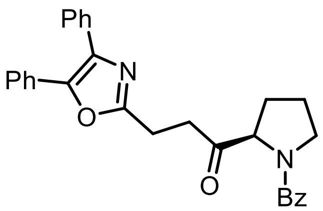

(*S*, *R*)-**L1**: 99% ee

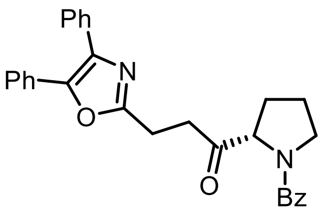

(*R*, *S*)-**L1**: 98% ee

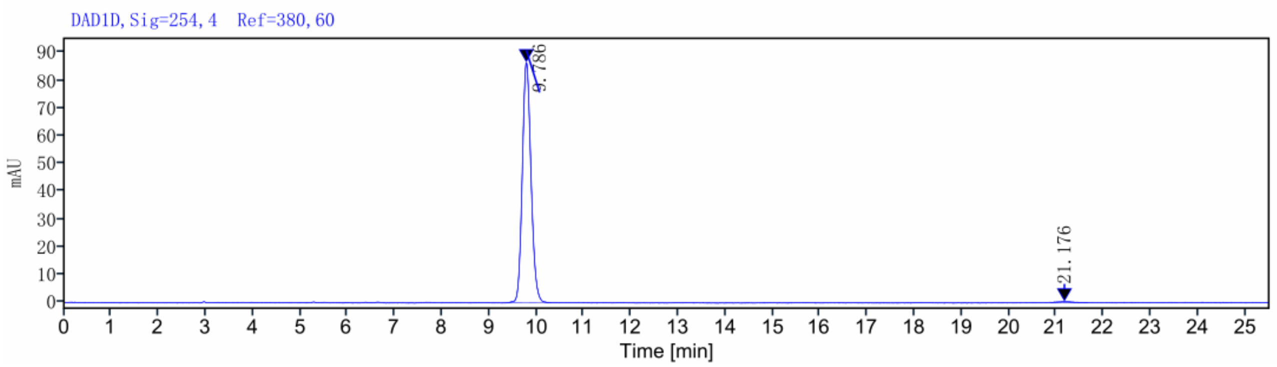

Signal: DAD1D, Sig=254, 4 Ref=380, 60

RetTime [min]	Type	Width [min]	Area [mAU*s]	Height [mAU]	Area %
9.786	MM m	0.91714	1105.52689	86.62842	99.3146
21.176	MM m	0.79646	7.62917	0.40497	0.6854
		Totals	1113.15606		

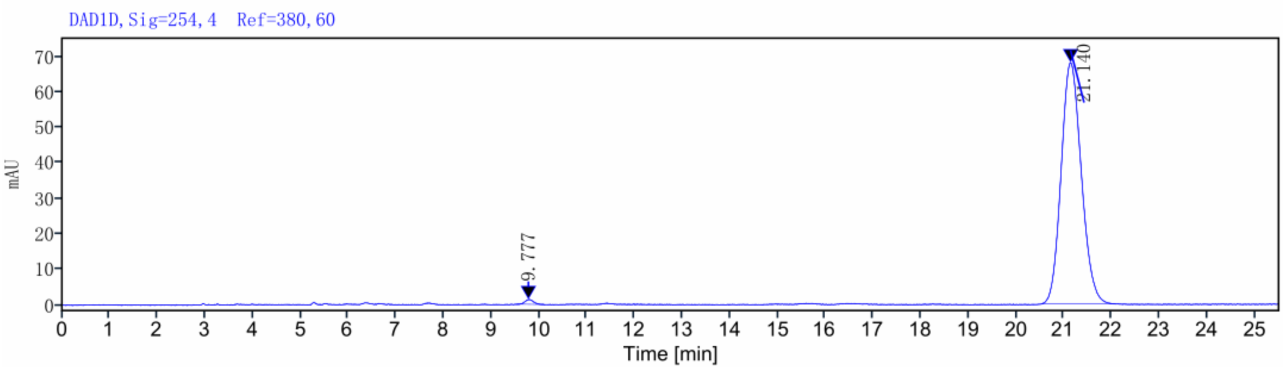

Signal: DAD1D, Sig=254, 4 Ref=380, 60

RetTime [min]	Type	Width [min]	Area [mAU*s]	Height [mAU]	Area %
9.777	MM m	0.57924	15.94481	1.40743	0.7924
21.140	MM m	1.54465	1996.38834	67.96316	99.2076
		Totals	2012.33315		

Supplementary Fig. 122 HPLC data of **42**.

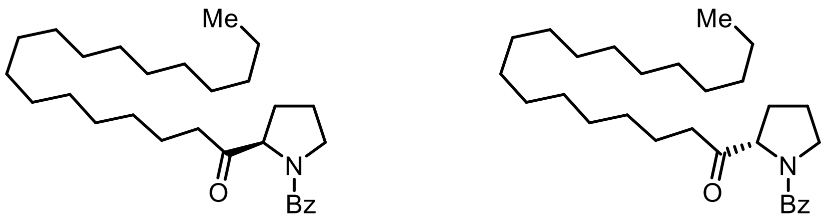

(*S*, *R*)-**L1**: 94% ee (*R*, *S*)-**L1**: 92% ee

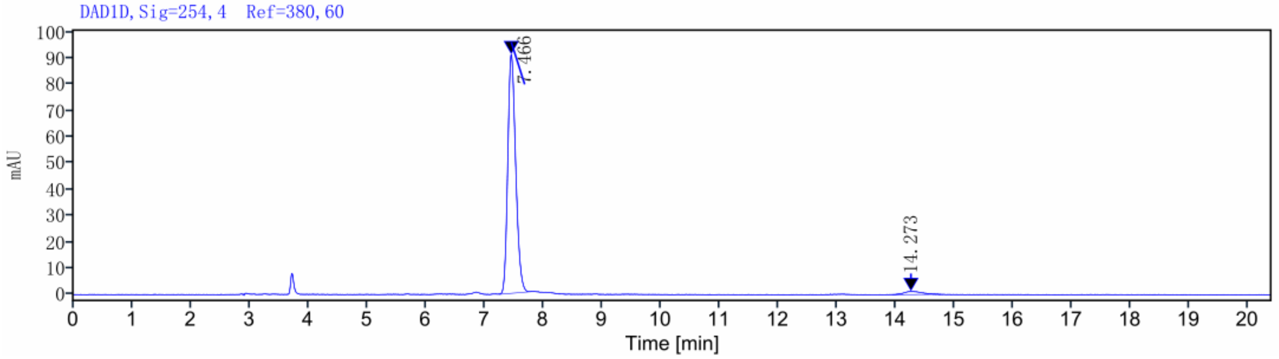

Signal: DAD1D, Sig=254, 4 Ref=380, 60

RetTime [min]	Type	Width [min]	Area [mAU*s]	Height [mAU]	Area %
7.466	BV	0.48816	853.21477	91.10440	97.1579
14.273	MM m	0.82508	24.95824	1.26856	2.8421
		Totals	878.17301		

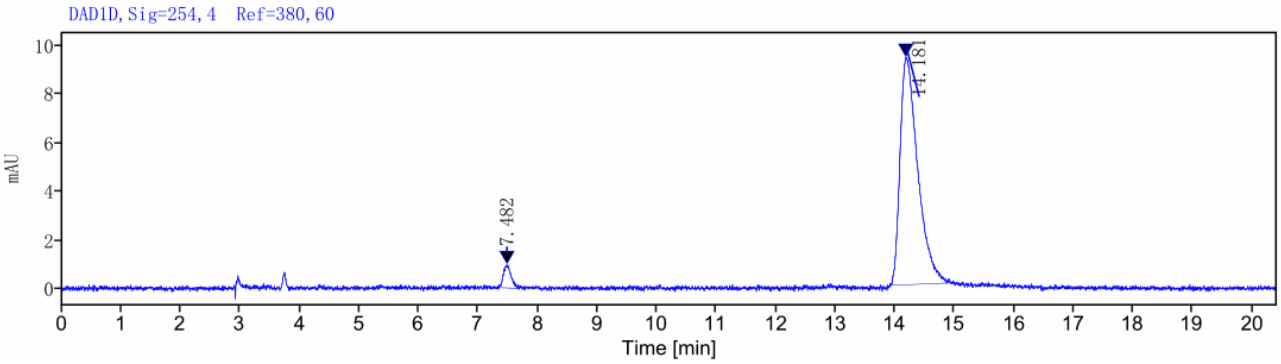

Signal: DAD1D, Sig=254, 4 Ref=380, 60

RetTime [min]	Type	Width [min]	Area [mAU*s]	Height [mAU]	Area %
7.482	MM m	0.36304	8.50605	0.97334	4.2283
14.181	VM m	0.99038	192.66292	9.36685	95.7717
		Totals	201.16897		

Supplementary Fig. 123 HPLC data of **43**.

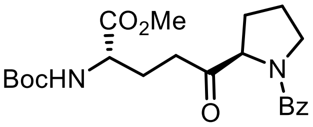

(*S*, *R*)-**L1**: 99:1 dr

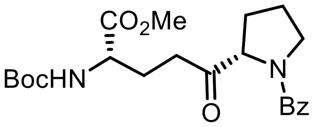

(*R*, *S*)-**L1**: 2:98 dr

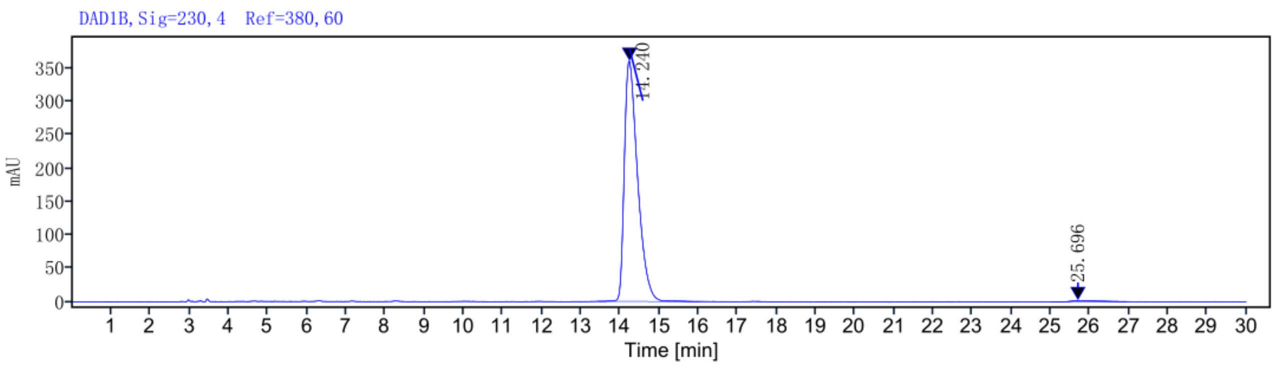

Signal: DAD1B, Sig=230, 4 Ref=380, 60

RetTime [min]	Type	Width [min]	Area [mAU*s]	Height [mAU]	Area %
14.240	MM m	2.59252	8454.43703	359.92132	98.9379
25.696	MM m	1.95762	90.76034	2.44789	1.0621
		Totals	8545.19736		

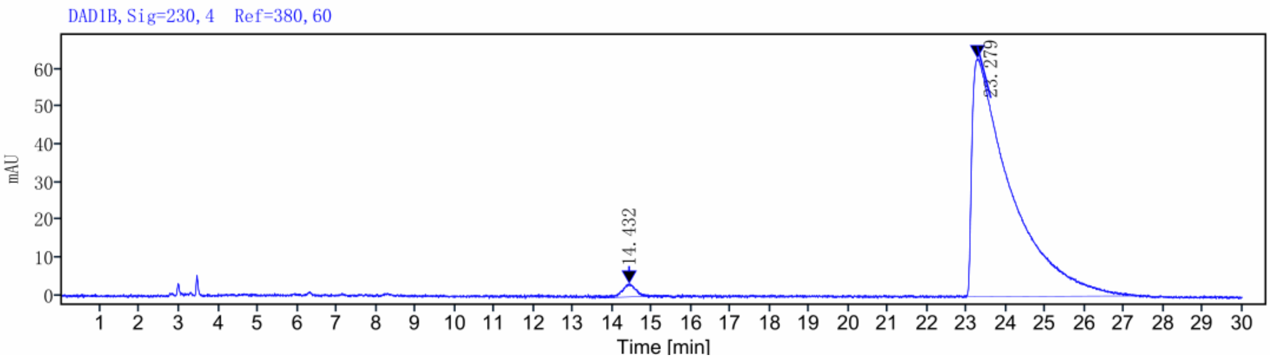

Signal: DAD1B, Sig=230, 4 Ref=380, 60

RetTime [min]	Type	Width [min]	Area [mAU*s]	Height [mAU]	Area %
14.432	MM m	1.71953	82.36731	3.47274	1.8839
23.279	MM m	4.52368	4289.69539	63.13946	98.1161
		Totals	4372.06270		

Supplementary Fig. 124 HPLC data of **44** and **45**.

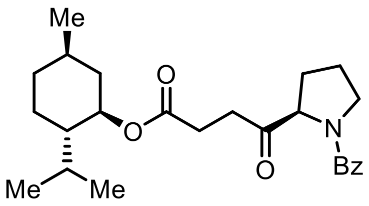

(*S*, *R*)-**L1**: 98:2 dr

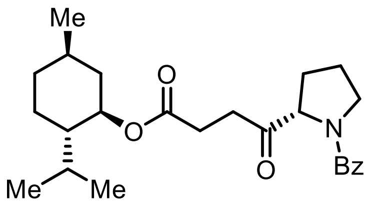

(*R*, *S*)-**L1**: 2:98 dr

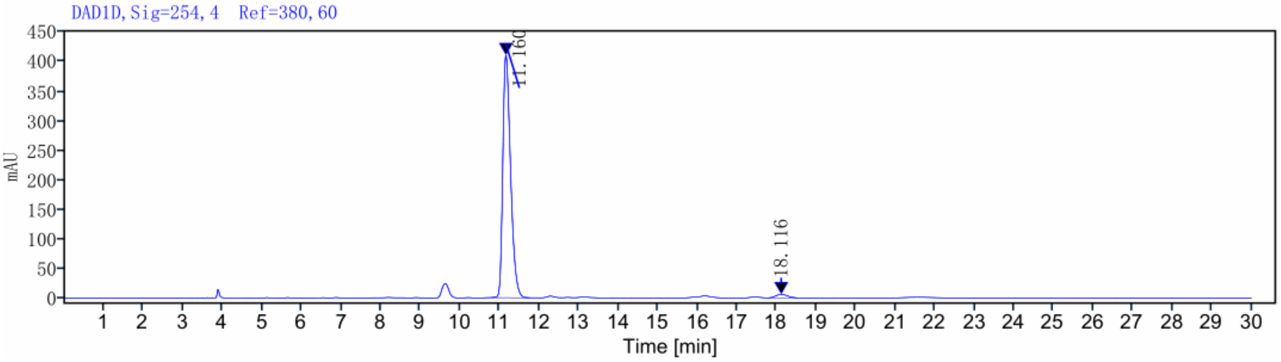

Signal: DAD1D, Sig=254, 4 Ref=380, 60

RetTime [min]	Type	Width [min]	Area [mAU*s]	Height [mAU]	Area %
11.160	VV	0.95853	5598.61851	410.81647	97.8161
18.116	BV	0.74961	124.99985	6.11826	2.1839
		Totals	5723.61836		

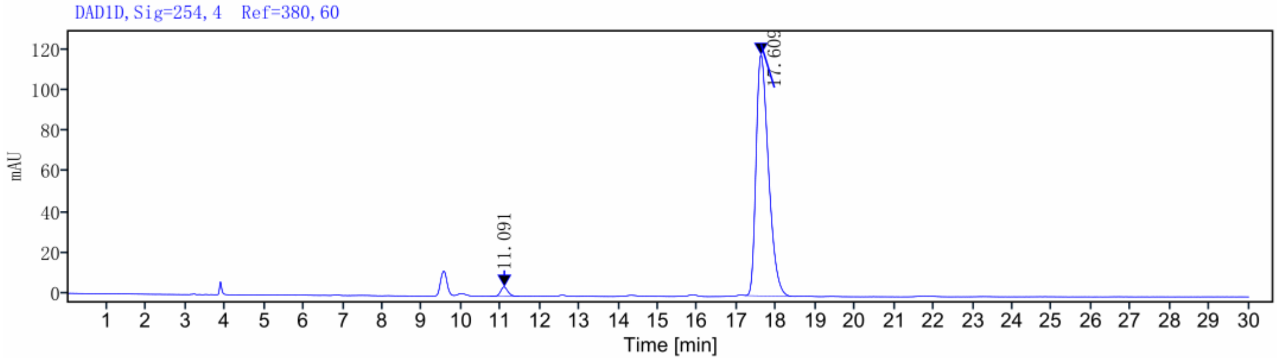

Signal: DAD1D, Sig=254, 4 Ref=380, 60

RetTime [min]	Type	Width [min]	Area [mAU*s]	Height [mAU]	Area %
11.091	MM m	0.74811	56.69769	4.58086	2.1231
17.609	BV	1.16462	2613.80501	119.15707	97.8769
		Totals	2670.50271		

Supplementary Fig. 125 HPLC data of **46** and **47**.

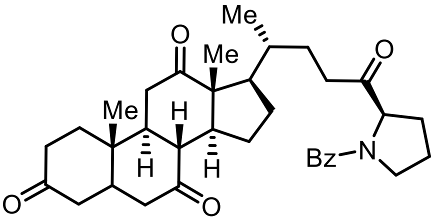

(*S*, *R*)-**L1**: 98:2 dr

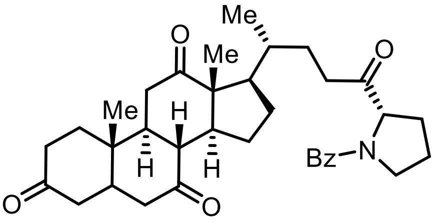

(*R*, *S*)-**L1**: 3:97 dr

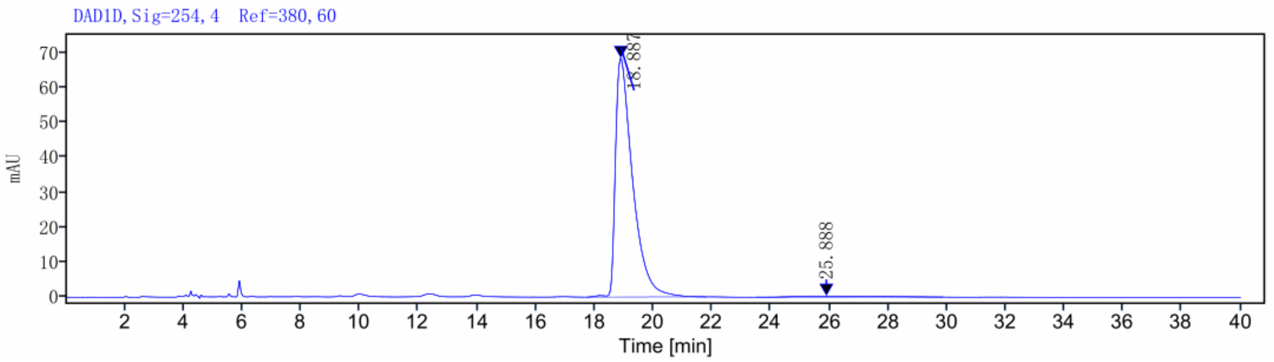

Signal: DAD1D, Sig=254, 4 Ref=380, 60

RetTime [min]	Type	Width [min]	Area [mAU*s]	Height [mAU]	Area %
18.887	MM m	4.02319	2774.18992	68.32968	98.2994
25.888	MM m	6.35066	47.99490	0.34877	1.7006
		Totals	2822.18482		

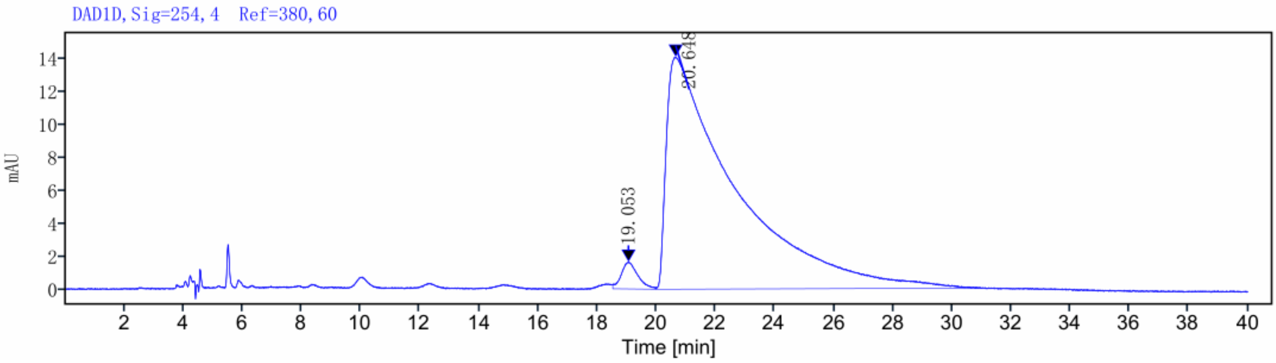

Signal: DAD1D, Sig=254, 4 Ref=380, 60

RetTime [min]	Type	Width [min]	Area [mAU*s]	Height [mAU]	Area %
19.053	MM m	1.46298	63.42910	1.64960	2.6969
20.648	MM m	10.90584	2288.53086	14.15401	97.3031
		Totals	2351.95996		

Supplementary Fig. 126 HPLC data of **48** and **49**.

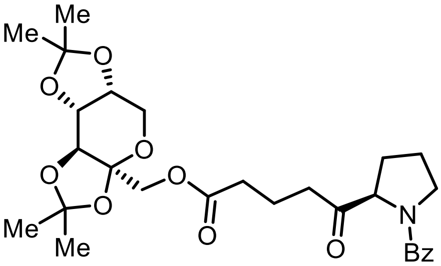

(*S*, *R*)-**L1**: 97:3 dr

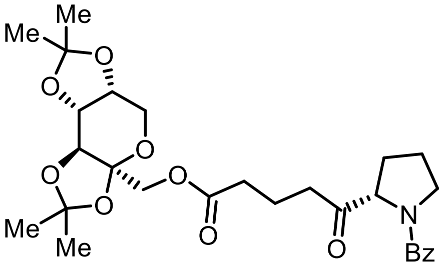

(*R*, *S*)-**L1**: 2:98 dr

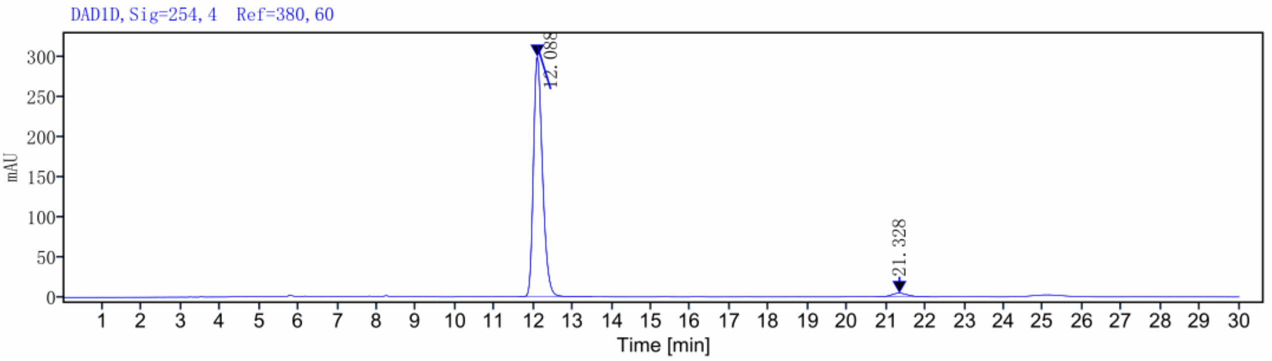

Signal: DAD1D, Sig=254, 4 Ref=380, 60

RetTime [min]	Type	Width [min]	Area [mAU*s]	Height [mAU]	Area %
12.088	MM m	1.89521	4844.25806	300.02346	97.3399
21.328	MM m	1.37153	132.38181	4.60519	2.6601
		Totals	4976.63987		

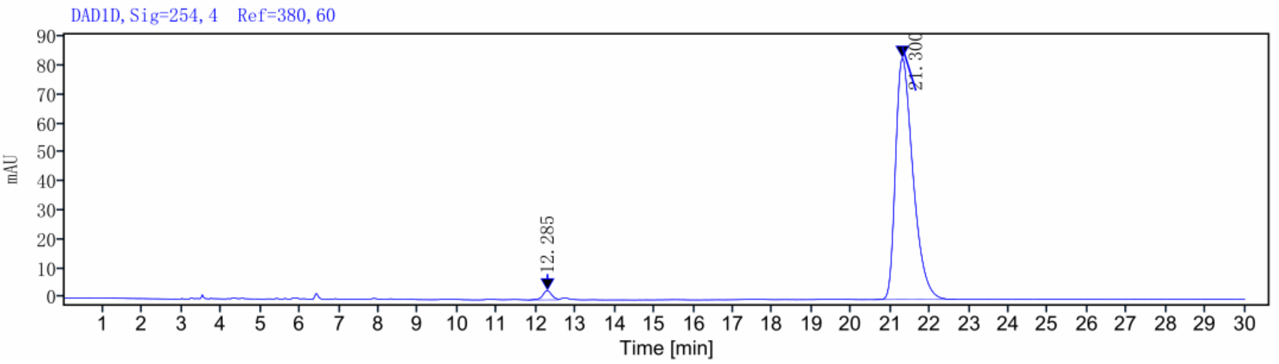

Signal: DAD1D, Sig=254, 4 Ref=380, 60

RetTime [min]	Type	Width [min]	Area [mAU*s]	Height [mAU]	Area %
12.285	MM m	0.77305	51.72620	3.20930	1.9739
21.300	MM m	2.04483	2568.78675	83.46187	98.0261
		Totals	2620.51295		

Supplementary Fig. 127 HPLC data of **50** and **51.**

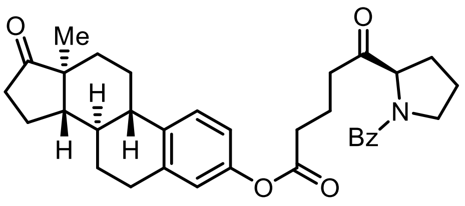

(*S*, *R*)-**L1**: 99:1 dr

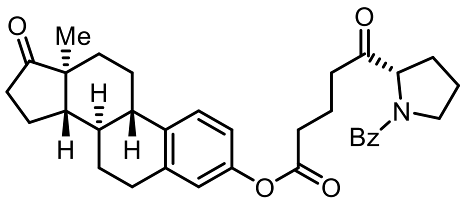

(*R*, *S*)-**L1**: 2:98 dr

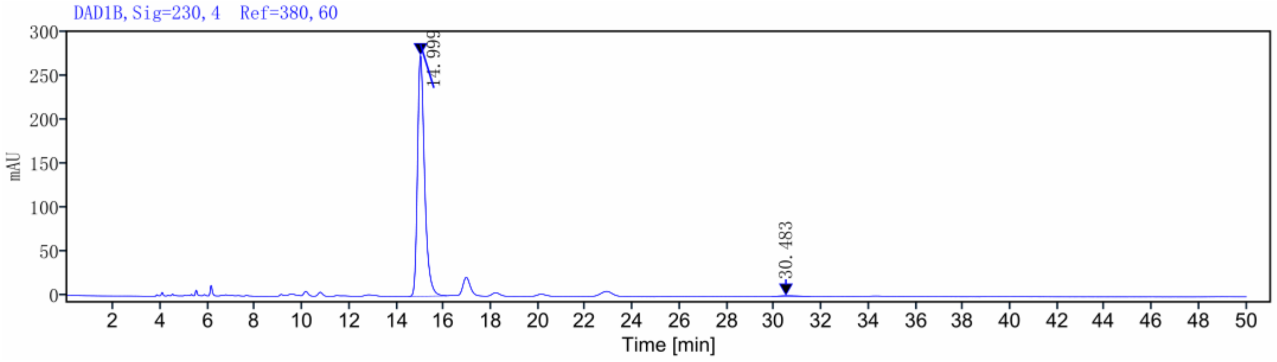

Signal: DAD1B, Sig=230, 4 Ref=380, 60

RetTime [min]	Type	Width [min]	Area [mAU*s]	Height [mAU]	Area %
14.999	BV	1.60905	5743.18656	274.64099	99.3091
30.483	MM m	1.63815	39.95544	0.80938	0.6909
		Totals	5783.14200		

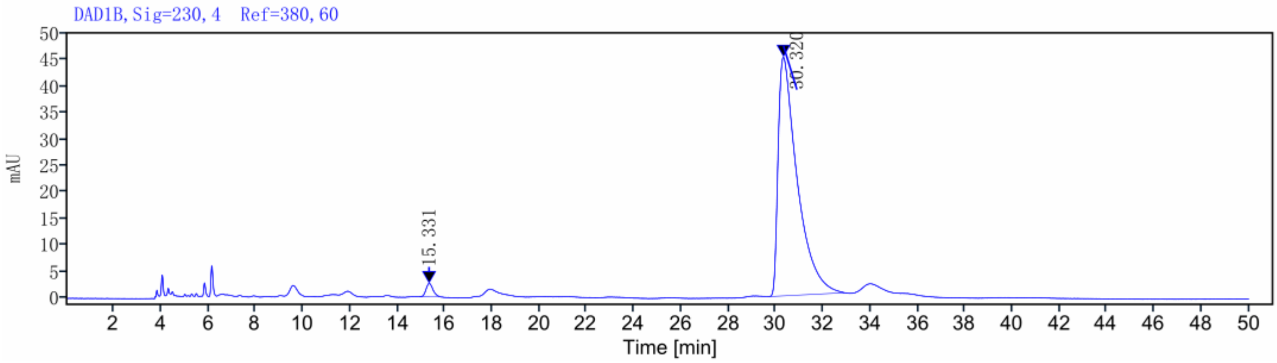

Signal: DAD1B, Sig=230, 4 Ref=380, 60

RetTime [min]	Type	Width [min]	Area [mAU*s]	Height [mAU]	Area %
15.331	MM m	0.85469	49.87604	2.48786	1.9366
30.320	VM m	3.12583	2525.54007	45.04071	98.0634
		Totals	2575.41611		

Supplementary Fig. 128 HPLC data of **52** and **53.**

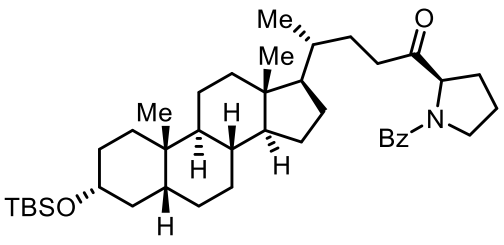

(*S, R*)-**L1**: 96:4 dr

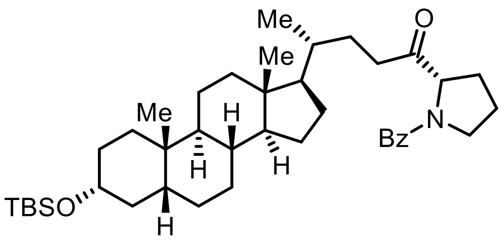

(*R, S*)-**L1**: 3:97 dr

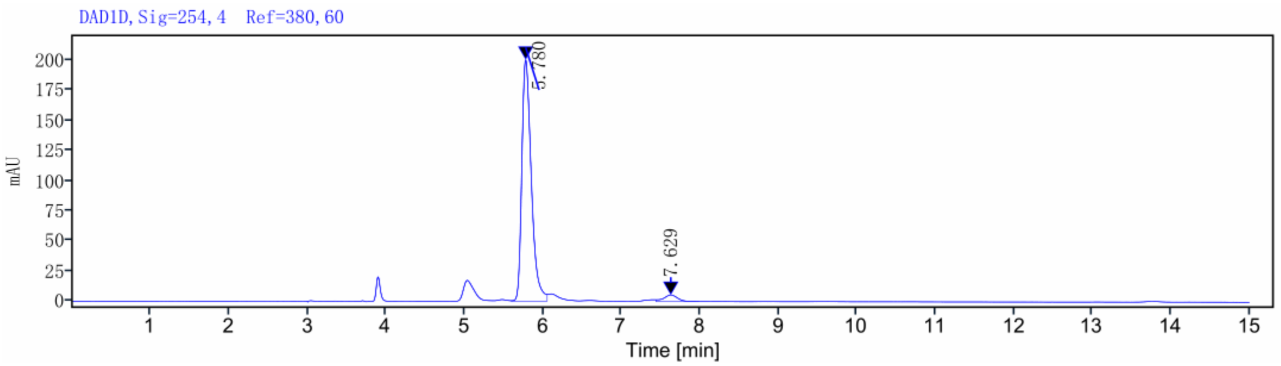

Signal: DAD1D, Sig=254, 4 Ref=380, 60

RetTime [min]	Type	Width [min]	Area [mAU*s]	Height [mAU]	Area %
5.780	VV	0.45887	1680.31053	200.10338	96.1278
7.629	VB	0.54751	67.68611	5.48072	3.8722
		Totals	1747.99664		

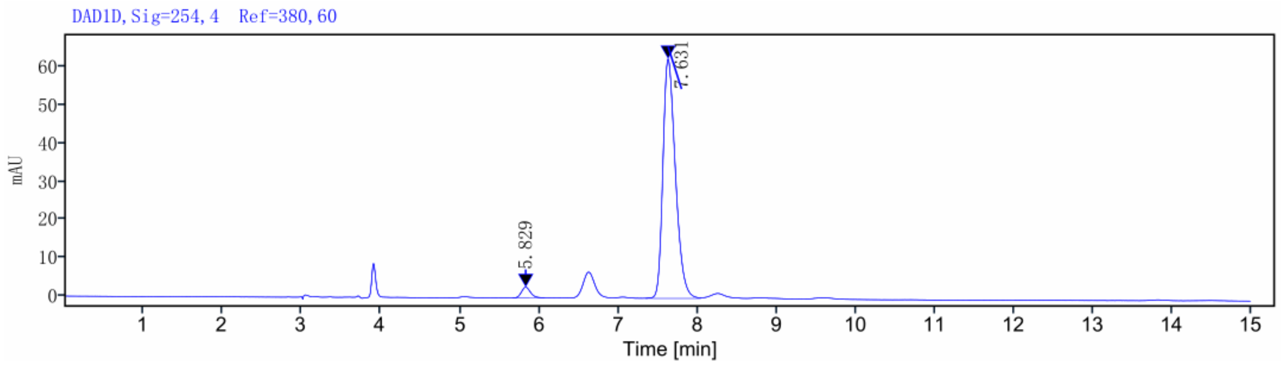

Signal: DAD1D, Sig=254, 4 Ref=380, 60

RetTime [min]	Type	Width [min]	Area [mAU*s]	Height [mAU]	Area %
5.829	VB	0.41217	22.26883	2.81679	3.1162
7.631	VV	0.69279	692.33852	62.92994	96.8838
		Totals	714.60736		

Supplementary Fig. 129 HPLC data of **54** and **55.**

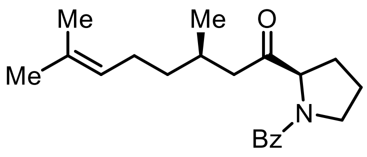

(*S*, *R*)-**L1**: 97:3 dr

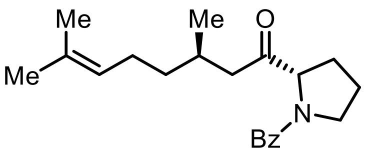

(*R*, *S*)-**L1**: 3:97 dr

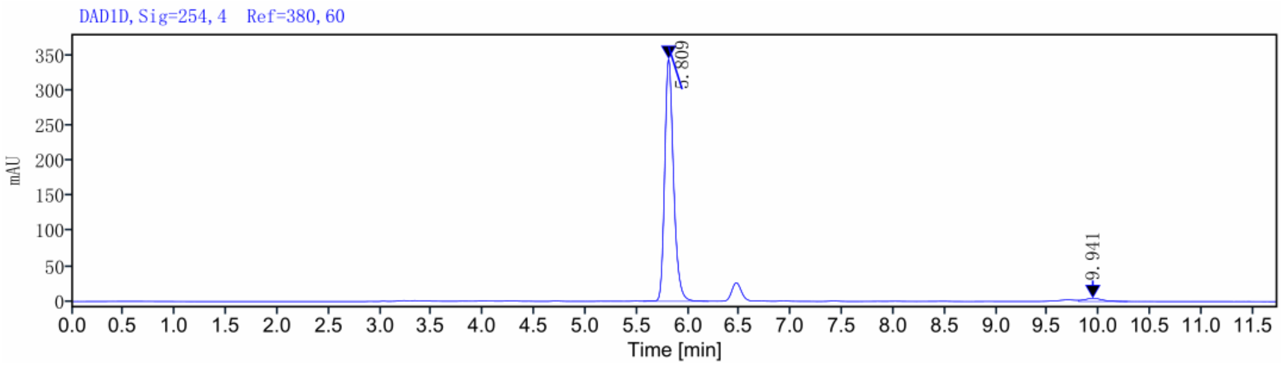

Signal: DAD1D, Sig=254, 4 Ref=380, 60

RetTime [min]	Type	Width [min]	Area [mAU*s]	Height [mAU]	Area %
5.809	VB	0.63260	2069.33096	344.28535	97.3441
9.941	VB	0.47866	56.45899	4.62971	2.6559
		Totals	2125.78995		

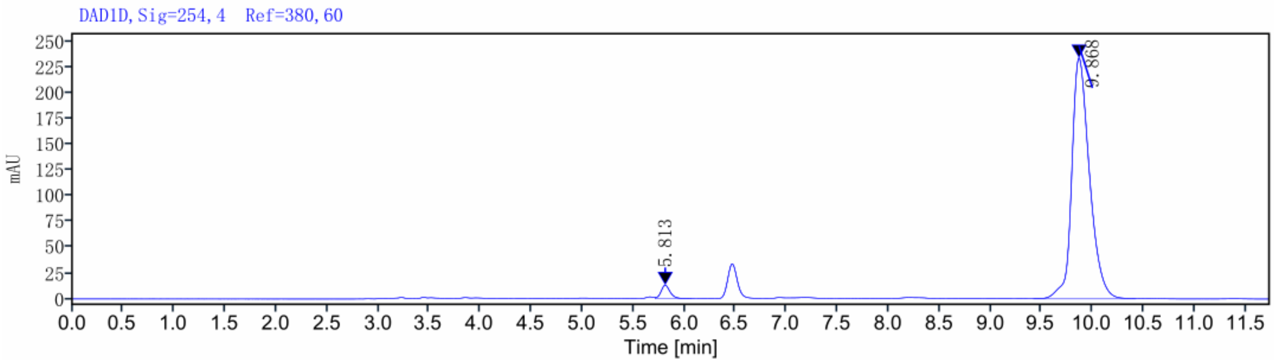

Signal: DAD1D, Sig=254, 4 Ref=380, 60

RetTime [min]	Type	Width [min]	Area [mAU*s]	Height [mAU]	Area %
5.813	VV	0.35635	82.04512	13.12213	2.8009
9.868	VV	1.00646	2847.20983	234.34811	97.1991
		Totals	2929.25495		

Supplementary Fig. 130 HPLC data of **56** and **57.**

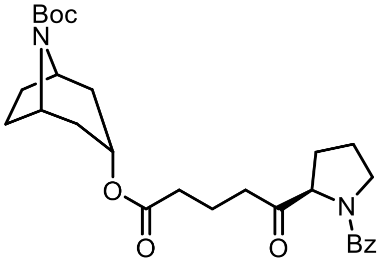

(*S*, *R*)-**L1**: 99:1 dr

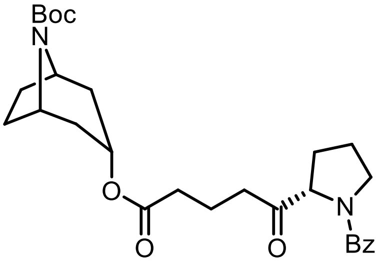

(*R*, *S*)-**L1**: 1:99 dr

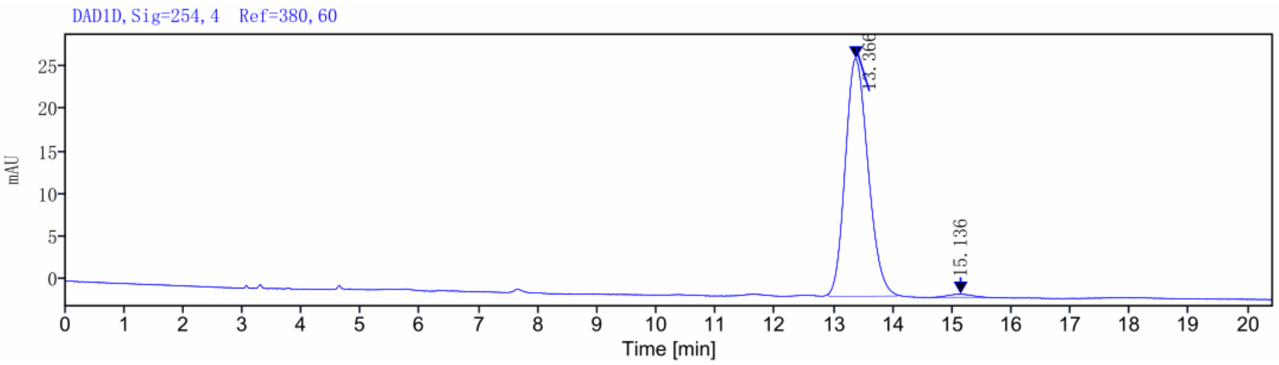

Signal: DAD1D, Sig=254, 4 Ref=380, 60

RetTime [min]	Type	Width [min]	Area [mAU*s]	Height [mAU]	Area %
13.366	VV	1.15598	747.61594	27.89144	98.6698
15.136	MM m	0.85355	10.07895	0.40539	1.3302
		Totals	757.69489		

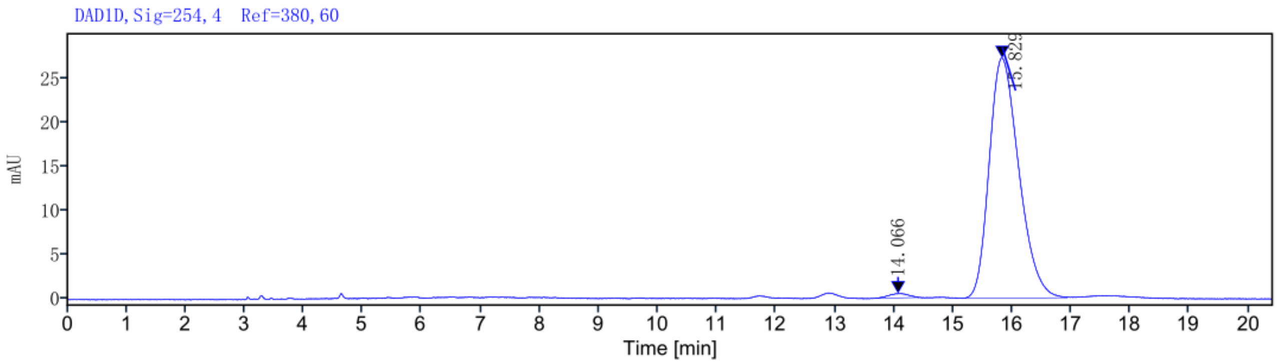

Signal: DAD1D, Sig=254, 4 Ref=380, 60

RetTime [min]	Type	Width [min]	Area [mAU*s]	Height [mAU]	Area %
14.066	MM m	0.73582	11.07518	0.50254	1.1570
15.829	VM m	1.71179	946.14787	27.10817	98.8430
		Totals	957.22304		

Supplementary Fig. 131 HPLC data of **58** and **59.**

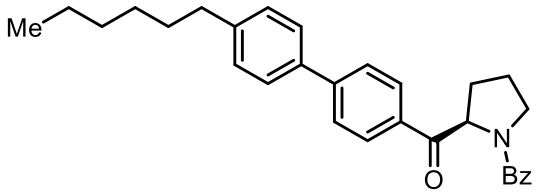

(*S*, *R*)-**L2**: 95% ee

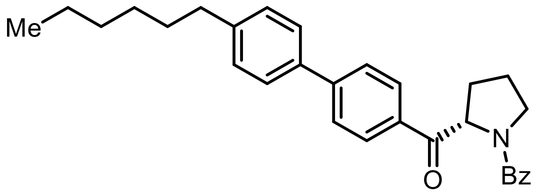

(*R*, *S*)-**L2**: 94% ee

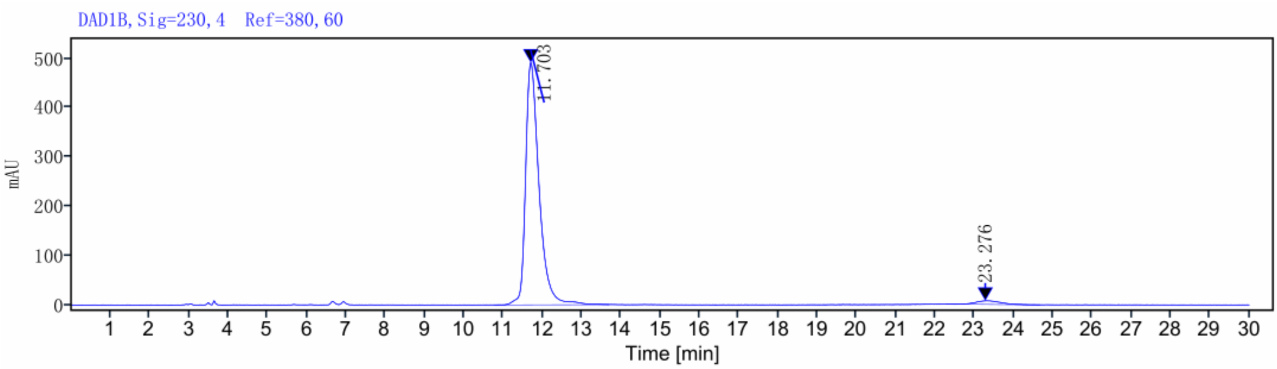

Signal: DAD1B, Sig=230, 4 Ref=380, 60

RetTime [min]	Type	Width [min]	Area [mAU*s]	Height [mAU]	Area %
11.703	MM m	2.92732	11727.16162	491.44861	97.3769
23.276	MM m	2.40734	315.90624	7.39323	2.6231
		Totals	12043.06786		

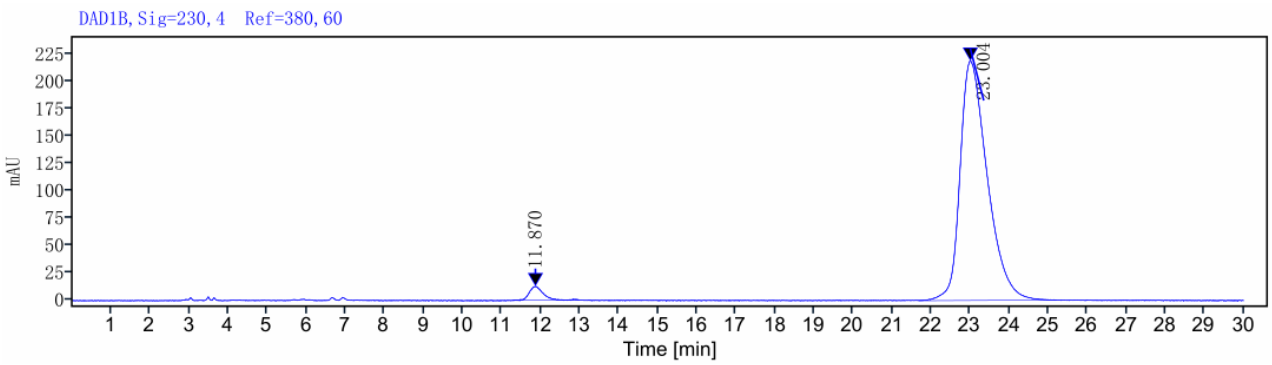

Signal: DAD1B, Sig=230, 4 Ref=380, 60

RetTime [min]	Type	Width [min]	Area [mAU*s]	Height [mAU]	Area %
11.870	MM m	1.69307	303.84372	12.74945	2.7844
23.004	MM m	3.51842	10608.34441	219.06971	97.2156
		Totals	10912.18812		

Supplementary Fig. 132 HPLC data of **60.**

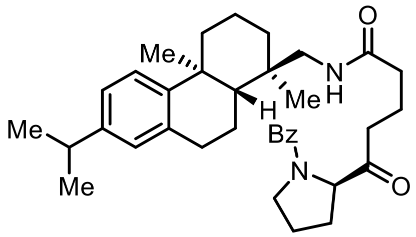

(S, R)-**L1**: 99:1 dr

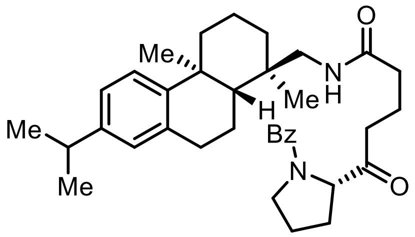

(R, S)-**L1**: 1:99 dr

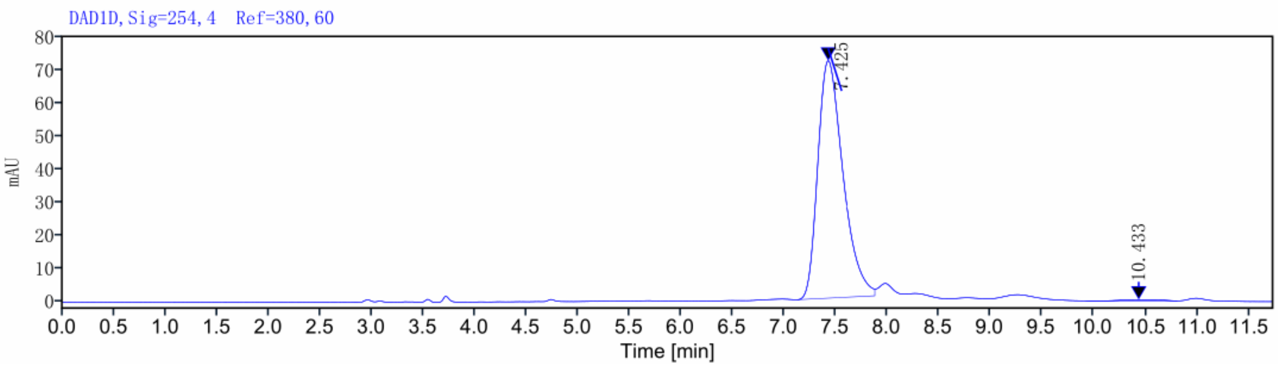

Signal: DAD1D, Sig=254, 4 Ref=380, 60

RetTime [min]	Type	Width [min]	Area [mAU*s]	Height [mAU]	Area %
7.425	BV	0.75106	1217.16340	71.66412	99.2268
10.433	MM m	0.88656	9.48476	0.38906	0.7732
		Totals	1226.64816		

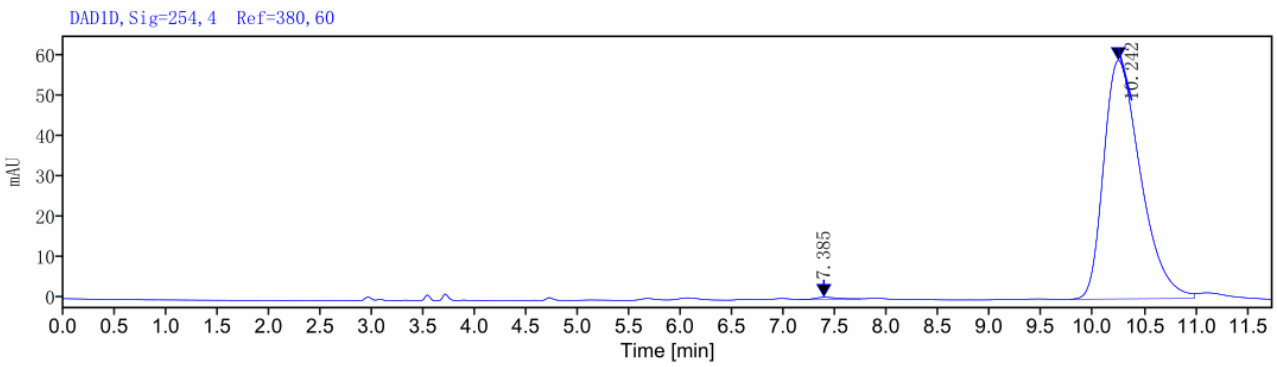

Signal: DAD1D, Sig=254, 4 Ref=380, 60

RetTime [min]	Type	Width [min]	Area [mAU*s]	Height [mAU]	Area %
7.385	MM m	0.59902	6.28652	0.47162	0.4278
10.242	BV	1.20348	1463.35403	58.78599	99.5722
		Totals	1469.64055		

Supplementary Fig. 133 HPLC data of **61** and **62.**

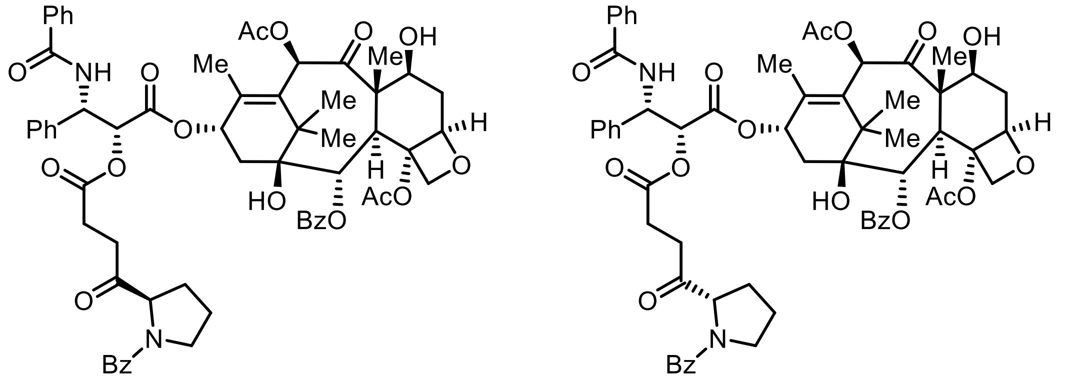

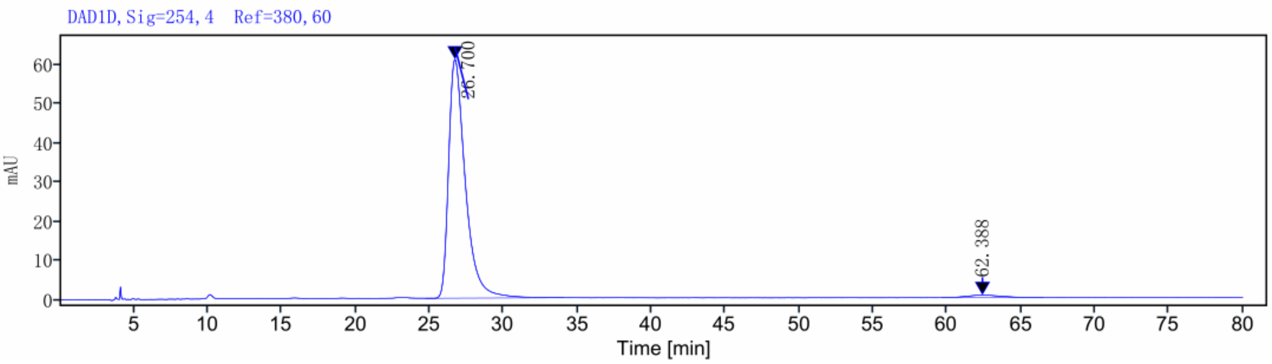

Signal: DAD1D, Sig=254, 4 Ref=380, 60

RetTime [min]	Type	Width [min]	Area [mAU*s]	Height [mAU]	Area %
26.700	MM m	9.38262	4783.57652	60.77019	97.9252
62.388	MM m	6.91351	101.35183	0.66165	2.0748
		Totals	4884.92835		

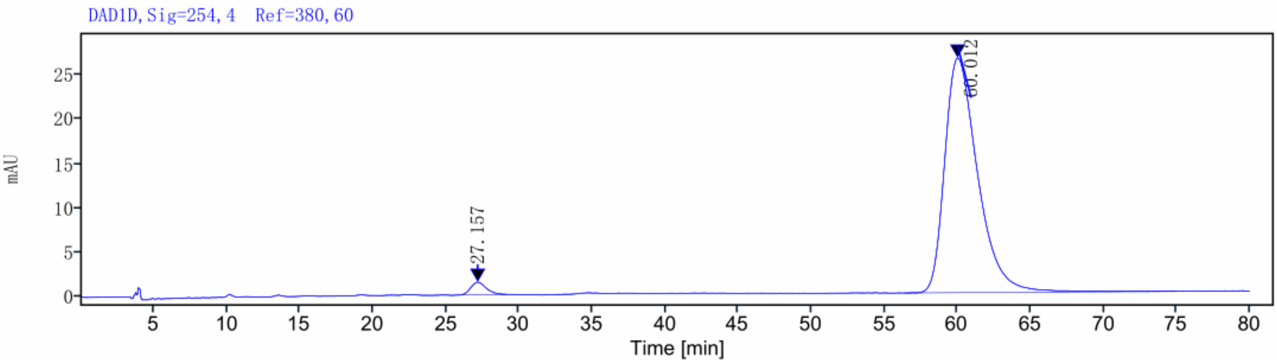

Signal: DAD1D, Sig=254, 4 Ref=380, 60

RetTime [min]	Type	Width [min]	Area [mAU*s]	Height [mAU]	Area %
27.157	MM m	3.03348	99.18794	1.40117	2.3390
60.012	MM m	18.69469	4141.37900	26.41786	97.6610
		Totals	4240.56694		

Supplementary Fig. 134 HPLC data of **63** and **64.**

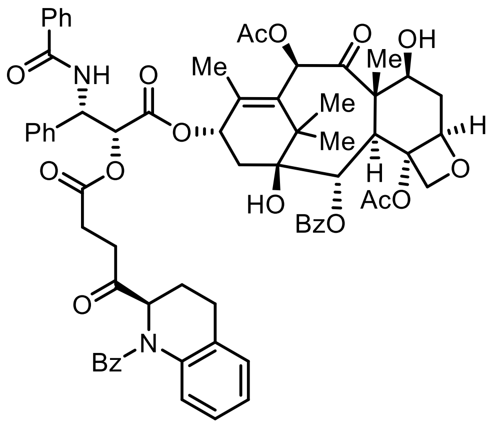

(*S*, *R*)-**L1**: 98:2 dr

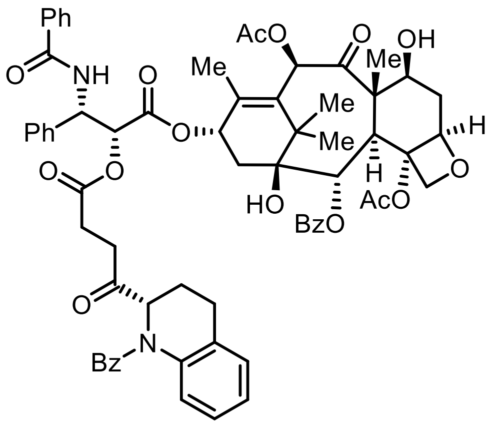

(*R*, *S*)-**L1**: 2:98 dr

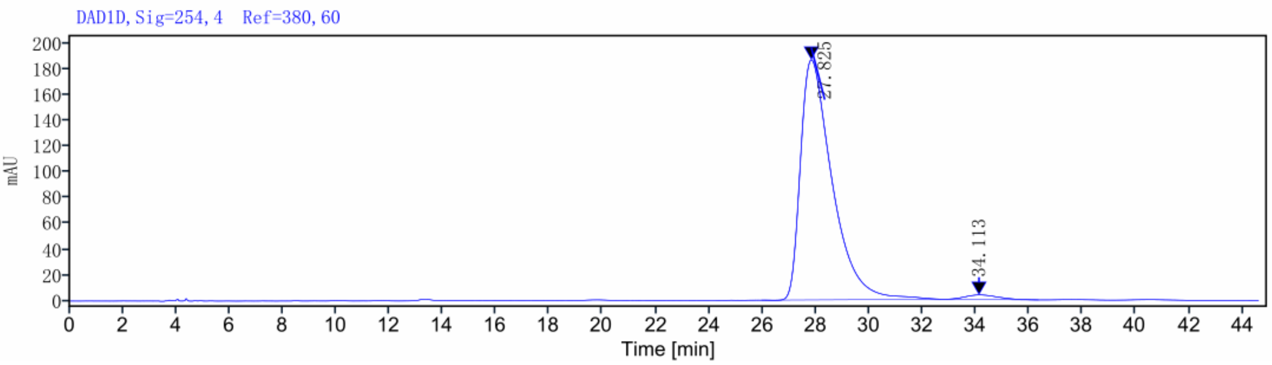

Signal: DAD1D, Sig=254, 4 Ref=380, 60

RetTime [min]	Type	Width [min]	Area [mAU*s]	Height [mAU]	Area %
27.825	MM m	7.00188	15388.58555	186.30619	98.1102
34.113	MM m	3.38293	296.40989	3.49214	1.8898
		Totals	15684.99544		

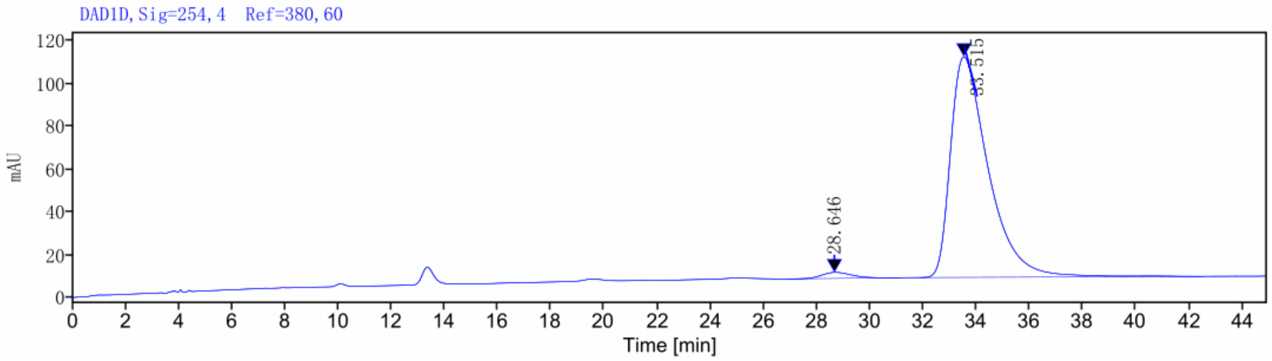

Signal: DAD1D, Sig=254, 4 Ref=380, 60

RetTime [min]	Type	Width [min]	Area [mAU*s]	Height [mAU]	Area %
28.646	MM m	4.23276	218.52909	2.84000	2.1074
33.515	MM m	10.58190	10151.25622	102.83549	97.8926
		Totals	10369.78531		

Supplementary Fig. 135 HPLC data of **65** and **66.**

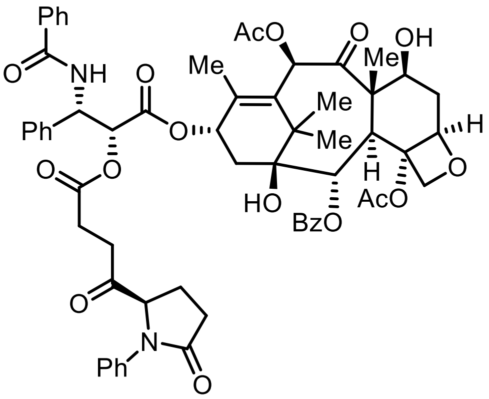

(*S*, *R*)-**L2**: 95:5 dr

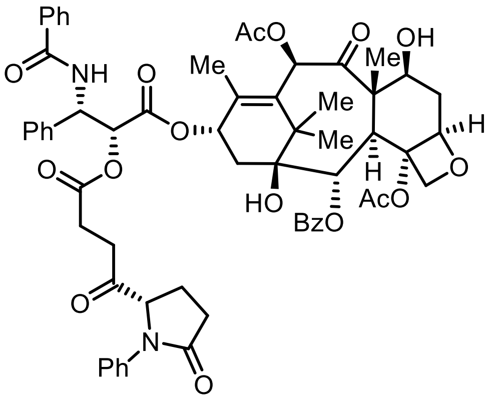

(*R*, *S*)-**L2**: 5:95 dr

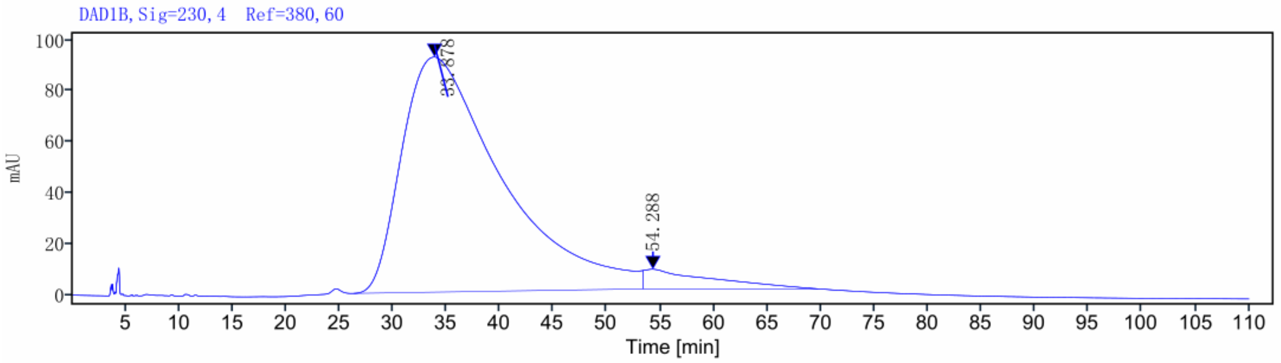

Signal: DAD1B, Sig=230, 4 Ref=380, 60

RetTime [min]	Type	Width [min]	Area [mAU*s]	Height [mAU]	Area %
33.878	MM m	27.00935	59989.48362	92.27735	94.7039
54.288	MM m	16.35514	3354.77289	7.71490	5.2961
		Totals	63344.25651		

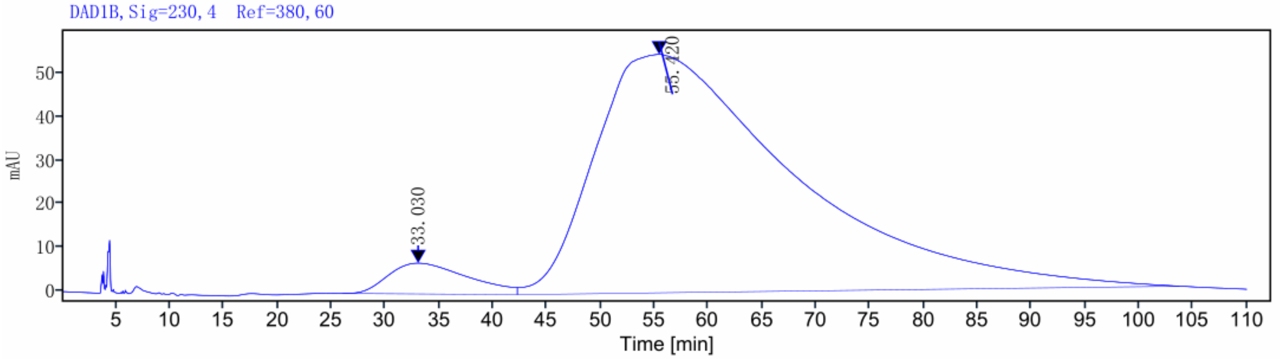

Signal: DAD1B, Sig=230, 4 Ref=380, 60

RetTime [min]	Type	Width [min]	Area [mAU*s]	Height [mAU]	Area %
33.030	MM m	16.26168	3633.15598	7.06149	4.8848
55.420	MM m	61.49533	70742.87116	54.59314	95.1152
		Totals	74376.02714		

Supplementary Fig. 136 HPLC data of **67** and **68.**

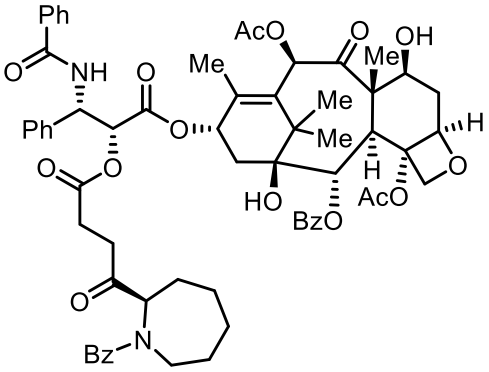

(*S*, *R*)-**L1**: 95:5 dr

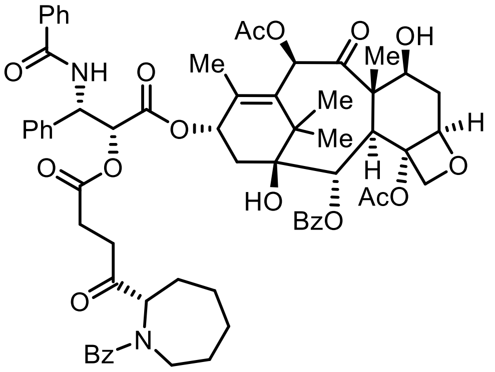

(*R*, *S*)-**L1**: 7:93 dr

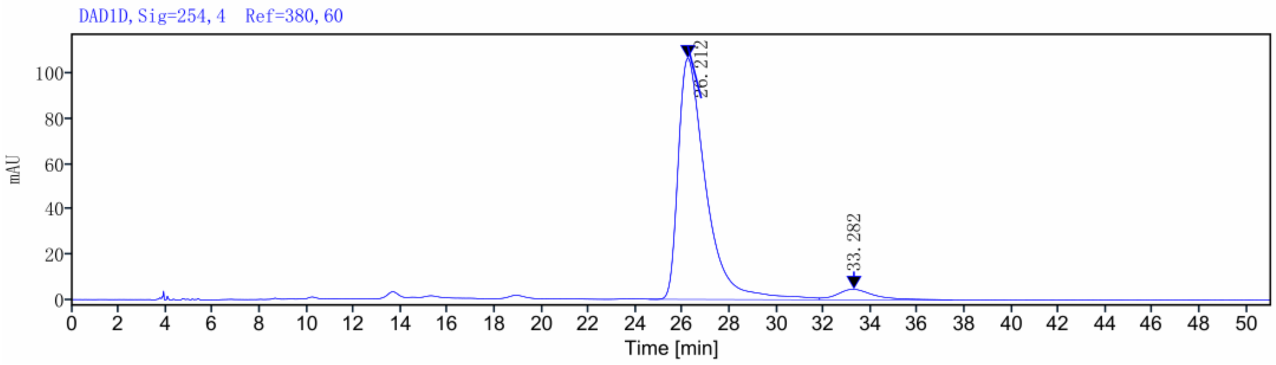

Signal: DAD1D, Sig=254, 4 Ref=380, 60

RetTime [min]	Type	Width [min]	Area [mAU*s]	Height [mAU]	Area %
26.212	MM m	7.25437	8775.45751	107.00809	94.7167
33.282	MM m	5.66572	489.49470	4.66956	5.2833
		Totals	9264.95221		

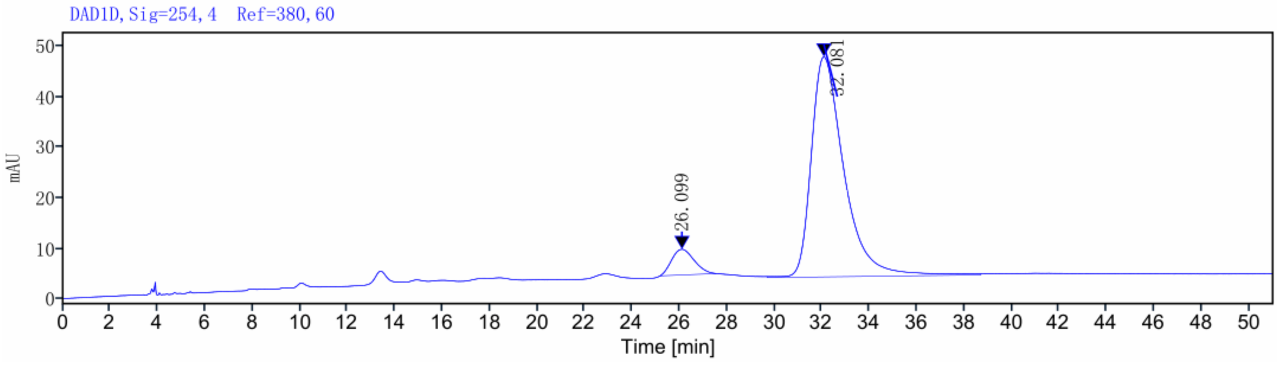

Signal: DAD1D, Sig=254, 4 Ref=380, 60

RetTime [min]	Type	Width [min]	Area [mAU*s]	Height [mAU]	Area %
26.099	MM m	2.36684	335.60840	5.10551	7.4203
32.081	MM m	9.00432	4187.21470	43.64354	92.5797
		Totals	4522.82310		

Supplementary Fig. 137 HPLC data of **69** and **70.**

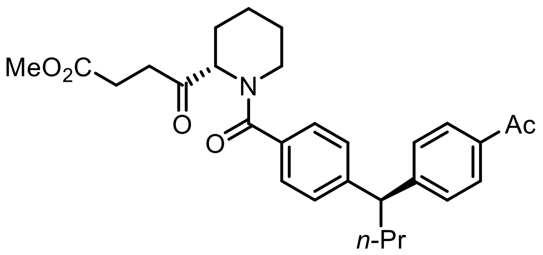

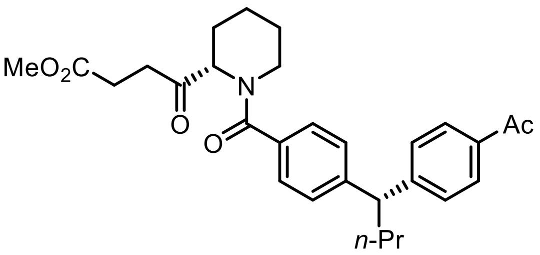

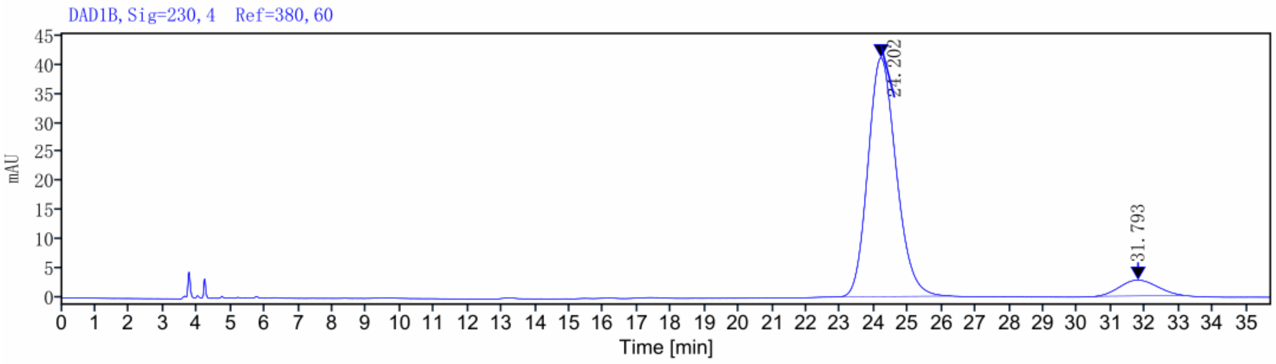

Signal: DAD1B, Sig=230, 4 Ref=380, 60

RetTime [min]	Type	Width [min]	Area [mAU*s]	Height [mAU]	Area %
24.202	VM m	3.22008	2390.04028	41.13919	91.9664
31.793	MM m	2.60721	208.77855	2.73659	8.0336
		Totals	2598.81883		

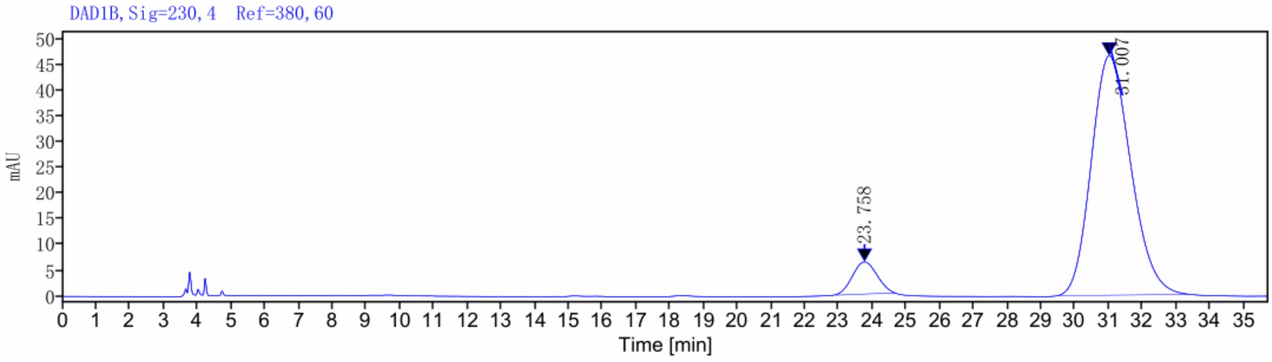

Signal: DAD1B, Sig=230, 4 Ref=380, 60

RetTime [min]	Type	Width [min]	Area [mAU*s]	Height [mAU]	Area %
23.758	VM m	1.74700	316.41971	6.27877	7.6970
31.007	VM m	3.87090	3794.54717	46.64473	92.3030
		Totals	4110.96688		

Supplementary Fig. 138 HPLC data of **71** and **72.**

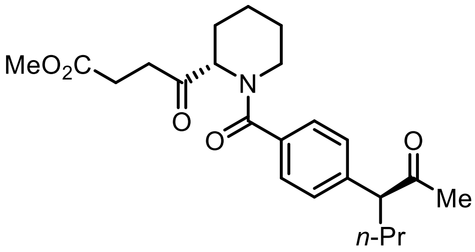

(*S*)-**L3**: 97:3 dr

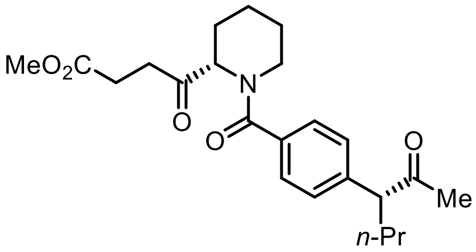

(*R*)-**L3**: 2:98 dr

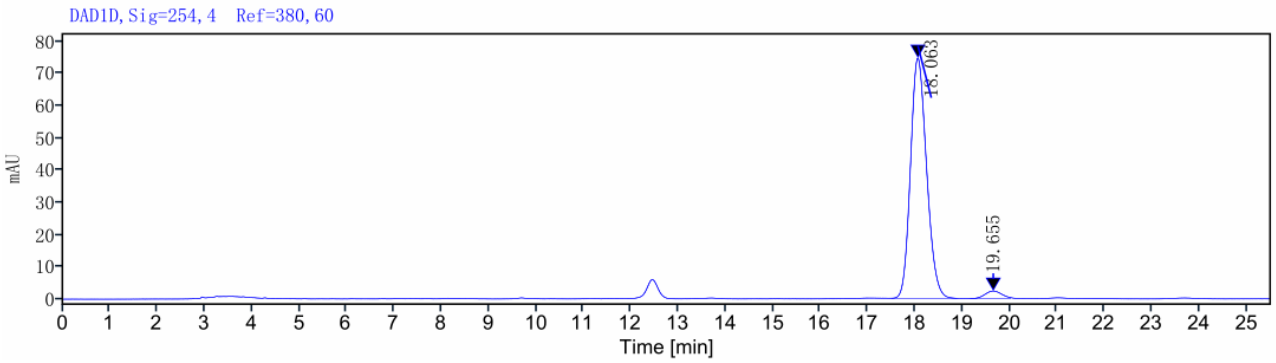

Signal: DAD1D, Sig=254, 4 Ref=380, 60

RetTime [min]	Type	Width [min]	Area [mAU*s]	Height [mAU]	Area %
18.063	VV	1.38008	1767.31892	74.70148	96.8406
19.655	BV m	0.84835	57.65918	2.37505	3.1594
		Totals	1824.97810		

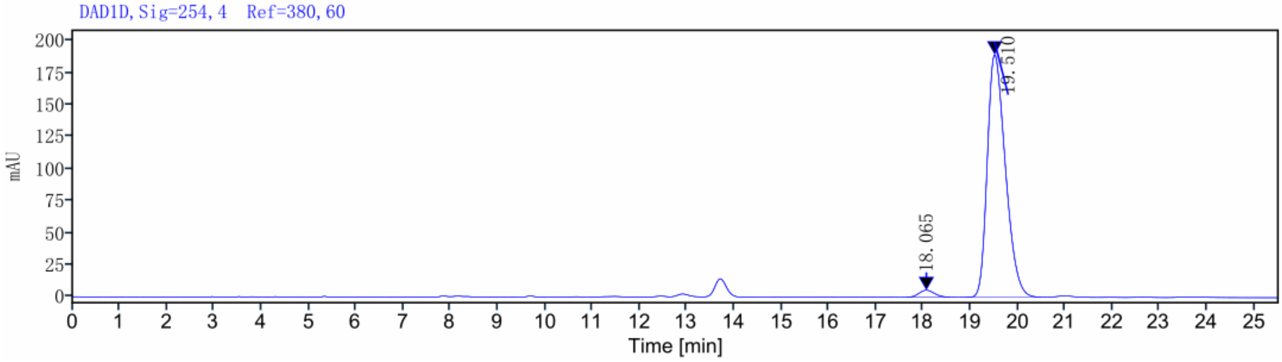

Signal: DAD1D, Sig=254, 4 Ref=380, 60

RetTime [min]	Type	Width [min]	Area [mAU*s]	Height [mAU]	Area %
18.065	VV	0.91903	129.84812	5.61779	2.4959
19.510	VV	1.55511	5072.63788	190.08166	97.5041
		Totals	5202.48600		

Supplementary Fig. 139 HPLC data of **73** and **74.**

II. Supplementary References

1. Aubineau, T. & Cossy, J. Chemoselective alkynylation of *N*-sulfonylamides *versus* amides and carbamates – synthesis of tetrahydropyrazines. *Chem. Commun.* **49**, 3303−3305 (2013).

2. Cheng, X., Lu, H. & Lu, Z. Enantioselective benzylic C–H arylation via photoredox and nickel dual catalysis. *Nat. Commun.* **10**, 3549 (2019).

3. Shu, X., Zhong, D., Lin, Y., Qin, X. & Huo, H. Modular access to chiral α-(hetero)aryl amines via Ni/photoredox-catalyzed enantioselective cross-coupling. *J. Am. Chem. Soc.* **144**, 8797–8806 (2022).

4. Huan, L., Shu, X., Zu, W., Zhong, D. & Huo, H. Asymmetric benzylic C(sp^3)−H acylation via dual nickel and photoredox catalysis. *Nat. Commun.* **12**, 3536 (2021).

5. Sun, Z., Kumagai, N. & Shibasaki, M. Photocatalytic α–acylation of ethers. *Org.Lett.* **19**, 3727−3730 (2017).

6. Dolomanov, O. V., Bourhis, L. J., Gildea, R. J., Howard, J. A. K. & Puschmann, H. OLEX2: A complete structure solution, refinement and analysis program. *J. Appl. Cryst.* **42**, 339−341 (2009).

7. Bourhis, L. J., Dolomanov, O. V., Gildea, R. J., Howard, J. A. K. & Puschmann, H. The anatomy of a comprehensive constrained, restrained refinement program for the modern computing environment - Olex2 dissected. *Acta Cryst.* **A 71**, 59−75 (2015).

8. Sheldrick, G. M. Crystal structure refinement with SHELXL. *Acta Cryst.* **C 71**, 3−8 (2015).
